# A novel cuproptosis-related molecular pattern and its tumor microenvironment characterization in colorectal cancer

**DOI:** 10.3389/fimmu.2022.940774

**Published:** 2022-09-30

**Authors:** Zhonglin Zhu, Qiuyan Zhao, Wang Song, Junyong Weng, Shanbao Li, Tianan Guo, Congcong Zhu, Ye Xu

**Affiliations:** ^1^ Department of Colorectal Surgery, Fudan University Shanghai Cancer Center, Shanghai, China; ^2^ Department of Oncology, Shanghai Medical College, Fudan University, Shanghai, China; ^3^ Department of Gastroenterology, Shanghai General Hospital, Shanghai Jiao Tong University School of Medicine, Shanghai, China; ^4^ Shanghai Key Laboratory of Pancreatic Diseases, Shanghai General Hospital, Shanghai Jiao Tong University School of Medicine, Shanghai, China; ^5^ Department of General Surgery, Shanghai General Hospital, Shanghai Jiao Tong University School of Medicine, Shanghai, China

**Keywords:** cuproptosis, tumor microenvironment, immunotherapy, colorectal cancer, molecular subtype

## Abstract

Cuproptosis, or copper-induced cell death, has been reported as a novel noncanonical form of cell death in recent times. However, the potential roles of cuproptosis in the alteration of tumor clinicopathological features and the formation of a tumor microenvironment (TME) remain unclear. In this study, we comprehensively analyzed the cuproptosis-related molecular patterns of 1,274 colorectal cancer samples based on 16 cuproptosis regulators. The consensus clustering algorithm was conducted to identify cuproptosis-related molecular patterns and gene signatures. The ssGSEA and ESTIMATE algorithms were used to evaluate the enrichment levels of the infiltrated immune cells and tumor immune scores, respectively. The cuproptosis score was established to assess the cuproptosis patterns of individuals with principal component analysis algorithms based on the expression of cuproptosis-related genes. Three distinct cuproptosis patterns were confirmed and demonstrated to be associated with distinguishable biological processes and clinical prognosis. Interestingly, the three cuproptosis patterns were revealed to be consistent with three immune infiltration characterizations: immune-desert, immune-inflamed, and immune-excluded. Enhanced survival, activation of immune cells, and high tumor purity were presented in patients with low cuproptosisScore, implicating the immune-inflamed phenotype. In addition, low scores were linked to high tumor mutation burden, MSI-H and high CTLA4 expression, showing a higher immune cell proportion score (IPS). Taken together, our study revealed a novel cuproptosis-related molecular pattern associated with the TME phenotype. The formation of cuproptosisScore will further strengthen our understanding of the TME feature and instruct a more personalized immunotherapy schedule in colorectal cancer.

## Introduction

Colorectal cancer is the third most common cancer and the second most deadly cancer worldwide (1, 2). With the spread of multiple-disciplinary treatment, the death rate gradually declined from declining 3% per year during the 2000s to declining 1.8% per year from 2012 to 2017 (3). The overall 5-year survival of colorectal cancer has been more than 60% in recent years. However, it decreases to 14% for patients with distant metastases (3). Therefore, in-depth understanding of the multiple tumor features and identifying effective prognostic indicators contribute to constructing a more significant therapy schedule for the individual person.

Copper is an essential trace element for eukaryotes. It is involved in numerous fundamental biological processes, such as iron transport, oxygen radical detoxification, and mitochondrial respiration (4). Intracellular copper concentration is in a state of dynamic balance based on the across homeostatic gradients, in which the dynamic signaling influences a diverse number of cellular processes including lipolysis, proliferation, and autophagy (5–9). Dancis et al. (10) and Knight et al. (11) discovered the copper transport proteins 1–5 (Ctr1–5), especially Ctr1 (SLC31A1), with the strongest copper transport capacity. In addition, the excretion of copper irons is mediated by P-type ATPases (ATP7A, ATP7B), whose N-terminal possesses metal binding sites (12). Due to the dysregulation of transmembrane transport of copper, the accumulation of intracellular copper results in cytotoxicity and cell death (13). Nevertheless, the detailed mechanism of copper-induced cell death remains uncertain. It has been reported that excessive copper elevated the level of intracellular reactive oxygen species (ROS), induced endoplasmic reticulum (ER) stress, enhanced damage-associated molecular patterns (DAMPs), and promoted phagocytosis by macrophages (14). In recent, a novel mechanism of copper-induced cell death has been uncovered on *Science*: copper directly binds to lipoylated proteins of mitochondrial tricarboxylic acid (TCA) cycle, then lipoylated protein aggregation, loss of Fe–S cluster and induction of HSP70 lead to proteotoxic stress and cell death (15). The ancient protein lipoylation mechanism of copper homeostasis presents a novel pathway of cell death, termed cuproptosis, which is distinguishable from other known mechanisms including necrosis (16), apoptosis (17), autophagy (18), necroptosis (19), pyrotosis (20), and ferroptosis (21). Also, cuproptosis provides a new sight in disease treatment (22). For Wilson’s disease (23) and Menke’s disease (24) derived from copper homeostasis dysregulation, less import or more export is an ideal therapy. In several cancer types, copper has been found at higher levels in both serum and tissues compared with those of normal people (25–28). It is evident that intracellular copper accumulation contributes to tumor cell proliferation, angiogenesis, and metastasis (29, 30). However, how to dissect the correlation of cuproptosis with tumor promotion of copper accumulation and how to employ copper toxicity in clinical tumor therapy needs to be further exploited.

Increasing evidence has shown that the tumor microenvironment (TME) is widely involved in tumor development and progression, chemoradiotherapy and immune therapy (31–33). TME is mainly composed of tumor cells and stromal components, of which stromal components consist of residential fibroblasts, endothelial cells, infiltrating immune cells, secreted cytokines and chemokines, and nascent blood and lymphatic vessels. TME is diverse, complex, and plastic toward both phenotypes of tumor promotion and immune escape or tumor suppression and immune enhancement (34–36). For example, macrophages, accounting for the majority of tumor stromal cells, can be molded into classically (M1) or (M2) activated cells in different TMEs. M1 macrophages exert pro-inflammatory and antitumor roles, while M2 macrophages exhibit high levels of anti-inflammatory cytokines and promote cancer cell growth and metastasis (37). Caner-associated fibroblasts (CAFs) are also a main component of TME and play dual roles through matrix deposition and remodeling and extensive crosstalk with cancer cells and infiltrating leukocytes (38). According to the diverse components, TME is widely accepted to be classified into three subtypes: immune-desert, immune-inflamed, and immune-excluded (39). With more and more focus on TME, researchers have found that TME is closely associated with the prognosis of multiple tumors and response to chemoradiotherapy and immunotherapy, such as colorectal cancer, melanoma, gastric cancer, and intrahepatic cholangiocarcinoma (40–44). In colorectal cancer, the high level of infiltrated cytotoxic CD8^+^ T cells at the center or margin of the tumor predicts a low risk of recurrence at 5 years (45). Considering the different TME components in an individual patient, there is an urgent need to thoroughly analyze the TME infiltration patterns to administer a better therapy regime.

In this study, we systemically integrated the expression profiles of 1,274 colorectal cancer samples to evaluate the cuproptosis-related molecular patterns. Further analysis confirmed the close relationships between the three cuproptosis patterns and TME infiltration characteristics. Based on the differentially expressed genes, patients were classified into three gene clusters. Finally, a novel cuproptosis score system was constructed to characterize the TME phenotype, which may serve as a biomarker of prognosis evaluation and a target for immunotherapy in colorectal cancer.

## Materials and methods

### Colorectal cancer data sources

The process of this work is exhibited in **Figure S1A**. RNA-sequencing data, clinical annotation, and survival time of colon cancer and rectal cancer were downloaded from The Cancer Genome Atlas (TCGA database, https://portal.gdc.cancer.gov/) and the Gene Expression Omnibus (GEO database, https://www.ncbi.nlm.nih.gov/geo/). Transcriptome profiles of 689 samples in the TCGA-colon adenocarcinoma/rectum adenocarcinoma (COAD/READ) were obtained in the format of fragments per kilobase million (FPKM), including 51 normal samples and 638 cancer samples. Then, FPKM values were transformed into transcripts per kilobase million (TPM) for identical analysis with the GEO data (GSE39582, 19 normal samples, and 566 cancer samples) (46). The three datasets were merged with a combat algorithm to correct batch effects with the ‘sva’ R package. All the data were analyzed with the R program (version 4.1.2).

### Unsupervised consensus cluster for cuproptosis regulators

A total of 16 cuproptosis regulators were retrieved, including 13 regulators of the lipoylated TCA cycle pathway (FDX1, LIPT1, LIAS, DLD, MTF1, GLS, CDKN2A, DLAT, PDHA1, PDHB, DBT, GCSH, and DLST) (15) and three copper transport proteins (SLC31A1, ATP7A, and ATP7B) (10–12). Based on their roles in the lipoylated TCA cycle pathway, these regulators were classified into four groups: seven upregulators, three downregulators, three enzymes, and three carriers. According to the expression of these genes, the unsupervised consensus clustering analysis was employed to classify these samples into three distinct molecular patterns with the R package ‘ConsensusClusterPlus’.

### Gene set variation analysis and functional annotation

GSVA was performed to detect the different biological functions between the distinct cuproptosis clusters with the R package ‘GSVA’. The gene sets of hallmark gene sets (v7.5.1) derived from the MSigDB database were used for GSVA analysis (47). Function annotation of a gene list was analyzed by KEGG_Pathway with the Bioconductor package “clusterProfiler” (48) and by the Metascape database (https://metascape.org/).

### Three ways of TME cell infiltration analysis

The single-sample gene set enrichment analysis (ssGSEA) was performed to evaluate the relative immune cell infiltration and immune functions in each sample (49). The stromal score and tumor purity of each sample were quantified with the ESTIMATE algorithm. The consensus molecular subtype (CMS) of colorectal cancer was computed with the ‘CMScaller’ package. CMS1 is classified as a microsatellite unstable and immune activated phenotype, which is characterized by mismatch repair gene mutation and microsatellite instability. CMS4 is a stromal type, consistent with an immune-exclude phenotype. CMS2 is a classic type, with abnormal activation of Wnt and myc signaling pathways and significant variation in somatic copy number. CMS3 is a metabolic type with a high mutation rate of KRAS. Relatively, CMS2 and CMS3 are classified as immune-desert phenotypes.

### Differentially expressed genes between the three cuproptosis clusters and establishment of cuproptosis gene signature

To identify cuproptosis-related genes, DEGs between the three cuproptosis clusters were compared in pairs three times with the R package ‘limma’ (significant criteria, adjusted P-value <0.01). The intersect genes of the three DEGs were next employed to generate cuproptosis gene signatures and to construct a cuproptosis score system to assess the cuproptosis pattern of individuals. First, 965 intersect genes were subjected to GO and KEGG enrichment analysis to explore potential functions and pathways. Next, univariate Cox regression analysis for each gene was employed to filter genes with significant prognostic correlation. Then, the unsupervised consensus clustering analysis was employed again to classify these samples into distinct cuproptosis gene signature patterns with the R package ‘ConsensusClusterPlus’. Finally, principal component analysis (PCA) was carried out to separate the cuproptosis gene signature patterns. This algorithm makes full use of the score on the set with the largest block of well-correlated (or anticorrelated) genes. Also, the algorithm downweights attributions that do not track other set members. Followed by obtaining the prognostic value of each gene, the cuproptosisScore was defined with principal components 1 and 2, similar to the gene expression grade index (GGI) (50, 51): cuproptosisScore = ∑(PC1_i_ + PC2_i_). PC1_i_ and PC2_i_ represent the expression score of each intersecting gene in two dimensions, respectively.

### Somatic mutation, copy number variation, microsatellite instability, and immune cell proportion score analysis

The somatic mutation data of TCGA-COAD/READ was accessed from the TCGA database in varscan file format. The CNV data were downloaded from the UCSC Xena (https://xenabrowser.net/datapages/). The significant mutated genes and tumor mutation burden (TMB) were calculated using the R package ‘maftool’. The percentages of microsatellite stability (MSS), high microsatellite instability (MSI-H) and low microsatellite instability (MSI-L) were computed in the different cuporptosis scoring groups. The IPS data were accessed from The Cancer Immunome Altas (https://tcia.at/home). The IPS scores of anti-CTLA4 drugs were compared between the cuporptosis score groups.

### Kaplan–Meier survival analysis

The Kaplan–Meier survival curves were plotted and analyzed by the R package ‘Survminer’. The samples were stratified into different subgroups with different gene expression, cuproptosisCluster subgroups, geneCluster subgroups, cuproptosisScore, and TMB.

### Statistical analyses

All the data analysis was performed using R software (version 4.1.2) and GraphPad Prism (version 9.2). The measurement data were expressed as ( x ± s), and the t-test was used for the comparison of two groups, while analysis of variance was used for the comparison of more than two groups. The count data were compared between groups by χ^2^ test. The statistical significance level was set at a P-value of <0.05.

## Results

### Landscape of genetic variation of cuproptosis regulators in colorectal cancer

**Figure 1:**
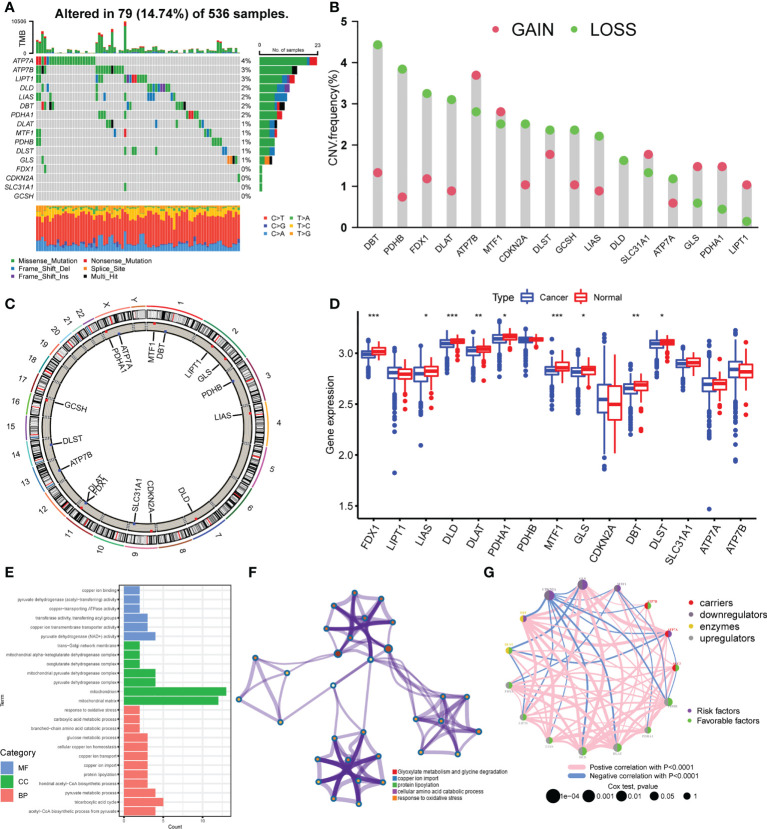
Landscape of genetic variation of cuproptosis regulators in colorectal cancer. **(A)** The mutation frequency of 16 cuproptosis regulators in 536 samples from TCGA-COAD/READ. **(B)** The CNV variation frequency of 16 cuproptosis regulators in TCGA-COAD/READ. **(C)** The location diagram of CNV of 16 cuproptosis regulators on 23 chromosomes. **(D)** The expression of 16 cuproptosis regulators in cancer and normal samples of TCGA-COAD/READ. **(E)** Go analysis results of 16 cuproptosis regulators **(F)**. Metascape analysis of 16 cuproptosis regulators **(G)**. The overall landscape of the interaction between cuproptosis regulators and the prognostic significance of the regulators in colorectal cancer patients. *P <0.05; **P <0.01; ***P <0.001.

A total of 16 cuproptosis regulators, including seven upregulators, three downregulators, three enzymes, and three carriers, were analyzed in this study. We first summarized the incidences of somatic mutation of 16 cuproptosis regulators in 536 colorectal cancer samples with somatic mutation data. Among these, 79 samples experienced mutations with a frequency of 14.74% (**Figure 1A**). It was found that the mutation frequency of each regulator is relatively low, of which ATP7A showed the highest mutation rate, followed by ATP7B and LIPT1, while FDX1, CDKN2A, SLC31A1, and GCSH showed no mutation in all colorectal cancer tissues. The CNV analysis showed a copy number loss in most genes, while ATP7B, GLS, PDHA1, and LIPT1 presented a widespread frequency of CNV gain (**Figure 1B**). The location of CNV of all cuproptosis regulators on chromosomes was exhibited in **Figure 1C**. To further explore the influence of somatic mutation and CNV on the genomic expression of cuproptosis regulators, mRNA levels of these regulators were investigated in colorectal cancer tissues and normal tissues. The results indicated that the expression level of FDX1, LIAS, DLD, DLAT, PDHA1, MTF1, GLS, DBT, and DLST was lower in colorectal cancer tissues compared with normal tissues (**Figure 1D**), consistent with the CNV alteration, which implies that CNV may be the dominant effector on the expression alteration of cuproptosis regulators. In addition, we observed that PDHB and CDKN2A with a high frequency of CNV loss showed no significant differences in expression between cancer and normal tissues, and ATP7A with the highest frequency of mutation also showed no difference. Thus, CNV and somatic mutation cannot explain all the mRNA expression alterations, while epigenetic regulation, pre-mRNA splicing, and transcription factors also participate in the regulation of mRNA expression. The above results showed the high heterogeneity of genetic alteration of cuproptosis regulators in colorectal cancer patients.

### Identification of cuproptosis patterns in colorectal cancer

To explore the biological functions of these 16 cuproptosis regulators, we performed KEGG enrichment analysis. The function annotation results indicated that the cuproptosis regulators participated in cellular copper ion homeostasis, copper transport, protein lipoylation, TCA cycle, and response to oxidative stress (**Figure 1E**; **Supplementary Figure S1B**, **Supplementary Table S1**), consistent with the mechanism of cuproptosis. The metascape analysis also showed the same results (**Figure 1F**; **Supplementary Table S2**). Then, we analyzed the interaction correlations of the expression of each regulator (**Supplementary Figure S1C**). It was found that the expression of ATP7A was positively correlated with the expression of GLS, DBT, and MTF1, while the expression of CDKN2A was negatively correlated with the expression of DBT, DLD, DLAT, and PDHB. Furthermore, a univariate Cox regression model was constructed to analyze the prognostic roles of 16 cuproptosis regulators in colorectal cancer patients (**Supplementary Figure S2A**, **Supplementary Table S3**). The Kaplan–Meier curves indicated that patients with high expression of ATP7A, DLAT, DLD, FDX1, LIAS, PDHA1, and PDHB showed a longer overall survival compared with patients with low expression of these genes, respectively. Nonetheless, patients with high expression of CDKN2A and GLS showed an opposite overall survival (**Supplementary Figures S2B–J**). The cuproptosis regulators network describes the overall landscape of the interactions between cuproptosis regulators and the prognostic significance of these regulators for colorectal cancer patients (**Figure 1G**), indicating that the expression of cuproptosis regulators may serve a critical role in the progression and prognosis of colorectal cancer.

**Figure 2:**
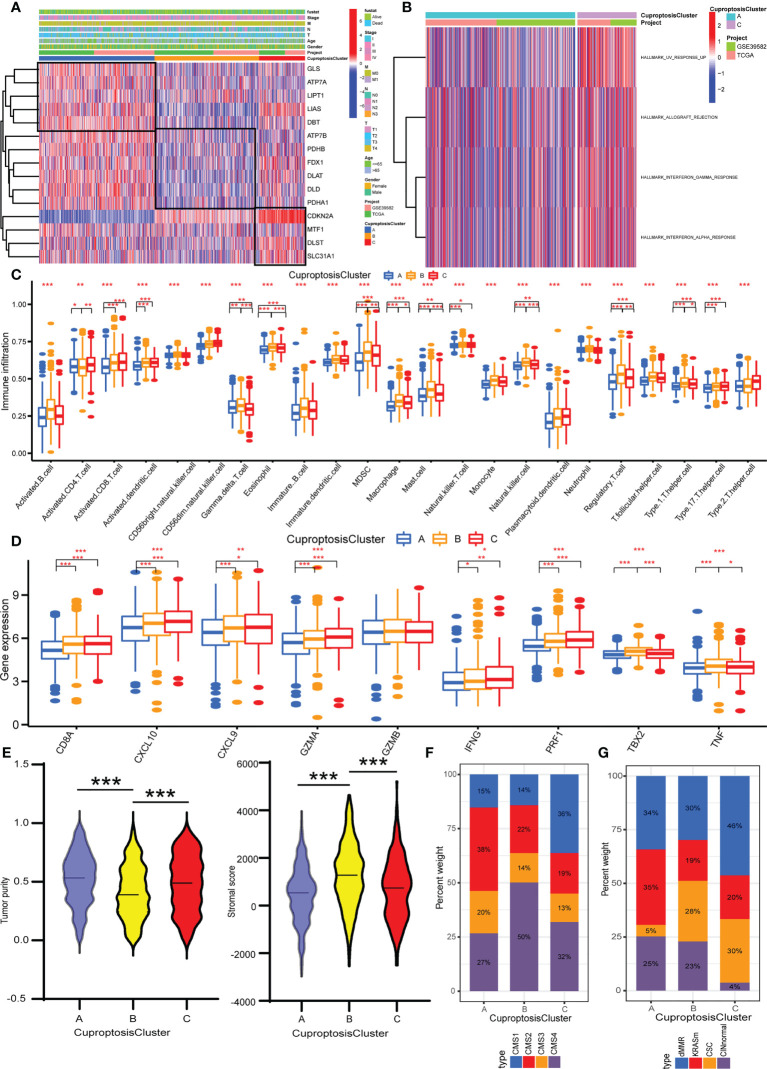
Identification of cuproptosis patterns and their TME characteristics in colorectal cancer. **(A)** Unsupervised consensus clustering of 16 cuproptosis regulators in the TCGA and GEO data. The CuproptosisCluster, gender, age, T stage, N stage, M stage, TNM stage, and survival status were used as annotations. **(B)** GSVA enrichment analysis showing the biological processes in CuproptosisClusters A and C. **(C)** The abundance analysis of each immune cell in the TME of three CuproptosisClusters. **(D)** The expression levels of immune activity related genes in three CuproptosisClusters. **(E)** The stromal score and tumor purity in three CuproptosisClusters with ESTIMATE algorithm. **(F)** The CMS analysis of the three CuproptosisClusters. **(G)** The molecular subtypes analysis of GSE39582 dataset in three CuproptosisClusters. *P <0.05; **P <0.01; ***P <0.001.

We merged the colorectal cancer samples of the TCGA data and GEO (GSE39582) data into one meta-cohort and performed PCA to explore whether the expression of 16 cuproptosis regulators could separate the cancer and normal samples. The scatter diagram showed that normal and cancer samples overlapped (**Supplementary Figure S3A**). The less normal samples may have led to this result. Then, based on the expression of 16 cuproptosis regulators, we performed an unsupervised consensus clustering algorithm to classify samples with distinct cuproptosis patterns. *K* from 2 to 9 was conducted and *K* = 3 showed the best results in terms of clustering (**Supplementary Figures S3B–D**). Three different cuproptosis patterns were finally confirmed. The three patterns were termed CuproptosisClusters A–C, of which 516 cases were in CuproptosisCluster A, 468 cases in CuproptosisCluster B, and 204 cases in CuproptosisCluster C (**Supplementary Table S4**). As shown in **Figure 2A**, CuproptosisCluster C has a high expression of CDKN2A, MTF1, DLST, and SLC31A1. CuproptosisCluster A shows a high expression of GLS, ATP7A, LIPT1, LIAS, and DBT, while CuproptosisCluster B exhibits a low expression of ATP7B, PDHB, FDX1, DLAT, DLD, and PDHA1. In addition, we observed that CuproptosisCluster B was preferentially related to the lower stage, and CuproptosisCluster C to the more dead status. The Kaplan–Meier curves showed no significant differences in overall survival between the three CuproptosisClusters, but a rising trend from CuproptosisCluster A to CuproptosisCluster B to CuproptosisCluster C (**Supplementary Figure S3E**).

### TME characteristics in distinct cuproptosis patterns

To appraise the biological functions in the three CuproptosisClusters, GSVA was performed with hallmark gene sets. CuproptosisCluster C was markedly enriched in immune and carcinogenic activation pathways, such as allograft rejection, interferon gamma response, and interferon alpha response (**Figure 2B**). CuproptosisCluster B was prominently related to stromal activation biological processes: epithelial-to-mesenchymal transition (EMT), myogenesis, and apical junction (**Supplementary Figure S4A**). However, there was no immune-related pathway enriched in CuproptosisCluster A. The ssGSEA results showed that CuproptosisCluster B was predominately enriched in immunosuppressive cells and innate immune cells including myeloid-derived suppressor cells (MDSCs), macrophage, monocyte, regulatory T cell, Th1 cell, Th17 cell, and dendritic cell, eosinophil, and mast cells compared with CuproptosisClusters A and C, while CuproptosisCluster C was enriched in immunocompetent cells such as activated CD4 T cell and CD8 T cell, natural killer cell, and Gamma delta T cell (**Figure 2C**). Amounts of research demonstrated that tumors with immune-excluded phenotype always exhibited with stromal activation and significant enrichment of immunosuppressive cells, which could not infiltrate in the tumor parenchyma but surround tumor cell focus. Therefore, we classified CuproptosisCluster B as an immune-excluded phenotype. Next, we examined the stromal activity related pathways, of which genes in EMT and transforming growth factor beta (TGFβ) pathways-ACTA2, CLDN3, COL4A1, SMAD9, TGFBR2, TWIST1, and VIM, were all highly expressed in CuproptosisCluster B (**Supplementary Figure S4B**), which confirmed the above conclusion. CuproptosisCluster C was remarkably rich in adaptive immune cells, including activated CD8 T cells, natural killer cells, and gamma delta T cells (**Figure 2C**). To be as it is, CuproptosisCluster C was termed as immune-inflamed phenotype. Similarly, compared with CuproptosisClusters A and B, immune activity-related genes such as CD8A, CXCL10, CXCL9, GZMA, GZMB, IFNG, PRF1, TBX2A, and TNF were more enriched in CuproptosisCluster C (**Figure 2D**). Then, we adapted the ESTIMATE algorithm to evaluate the stromal score and tumor purity (**Supplementary Table S5**). To our surprise, the results showed that CuproptosisCluster B was presented with less tumor purity and a higher stromal score compared with CuproptosisClusters A and C (**Figure 2E**), implying that samples in CuproptosisClusters A and C possessed more tumor parenchyma and CuproptosisCluster B more stromal cell components. In addition, we observed that CuproptosisCluster A presented with high tumor purity, combined with less enrichment of all the immune cells and lower expression levels of immune activity-related genes, indicating the immune-desert phenotype.

To further verify the TME characteristics of the three CuproptosisClusters, a consensus molecular subtype (CMS) of colorectal cancer was computed with the CMScaller package (**Supplementary Table S6**). At present, the recognized classification of CMS by the academic community is five types as follows: CMS1 is classified as an immune-inflamed phenotype, which is characterized by mismatch repair gene mutation and microsatellite instability. CMS4 is a stromal cell type with abnormal activation of the TGFβ signaling pathway, consistent with an immune-exclude phenotype. Relatively, CMS2 and CMS3 are classified as immune-desert phenotypes. The remaining samples, called mixed phenotypes, cannot be grouped into any single type. The results indicated that CuproptosisCluster A was predominated in CMS2, while CuproptosisCluster B was CMS4 and CuproptosisCluster C was CMS1 (**Figure 2F**). In addition, the samples of the GSE39582 dataset are divided into six molecular subtypes (**Supplementary Table S7**) (46). Samples with dMMR are preferably related to immune activation. CINnormal and CSC are closely correlated with EMT, stem feature, and stromal activation. KRASm is prone to being an immune-desert phenotype. Our results indicated that dMMR accounted for the most part of the CuproptosisCluster C, while CINnormal and CSC were the main part of CuproptosisCluster B, and KRASm is the major part of CuproptosisCluster A (**Figure 2G**), consistent with our above results. In addition, almost all the cuproptosis regulators showed a significant difference between the three CuproptosisClusters (**Supplementary Figure S4C**). Taken together, we identified the TME infiltration characteristics in three distinct CuproptosisClusters.

### Generation of cuproptosis gene signatures

**Figure 3:**
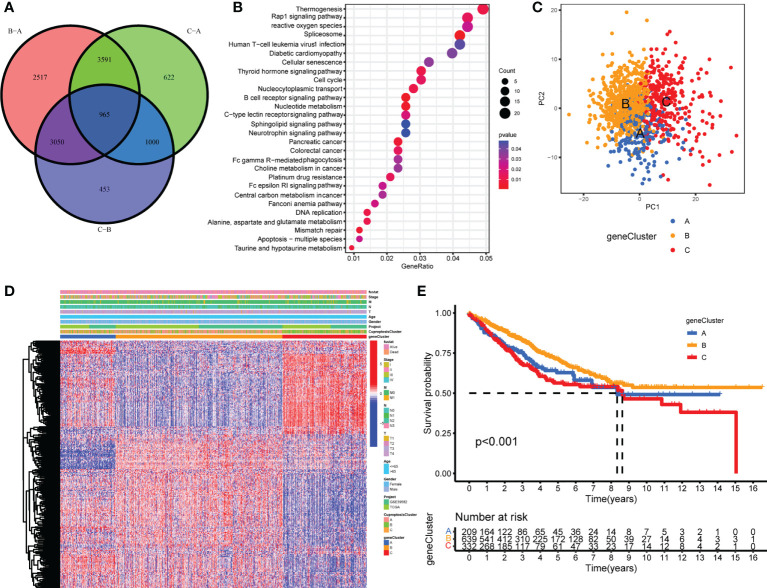
Generation of cuproptosis-related geneclusters. **(A)** The Venn diagram of DEGs between three CuproptosisClusters. **(B)** KEGG results of 965 DEGs. **(C)** PCA for three geneClusters to distinguish samples in TCGA COAD/READ and GSE39582. **(D)** Unsupervised clustering of 260 cuproptosis-related genes. **(E)** The Kaplan–Meier curves of overall survival between different geneClusters.

To further explore the underlying biological functions of the three cuproptosis patterns, DEGs between the three CuproptosisClusters were compared in pairs three times (significant criteria, adjusted P-value <0.01). Then, the intersecting genes of the three DEGs were accessed (**Figure 3A**). GO and KEGG enrichment analyses were performed using the 965 intersected genes. GO results showed that these genes participated in the biological processes of electron carrier activity, mitochondrial inner membrane, adherens junction, T-cell activation, B-cell differentiation, apoptosis process, and lymphatic endothelial cell differentiation (**Supplementary Figure S5A**). KEGG results revealed the pathways of colorectal cancer, reactive oxygen species, apoptosis, mismatch repair, platinum drug resistance, B-cell receptor signaling pathway, and Fc gamma R-mediated phagocytosis (**Figure 3B**). To our surprise, these biological processes and pathways are extraordinarily correlated with cuproptosis and immunity, which further verified the vital roles of cuproptosis patterns on the immune variation of TME.

In order to investigate the vital roles forward, univariate Cox regression was constructed to analyze the prognostic roles of the 965 intersect genes in colorectal cancer patients (**Supplementary Table S8**). A total of 260 genes were identified with significant prognostic values by P <0.05. Then, consensus clustering was performed again based on the expression of the 260 cuproptosis-related genes in order to group samples into different gene signature subtypes. Eventually, three cuproptosis-related gene phenotypes were generated, termed geneClusters A–C (**Supplementary Figures S5B–D**, **Supplementary Table S9**). The PCA results indicated an obvious distinction between the three geneClusters (**Figure 3C**), demonstrating the authentic existence of the cuproptosis molecular patterns in colorectal cancer. The correlations between geneClusters with clinicalpathological features were shown in **Figure 3D**. We observed significantdifferences in overall survival between the three geneClusters, with geneCluster B presenting the longest overall survival (**Figure 3E**).

### TME characteristics in the three cuproptosis gene feature patterns

**Figure 4:**
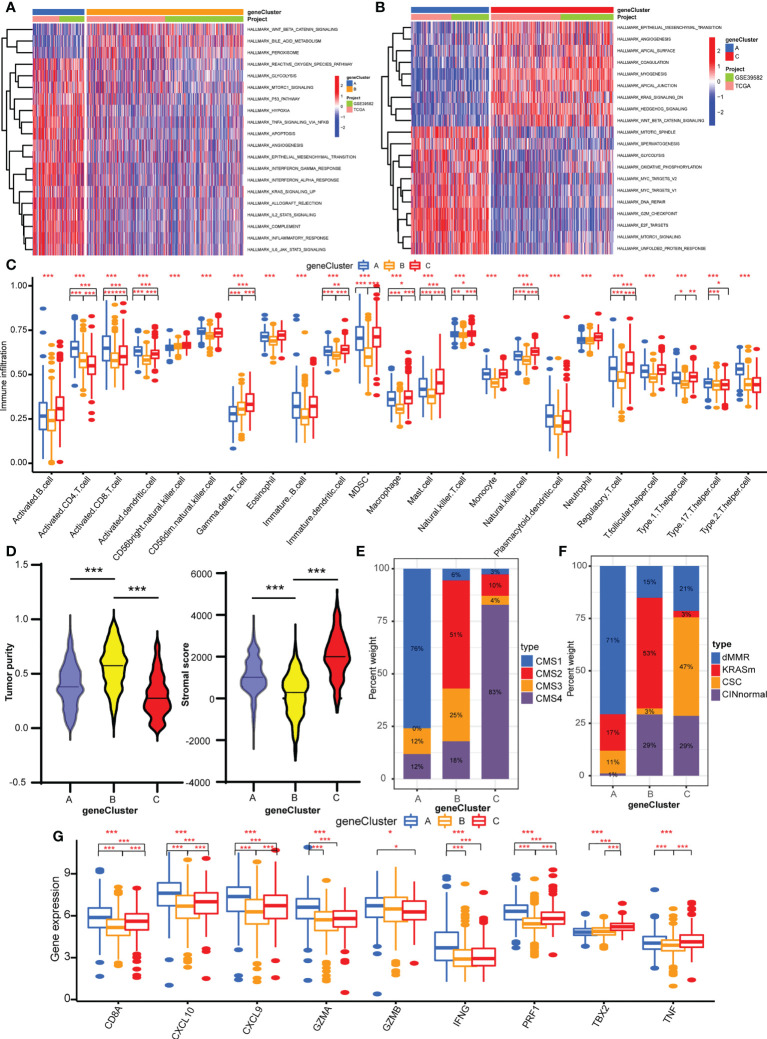
TME characteristics of three geneClusters. **(A)** The heatmap of GSVA results between geneCluster A and geneCluster B. **(B)** The heatmap of GSVA results between geneCluster A and geneCluster C. **(C)** The abundances of TME infiltrating immune cells in three geneClusters by ssGSVA algorithm. **(D)** The stromal score and tumor purity in three geneClusters by ESTIMATE algorithm. **(E)** The CMS analysis of the three geneClusters. **(F)** The molecular subtypes analysis of GSE39582 dataset in three geneClusters. **(G)** The expression of immune activated genes between three geneClusters. *P <0.05; **P <0.01; ***P <0.001.

GSVA was performed with hallmark gene sets to evaluate the biological functions of the three geneClusters. In comparison with geneClusters B and C, geneCluster A was markedly enriched in immune and carcinogenic activation pathways, such as hypoxia, p53 pathway, glycolysis, TNFA signaling *via* NFKB, allograft rejection, inflammatory response, interferon gamma response, interferon alpha response, IL2/STAT5 pathway, IL6/JAK/STAT3 pathway, and DNA repair (**Figure 4A**). GeneCluster C was prominently related to stromal activation biological process: EMT, angiogenesis, and Wnt/β-catenin signaling (**Figure 4B**). Then, the ssGSEA was performed to quantify the enrichment level of 22 immune cells in the three geneClusters. As shown in **Figure 4C**, geneCluster A was predominately enriched in adaptive immune cells including activated CD4 T cells and CD8 T cells, activated dendritic cells, and natural killer cells, and geneCluster C was remarkably rich in immunosuppressive cells and innate immune cells including MDSC, immature dendritic cells, macrophages, monocytes, regulatory T cells, Th1 cells, T follicular helper cells, and dendritic cells, eosinophils, and mast cells, while geneCluster B presented a lower enrichment level of immune cells. Furthermore, the stromal score and tumor purity of all samples were analyzed. GeneCluster B showed the highest tumor purity compared with geneCluster A and geneCluster C (**Figure 4D**), indicating the lowest infiltration of immune cells and stromal components. Therefore, geneCluster B was in accord with the immune-desert phenotype. Similarly, geneCluster C presented with the highest stromal score compared with geneCluster A and geneCluster B (**Figure 4D**). To further verify our speculation, two other known molecular subtypes were analyzed in the three gene clusters. GeneCluster A predominated in CMS1, while geneCluster C was CMS4, and geneCluster B was CMS2 and CMS3 (**Figure 4E**). The subtype of dMMR accounted for the majority of geneCluster A, while CINnormal and CSC were the main parts of geneCluster C, and KRASm is the major part of geneCluster B (**Figure 4F**). In addition, the gene levels of EMT and TGFβ pathways were higher in geneCluster C compared with geneClusters A and B (**Supplementary Figure S5E**), whereas immune activity related-genes were higher in geneCluster A compared with geneClusters C and B (**Figure 4G**). Combined with the features of immune cell infiltration, geneCluster C was consistent with the immune-excluded phenotype, while geneCluster A was the immune-inflamed phenotype. We also observed that all the cuproptosis regulators presented differentiated expression levels in the three geneClusters (**Supplementary Figure S5F**).

### Construction and characteristics of cuproptosisScore

**Figure 5:**
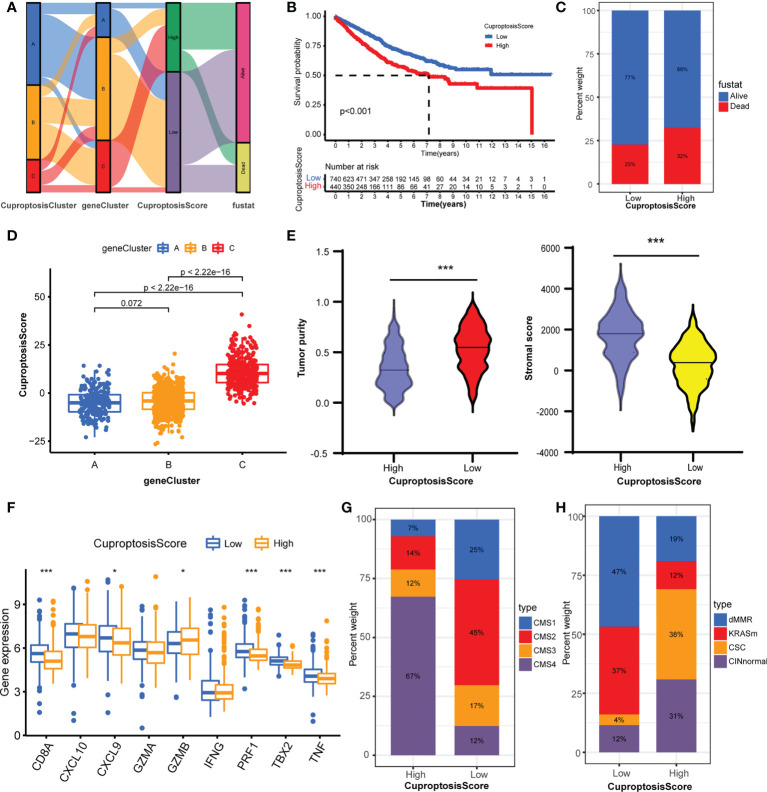
Construction of CuproptosisScore and their TME characteristics in colorectal cancer. **(A)** Alluvial diagram showing the attribute changes from CuproptosisClusters to gene Clusters to CuproptosisScore to survival status. **(B)** The Kaplan–Meier curves of overall survival between high and low CuproptosisScore group. **(C)** The frequencies of alive and dead status in high and low CuproptosisScore group. **(D)** The CuproptosisScores of the three geneClusters. **(E)** The stromal score and tumor purity in high and low CuproptosisScore group. **(F)** The expression levels of immune activity-related genes in high and low CuproptosisScore groups. **(G)** The CMS analysis of the two CuproptosisScore groups. **(H)** The molecular subtypes analysis of GSE39582 dataset in the two CuproptosisScore groups. *P <0.05; ***P <0.001.

To assess the cuproptosis patterns of individual patients, we constructed a cuproptosis score system with these cuproptosis-related genes, termed CuproptosisScore (**Supplementary Table S10** **)**. The alluvial diagram showed the attribute changes of individual samples (**Figure 5A**). The Kaplan–Meier curves indicated that patients with a high CuproptosisScore had shorter overall survival compared with patients with a low CuproptosisScore (**Figure 5B**). Also, more patients in the high CuproptosisScore group are in the status of death (**Figure 5C**). This Kruskal–Wallis test indicated significant differences in CuproptosisScore between cuproptosis-related gene clusters (**Figure 5D**). Genecluster C showed the highest median score, which indicated that a high CuproptosisScore may be correlated with stromal activation-related signatures. whereas geneCluster A had the lowest median score, showing that low CuproptosisScore may be related to an immune activation phenotype. Next, the analysis of stromal score and tumor purity found that samples with a high CuproptosisScore exhibited a high stromal score and low tumor purity (**Figure 5E**), implicating a stromal activation phenotype. While low CuproptosisScore presented low stromal score and high tumor purity, it also showed high expression of immune activated genes (**Figure 5F**), implying an immune activation phenotype. These results give us some hints that CuproptosisScore may contribute to predicting the immune cell filtrating in the cancer samples and further evaluating the immune response to the targeted drugs. The analysis of CMS subtypes showed that CMS4 comprises most of the high CuproptosisScore group, while the low CuproptosisScore group consisted of more CMS1 and less CMS4 (**Figure 5G**). In the GSE39582 dataset, the low CuproptosisScore group mainly belongs to the dMMR type, and the high CuproptosisScore group is mostly divided into the CINnormal and CSC types (**Figure 5H**). The above results strongly demonstrated that a high CuproptosisScore was remarkably related to an immune-exclude phenotype, while a low CuproptosisScore was correlated with an immune-inflamed phenotype. Therefore, the distinct cuproptosis-related genes play indispensable regulatory roles in shaping different TME characteristics. The CuproptosisScore could better assess the cuproptosis-related molecular patterns of individual samples and TME infiltration characteristics, which is conducive to selecting appropriate targeted drugs in the clinical treatment of colorectal cancer.

### Relationship of cuproptosis patterns with tumor somatic mutation and immunotherapy

**Figure 6:**
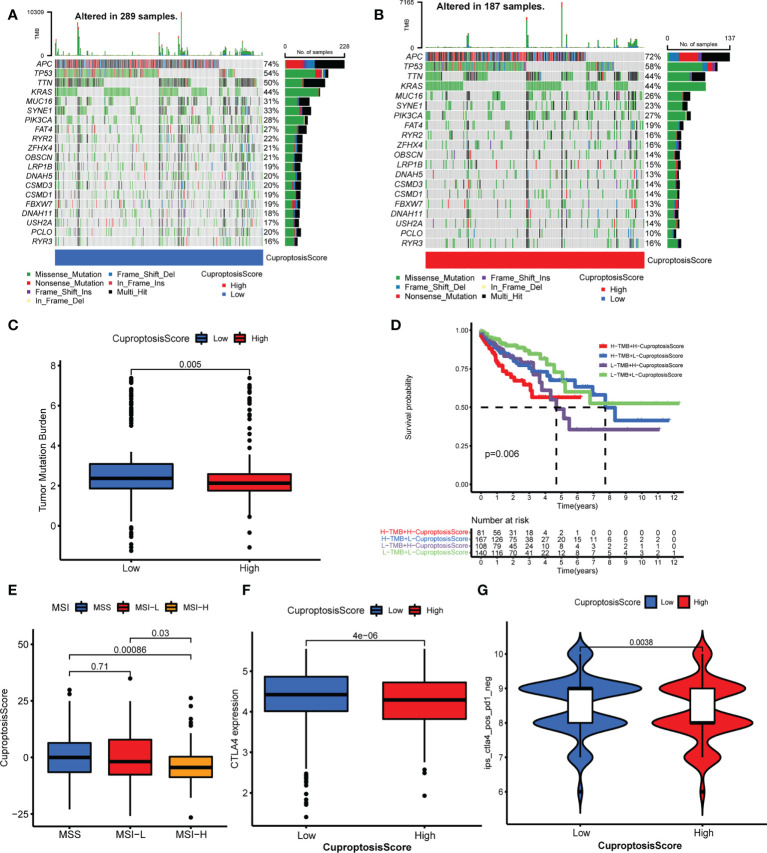
Relationship of cuproptosis patterns with tumor somatic mutation and immunotherapy. **(A, B)** The waterfall plot of tumor somatic mutation of low CuproptosisScore group **(A)** and high CuproptosisScore group **(B)**. **(C)** The TMB of high and low CuproptosisScore group. **(D)** The Kaplan–Meier curves of overall survival in different groups of TMB combination with CuproptosisScore. **(E)** The CuproptosisScores of samples with MSS, MSI-L, and MSI-H. **(F)** CTLA4 expression of the two CuproptosisScore groups. **(G)** IPS of anti-CTLA4 drug in the two CuproptosisScore groups.

In this part, we analyzed the differences in tumor somatic mutation, MSI, and immunotherapy response between different CuproptosisScore groups. First, we depicted the landscape of gene mutation in the high and low CuproptosisScore groups (**Figures 6A, B**), and then computed the TMB. The TMB of the low CuproptosisScore group was higher than that of the high CuproptosisScore group (**Figure 6C**), and CuproptosisScore was negatively correlated with TMB (**Supplementary Figure S6A**). Second, the Kaplan–Meier curves showed no significant difference in overall survival between patients with high and low TMB (**Supplementary Figure S6B**), but a trend of shorter 5-year survival in the low TMB group. However, when the combination of TMB and CuproptosisScore served as a prognostic indicator, the differences in overall survival between different groups were obvious (**Figure 6D**). The patients with low CuproptosisScore had longer overall survival compared with patients with high CuproptosisScore, both in the high and low TMB groups. Subsequently, we evaluated the correlation between CuproptosisScore and MSI. The results showed that MSI-H group scored low CuproptosisScore, while MSS and MSI-L group scored high CuproptosisScore (**Figure 6E**). Considering that CuproptosisScore was closely associated with TME infiltration features, we evaluated the influence of CuproptosisScore on immunotherapy. High CTLA4 expression was observed in the low CuproptosisScore group compared with the high CuproptosisScore group (**Figure 6F**). The IPS of the TCGA samples was downloaded online (https://tcia.at/home, **Supplementary Table S11**). When treated with an anti-CTLA4 drug, the low CuproptosisScore group presented a high IPS, meaning better immunotherapy response (**Figure 6G**). When treated with an anti-PD1 drug, no significant difference in IPS was found between the two CuproptosisScore groups (**Supplementary Figure S6C**). In addition, CuproptosisScore can serve as a prognostic marker in different stages of colorectal cancer patients. Longer overall survival was observed in low CuproptosisScore group compared with high CuproptosisScore group, in patients with T3–T4 stage, N1–N3 stage, M0 stage and TNM III–IV stage (**Supplementary Figures S7A–H**).

## Discussion

Cuproptosis is a novel cell death format regulated by an ancient mechanism, distinct from all other known mechanisms (15). Both copper chelators and copper ionophores have been exploited as antitumor drugs and tested in clinical trials (9, 52, 53). However, the correlations between cuproptosis regulators with molecular patterns, clinicalpathological subtypes, prognostic values, TME infiltration features, and immunotherapy response have not been investigated. Therefore, identifying the roles of cuproptosis-related molecular patterns will promote our cognition of cuproptosis and its features in colorectal cancer, which contributes to exploring the potential of cuproptosis-related gene signatures to serve as a marker for evaluating prognosis and antitumor immune response.

In this study, we accessed 16 cuproptosis regulators and analyzed their mutation, CNV, expression level, and biological functions in colorectal cancer. CNV was the main interfering factor in the expression level of 16 cuproptosis regulators. These cuproptosis regulators participate in copper ion homeostasis, protein lipoylation, TCA cycle, and response to oxidative stress. Clinically, most of these cuproptosis regulators are the prognostic effectors of colorectal cancer patients. Then, we constructed three distinct cuproptosis clusters based on the expression of these cuproptosis regulators. CuproptosisCluster B was preferentially related to lower stage and CuproptosisCluster C more dead status, consistent with the trend of longer overall survival in CuproptosisCluster B than CuproptosisCluster C. Through multiple evidences of different research methods, we demonstrated that the three CuproptosisClusters were characterized by distinct TME characteristics. CuproptosisCluster B was characterized by stromal activation and immunosuppressive phenotype, corresponding to immune-exclude subtype; CuproptosisCluster C was enriched in immune and carcinogenic activation pathway, related to immune-inflamed subtype; while no immune-related pathway and little immune cells were enriched in CuproptosisCluster A, grouped into immune-desert subtype. ESTIMATE algorithm showed high tumor purity and little stromal component in CuproptosisCluster C, whereas CuproptosisCluster B had more stromal component. Surprisingly, the ssGSEA research found unique high level of adaptive immune cells including activated CD8 T cell, natural killer cell, and gamma delta T cell in CuproptosisCluster C, while marked enrichment of immunosuppressive cells and innate immune cells including macrophage, monocyte, regulatory T cell, Th1 cell, Th17 cell, and dendritic cell, eosinophil, and mast cell in CuproptosisCluster B. Activated CD8 T cell by neoantigen is the predominate effector cell exerting tumor killing and at the hotspot of antitumor immunotherapy (54). Nonetheless, the immune system consists of a complicated process of antitumor response in which multiple immune cells, cytokines, and chemical factors participate. These mechanisms must coordinate with each other and play a mutually reinforcing role. Natural killer cells are also a key antitumor effector cell working through perforin, granular enzyme or antibody dependent cell-mediated cytotoxicity (ADCC) (55). γδ T cells can be elicited by butyrophilin or butyrophilin-like molecules in a major histocompatibility complex (MHC)-independent manner and bridge innate and adaptive immunity, therefore responding to multiple types of cancers (56, 57). In addition, regulatory T cells act as immunosuppressive cells through many cytokines and cells. However, macrophages are known to be classified into two main phenotypes: proinflammatory and antitumor M1 macrophages and immunosuppressive M2 macrophages (58, 59). M0 macrophages can be selectively polarized into M1 macrophages or M2 macrophages according to the TME characteristics. M1 macrophages produce type I pro-inflammatory cytokines to promote immune response, while M2 macrophages enhance matrix remodeling, EMT, and Wnt/β-catenin pathway (60, 61). Therefore, a broad range of immune cells were activated and played nonnegligible roles in the antitumor immune response, which provided great plasticity for TME characteristics. Although CuproptosisCluster B was rich in immune cells, these cells played immunosuppressive roles or surrounded the tumor focus but penetrated into the tumor parenchyma, rendering them unable to exert antitumor effects (62, 63). Combined with the above results, CuproptosisCluster B was classified as an immune-excluded phenotype. Furthermore, two molecular subtypes of colorectal cancer (the recognized classification of CMS and molecular subtypes of the GSE39582 dataset) were adapted to verify the above speculation. The results indicated that CMS1 or dMMR accounted for the most part of the CuproptosisCluster C, while CMS4 or CINnormal and CSC were the main part of CuproptosisCluster B, and CMS2 and CMS3 or KRASm consisted of the major part of CuproptosisCluster A. The similar TME characteristics of different molecular subtypes proved the availability and effectiveness of CuproptosisClusters. Finally, we examined the level of immune activity related genes and stromal activity related pathways. The immune activity related genes were significantly highly expressed in CuproptosisCluster C, and the stromal activity related pathways were remarkably rich in CuproptosisCluster C. In general, after comprehensively exploring the TME characteristics of distinct cuproptosis clusters, the novel molecular subtype was proved to be a reliable and effective classification of colorectal cancer patients and immune phenotype.

The differentially expressed genes between the three cuproptosis clusters were proved to be correlated with cuproptosis and immune activation pathways, and considered as cuproptosis-related signature genes. Based on the expression of these genes, three gene clusters were identified to group samples with distinct clinical–pathological features and a TME phenotype. GeneCluster A was markedly enriched in immune and carcinogenic activation pathways, CMS1 subtype, dMMR subtype and high levels of immune activation genes with high tumor purity and low stromal component, classified as the immune-inflamed phenotype. GeneCluster C was remarkably rich in immunosuppressive cells, CMS4 subtype, CINnormal,and CSC subtypes, and high levels of stromal activation genes with high stromal component and low tumor purity, classified as an immune-excluded phenotype. While GeneCluster B was short of immune cells, it was classified as an immune-desert phenotype. This indicates that the cuproptosis-related genes play crucial roles in shaping different TME characteristics. Considering the crucial roles of cuproptosis patterns in the TME formation of colorectal cancer, we constructed a score system, termed CuproptosisScore, to quantify the cuproptosis patterns of individual samples. The low CuproptosisScore indicated the immune-inflamed phenotype with longer overall survival, while the high CuproptosisScore indicated the immune-excluded phenotype with shorter overall survival. Integrated analysis demonstrated that CuproptosisScore could serve as an effective prognostic marker and indicator of immune subtype (64). For clinical patients, the cuproptosis-related genes can be detected with transcriptome sequencing to calculate the CuproptosisScore for individuals, therefore evaluating their prognosis and immune subtype.

With the development of tumor behaviors and immunological molecular mechanisms, immunotherapy provides a novel site for tumor targeting therapy, especially immune checkpoint inhibitors (ICIs) (65–67), including CTLA4, PD-1, and PD-L1. MSI-H or dMMR are indicators of immunotherapy response for colorectal cancer. We found low CuproptosisScore presented with high TMB, MSI-H, and high expression of CTLA4, and better response to anti-CTLA4 immunotherapy. Previous research reported that TGFβ and EMT related pathways impaired the penetrating of T cells into tumor focus and weakened antitumor effects (21). This is consistent with our results that stromal activation phenotypes with activated TGFβ and EMT pathways exhibited the immune-exclude phenotype and were resistant to ICI response. Combined with tumor stage, TMB, CTLA4, and PD-L1 expression, MSI status, and TME phenotype, CuproptosisScore can serve as an effective predictive schedule for prognosis and contribute to performing patient stratification for the determination of immunotherapy regimen in colorectal cancer patients.

Although we first reveal the clinical pathology and TME phenotype of cuproptosis-related gene patterns in colorectal cancer, there are several shortcomings in the study. First, cuproptosis was established in recent years, so more cuproptosis-related genes remain to be discovered, which will provide more profound insights for cuproptosis. Second, there is no report about cuproptosis with tumor progression and therapy up to now. Therefore, more mechanism studies need to be performed to enhance our cognition of the correlation between cuproptosis and cancer. Third, despite that we provided a novel direction for cuproptosis and TME phenotype, no report about the mechanisms of cuproptosis on shaping TME infiltration has been retrieved. Fourth, the CuproptosisScore is short of verification from other data. More data needs to be collected to confirm the authenticity and reliability of CuproptosisScore. Finally, all the data were accessed from public dataset, and selection bias of samples may exist. Large-scale prospective studies need to be performed to verify the findings.

## Conclusion

In conclusion, this study revealed a novel cuproptosis-related gene pattern with different clinical–pathological and TME phenotypes. The integrated analysis of different cuproptosis patterns contributes to our understanding of TME and provides an effective marker for prognosis and immunotherapy of colorectal cancer.

## Data availability statement

The original contributions presented in the study are included in the article/**Supplementary Material**. Further inquiries can be directed to the corresponding author.

## Author contributions

YX and ZZ designed the idea of the article. ZZ and QZ carried out the experiments and analyzed the data. WS and JW wrote the manuscript. SL, TG, and CZ supervised and verified the data analysis. YX validated the manuscript. All authors contributed to the article and approved the submitted version.

## Funding

This research was supported by the Science and Technology Commission of Shanghai Municipality (20DZ1100101), the Shanghai Hospital Development Center (SKXZ2028), and the National Natural Science Foundation of China (82003060).

## Conflict of interest

The authors declare that the research was conducted in the absence of any commercial or financial relationships that could be construed as a potential conflict of interest.

## Publisher’s note

All claims expressed in this article are solely those of the authors and do not necessarily represent those of their affiliated organizations, or those of the publisher, the editors and the reviewers. Any product that may be evaluated in this article, or claim that may be made by its manufacturer, is not guaranteed or endorsed by the publisher.
