# Supplementary material for: A novel cuproptosis-related molecular pattern and its tumor microenvironment characterization in colorectal cancer
Source: Front Immunol. 2022 Sep 30;13:940774. doi: 10.3389/fimmu.2022.940774 (PMC9561547; doi:10.3389/fimmu.2022.940774)
Supplement: Supplementary file 1 [file DataSheet_1.docx]

**Supplementary files:**

**Figure S1**


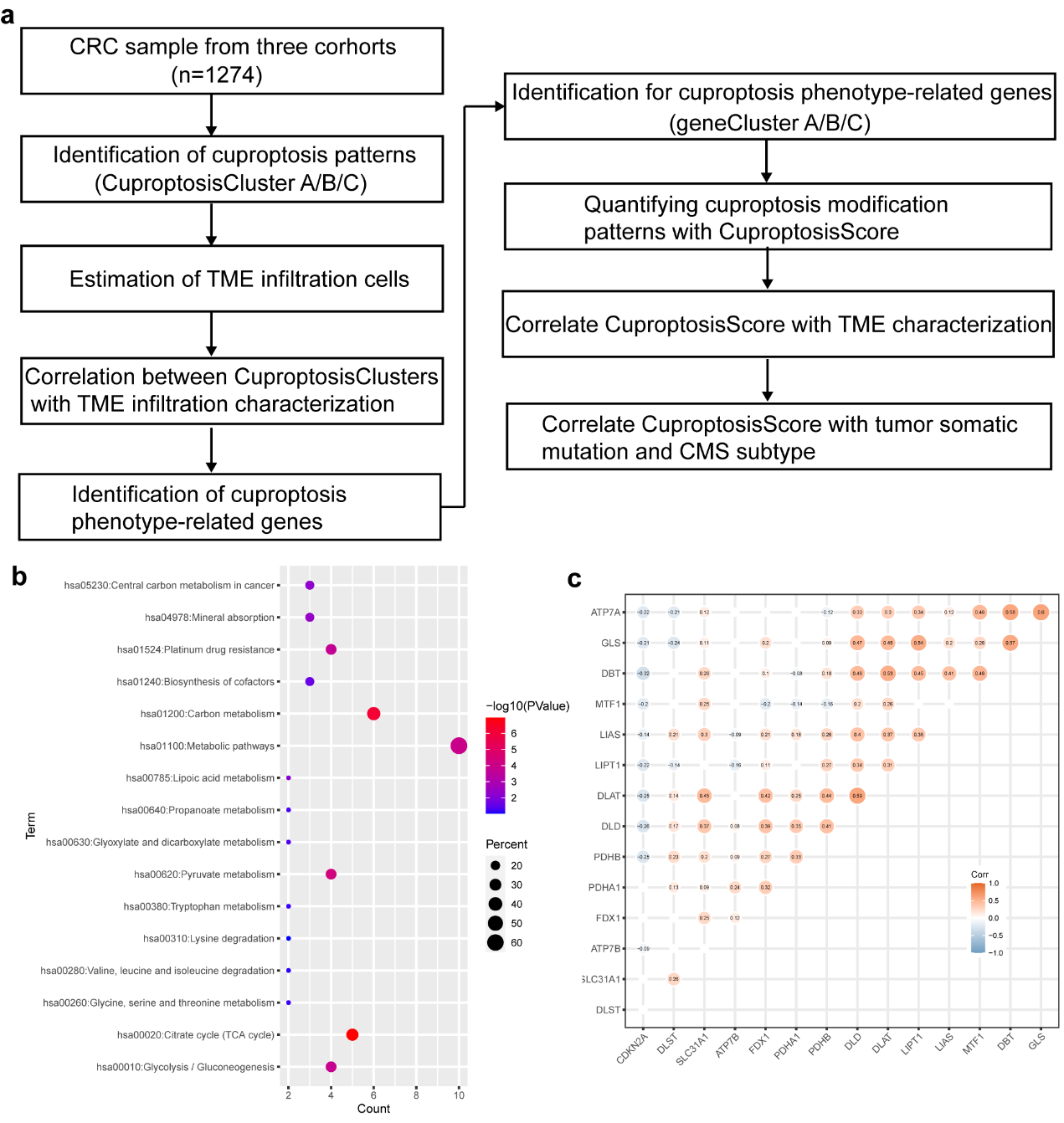


**Figure S2**


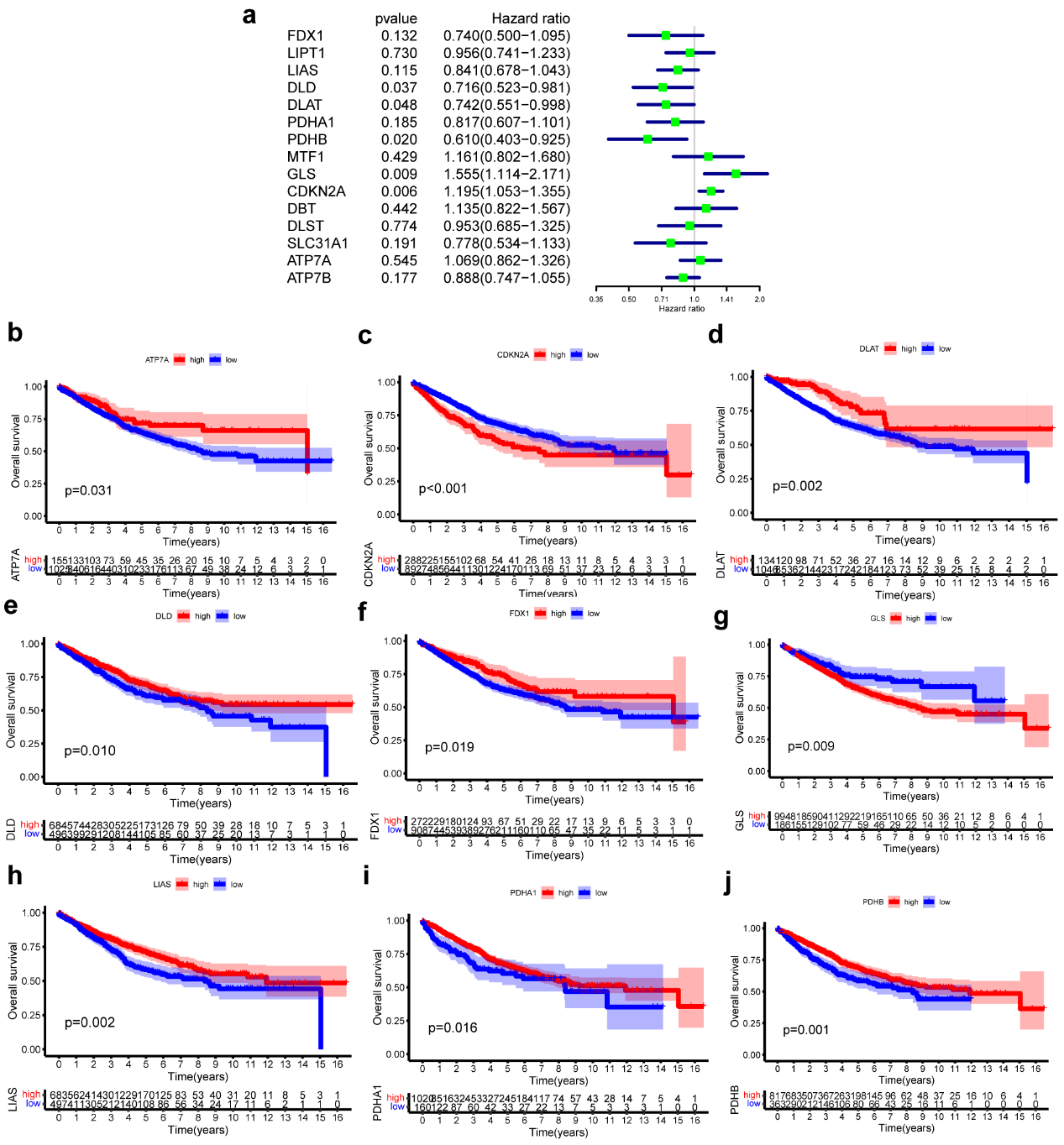


**Figure S3**

**
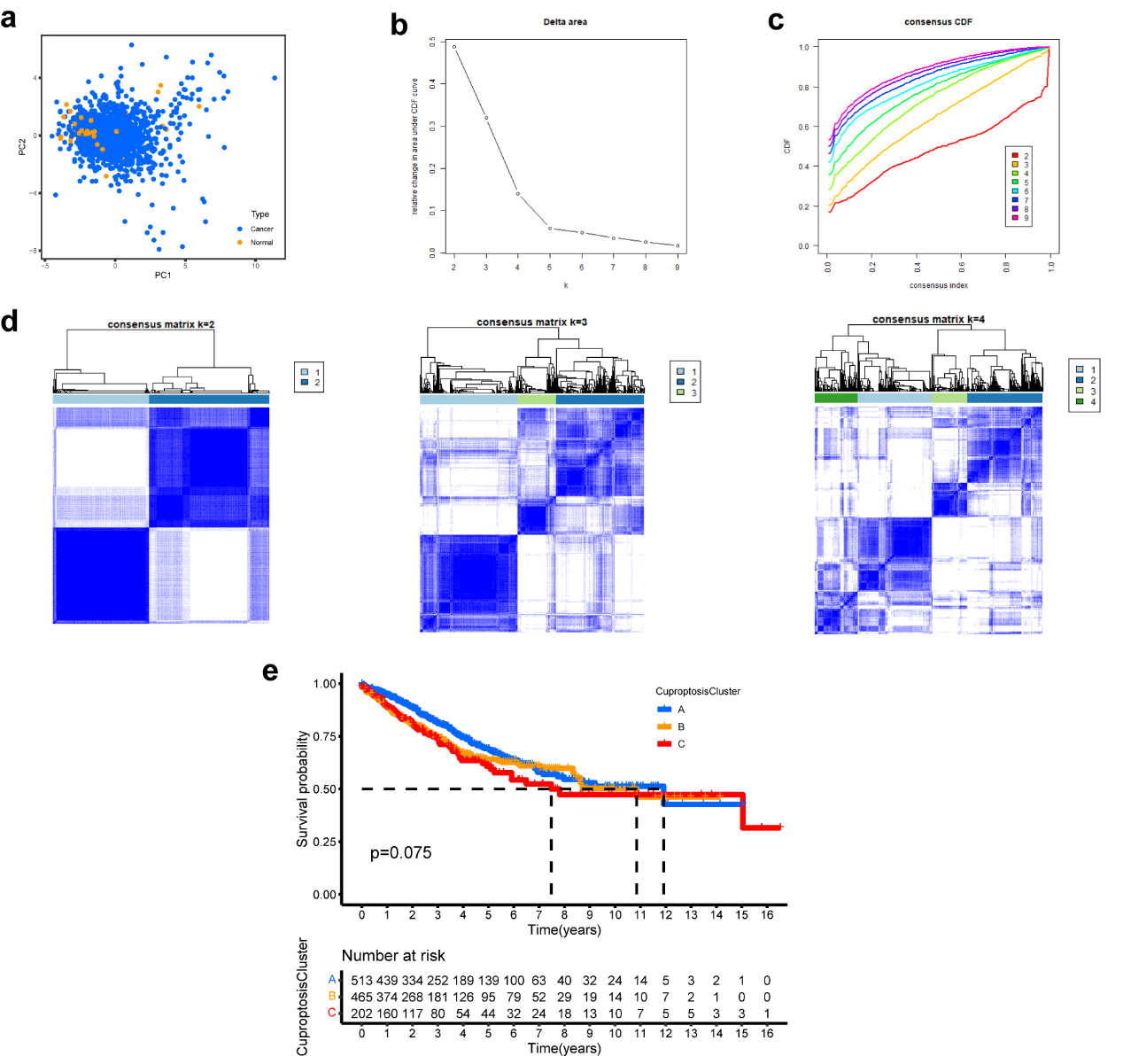
**

**Figure S4**

**
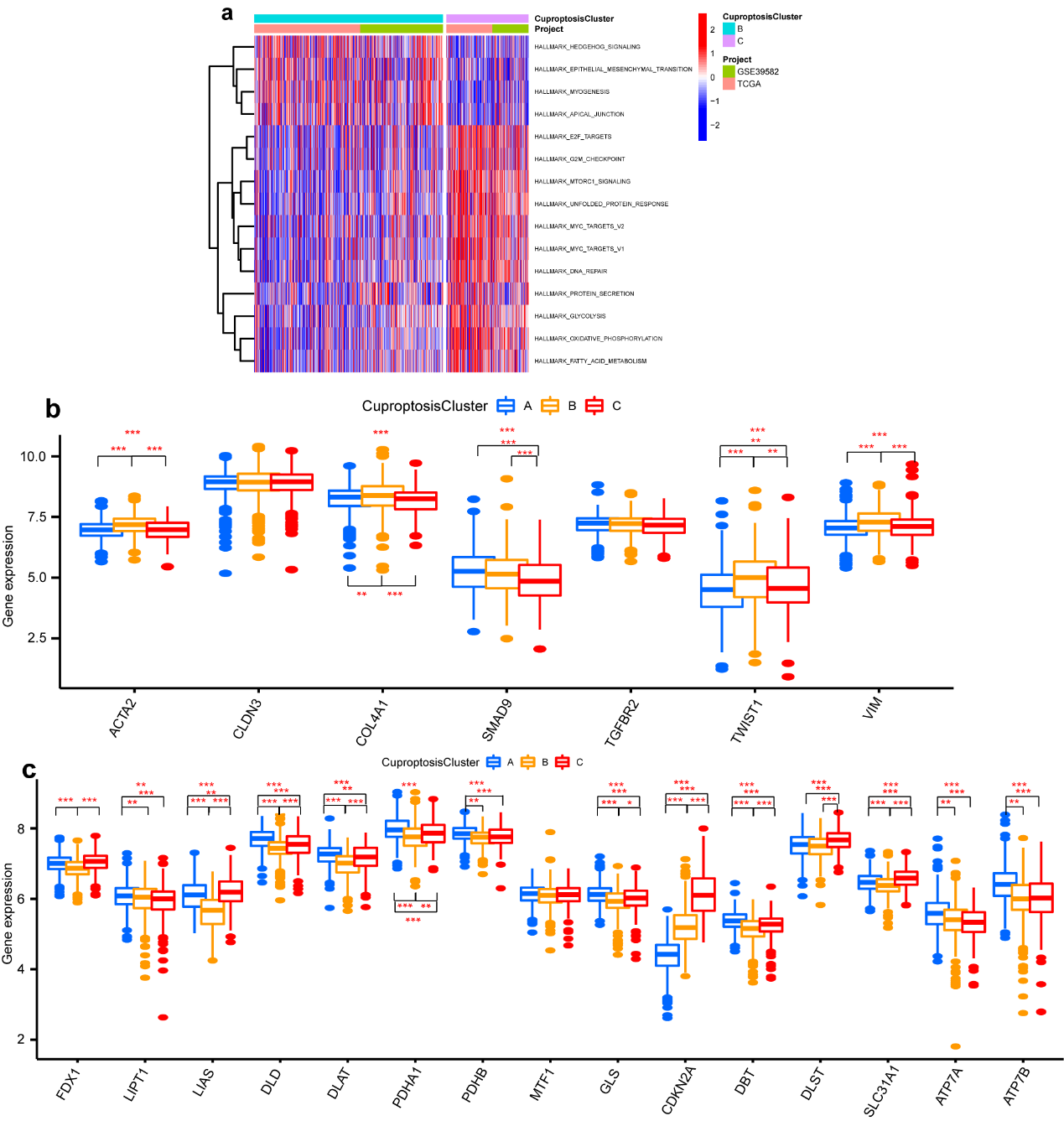
**

**Figure S5**


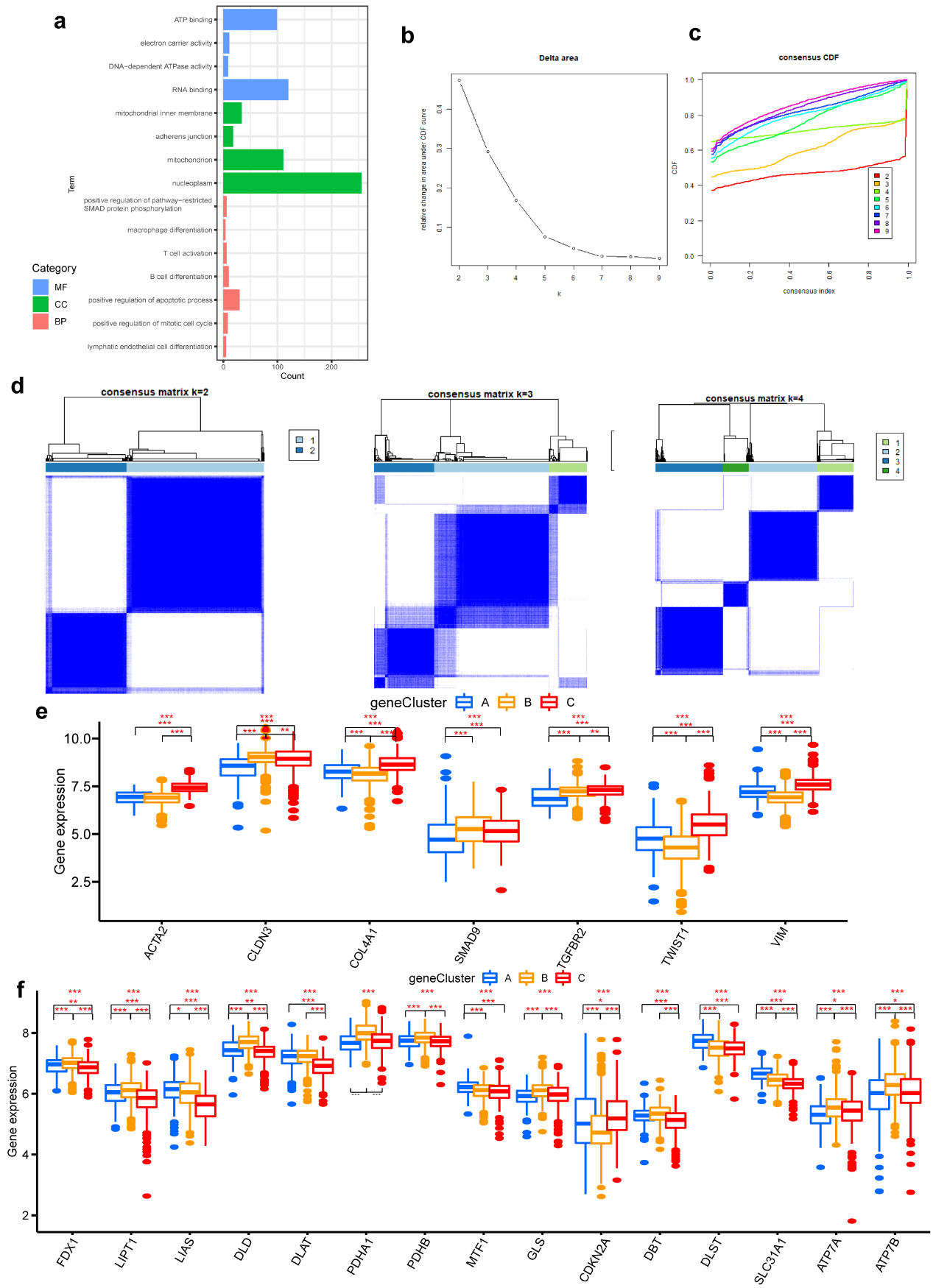


**Figure S6**

**
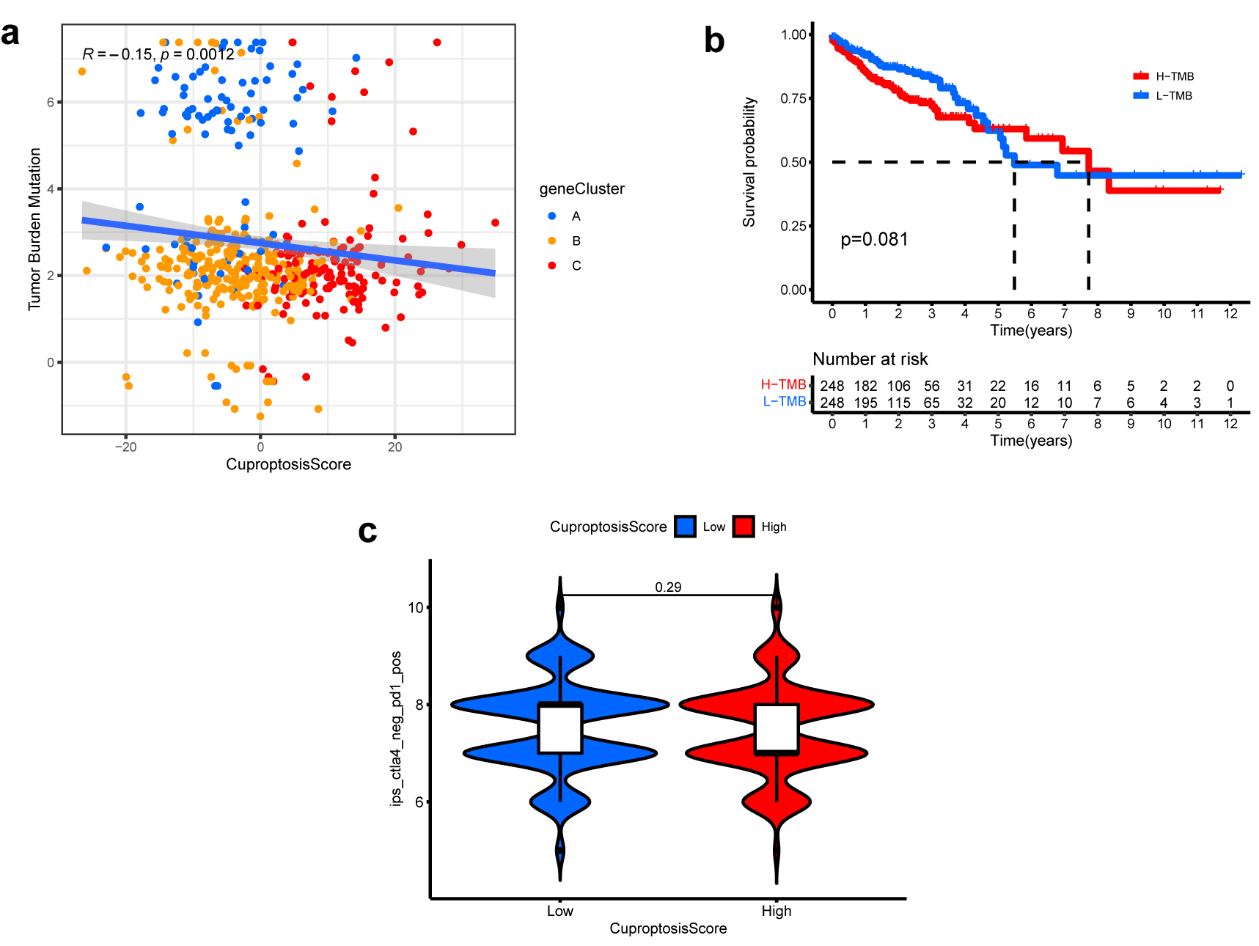
**

**Figure S7**


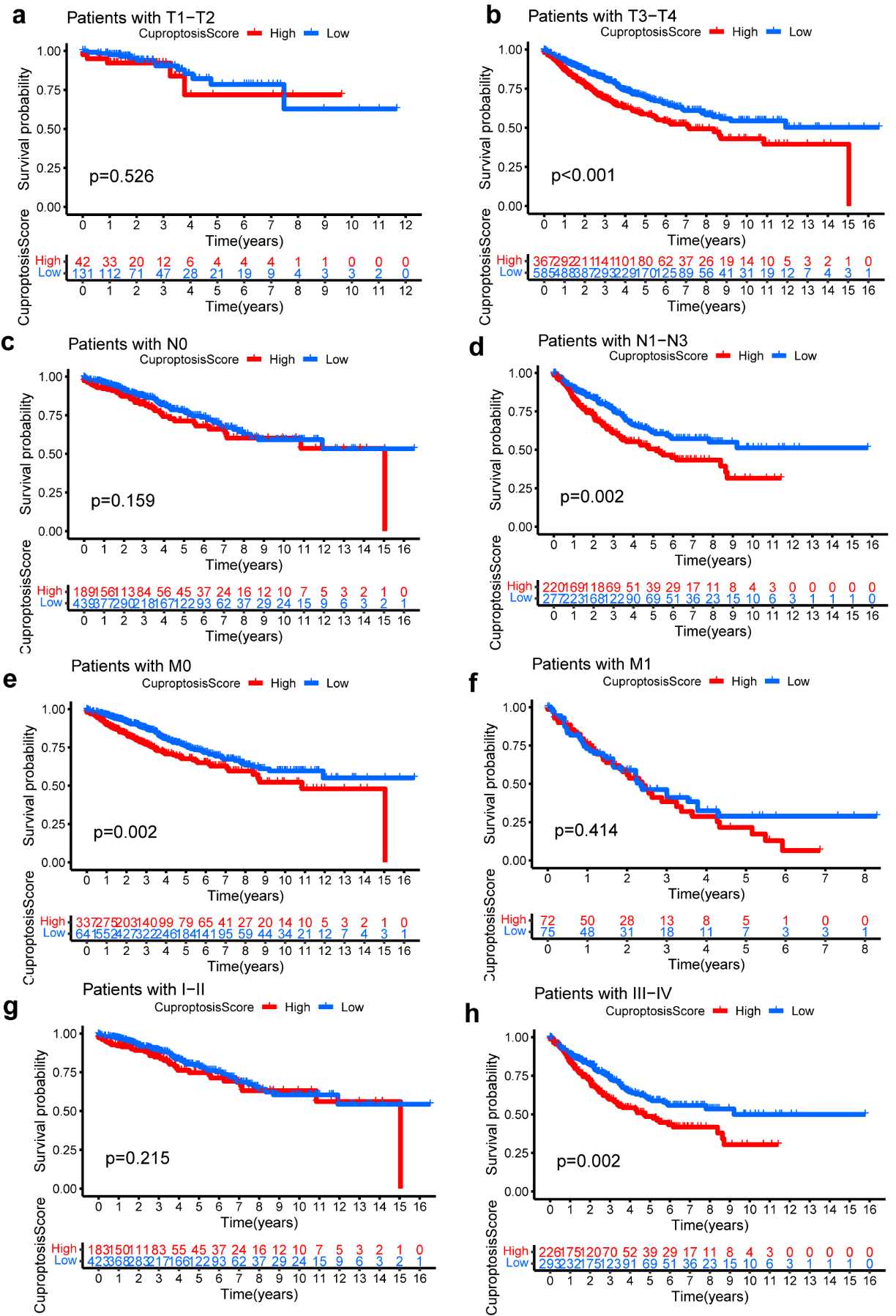


**Supplementary FIGURE S1** | The process of this work and function annotation of 16 cuproptosis regulators. **a** The process of this work. **b** KEGG analysis results of 16 cuproptosis regulators**. c** The correlation heatmap of 16 cuproptosis regulators.

**Supplementary FIGURE S2** | The prognostic significance of 16 cuproptosis regulators**. a** The univariate COX regression analysis of 16 cuproptosis regulators in colorectal cancer patients. **b-j** The Kaplan-Meier curves of ATP7A, DLAT, DLD, FDX1, LIAS, PDHA1 and PDHB in colorectal cancer patients with significant differences.

**Supplementary FIGURE S3** | Unsupervised consensus clustering of cuproptosis regulators and their characteristics. **a** PCA with the expression of 16 cuproptosis regulators in TCGA and GEO data. **b** The relative change in area under the cumulative distribution function (CDF) curves. **c** The curves of consensus CDF. **d** The heat maps for consensus matrices (k=2-4). **e** The Kaplan-Meier curves of overall survival between different CuproptosisCluster groups.

**Supplementary FIGURE S4** | TME characteristics of three geneClusters. **a** The heatmap of GSVA results between CuproptosisClusters B and CuproptosisCluster C with hallmark gene sets. **b** The expression of EMT and TGFβ pathways-related genes between three CuproptosisClusters. **c** The expression of 16 Cuproptosis regulators between three CuproptosisClusters. *P < 0.05; **P < 0.01; ***P < 0.001.

**Supplementary FIGURE S5** | Construction of cuproptosis-related geneClusters. **a** GO results of 965 DEGs. **b** The relative change in area under the cumulative distribution function (CDF) curves for geneCluster construction. **c** The curves of consensus CDF for geneCluster construction. **d** The heat maps for consensus matrices (k=2-4). **e** The expression of EMT and TGFβ pathways-related genes between three geneClusters. **f** The expression of 16 Cuproptosis regulators between three geneClusters. *P < 0.05; **P < 0.01; ***P < 0.001.

**Supplementary FIGURE S6** | Relationship of CuproptosisScores with tumor somatic mutation and immunotherapy. **a** Spearman correlation analysis of the CuproptosisScore and TMB in TCGA sample. **b** The Kaplan-Meier curves of TCGA samples with different TMB. **c** IPS of anti-PD-1 drug in the two CuproptosisScore groups.

**Supplementary FIGURE S7** | The Kaplan-Meier curves of high and low CuproptosisScore groups in different clinicalpathological stages. The Kaplan-Meier curves of high and low CuproptosisScore groups in TCGA and GEO samples with T1-T2 stages (**a**), T3-T4 stages (**b**), N0 stages (**c**), N1-N3 stages (**d**), M0 stages (**e**), M1 stages (**f**), TNM I-II stages (**g**) and TNM III-IV stages (**h**), respectively.

**Supplementary Table S1** | The results of GO analysis of 16 cuproptosis regulators.

| Category | Term | Count | % | PValue |
| --- | --- | --- | --- | --- |
| GOTERM_CC_DIRECT | GO:0005759~mitochondrial matrix | 12 | 75 | 1.43E-16 |
| GOTERM_CC_DIRECT | GO:0005739~mitochondrion | 13 | 81.25 | 5.13E-12 |
| GOTERM_CC_DIRECT | GO:0045254~pyruvate dehydrogenase complex | 4 | 25 | 3.33E-09 |
| GOTERM_BP_DIRECT | GO:0006086~acetyl-CoA biosynthetic process from pyruvate | 4 | 25 | 7.53E-09 |
| GOTERM_MF_DIRECT | GO:0034604~pyruvate dehydrogenase (NAD+) activity | 4 | 25 | 8.25E-09 |
| GOTERM_BP_DIRECT | GO:0006099~tricarboxylic acid cycle | 5 | 31.25 | 1.52E-08 |
| GOTERM_CC_DIRECT | GO:0005967~mitochondrial pyruvate dehydrogenase complex | 4 | 25 | 1.86E-08 |
| KEGG_PATHWAY | hsa00020:Citrate cycle (TCA cycle) | 5 | 31.25 | 1.06E-07 |
| GOTERM_BP_DIRECT | GO:0006090~pyruvate metabolic process | 4 | 25 | 5.75E-07 |
| KEGG_PATHWAY | hsa01200:Carbon metabolism | 6 | 37.5 | 6.17E-07 |
| GOTERM_BP_DIRECT | GO:0061732~mitochondrial acetyl-CoA biosynthetic process from pyruvate | 3 | 18.75 | 5.60E-06 |
| GOTERM_MF_DIRECT | GO:0005375~copper ion transmembrane transporter activity | 3 | 18.75 | 5.95E-06 |
| GOTERM_BP_DIRECT | GO:0009249~protein lipoylation | 3 | 18.75 | 8.40E-06 |
| GOTERM_BP_DIRECT | GO:0015677~copper ion import | 3 | 18.75 | 8.40E-06 |
| GOTERM_BP_DIRECT | GO:0006825~copper ion transport | 3 | 18.75 | 4.35E-05 |
| KEGG_PATHWAY | hsa00620:Pyruvate metabolism | 4 | 25 | 5.01E-05 |
| GOTERM_BP_DIRECT | GO:0006878~cellular copper ion homeostasis | 3 | 18.75 | 6.69E-05 |
| KEGG_PATHWAY | hsa01100:Metabolic pathways | 10 | 62.5 | 1.03E-04 |
| KEGG_PATHWAY | hsa00010:Glycolysis / Gluconeogenesis | 4 | 25 | 1.45E-04 |
| KEGG_PATHWAY | hsa01524:Platinum drug resistance | 4 | 25 | 1.87E-04 |
| GOTERM_MF_DIRECT | GO:0016746~transferase activity, transferring acyl groups | 3 | 18.75 | 7.72E-04 |
| GOTERM_BP_DIRECT | GO:0006006~glucose metabolic process | 3 | 18.75 | 0.001066 |
| GOTERM_MF_DIRECT | GO:0043682~copper-transporting ATPase activity | 2 | 12.5 | 0.001598 |
| GOTERM_BP_DIRECT | GO:0060003~copper ion export | 2 | 12.5 | 0.002324 |
| GOTERM_MF_DIRECT | GO:0004739~pyruvate dehydrogenase (acetyl-transferring) activity | 2 | 12.5 | 0.002396 |
| GOTERM_CC_DIRECT | GO:0045252~oxoglutarate dehydrogenase complex | 2 | 12.5 | 0.002973 |
| GOTERM_CC_DIRECT | GO:0005947~mitochondrial alpha-ketoglutarate dehydrogenase complex | 2 | 12.5 | 0.003714 |
| GOTERM_BP_DIRECT | GO:0055070~copper ion homeostasis | 2 | 12.5 | 0.003871 |
| GOTERM_BP_DIRECT | GO:0006546~glycine catabolic process | 2 | 12.5 | 0.003871 |
| KEGG_PATHWAY | hsa04978:Mineral absorption | 3 | 18.75 | 0.003982 |
| GOTERM_CC_DIRECT | GO:0005770~late endosome | 3 | 18.75 | 0.004936 |
| KEGG_PATHWAY | hsa05230:Central carbon metabolism in cancer | 3 | 18.75 | 0.005384 |
| GOTERM_BP_DIRECT | GO:0051353~positive regulation of oxidoreductase activity | 2 | 12.5 | 0.006186 |
| KEGG_PATHWAY | hsa00785:Lipoic acid metabolism | 2 | 12.5 | 0.006396 |
| GOTERM_BP_DIRECT | GO:0006554~lysine catabolic process | 2 | 12.5 | 0.009266 |
| GOTERM_BP_DIRECT | GO:0006103~2-oxoglutarate metabolic process | 2 | 12.5 | 0.013869 |
| GOTERM_BP_DIRECT | GO:0009083~branched-chain amino acid catabolic process | 2 | 12.5 | 0.016163 |
| GOTERM_BP_DIRECT | GO:0019752~carboxylic acid metabolic process | 2 | 12.5 | 0.017689 |
| KEGG_PATHWAY | hsa01240:Biosynthesis of cofactors | 3 | 18.75 | 0.024055 |
| GOTERM_BP_DIRECT | GO:0007595~lactation | 2 | 12.5 | 0.036585 |
| GOTERM_MF_DIRECT | GO:0005507~copper ion binding | 2 | 12.5 | 0.046143 |
| KEGG_PATHWAY | hsa00630:Glyoxylate and dicarboxylate metabolism | 2 | 12.5 | 0.047059 |
| KEGG_PATHWAY | hsa00640:Propanoate metabolism | 2 | 12.5 | 0.050122 |
| KEGG_PATHWAY | hsa00260:Glycine, serine and threonine metabolism | 2 | 12.5 | 0.062285 |
| KEGG_PATHWAY | hsa00380:Tryptophan metabolism | 2 | 12.5 | 0.065303 |
| GOTERM_CC_DIRECT | GO:0032588~trans-Golgi network membrane | 2 | 12.5 | 0.07258 |
| KEGG_PATHWAY | hsa00280:Valine, leucine and isoleucine degradation | 2 | 12.5 | 0.074304 |
| GOTERM_BP_DIRECT | GO:0006979~response to oxidative stress | 2 | 12.5 | 0.088416 |
| KEGG_PATHWAY | hsa00310:Lysine degradation | 2 | 12.5 | 0.096456 |

**Supplementary Table S2** | The metascape analysis of 16 cuproptosis regulators.

| Category | Term | LogP |
| --- | --- | --- |
| Reactome Gene Sets | R-HSA-389661 | -23.4003 |
| Reactome Gene Sets | R-HSA-389661 | -23.4003 |
| Reactome Gene Sets | R-HSA-71291 | -15.2502 |
| GO Biological Processes | GO:0006086 | -11.7366 |
| GO Biological Processes | GO:0006084 | -11.6097 |
| KEGG Pathway | hsa00020 | -11.5307 |
| KEGG Pathway | hsa01200 | -10.6841 |
| GO Biological Processes | GO:0006085 | -10.2812 |
| Reactome Gene Sets | R-HSA-71406 | -10.1464 |
| Reactome Gene Sets | R-HSA-204174 | -10.0219 |
| WikiPathways | WP2453 | -10.0219 |
| GO Biological Processes | GO:0006637 | -9.09915 |
| GO Biological Processes | GO:0035383 | -9.09915 |
| GO Biological Processes | GO:0061732 | -8.91439 |
| GO Biological Processes | GO:0006099 | -8.78619 |
| Reactome Gene Sets | R-HSA-70268 | -8.78619 |
| GO Biological Processes | GO:0033865 | -8.41632 |
| GO Biological Processes | GO:0033875 | -8.41632 |
| GO Biological Processes | GO:0034032 | -8.41632 |
| GO Biological Processes | GO:0035384 | -8.32436 |
| GO Biological Processes | GO:0071616 | -8.32436 |
| Reactome Gene Sets | R-HSA-5362517 | -8.19433 |
| WikiPathways | WP4290 | -8.15307 |
| GO Biological Processes | GO:0044272 | -8.07894 |
| KEGG Pathway | hsa00620 | -8.03492 |
| GO Biological Processes | GO:0006790 | -7.91828 |
| GO Biological Processes | GO:0009060 | -7.91384 |
| GO Biological Processes | GO:0033866 | -7.75451 |
| GO Biological Processes | GO:0034030 | -7.75451 |
| GO Biological Processes | GO:0034033 | -7.75451 |
| GO Biological Processes | GO:0006090 | -7.59993 |
| Reactome Gene Sets | R-HSA-1428517 | -7.55575 |
| GO Biological Processes | GO:0045333 | -7.53147 |
| GO Biological Processes | GO:0006091 | -7.50842 |
| KEGG Pathway | hsa00010 | -7.40449 |
| GO Biological Processes | GO:0015980 | -6.8818 |
| WikiPathways | WP3925 | -6.86536 |
| GO Biological Processes | GO:0009150 | -6.41408 |
| GO Biological Processes | GO:0006163 | -6.32369 |
| GO Biological Processes | GO:0009259 | -6.28994 |
| GO Biological Processes | GO:0019693 | -6.22403 |
| GO Biological Processes | GO:0072521 | -6.18546 |
| GO Biological Processes | GO:0009152 | -6.06464 |
| WikiPathways | WP5083 | -6.06464 |
| GO Biological Processes | GO:0006164 | -5.97086 |
| GO Biological Processes | GO:0009260 | -5.90377 |
| GO Biological Processes | GO:0072522 | -5.8712 |
| GO Biological Processes | GO:0046390 | -5.82872 |
| GO Biological Processes | GO:0009117 | -5.77111 |
| WikiPathways | WP534 | -5.76802 |
| GO Biological Processes | GO:0006753 | -5.7247 |
| GO Biological Processes | GO:0018130 | -5.69727 |
| GO Biological Processes | GO:0055086 | -5.41299 |
| GO Biological Processes | GO:0009165 | -5.38012 |
| GO Biological Processes | GO:0032787 | -5.37823 |
| GO Biological Processes | GO:1901293 | -5.36413 |
| GO Biological Processes | GO:1901137 | -5.20885 |
| KEGG Pathway | hsa05230 | -5.1852 |
| Reactome Gene Sets | R-HSA-9006931 | -4.80738 |
| GO Biological Processes | GO:0006006 | -4.73209 |
| GO Biological Processes | GO:0034654 | -4.58048 |
| GO Biological Processes | GO:0043603 | -4.48982 |
| GO Biological Processes | GO:0019438 | -4.35859 |
| GO Biological Processes | GO:0019318 | -4.27326 |
| GO Biological Processes | GO:0005996 | -4.08618 |
| GO Biological Processes | GO:0043604 | -4.028 |
| GO Biological Processes | GO:0090407 | -3.97922 |
| GO Biological Processes | GO:0005975 | -2.76558 |
| GO Biological Processes | GO:0015677 | -8.6135 |
| GO Biological Processes | GO:0015677 | -8.6135 |
| GO Biological Processes | GO:0035434 | -8.6135 |
| WikiPathways | WP3286 | -7.78716 |
| KEGG Pathway | hsa01524 | -7.25302 |
| GO Biological Processes | GO:0006825 | -7.16775 |
| GO Biological Processes | GO:0006878 | -7.16775 |
| GO Biological Processes | GO:0055070 | -6.93003 |
| KEGG Pathway | hsa04978 | -5.38783 |
| GO Biological Processes | GO:0000041 | -4.71901 |
| GO Biological Processes | GO:0046916 | -4.61888 |
| GO Biological Processes | GO:0055076 | -4.3488 |
| GO Biological Processes | GO:0050801 | -3.25856 |
| GO Biological Processes | GO:0010038 | -3.10889 |
| GO Biological Processes | GO:0010035 | -2.63604 |
| GO Biological Processes | GO:0006875 | -2.55033 |
| GO Biological Processes | GO:0098662 | -2.50437 |
| GO Biological Processes | GO:0030001 | -2.42163 |
| GO Biological Processes | GO:0055065 | -2.40775 |
| GO Biological Processes | GO:0030003 | -2.40381 |
| GO Biological Processes | GO:0006873 | -2.37472 |
| GO Biological Processes | GO:0098660 | -2.3709 |
| GO Biological Processes | GO:0098655 | -2.36519 |
| GO Biological Processes | GO:0055080 | -2.27057 |
| GO Biological Processes | GO:0098771 | -2.25325 |
| Reactome Gene Sets | R-HSA-382551 | -2.20781 |
| GO Biological Processes | GO:0055082 | -2.18012 |
| GO Biological Processes | GO:0009249 | -8.6135 |
| GO Biological Processes | GO:0009249 | -8.6135 |
| GO Biological Processes | GO:0051604 | -5.10639 |
| GO Biological Processes | GO:0018205 | -4.85949 |
| KEGG Pathway | hsa01240 | -4.16779 |
| GO Biological Processes | GO:0009063 | -8.60732 |
| GO Biological Processes | GO:0009063 | -8.60732 |
| GO Biological Processes | GO:0006520 | -8.38894 |
| GO Biological Processes | GO:0046395 | -7.09656 |
| GO Biological Processes | GO:0016054 | -7.05761 |
| GO Biological Processes | GO:0044282 | -6.07386 |
| GO Biological Processes | GO:1901605 | -5.54021 |
| GO Biological Processes | GO:1901606 | -4.82767 |
| GO Biological Processes | GO:0006979 | -3.05976 |
| GO Biological Processes | GO:0006979 | -3.05976 |
| Reactome Gene Sets | R-HSA-8953897 | -2.14064 |

**Supplementary Table S3** | The univariate COX regression model of 16 cuproptosis regulators in TCGA and GEO data.

| id | HR | HR.95L | HR.95H | pvalue | km |
| --- | --- | --- | --- | --- | --- |
| FDX1 | 0.740152 | 0.500301 | 1.094989 | 0.132109 | 0.018928 |
| LIPT1 | 0.956166 | 0.741383 | 1.233173 | 0.729857 | 0.246578 |
| LIAS | 0.84115 | 0.678219 | 1.043222 | 0.115311 | 0.001847 |
| DLD | 0.71647 | 0.52347 | 0.980629 | 0.037332 | 0.01016 |
| DLAT | 0.741644 | 0.551147 | 0.997983 | 0.048462 | 0.001574 |
| PDHA1 | 0.817459 | 0.606983 | 1.100918 | 0.184516 | 0.016278 |
| PDHB | 0.610378 | 0.402758 | 0.925025 | 0.019945 | 0.001473 |
| MTF1 | 1.160724 | 0.802075 | 1.679743 | 0.429308 | 0.127527 |
| GLS | 1.555123 | 1.113964 | 2.170992 | 0.009487 | 0.008571 |
| CDKN2A | 1.19452 | 1.052865 | 1.355232 | 0.005783 | 0.00017 |
| DBT | 1.134833 | 0.821841 | 1.567025 | 0.442343 | 0.153135 |
| DLST | 0.952889 | 0.685296 | 1.324972 | 0.774176 | 0.144488 |
| SLC31A1 | 0.778016 | 0.534115 | 1.133294 | 0.190891 | 0.067755 |
| ATP7A | 1.068786 | 0.861772 | 1.32553 | 0.544763 | 0.031277 |
| ATP7B | 0.887716 | 0.746798 | 1.055224 | 0.176864 | 0.055388 |

**Supplementary Table S4** | The CuproptosisCluster group of TCGA and GEO samples.

| ID | CuproptosisCluster |
| --- | --- |
| TCGA_TCGA-DM-A288 | A |
| TCGA_TCGA-QL-A97D | A |
| TCGA_TCGA-CM-6164 | B |
| TCGA_TCGA-G4-6299 | C |
| TCGA_TCGA-F4-6463 | B |
| TCGA_TCGA-AZ-4615 | B |
| TCGA_TCGA-AA-3549 | A |
| TCGA_TCGA-AY-4071 | B |
| TCGA_TCGA-CM-4752 | A |
| TCGA_TCGA-DM-A1D9 | A |
| TCGA_TCGA-AA-3688 | A |
| TCGA_TCGA-AA-3854 | B |
| TCGA_TCGA-A6-3809 | C |
| TCGA_TCGA-CM-6165 | A |
| TCGA_TCGA-CM-4751 | C |
| TCGA_TCGA-A6-5659 | A |
| TCGA_TCGA-AA-3494 | B |
| TCGA_TCGA-CM-4750 | B |
| TCGA_TCGA-AZ-4682 | A |
| TCGA_TCGA-G4-6625 | B |
| TCGA_TCGA-DM-A0XF | A |
| TCGA_TCGA-AA-3529 | C |
| TCGA_TCGA-AA-3949 | C |
| TCGA_TCGA-AA-3848 | B |
| TCGA_TCGA-CA-6715 | A |
| TCGA_TCGA-AA-3818 | A |
| TCGA_TCGA-AA-3710 | B |
| TCGA_TCGA-AA-3950 | A |
| TCGA_TCGA-AA-A00N | C |
| TCGA_TCGA-A6-6138 | B |
| TCGA_TCGA-DM-A28E | A |
| TCGA_TCGA-D5-6538 | C |
| TCGA_TCGA-AA-A00K | A |
| TCGA_TCGA-AA-3715 | B |
| TCGA_TCGA-AA-3506 | B |
| TCGA_TCGA-AU-3779 | A |
| TCGA_TCGA-CK-5916 | A |
| TCGA_TCGA-QG-A5Z1 | A |
| TCGA_TCGA-AD-6890 | A |
| TCGA_TCGA-A6-2685 | B |
| TCGA_TCGA-AA-A00W | B |
| TCGA_TCGA-CK-5914 | A |
| TCGA_TCGA-A6-2686 | C |
| TCGA_TCGA-CA-5255 | C |
| TCGA_TCGA-A6-A5ZU | C |
| TCGA_TCGA-NH-A50V | B |
| TCGA_TCGA-QG-A5YX | A |
| TCGA_TCGA-A6-2677 | A |
| TCGA_TCGA-CA-6717 | A |
| TCGA_TCGA-A6-6649 | B |
| TCGA_TCGA-NH-A6GC | B |
| TCGA_TCGA-AA-3562 | B |
| TCGA_TCGA-A6-4105 | B |
| TCGA_TCGA-A6-6141 | A |
| TCGA_TCGA-AA-A00R | A |
| TCGA_TCGA-AZ-4684 | B |
| TCGA_TCGA-CK-6747 | A |
| TCGA_TCGA-AU-6004 | A |
| TCGA_TCGA-A6-2672 | B |
| TCGA_TCGA-QG-A5Z2 | A |
| TCGA_TCGA-A6-6780 | C |
| TCGA_TCGA-F4-6459 | B |
| TCGA_TCGA-AA-3970 | B |
| TCGA_TCGA-NH-A8F7 | A |
| TCGA_TCGA-G4-6321 | A |
| TCGA_TCGA-AY-6196 | B |
| TCGA_TCGA-AA-3524 | C |
| TCGA_TCGA-DM-A1D0 | A |
| TCGA_TCGA-G4-6307 | B |
| TCGA_TCGA-DM-A1HB | C |
| TCGA_TCGA-AA-3697 | A |
| TCGA_TCGA-CM-6679 | A |
| TCGA_TCGA-AY-6197 | A |
| TCGA_TCGA-AA-3672 | C |
| TCGA_TCGA-AA-3684 | B |
| TCGA_TCGA-A6-2684 | B |
| TCGA_TCGA-AZ-4323 | B |
| TCGA_TCGA-AA-3971 | A |
| TCGA_TCGA-AA-3989 | C |
| TCGA_TCGA-AA-A00D | B |
| TCGA_TCGA-AD-6548 | A |
| TCGA_TCGA-D5-6539 | A |
| TCGA_TCGA-G4-6302 | B |
| TCGA_TCGA-A6-2679 | B |
| TCGA_TCGA-A6-2674 | B |
| TCGA_TCGA-AZ-4614 | C |
| TCGA_TCGA-NH-A5IV | A |
| TCGA_TCGA-A6-5664 | B |
| TCGA_TCGA-AZ-4315 | B |
| TCGA_TCGA-F4-6806 | A |
| TCGA_TCGA-AA-3510 | A |
| TCGA_TCGA-AY-A71X | A |
| TCGA_TCGA-AA-3488 | B |
| TCGA_TCGA-DM-A28F | A |
| TCGA_TCGA-G4-6293 | A |
| TCGA_TCGA-5M-AAT4 | A |
| TCGA_TCGA-D5-6533 | A |
| TCGA_TCGA-A6-5657 | B |
| TCGA_TCGA-AA-3941 | C |
| TCGA_TCGA-DM-A28K | A |
| TCGA_TCGA-CM-5344 | B |
| TCGA_TCGA-AA-3517 | A |
| TCGA_TCGA-G4-6309 | A |
| TCGA_TCGA-AY-6386 | A |
| TCGA_TCGA-AA-3678 | B |
| TCGA_TCGA-AA-A01X | B |
| TCGA_TCGA-AA-3821 | C |
| TCGA_TCGA-CM-6677 | A |
| TCGA_TCGA-CM-5861 | C |
| TCGA_TCGA-D5-6923 | B |
| TCGA_TCGA-F4-6703 | B |
| TCGA_TCGA-AZ-6605 | B |
| TCGA_TCGA-NH-A6GB | B |
| TCGA_TCGA-CM-5349 | A |
| TCGA_TCGA-NH-A50U | B |
| TCGA_TCGA-AA-3530 | A |
| TCGA_TCGA-G4-6627 | A |
| TCGA_TCGA-DM-A28G | A |
| TCGA_TCGA-D5-6537 | A |
| TCGA_TCGA-D5-5539 | B |
| TCGA_TCGA-CM-6163 | B |
| TCGA_TCGA-CM-4744 | C |
| TCGA_TCGA-AA-A004 | C |
| TCGA_TCGA-A6-3808 | B |
| TCGA_TCGA-AA-A010 | C |
| TCGA_TCGA-AA-A02J | A |
| TCGA_TCGA-AA-3837 | B |
| TCGA_TCGA-CK-4947 | A |
| TCGA_TCGA-CA-5256 | C |
| TCGA_TCGA-CM-6167 | B |
| TCGA_TCGA-4N-A93T | A |
| TCGA_TCGA-AA-3521 | A |
| TCGA_TCGA-CA-5797 | B |
| TCGA_TCGA-CM-6166 | A |
| TCGA_TCGA-CK-4951 | A |
| TCGA_TCGA-CA-6719 | B |
| TCGA_TCGA-AA-A02R | A |
| TCGA_TCGA-AA-A02O | B |
| TCGA_TCGA-A6-5666 | A |
| TCGA_TCGA-AY-5543 | A |
| TCGA_TCGA-AA-3845 | B |
| TCGA_TCGA-F4-6704 | B |
| TCGA_TCGA-AA-3856 | B |
| TCGA_TCGA-AA-A00Q | B |
| TCGA_TCGA-A6-6781 | B |
| TCGA_TCGA-AA-A00F | B |
| TCGA_TCGA-D5-6920 | A |
| TCGA_TCGA-AA-3815 | C |
| TCGA_TCGA-D5-5538 | A |
| TCGA_TCGA-AA-3867 | B |
| TCGA_TCGA-A6-6782 | B |
| TCGA_TCGA-A6-6654 | B |
| TCGA_TCGA-AA-A01D | B |
| TCGA_TCGA-G4-6311 | B |
| TCGA_TCGA-A6-3807 | C |
| TCGA_TCGA-AZ-5403 | C |
| TCGA_TCGA-AD-6888 | C |
| TCGA_TCGA-G4-6628 | A |
| TCGA_TCGA-DM-A1D6 | A |
| TCGA_TCGA-CM-5341 | C |
| TCGA_TCGA-AA-3862 | A |
| TCGA_TCGA-AA-3939 | A |
| TCGA_TCGA-AA-3514 | B |
| TCGA_TCGA-AA-3660 | B |
| TCGA_TCGA-AA-3833 | C |
| TCGA_TCGA-AA-A01P | B |
| TCGA_TCGA-AA-A00Z | B |
| TCGA_TCGA-CM-5863 | B |
| TCGA_TCGA-AZ-6598 | A |
| TCGA_TCGA-AA-3679 | B |
| TCGA_TCGA-G4-6304 | C |
| TCGA_TCGA-DM-A285 | B |
| TCGA_TCGA-G4-6294 | A |
| TCGA_TCGA-A6-4107 | B |
| TCGA_TCGA-DM-A1D4 | A |
| TCGA_TCGA-CA-6716 | A |
| TCGA_TCGA-CK-4952 | C |
| TCGA_TCGA-AA-A01V | B |
| TCGA_TCGA-AA-3844 | A |
| TCGA_TCGA-D5-6541 | A |
| TCGA_TCGA-AA-A01G | C |
| TCGA_TCGA-AA-3870 | B |
| TCGA_TCGA-D5-6532 | C |
| TCGA_TCGA-CM-6169 | B |
| TCGA_TCGA-D5-5541 | A |
| TCGA_TCGA-DM-A1DB | A |
| TCGA_TCGA-AA-A00A | A |
| TCGA_TCGA-D5-6530 | B |
| TCGA_TCGA-AA-3855 | B |
| TCGA_TCGA-CM-5868 | A |
| TCGA_TCGA-AA-3519 | B |
| TCGA_TCGA-AA-3526 | B |
| TCGA_TCGA-DM-A28M | A |
| TCGA_TCGA-D5-6926 | A |
| TCGA_TCGA-AA-3534 | A |
| TCGA_TCGA-G4-6317 | A |
| TCGA_TCGA-AA-A03J | B |
| TCGA_TCGA-CM-6680 | B |
| TCGA_TCGA-AA-A01K | B |
| TCGA_TCGA-AA-3984 | C |
| TCGA_TCGA-D5-6536 | B |
| TCGA_TCGA-AD-A5EK | C |
| TCGA_TCGA-AA-3663 | C |
| TCGA_TCGA-AZ-6607 | B |
| TCGA_TCGA-AA-3520 | B |
| TCGA_TCGA-AA-3543 | B |
| TCGA_TCGA-AZ-5407 | A |
| TCGA_TCGA-G4-6314 | A |
| TCGA_TCGA-CM-5348 | C |
| TCGA_TCGA-CK-6751 | A |
| TCGA_TCGA-D5-6929 | B |
| TCGA_TCGA-AA-3675 | A |
| TCGA_TCGA-A6-2681 | B |
| TCGA_TCGA-AA-3869 | A |
| TCGA_TCGA-DM-A282 | A |
| TCGA_TCGA-D5-7000 | A |
| TCGA_TCGA-AM-5821 | C |
| TCGA_TCGA-G4-6298 | B |
| TCGA_TCGA-AA-A01S | C |
| TCGA_TCGA-NH-A8F8 | A |
| TCGA_TCGA-AA-3966 | C |
| TCGA_TCGA-AA-3516 | C |
| TCGA_TCGA-A6-2676 | C |
| TCGA_TCGA-AA-3846 | B |
| TCGA_TCGA-AA-3544 | B |
| TCGA_TCGA-AA-3956 | A |
| TCGA_TCGA-AA-3532 | B |
| TCGA_TCGA-AA-3955 | A |
| TCGA_TCGA-CK-5912 | A |
| TCGA_TCGA-AD-6895 | B |
| TCGA_TCGA-AA-3556 | B |
| TCGA_TCGA-CM-6678 | A |
| TCGA_TCGA-AA-3851 | B |
| TCGA_TCGA-CM-5860 | C |
| TCGA_TCGA-A6-5661 | B |
| TCGA_TCGA-AA-A02F | B |
| TCGA_TCGA-AA-3861 | C |
| TCGA_TCGA-DM-A1DA | C |
| TCGA_TCGA-CM-6162 | B |
| TCGA_TCGA-AA-3522 | A |
| TCGA_TCGA-AA-A00U | A |
| TCGA_TCGA-F4-6807 | B |
| TCGA_TCGA-AA-3930 | B |
| TCGA_TCGA-AZ-6599 | B |
| TCGA_TCGA-AA-3947 | A |
| TCGA_TCGA-AA-3841 | C |
| TCGA_TCGA-CM-6171 | A |
| TCGA_TCGA-CM-4743 | A |
| TCGA_TCGA-AA-3814 | B |
| TCGA_TCGA-NH-A6GA | C |
| TCGA_TCGA-A6-2675 | B |
| TCGA_TCGA-G4-6297 | A |
| TCGA_TCGA-A6-5656 | A |
| TCGA_TCGA-DM-A0X9 | A |
| TCGA_TCGA-AA-3553 | C |
| TCGA_TCGA-DM-A0XD | C |
| TCGA_TCGA-AA-3511 | A |
| TCGA_TCGA-A6-A56B | A |
| TCGA_TCGA-A6-5667 | A |
| TCGA_TCGA-AA-3866 | A |
| TCGA_TCGA-AA-A017 | A |
| TCGA_TCGA-CM-6674 | A |
| TCGA_TCGA-DM-A1D8 | B |
| TCGA_TCGA-CM-6170 | A |
| TCGA_TCGA-AZ-4616 | C |
| TCGA_TCGA-AA-3994 | B |
| TCGA_TCGA-AA-3877 | A |
| TCGA_TCGA-WS-AB45 | B |
| TCGA_TCGA-AA-A00L | A |
| TCGA_TCGA-CM-5864 | A |
| TCGA_TCGA-CA-5254 | A |
| TCGA_TCGA-D5-6898 | A |
| TCGA_TCGA-AA-3696 | B |
| TCGA_TCGA-DM-A280 | A |
| TCGA_TCGA-AA-A00O | B |
| TCGA_TCGA-QG-A5YW | B |
| TCGA_TCGA-AD-6965 | B |
| TCGA_TCGA-CK-4948 | B |
| TCGA_TCGA-DM-A1HA | C |
| TCGA_TCGA-5M-AAT5 | B |
| TCGA_TCGA-F4-6809 | B |
| TCGA_TCGA-CM-4748 | B |
| TCGA_TCGA-AA-3858 | B |
| TCGA_TCGA-D5-6531 | C |
| TCGA_TCGA-AA-3662 | C |
| TCGA_TCGA-AM-5820 | C |
| TCGA_TCGA-AA-A02K | B |
| TCGA_TCGA-G4-6310 | A |
| TCGA_TCGA-AD-6889 | A |
| TCGA_TCGA-AA-3872 | B |
| TCGA_TCGA-AA-A01R | B |
| TCGA_TCGA-AA-3975 | B |
| TCGA_TCGA-5M-AAT6 | A |
| TCGA_TCGA-D5-6931 | B |
| TCGA_TCGA-D5-6924 | A |
| TCGA_TCGA-AA-3860 | B |
| TCGA_TCGA-A6-5665 | B |
| TCGA_TCGA-5M-AATA | C |
| TCGA_TCGA-D5-6540 | B |
| TCGA_TCGA-CA-6718 | C |
| TCGA_TCGA-AA-3986 | B |
| TCGA_TCGA-G4-6323 | B |
| TCGA_TCGA-A6-A566 | B |
| TCGA_TCGA-AZ-6608 | A |
| TCGA_TCGA-AA-3712 | B |
| TCGA_TCGA-A6-5662 | B |
| TCGA_TCGA-AA-3831 | A |
| TCGA_TCGA-AA-A02H | B |
| TCGA_TCGA-CM-6676 | A |
| TCGA_TCGA-AD-6899 | B |
| TCGA_TCGA-AA-3685 | B |
| TCGA_TCGA-CK-5915 | A |
| TCGA_TCGA-AA-3548 | B |
| TCGA_TCGA-F4-6569 | B |
| TCGA_TCGA-F4-6461 | B |
| TCGA_TCGA-F4-6854 | B |
| TCGA_TCGA-F4-6856 | A |
| TCGA_TCGA-AY-A54L | A |
| TCGA_TCGA-AA-3538 | B |
| TCGA_TCGA-AA-3842 | C |
| TCGA_TCGA-AZ-4308 | C |
| TCGA_TCGA-CK-6746 | A |
| TCGA_TCGA-AA-3509 | A |
| TCGA_TCGA-AA-3680 | B |
| TCGA_TCGA-AA-3542 | C |
| TCGA_TCGA-AD-6964 | B |
| TCGA_TCGA-A6-6650 | A |
| TCGA_TCGA-A6-6140 | A |
| TCGA_TCGA-AA-A024 | A |
| TCGA_TCGA-A6-2682 | B |
| TCGA_TCGA-CM-6675 | B |
| TCGA_TCGA-DM-A1D7 | A |
| TCGA_TCGA-4T-AA8H | A |
| TCGA_TCGA-A6-6653 | C |
| TCGA_TCGA-AA-3666 | B |
| TCGA_TCGA-F4-6460 | B |
| TCGA_TCGA-AA-3655 | C |
| TCGA_TCGA-AA-3673 | B |
| TCGA_TCGA-AA-3952 | B |
| TCGA_TCGA-T9-A92H | A |
| TCGA_TCGA-AY-4070 | C |
| TCGA_TCGA-AY-A8YK | A |
| TCGA_TCGA-CK-4950 | A |
| TCGA_TCGA-F4-6570 | A |
| TCGA_TCGA-AZ-6600 | C |
| TCGA_TCGA-AA-A00J | A |
| TCGA_TCGA-AA-3667 | B |
| TCGA_TCGA-AA-3554 | B |
| TCGA_TCGA-AZ-6606 | C |
| TCGA_TCGA-AA-3852 | C |
| TCGA_TCGA-D5-6922 | B |
| TCGA_TCGA-A6-6137 | A |
| TCGA_TCGA-AA-3495 | C |
| TCGA_TCGA-AA-3489 | B |
| TCGA_TCGA-G4-6322 | B |
| TCGA_TCGA-SS-A7HO | A |
| TCGA_TCGA-AA-3976 | C |
| TCGA_TCGA-AA-3811 | C |
| TCGA_TCGA-AD-A5EJ | C |
| TCGA_TCGA-AA-3819 | B |
| TCGA_TCGA-AA-A02Y | A |
| TCGA_TCGA-AZ-6603 | B |
| TCGA_TCGA-AA-A022 | B |
| TCGA_TCGA-G4-6626 | B |
| TCGA_TCGA-AA-3875 | B |
| TCGA_TCGA-G4-6306 | C |
| TCGA_TCGA-G4-6320 | B |
| TCGA_TCGA-CK-6748 | A |
| TCGA_TCGA-AA-3864 | C |
| TCGA_TCGA-A6-6142 | B |
| TCGA_TCGA-A6-2678 | C |
| TCGA_TCGA-CA-5796 | A |
| TCGA_TCGA-A6-A567 | A |
| TCGA_TCGA-AA-3713 | A |
| TCGA_TCGA-A6-6648 | A |
| TCGA_TCGA-A6-A565 | A |
| TCGA_TCGA-A6-5660 | A |
| TCGA_TCGA-AZ-6601 | B |
| TCGA_TCGA-5M-AATE | A |
| TCGA_TCGA-CM-6161 | A |
| TCGA_TCGA-AA-A01Q | C |
| TCGA_TCGA-AZ-4313 | C |
| TCGA_TCGA-A6-6652 | A |
| TCGA_TCGA-AA-A00E | B |
| TCGA_TCGA-F4-6808 | A |
| TCGA_TCGA-AA-A02W | A |
| TCGA_TCGA-DM-A28C | A |
| TCGA_TCGA-D5-6927 | A |
| TCGA_TCGA-AA-3980 | A |
| TCGA_TCGA-AA-3681 | C |
| TCGA_TCGA-AA-3492 | A |
| TCGA_TCGA-A6-2671 | B |
| TCGA_TCGA-D5-6535 | A |
| TCGA_TCGA-AA-A029 | A |
| TCGA_TCGA-AD-6963 | A |
| TCGA_TCGA-CM-4747 | B |
| TCGA_TCGA-NH-A50T | A |
| TCGA_TCGA-CM-6168 | A |
| TCGA_TCGA-AA-3518 | A |
| TCGA_TCGA-AA-A01T | B |
| TCGA_TCGA-RU-A8FL | A |
| TCGA_TCGA-A6-6651 | B |
| TCGA_TCGA-AA-3850 | A |
| TCGA_TCGA-AA-3972 | A |
| TCGA_TCGA-AA-3812 | C |
| TCGA_TCGA-AY-A69D | A |
| TCGA_TCGA-AA-A01Z | C |
| TCGA_TCGA-A6-3810 | A |
| TCGA_TCGA-DM-A28H | C |
| TCGA_TCGA-G4-6586 | C |
| TCGA_TCGA-D5-6928 | B |
| TCGA_TCGA-G4-6315 | A |
| TCGA_TCGA-D5-6534 | B |
| TCGA_TCGA-AA-3664 | C |
| TCGA_TCGA-G4-6303 | A |
| TCGA_TCGA-AA-3692 | B |
| TCGA_TCGA-AA-3968 | B |
| TCGA_TCGA-F4-6805 | B |
| TCGA_TCGA-AA-3560 | C |
| TCGA_TCGA-D5-5537 | B |
| TCGA_TCGA-CM-5862 | C |
| TCGA_TCGA-D5-6932 | A |
| TCGA_TCGA-AA-3982 | A |
| TCGA_TCGA-DM-A28A | C |
| TCGA_TCGA-CM-6172 | A |
| TCGA_TCGA-D5-6930 | B |
| TCGA_TCGA-D5-6529 | B |
| TCGA_TCGA-A6-2683 | B |
| TCGA_TCGA-CK-5913 | A |
| TCGA_TCGA-AA-3977 | C |
| TCGA_TCGA-AA-3496 | A |
| TCGA_TCGA-D5-5540 | A |
| TCGA_TCGA-AD-6901 | B |
| TCGA_TCGA-AA-A01F | A |
| TCGA_TCGA-AA-3552 | B |
| TCGA_TCGA-F4-6855 | B |
| TCGA_TCGA-AD-5900 | B |
| TCGA_TCGA-AA-3979 | C |
| TCGA_TCGA-AA-A01I | A |
| TCGA_TCGA-AA-A03F | B |
| TCGA_TCGA-3L-AA1B | B |
| TCGA_TCGA-AA-3693 | A |
| TCGA_TCGA-AA-3502 | B |
| TCGA_TCGA-AA-A02E | B |
| TCGA_TCGA-CM-4746 | B |
| TCGA_TCGA-G4-6295 | B |
| TCGA_TCGA-AA-A01C | B |
| TCGA_TCGA-G4-6588 | C |
| TCGA_TCGA-A6-2680 | B |
| TCGA_TCGA-QG-A5YV | A |
| TCGA_TCGA-AA-3561 | C |
| TCGA_TCGA-AA-3525 | A |
| TCGA_TCGA-AA-3531 | B |
| TCGA_TCGA-AA-3973 | A |
| TCGA_TCGA-AA-3527 | B |
| TCGA_TCGA-AA-3555 | B |
| TCGA_TCGA-AG-A025 | A |
| TCGA_TCGA-EI-6884 | A |
| TCGA_TCGA-AG-3582 | A |
| TCGA_TCGA-DC-6154 | A |
| TCGA_TCGA-AG-3611 | B |
| TCGA_TCGA-DY-A1DD | B |
| TCGA_TCGA-AG-3609 | B |
| TCGA_TCGA-AG-A01L | A |
| TCGA_TCGA-AG-4022 | A |
| TCGA_TCGA-AG-A01W | B |
| TCGA_TCGA-AG-A02N | B |
| TCGA_TCGA-AH-6549 | C |
| TCGA_TCGA-AH-6903 | A |
| TCGA_TCGA-AG-3727 | B |
| TCGA_TCGA-F5-6863 | B |
| TCGA_TCGA-AG-3887 | A |
| TCGA_TCGA-AG-A01J | B |
| TCGA_TCGA-AF-5654 | C |
| TCGA_TCGA-DY-A1DE | B |
| TCGA_TCGA-AF-3913 | A |
| TCGA_TCGA-AG-A014 | C |
| TCGA_TCGA-AG-4015 | B |
| TCGA_TCGA-G5-6572 | A |
| TCGA_TCGA-AG-A011 | C |
| TCGA_TCGA-EI-6511 | B |
| TCGA_TCGA-AG-4008 | B |
| TCGA_TCGA-AG-3731 | B |
| TCGA_TCGA-AG-A032 | A |
| TCGA_TCGA-AH-6544 | A |
| TCGA_TCGA-AG-A020 | C |
| TCGA_TCGA-AG-A026 | B |
| TCGA_TCGA-DY-A1H8 | A |
| TCGA_TCGA-AG-3883 | B |
| TCGA_TCGA-EI-7004 | B |
| TCGA_TCGA-AF-6136 | A |
| TCGA_TCGA-DC-4745 | A |
| TCGA_TCGA-EI-6883 | C |
| TCGA_TCGA-EI-6917 | C |
| TCGA_TCGA-F5-6861 | A |
| TCGA_TCGA-EI-6508 | A |
| TCGA_TCGA-F5-6465 | B |
| TCGA_TCGA-AF-6672 | A |
| TCGA_TCGA-AG-3885 | C |
| TCGA_TCGA-EI-6509 | B |
| TCGA_TCGA-DC-5337 | A |
| TCGA_TCGA-AG-3893 | B |
| TCGA_TCGA-AG-A02G | B |
| TCGA_TCGA-AG-3901 | B |
| TCGA_TCGA-AG-3726 | B |
| TCGA_TCGA-AG-3896 | A |
| TCGA_TCGA-DC-6160 | A |
| TCGA_TCGA-AG-3581 | B |
| TCGA_TCGA-G5-6641 | B |
| TCGA_TCGA-F5-6812 | B |
| TCGA_TCGA-AG-4001 | B |
| TCGA_TCGA-AF-2687 | B |
| TCGA_TCGA-AH-6644 | A |
| TCGA_TCGA-AG-3586 | A |
| TCGA_TCGA-AG-3909 | B |
| TCGA_TCGA-AG-3878 | B |
| TCGA_TCGA-AG-3592 | A |
| TCGA_TCGA-F5-6814 | C |
| TCGA_TCGA-AG-3605 | B |
| TCGA_TCGA-EI-6513 | A |
| TCGA_TCGA-DC-6158 | B |
| TCGA_TCGA-EI-6512 | B |
| TCGA_TCGA-AG-3601 | A |
| TCGA_TCGA-CI-6622 | A |
| TCGA_TCGA-BM-6198 | B |
| TCGA_TCGA-AG-3881 | B |
| TCGA_TCGA-DY-A1DC | C |
| TCGA_TCGA-AG-3612 | B |
| TCGA_TCGA-AG-3600 | B |
| TCGA_TCGA-AG-3599 | B |
| TCGA_TCGA-AG-3584 | C |
| TCGA_TCGA-DC-6156 | B |
| TCGA_TCGA-DT-5265 | B |
| TCGA_TCGA-AG-3902 | B |
| TCGA_TCGA-AG-A036 | B |
| TCGA_TCGA-AG-3593 | B |
| TCGA_TCGA-AG-A01Y | B |
| TCGA_TCGA-AG-3580 | B |
| TCGA_TCGA-AG-3742 | A |
| TCGA_TCGA-AG-A002 | C |
| TCGA_TCGA-DC-6683 | A |
| TCGA_TCGA-AF-6655 | B |
| TCGA_TCGA-AG-A023 | B |
| TCGA_TCGA-AG-3728 | B |
| TCGA_TCGA-AF-3400 | C |
| TCGA_TCGA-AF-2691 | C |
| TCGA_TCGA-CL-5918 | B |
| TCGA_TCGA-F5-6864 | A |
| TCGA_TCGA-AF-A56K | B |
| TCGA_TCGA-AG-4021 | A |
| TCGA_TCGA-AG-A01N | B |
| TCGA_TCGA-DC-5869 | A |
| TCGA_TCGA-AG-3608 | C |
| TCGA_TCGA-DY-A0XA | A |
| TCGA_TCGA-AG-3594 | C |
| TCGA_TCGA-EF-5831 | B |
| TCGA_TCGA-AG-3725 | A |
| TCGA_TCGA-EI-6514 | A |
| TCGA_TCGA-CI-6624 | A |
| TCGA_TCGA-CI-6620 | A |
| TCGA_TCGA-CI-6621 | A |
| TCGA_TCGA-EI-6510 | A |
| TCGA_TCGA-DY-A1DG | A |
| TCGA_TCGA-AG-A02X | C |
| TCGA_TCGA-AG-3587 | B |
| TCGA_TCGA-AG-3894 | C |
| TCGA_TCGA-AG-3999 | B |
| TCGA_TCGA-AG-A00H | B |
| TCGA_TCGA-AF-A56N | A |
| TCGA_TCGA-AG-A00C | C |
| TCGA_TCGA-EI-6885 | B |
| TCGA_TCGA-AG-3732 | B |
| TCGA_TCGA-AG-4007 | B |
| TCGA_TCGA-AG-A016 | C |
| TCGA_TCGA-EI-6882 | B |
| TCGA_TCGA-AG-3575 | C |
| TCGA_TCGA-AF-2693 | B |
| TCGA_TCGA-DC-6157 | A |
| TCGA_TCGA-AF-4110 | B |
| TCGA_TCGA-AG-3591 | B |
| TCGA_TCGA-EI-6881 | A |
| TCGA_TCGA-AG-A008 | A |
| TCGA_TCGA-AG-A015 | A |
| TCGA_TCGA-AG-3892 | B |
| TCGA_TCGA-G5-6233 | C |
| TCGA_TCGA-AG-3898 | B |
| TCGA_TCGA-AG-3578 | B |
| TCGA_TCGA-DC-6682 | C |
| TCGA_TCGA-F5-6813 | B |
| TCGA_TCGA-F5-6702 | B |
| TCGA_TCGA-CL-5917 | A |
| TCGA_TCGA-CL-4957 | A |
| TCGA_TCGA-EI-6507 | C |
| TCGA_TCGA-DC-4749 | A |
| TCGA_TCGA-AF-2690 | B |
| TCGA_TCGA-EF-5830 | C |
| TCGA_TCGA-AF-3911 | B |
| TCGA_TCGA-DC-6155 | B |
| TCGA_TCGA-DC-6681 | A |
| TCGA_TCGA-AF-A56L | A |
| TCGA_TCGA-CI-6623 | A |
| TCGA_TCGA-AH-6897 | A |
| TCGA_TCGA-AG-3574 | A |
| TCGA_TCGA-G5-6235 | A |
| TCGA_TCGA-AG-4005 | B |
| TCGA_TCGA-AG-3583 | A |
| TCGA_TCGA-AH-6547 | A |
| TCGA_TCGA-EI-7002 | A |
| TCGA_TCGA-AG-A00Y | B |
| TCGA_TCGA-EI-6506 | A |
| TCGA_TCGA-AH-6643 | A |
| TCGA_TCGA-AG-3890 | B |
| TCGA_TCGA-DY-A1DF | B |
| TCGA_TCGA-F5-6571 | A |
| TCGA_TCGA-AG-3882 | B |
| TCGA_TCGA-AG-3598 | C |
| TCGA_TCGA-F5-6811 | A |
| TCGA_TCGA-F5-6810 | B |
| TCGA_TCGA-F5-6464 | C |
| TCGA_TCGA-CI-6619 | B |
| TCGA_TCGA-AG-3602 | B |
| TCGA_TCGA-AF-2692 | A |
| GSE39582Cancer_GSM971957 | A |
| GSE39582Cancer_GSM971958 | B |
| GSE39582Cancer_GSM971959 | A |
| GSE39582Cancer_GSM971960 | A |
| GSE39582Cancer_GSM971961 | B |
| GSE39582Cancer_GSM971962 | B |
| GSE39582Cancer_GSM971963 | B |
| GSE39582Cancer_GSM971964 | C |
| GSE39582Cancer_GSM971965 | A |
| GSE39582Cancer_GSM971966 | C |
| GSE39582Cancer_GSM971967 | B |
| GSE39582Cancer_GSM971968 | B |
| GSE39582Cancer_GSM971969 | C |
| GSE39582Cancer_GSM971970 | A |
| GSE39582Cancer_GSM971971 | A |
| GSE39582Cancer_GSM971972 | A |
| GSE39582Cancer_GSM971973 | C |
| GSE39582Cancer_GSM971974 | A |
| GSE39582Cancer_GSM971975 | A |
| GSE39582Cancer_GSM971976 | B |
| GSE39582Cancer_GSM971977 | A |
| GSE39582Cancer_GSM971978 | C |
| GSE39582Cancer_GSM971979 | A |
| GSE39582Cancer_GSM971980 | A |
| GSE39582Cancer_GSM971981 | B |
| GSE39582Cancer_GSM971982 | A |
| GSE39582Cancer_GSM971983 | B |
| GSE39582Cancer_GSM971984 | A |
| GSE39582Cancer_GSM971985 | A |
| GSE39582Cancer_GSM971986 | B |
| GSE39582Cancer_GSM971987 | A |
| GSE39582Cancer_GSM971988 | C |
| GSE39582Cancer_GSM971989 | B |
| GSE39582Cancer_GSM971990 | A |
| GSE39582Cancer_GSM971991 | A |
| GSE39582Cancer_GSM971992 | A |
| GSE39582Cancer_GSM971993 | A |
| GSE39582Cancer_GSM971994 | C |
| GSE39582Cancer_GSM971995 | B |
| GSE39582Cancer_GSM971996 | B |
| GSE39582Cancer_GSM971997 | B |
| GSE39582Cancer_GSM971998 | B |
| GSE39582Cancer_GSM971999 | A |
| GSE39582Cancer_GSM972000 | A |
| GSE39582Cancer_GSM972001 | A |
| GSE39582Cancer_GSM972002 | A |
| GSE39582Cancer_GSM972003 | A |
| GSE39582Cancer_GSM972004 | B |
| GSE39582Cancer_GSM972005 | A |
| GSE39582Cancer_GSM972006 | B |
| GSE39582Cancer_GSM972007 | C |
| GSE39582Cancer_GSM972008 | C |
| GSE39582Cancer_GSM972009 | B |
| GSE39582Cancer_GSM972010 | A |
| GSE39582Cancer_GSM972011 | B |
| GSE39582Cancer_GSM972012 | A |
| GSE39582Cancer_GSM972013 | A |
| GSE39582Cancer_GSM972014 | A |
| GSE39582Cancer_GSM972015 | B |
| GSE39582Cancer_GSM972016 | C |
| GSE39582Cancer_GSM972017 | A |
| GSE39582Cancer_GSM972018 | A |
| GSE39582Cancer_GSM972019 | C |
| GSE39582Cancer_GSM972020 | A |
| GSE39582Cancer_GSM972021 | C |
| GSE39582Cancer_GSM972022 | A |
| GSE39582Cancer_GSM972023 | A |
| GSE39582Cancer_GSM972024 | A |
| GSE39582Cancer_GSM972025 | A |
| GSE39582Cancer_GSM972026 | C |
| GSE39582Cancer_GSM972027 | B |
| GSE39582Cancer_GSM972028 | B |
| GSE39582Cancer_GSM972029 | A |
| GSE39582Cancer_GSM972030 | B |
| GSE39582Cancer_GSM972031 | B |
| GSE39582Cancer_GSM972032 | A |
| GSE39582Cancer_GSM972033 | B |
| GSE39582Cancer_GSM972034 | B |
| GSE39582Cancer_GSM972035 | A |
| GSE39582Cancer_GSM972036 | A |
| GSE39582Cancer_GSM972037 | C |
| GSE39582Cancer_GSM972038 | A |
| GSE39582Cancer_GSM972039 | A |
| GSE39582Cancer_GSM972040 | A |
| GSE39582Cancer_GSM972041 | A |
| GSE39582Cancer_GSM972042 | A |
| GSE39582Cancer_GSM972043 | A |
| GSE39582Cancer_GSM972044 | B |
| GSE39582Cancer_GSM972045 | A |
| GSE39582Cancer_GSM972046 | B |
| GSE39582Cancer_GSM972047 | A |
| GSE39582Cancer_GSM972048 | A |
| GSE39582Cancer_GSM972049 | C |
| GSE39582Cancer_GSM972050 | A |
| GSE39582Cancer_GSM972051 | A |
| GSE39582Cancer_GSM972052 | A |
| GSE39582Cancer_GSM972053 | C |
| GSE39582Cancer_GSM972054 | B |
| GSE39582Cancer_GSM972055 | C |
| GSE39582Cancer_GSM972056 | A |
| GSE39582Cancer_GSM972057 | A |
| GSE39582Cancer_GSM972058 | B |
| GSE39582Cancer_GSM972059 | C |
| GSE39582Cancer_GSM972060 | A |
| GSE39582Cancer_GSM972061 | B |
| GSE39582Cancer_GSM972062 | A |
| GSE39582Cancer_GSM972063 | A |
| GSE39582Cancer_GSM972064 | B |
| GSE39582Cancer_GSM972065 | A |
| GSE39582Cancer_GSM972066 | A |
| GSE39582Cancer_GSM972067 | C |
| GSE39582Cancer_GSM972068 | B |
| GSE39582Cancer_GSM972069 | C |
| GSE39582Cancer_GSM972070 | A |
| GSE39582Cancer_GSM972071 | A |
| GSE39582Cancer_GSM972072 | A |
| GSE39582Cancer_GSM972073 | C |
| GSE39582Cancer_GSM972074 | A |
| GSE39582Cancer_GSM972075 | A |
| GSE39582Cancer_GSM972076 | B |
| GSE39582Cancer_GSM972077 | B |
| GSE39582Cancer_GSM972078 | A |
| GSE39582Cancer_GSM972079 | C |
| GSE39582Cancer_GSM972080 | B |
| GSE39582Cancer_GSM972081 | B |
| GSE39582Cancer_GSM972082 | B |
| GSE39582Cancer_GSM972083 | C |
| GSE39582Cancer_GSM972084 | A |
| GSE39582Cancer_GSM972085 | A |
| GSE39582Cancer_GSM972086 | C |
| GSE39582Cancer_GSM972087 | A |
| GSE39582Cancer_GSM972088 | A |
| GSE39582Cancer_GSM972089 | B |
| GSE39582Cancer_GSM972090 | A |
| GSE39582Cancer_GSM972091 | A |
| GSE39582Cancer_GSM972092 | C |
| GSE39582Cancer_GSM972093 | A |
| GSE39582Cancer_GSM972094 | C |
| GSE39582Cancer_GSM972095 | A |
| GSE39582Cancer_GSM972096 | A |
| GSE39582Cancer_GSM972097 | B |
| GSE39582Cancer_GSM972098 | B |
| GSE39582Cancer_GSM972099 | B |
| GSE39582Cancer_GSM972100 | A |
| GSE39582Cancer_GSM972101 | B |
| GSE39582Cancer_GSM972102 | B |
| GSE39582Cancer_GSM972103 | A |
| GSE39582Cancer_GSM972104 | B |
| GSE39582Cancer_GSM972105 | B |
| GSE39582Cancer_GSM972106 | A |
| GSE39582Cancer_GSM972107 | C |
| GSE39582Cancer_GSM972108 | A |
| GSE39582Cancer_GSM972109 | A |
| GSE39582Cancer_GSM972110 | B |
| GSE39582Cancer_GSM972111 | B |
| GSE39582Cancer_GSM972112 | C |
| GSE39582Cancer_GSM972113 | B |
| GSE39582Cancer_GSM972114 | A |
| GSE39582Cancer_GSM972115 | B |
| GSE39582Cancer_GSM972116 | B |
| GSE39582Cancer_GSM972117 | B |
| GSE39582Cancer_GSM972118 | C |
| GSE39582Cancer_GSM972119 | A |
| GSE39582Cancer_GSM972120 | B |
| GSE39582Cancer_GSM972121 | A |
| GSE39582Cancer_GSM972122 | A |
| GSE39582Cancer_GSM972123 | B |
| GSE39582Cancer_GSM972124 | B |
| GSE39582Cancer_GSM972125 | B |
| GSE39582Cancer_GSM972126 | B |
| GSE39582Cancer_GSM972127 | B |
| GSE39582Cancer_GSM972128 | B |
| GSE39582Cancer_GSM972129 | C |
| GSE39582Cancer_GSM972130 | B |
| GSE39582Cancer_GSM972131 | B |
| GSE39582Cancer_GSM972132 | A |
| GSE39582Cancer_GSM972133 | B |
| GSE39582Cancer_GSM972134 | A |
| GSE39582Cancer_GSM972135 | A |
| GSE39582Cancer_GSM972136 | A |
| GSE39582Cancer_GSM972137 | A |
| GSE39582Cancer_GSM972138 | B |
| GSE39582Cancer_GSM972139 | A |
| GSE39582Cancer_GSM972140 | B |
| GSE39582Cancer_GSM972141 | A |
| GSE39582Cancer_GSM972142 | A |
| GSE39582Cancer_GSM972143 | A |
| GSE39582Cancer_GSM972144 | B |
| GSE39582Cancer_GSM972145 | A |
| GSE39582Cancer_GSM972146 | B |
| GSE39582Cancer_GSM972147 | A |
| GSE39582Cancer_GSM972148 | A |
| GSE39582Cancer_GSM972149 | B |
| GSE39582Cancer_GSM972150 | A |
| GSE39582Cancer_GSM972151 | A |
| GSE39582Cancer_GSM972152 | A |
| GSE39582Cancer_GSM972153 | A |
| GSE39582Cancer_GSM972154 | B |
| GSE39582Cancer_GSM972155 | A |
| GSE39582Cancer_GSM972156 | A |
| GSE39582Cancer_GSM972157 | A |
| GSE39582Cancer_GSM972158 | B |
| GSE39582Cancer_GSM972159 | A |
| GSE39582Cancer_GSM972160 | A |
| GSE39582Cancer_GSM972161 | C |
| GSE39582Cancer_GSM972162 | B |
| GSE39582Cancer_GSM972163 | A |
| GSE39582Cancer_GSM972164 | A |
| GSE39582Cancer_GSM972165 | A |
| GSE39582Cancer_GSM972166 | A |
| GSE39582Cancer_GSM972167 | A |
| GSE39582Cancer_GSM972168 | B |
| GSE39582Cancer_GSM972169 | A |
| GSE39582Cancer_GSM972170 | A |
| GSE39582Cancer_GSM972171 | B |
| GSE39582Cancer_GSM972172 | C |
| GSE39582Cancer_GSM972173 | C |
| GSE39582Cancer_GSM972174 | A |
| GSE39582Cancer_GSM972175 | B |
| GSE39582Cancer_GSM972176 | C |
| GSE39582Cancer_GSM972177 | A |
| GSE39582Cancer_GSM972178 | A |
| GSE39582Cancer_GSM972179 | B |
| GSE39582Cancer_GSM972180 | C |
| GSE39582Cancer_GSM972181 | B |
| GSE39582Cancer_GSM972182 | C |
| GSE39582Cancer_GSM972183 | B |
| GSE39582Cancer_GSM972184 | B |
| GSE39582Cancer_GSM972185 | A |
| GSE39582Cancer_GSM972186 | C |
| GSE39582Cancer_GSM972187 | B |
| GSE39582Cancer_GSM972188 | A |
| GSE39582Cancer_GSM972189 | C |
| GSE39582Cancer_GSM972190 | B |
| GSE39582Cancer_GSM972191 | A |
| GSE39582Cancer_GSM972192 | B |
| GSE39582Cancer_GSM972193 | C |
| GSE39582Cancer_GSM972194 | B |
| GSE39582Cancer_GSM972195 | C |
| GSE39582Cancer_GSM972196 | B |
| GSE39582Cancer_GSM972197 | A |
| GSE39582Cancer_GSM972198 | A |
| GSE39582Cancer_GSM972199 | C |
| GSE39582Cancer_GSM972200 | A |
| GSE39582Cancer_GSM972201 | A |
| GSE39582Cancer_GSM972202 | B |
| GSE39582Cancer_GSM972203 | A |
| GSE39582Cancer_GSM972204 | A |
| GSE39582Cancer_GSM972205 | A |
| GSE39582Cancer_GSM972206 | B |
| GSE39582Cancer_GSM972207 | A |
| GSE39582Cancer_GSM972208 | A |
| GSE39582Cancer_GSM972209 | B |
| GSE39582Cancer_GSM972210 | A |
| GSE39582Cancer_GSM972211 | A |
| GSE39582Cancer_GSM972212 | B |
| GSE39582Cancer_GSM972213 | B |
| GSE39582Cancer_GSM972214 | C |
| GSE39582Cancer_GSM972215 | A |
| GSE39582Cancer_GSM972216 | C |
| GSE39582Cancer_GSM972217 | B |
| GSE39582Cancer_GSM972218 | A |
| GSE39582Cancer_GSM972219 | A |
| GSE39582Cancer_GSM972220 | B |
| GSE39582Cancer_GSM972221 | A |
| GSE39582Cancer_GSM972222 | A |
| GSE39582Cancer_GSM972223 | A |
| GSE39582Cancer_GSM972224 | B |
| GSE39582Cancer_GSM972225 | B |
| GSE39582Cancer_GSM972226 | A |
| GSE39582Cancer_GSM972227 | B |
| GSE39582Cancer_GSM972228 | A |
| GSE39582Cancer_GSM972229 | A |
| GSE39582Cancer_GSM972230 | A |
| GSE39582Cancer_GSM972231 | C |
| GSE39582Cancer_GSM972232 | C |
| GSE39582Cancer_GSM972233 | A |
| GSE39582Cancer_GSM972234 | B |
| GSE39582Cancer_GSM972235 | A |
| GSE39582Cancer_GSM972236 | A |
| GSE39582Cancer_GSM972237 | C |
| GSE39582Cancer_GSM972238 | A |
| GSE39582Cancer_GSM972239 | C |
| GSE39582Cancer_GSM972240 | C |
| GSE39582Cancer_GSM972241 | A |
| GSE39582Cancer_GSM972242 | A |
| GSE39582Cancer_GSM972243 | A |
| GSE39582Cancer_GSM972244 | B |
| GSE39582Cancer_GSM972245 | B |
| GSE39582Cancer_GSM972246 | A |
| GSE39582Cancer_GSM972247 | C |
| GSE39582Cancer_GSM972248 | B |
| GSE39582Cancer_GSM972249 | A |
| GSE39582Cancer_GSM972250 | A |
| GSE39582Cancer_GSM972251 | A |
| GSE39582Cancer_GSM972252 | A |
| GSE39582Cancer_GSM972253 | A |
| GSE39582Cancer_GSM972254 | C |
| GSE39582Cancer_GSM972255 | A |
| GSE39582Cancer_GSM972256 | B |
| GSE39582Cancer_GSM972257 | B |
| GSE39582Cancer_GSM972258 | B |
| GSE39582Cancer_GSM972259 | B |
| GSE39582Cancer_GSM972260 | B |
| GSE39582Cancer_GSM972261 | C |
| GSE39582Cancer_GSM972262 | B |
| GSE39582Cancer_GSM972263 | A |
| GSE39582Cancer_GSM972264 | A |
| GSE39582Cancer_GSM972265 | B |
| GSE39582Cancer_GSM972266 | A |
| GSE39582Cancer_GSM972267 | B |
| GSE39582Cancer_GSM972268 | B |
| GSE39582Cancer_GSM972269 | C |
| GSE39582Cancer_GSM972270 | A |
| GSE39582Cancer_GSM972271 | B |
| GSE39582Cancer_GSM972272 | C |
| GSE39582Cancer_GSM972273 | A |
| GSE39582Cancer_GSM972274 | B |
| GSE39582Cancer_GSM972275 | B |
| GSE39582Cancer_GSM972276 | B |
| GSE39582Cancer_GSM972277 | A |
| GSE39582Cancer_GSM972278 | B |
| GSE39582Cancer_GSM972279 | B |
| GSE39582Cancer_GSM972280 | C |
| GSE39582Cancer_GSM972281 | C |
| GSE39582Cancer_GSM972282 | A |
| GSE39582Cancer_GSM972283 | B |
| GSE39582Cancer_GSM972284 | A |
| GSE39582Cancer_GSM972285 | B |
| GSE39582Cancer_GSM972286 | A |
| GSE39582Cancer_GSM972287 | A |
| GSE39582Cancer_GSM972288 | A |
| GSE39582Cancer_GSM972289 | A |
| GSE39582Cancer_GSM972290 | A |
| GSE39582Cancer_GSM972291 | A |
| GSE39582Cancer_GSM972292 | B |
| GSE39582Cancer_GSM972293 | C |
| GSE39582Cancer_GSM972294 | C |
| GSE39582Cancer_GSM972295 | A |
| GSE39582Cancer_GSM972296 | A |
| GSE39582Cancer_GSM972297 | B |
| GSE39582Cancer_GSM972298 | A |
| GSE39582Cancer_GSM972299 | A |
| GSE39582Cancer_GSM972300 | B |
| GSE39582Cancer_GSM972301 | B |
| GSE39582Cancer_GSM972302 | A |
| GSE39582Cancer_GSM972303 | B |
| GSE39582Cancer_GSM972304 | B |
| GSE39582Cancer_GSM972305 | B |
| GSE39582Cancer_GSM972306 | B |
| GSE39582Cancer_GSM972307 | A |
| GSE39582Cancer_GSM972308 | A |
| GSE39582Cancer_GSM972309 | A |
| GSE39582Cancer_GSM972310 | A |
| GSE39582Cancer_GSM972311 | C |
| GSE39582Cancer_GSM972312 | A |
| GSE39582Cancer_GSM972313 | C |
| GSE39582Cancer_GSM972314 | A |
| GSE39582Cancer_GSM972315 | B |
| GSE39582Cancer_GSM972316 | C |
| GSE39582Cancer_GSM972317 | C |
| GSE39582Cancer_GSM972318 | A |
| GSE39582Cancer_GSM972319 | A |
| GSE39582Cancer_GSM972320 | A |
| GSE39582Cancer_GSM972321 | C |
| GSE39582Cancer_GSM972322 | B |
| GSE39582Cancer_GSM972323 | A |
| GSE39582Cancer_GSM972324 | A |
| GSE39582Cancer_GSM972325 | A |
| GSE39582Cancer_GSM972326 | B |
| GSE39582Cancer_GSM972327 | C |
| GSE39582Cancer_GSM972328 | B |
| GSE39582Cancer_GSM972329 | A |
| GSE39582Cancer_GSM972330 | B |
| GSE39582Cancer_GSM972331 | A |
| GSE39582Cancer_GSM972332 | B |
| GSE39582Cancer_GSM972333 | B |
| GSE39582Cancer_GSM972334 | B |
| GSE39582Cancer_GSM972335 | B |
| GSE39582Cancer_GSM972336 | A |
| GSE39582Cancer_GSM972337 | B |
| GSE39582Cancer_GSM972338 | A |
| GSE39582Cancer_GSM972339 | B |
| GSE39582Cancer_GSM972340 | A |
| GSE39582Cancer_GSM972341 | A |
| GSE39582Cancer_GSM972342 | A |
| GSE39582Cancer_GSM972343 | A |
| GSE39582Cancer_GSM972344 | B |
| GSE39582Cancer_GSM972345 | B |
| GSE39582Cancer_GSM972346 | C |
| GSE39582Cancer_GSM972347 | A |
| GSE39582Cancer_GSM972348 | C |
| GSE39582Cancer_GSM972349 | B |
| GSE39582Cancer_GSM972350 | A |
| GSE39582Cancer_GSM972351 | A |
| GSE39582Cancer_GSM972352 | A |
| GSE39582Cancer_GSM972353 | C |
| GSE39582Cancer_GSM972354 | C |
| GSE39582Cancer_GSM972355 | B |
| GSE39582Cancer_GSM972356 | A |
| GSE39582Cancer_GSM972357 | B |
| GSE39582Cancer_GSM972358 | A |
| GSE39582Cancer_GSM972359 | A |
| GSE39582Cancer_GSM972360 | B |
| GSE39582Cancer_GSM972361 | C |
| GSE39582Cancer_GSM972362 | A |
| GSE39582Cancer_GSM972363 | C |
| GSE39582Cancer_GSM972364 | B |
| GSE39582Cancer_GSM972365 | C |
| GSE39582Cancer_GSM972366 | B |
| GSE39582Cancer_GSM972367 | B |
| GSE39582Cancer_GSM972368 | B |
| GSE39582Cancer_GSM972369 | B |
| GSE39582Cancer_GSM972370 | A |
| GSE39582Cancer_GSM972371 | B |
| GSE39582Cancer_GSM972372 | B |
| GSE39582Cancer_GSM972373 | A |
| GSE39582Cancer_GSM972374 | B |
| GSE39582Cancer_GSM972375 | B |
| GSE39582Cancer_GSM972376 | B |
| GSE39582Cancer_GSM972377 | B |
| GSE39582Cancer_GSM972378 | B |
| GSE39582Cancer_GSM972379 | B |
| GSE39582Cancer_GSM972380 | B |
| GSE39582Cancer_GSM972381 | C |
| GSE39582Cancer_GSM972382 | A |
| GSE39582Cancer_GSM972383 | B |
| GSE39582Cancer_GSM972384 | A |
| GSE39582Cancer_GSM972385 | B |
| GSE39582Cancer_GSM972386 | A |
| GSE39582Cancer_GSM972387 | A |
| GSE39582Cancer_GSM972388 | A |
| GSE39582Cancer_GSM972389 | B |
| GSE39582Cancer_GSM972390 | B |
| GSE39582Cancer_GSM972391 | A |
| GSE39582Cancer_GSM972392 | A |
| GSE39582Cancer_GSM972393 | B |
| GSE39582Cancer_GSM972394 | B |
| GSE39582Cancer_GSM972395 | A |
| GSE39582Cancer_GSM972396 | A |
| GSE39582Cancer_GSM972397 | C |
| GSE39582Cancer_GSM972398 | A |
| GSE39582Cancer_GSM972399 | A |
| GSE39582Cancer_GSM972400 | A |
| GSE39582Cancer_GSM972401 | B |
| GSE39582Cancer_GSM972402 | A |
| GSE39582Cancer_GSM972403 | A |
| GSE39582Cancer_GSM972404 | A |
| GSE39582Cancer_GSM972405 | C |
| GSE39582Cancer_GSM972406 | C |
| GSE39582Cancer_GSM972407 | A |
| GSE39582Cancer_GSM972408 | A |
| GSE39582Cancer_GSM972409 | B |
| GSE39582Cancer_GSM972410 | B |
| GSE39582Cancer_GSM972411 | A |
| GSE39582Cancer_GSM972412 | B |
| GSE39582Cancer_GSM972413 | B |
| GSE39582Cancer_GSM972414 | A |
| GSE39582Cancer_GSM972415 | A |
| GSE39582Cancer_GSM972416 | B |
| GSE39582Cancer_GSM972417 | B |
| GSE39582Cancer_GSM972418 | B |
| GSE39582Cancer_GSM972419 | B |
| GSE39582Cancer_GSM972420 | B |
| GSE39582Cancer_GSM972421 | B |
| GSE39582Cancer_GSM972422 | C |
| GSE39582Cancer_GSM972423 | A |
| GSE39582Cancer_GSM972424 | B |
| GSE39582Cancer_GSM972425 | B |
| GSE39582Cancer_GSM972426 | B |
| GSE39582Cancer_GSM972427 | B |
| GSE39582Cancer_GSM972428 | B |
| GSE39582Cancer_GSM972429 | C |
| GSE39582Cancer_GSM972430 | C |
| GSE39582Cancer_GSM972431 | A |
| GSE39582Cancer_GSM972432 | A |
| GSE39582Cancer_GSM972433 | B |
| GSE39582Cancer_GSM972434 | A |
| GSE39582Cancer_GSM972435 | A |
| GSE39582Cancer_GSM972436 | A |
| GSE39582Cancer_GSM972437 | A |
| GSE39582Cancer_GSM972438 | C |
| GSE39582Cancer_GSM972439 | A |
| GSE39582Cancer_GSM972440 | B |
| GSE39582Cancer_GSM972441 | A |
| GSE39582Cancer_GSM972442 | A |
| GSE39582Cancer_GSM972443 | C |
| GSE39582Cancer_GSM972444 | B |
| GSE39582Cancer_GSM972445 | A |
| GSE39582Cancer_GSM972446 | C |
| GSE39582Cancer_GSM972447 | A |
| GSE39582Cancer_GSM972448 | A |
| GSE39582Cancer_GSM972449 | B |
| GSE39582Cancer_GSM972450 | A |
| GSE39582Cancer_GSM972451 | A |
| GSE39582Cancer_GSM972452 | B |
| GSE39582Cancer_GSM972453 | C |
| GSE39582Cancer_GSM972454 | B |
| GSE39582Cancer_GSM972455 | A |
| GSE39582Cancer_GSM972456 | C |
| GSE39582Cancer_GSM972457 | B |
| GSE39582Cancer_GSM972458 | A |
| GSE39582Cancer_GSM972459 | B |
| GSE39582Cancer_GSM972460 | A |
| GSE39582Cancer_GSM972461 | A |
| GSE39582Cancer_GSM972462 | B |
| GSE39582Cancer_GSM972463 | B |
| GSE39582Cancer_GSM972464 | A |
| GSE39582Cancer_GSM972465 | A |
| GSE39582Cancer_GSM972466 | C |
| GSE39582Cancer_GSM972467 | B |
| GSE39582Cancer_GSM972468 | A |
| GSE39582Cancer_GSM972469 | A |
| GSE39582Cancer_GSM972470 | B |
| GSE39582Cancer_GSM972471 | A |
| GSE39582Cancer_GSM972472 | B |
| GSE39582Cancer_GSM972473 | A |
| GSE39582Cancer_GSM972474 | A |
| GSE39582Cancer_GSM972475 | C |
| GSE39582Cancer_GSM972476 | A |
| GSE39582Cancer_GSM972477 | A |
| GSE39582Cancer_GSM972478 | B |
| GSE39582Cancer_GSM972479 | C |
| GSE39582Cancer_GSM972480 | A |
| GSE39582Cancer_GSM972481 | B |
| GSE39582Cancer_GSM972482 | A |
| GSE39582Cancer_GSM972483 | A |
| GSE39582Cancer_GSM972484 | B |
| GSE39582Cancer_GSM972485 | B |
| GSE39582Cancer_GSM972486 | A |
| GSE39582Cancer_GSM972487 | A |
| GSE39582Cancer_GSM972488 | B |
| GSE39582Cancer_GSM972489 | B |
| GSE39582Cancer_GSM972490 | A |
| GSE39582Cancer_GSM972491 | A |
| GSE39582Cancer_GSM972492 | C |
| GSE39582Cancer_GSM972493 | A |
| GSE39582Cancer_GSM972494 | B |
| GSE39582Cancer_GSM972495 | A |
| GSE39582Cancer_GSM972496 | A |
| GSE39582Cancer_GSM972497 | A |
| GSE39582Cancer_GSM972498 | A |
| GSE39582Cancer_GSM972499 | A |
| GSE39582Cancer_GSM972500 | B |
| GSE39582Cancer_GSM972501 | B |
| GSE39582Cancer_GSM972502 | C |
| GSE39582Cancer_GSM972503 | B |
| GSE39582Cancer_GSM972504 | B |
| GSE39582Cancer_GSM972505 | B |
| GSE39582Cancer_GSM972506 | B |
| GSE39582Cancer_GSM972507 | A |
| GSE39582Cancer_GSM972508 | A |
| GSE39582Cancer_GSM972509 | A |
| GSE39582Cancer_GSM972510 | A |
| GSE39582Cancer_GSM972511 | C |
| GSE39582Cancer_GSM972512 | C |
| GSE39582Cancer_GSM972513 | A |
| GSE39582Cancer_GSM972514 | A |
| GSE39582Cancer_GSM972515 | A |
| GSE39582Cancer_GSM972516 | A |
| GSE39582Cancer_GSM972517 | B |
| GSE39582Cancer_GSM972518 | B |
| GSE39582Cancer_GSM972519 | B |
| GSE39582Cancer_GSM972520 | C |
| GSE39582Cancer_GSM972521 | B |
| GSE39582Cancer_GSM972522 | B |

**Supplementary Table S5** | The immune score, stromal score and tumor purity of TCGA and GEO samples by ESTIMATE algorithm.

| Sample | StromalScore | ImmuneScore | ESTIMATEScore | TumorPurity |
| --- | --- | --- | --- | --- |
| TCGA_TCGA-DM-A288 | -1064.36 | 561.9183 | -502.44 | 0.862182 |
| TCGA_TCGA-QL-A97D | -288.84 | 3139.759 | 2850.92 | 0.520407 |
| TCGA_TCGA-CM-6164 | 858.998 | 2553.859 | 3412.857 | 0.448281 |
| TCGA_TCGA-G4-6299 | 1707.619 | 3735.973 | 5443.592 | 0.165981 |
| TCGA_TCGA-F4-6463 | 1913.894 | 2580.694 | 4494.588 | 0.3013 |
| TCGA_TCGA-AZ-4615 | 1779.649 | 4122.623 | 5902.272 | 0.09926 |
| TCGA_TCGA-AA-3549 | 591.5384 | 2883.142 | 3474.681 | 0.440151 |
| TCGA_TCGA-AY-4071 | 1456.977 | 4115.45 | 5572.427 | 0.147304 |
| TCGA_TCGA-CM-4752 | 1490.017 | 3634.081 | 5124.097 | 0.212029 |
| TCGA_TCGA-DM-A1D9 | -453.759 | 968.8853 | 515.126 | 0.777194 |
| TCGA_TCGA-AA-3688 | 496.4294 | 2599.463 | 3095.892 | 0.489371 |
| TCGA_TCGA-AA-3854 | 6.278004 | 2172.351 | 2178.629 | 0.602007 |
| TCGA_TCGA-A6-3809 | 1908.267 | 4445.169 | 6353.437 | 0.033192 |
| TCGA_TCGA-CM-6165 | 2104.664 | 3127.329 | 5231.993 | 0.196526 |
| TCGA_TCGA-CM-4751 | 1424.006 | 3642.721 | 5066.728 | 0.220252 |
| TCGA_TCGA-A6-5659 | 868.7894 | 1197.642 | 2066.431 | 0.615075 |
| TCGA_TCGA-AA-3494 | 189.3199 | 2441.201 | 2630.521 | 0.547756 |
| TCGA_TCGA-CM-4750 | 405.6487 | 2372.008 | 2777.657 | 0.52956 |
| TCGA_TCGA-AZ-4682 | 44.32294 | 1971.402 | 2015.725 | 0.620927 |
| TCGA_TCGA-G4-6625 | 2076.499 | 4577.872 | 6654.371 | NA |
| TCGA_TCGA-DM-A0XF | 549.7634 | 2163.414 | 2713.178 | 0.537565 |
| TCGA_TCGA-AA-3529 | 259.3841 | 2158.636 | 2418.02 | 0.573582 |
| TCGA_TCGA-AA-3949 | 2311.098 | 5039.506 | 7350.604 | NA |
| TCGA_TCGA-AA-3848 | 48.56644 | 2354.055 | 2402.621 | 0.575432 |
| TCGA_TCGA-CA-6715 | -631.006 | 662.3765 | 31.37057 | 0.819882 |
| TCGA_TCGA-AA-3818 | 373.9722 | 2342.699 | 2716.672 | 0.537132 |
| TCGA_TCGA-AA-3710 | 1656.421 | 4988.427 | 6644.848 | NA |
| TCGA_TCGA-AA-3950 | 2506.206 | 4737.946 | 7244.152 | NA |
| TCGA_TCGA-AA-A00N | 2199.159 | 3138.845 | 5338.004 | 0.181245 |
| TCGA_TCGA-A6-6138 | 2274.187 | 4420.467 | 6694.655 | NA |
| TCGA_TCGA-DM-A28E | -1832.56 | 640.124 | -1192.44 | 0.908986 |
| TCGA_TCGA-D5-6538 | -929.892 | -17.1101 | -947.002 | 0.893383 |
| TCGA_TCGA-AA-A00K | 411.6968 | 2485.168 | 2896.865 | 0.514636 |
| TCGA_TCGA-AA-3715 | 2498.546 | 4663.922 | 7162.468 | NA |
| TCGA_TCGA-AA-3506 | 1148.319 | 3481.811 | 4630.13 | 0.28227 |
| TCGA_TCGA-AU-3779 | 1603.47 | 3750.239 | 5353.709 | 0.178977 |
| TCGA_TCGA-CK-5916 | 1790.147 | 4309.864 | 6100.011 | 0.07034 |
| TCGA_TCGA-QG-A5Z1 | 1108.569 | 1608.098 | 2716.667 | 0.537133 |
| TCGA_TCGA-AD-6890 | 695.8661 | 2306.896 | 3002.762 | 0.501246 |
| TCGA_TCGA-A6-2685 | 2850.221 | 4178.013 | 7028.234 | NA |
| TCGA_TCGA-AA-A00W | -1882.11 | 1744.821 | -137.291 | 0.833803 |
| TCGA_TCGA-CK-5914 | 882.3306 | 2200.421 | 3082.752 | 0.491052 |
| TCGA_TCGA-A6-2686 | 2194.031 | 4861.706 | 7055.737 | NA |
| TCGA_TCGA-CA-5255 | -1430.2 | 1122.287 | -307.916 | 0.847367 |
| TCGA_TCGA-A6-A5ZU | 1982.47 | 3442.567 | 5425.037 | 0.168667 |
| TCGA_TCGA-NH-A50V | 1757.931 | 3104.136 | 4862.066 | 0.249452 |
| TCGA_TCGA-QG-A5YX | -1476.29 | 1301.792 | -174.493 | 0.836806 |
| TCGA_TCGA-A6-2677 | 411.5947 | 1228.682 | 1640.276 | 0.663162 |
| TCGA_TCGA-CA-6717 | 2850.036 | 4361.813 | 7211.849 | NA |
| TCGA_TCGA-A6-6649 | 1626.739 | 3362.693 | 4989.433 | 0.231304 |
| TCGA_TCGA-NH-A6GC | 973.6192 | 1578.777 | 2552.397 | 0.557314 |
| TCGA_TCGA-AA-3562 | 1218.532 | 2938.323 | 4156.855 | 0.348182 |
| TCGA_TCGA-A6-4105 | 1865.106 | 3978.922 | 5844.028 | 0.107764 |
| TCGA_TCGA-A6-6141 | 795.5752 | 3459.508 | 4255.083 | 0.33463 |
| TCGA_TCGA-AA-A00R | 1296.225 | 4906.603 | 6202.828 | 0.055278 |
| TCGA_TCGA-AZ-4684 | 1704.287 | 3595.628 | 5299.914 | 0.186741 |
| TCGA_TCGA-CK-6747 | 823.3702 | 2599.849 | 3423.22 | 0.446921 |
| TCGA_TCGA-AU-6004 | 1709.252 | 3916.645 | 5625.897 | 0.139536 |
| TCGA_TCGA-A6-2672 | 1793.039 | 4642.878 | 6435.916 | 0.02109 |
| TCGA_TCGA-QG-A5Z2 | -1343.84 | 3305.552 | 1961.709 | 0.627122 |
| TCGA_TCGA-A6-6780 | 1673.664 | 4745.989 | 6419.653 | 0.023476 |
| TCGA_TCGA-F4-6459 | 2448.92 | 2673.64 | 5122.56 | 0.21225 |
| TCGA_TCGA-AA-3970 | 481.9335 | 3391.091 | 3873.025 | 0.386925 |
| TCGA_TCGA-NH-A8F7 | -1676.01 | -143.893 | -1819.91 | 0.943471 |
| TCGA_TCGA-G4-6321 | -715.97 | 3304.966 | 2588.995 | 0.552845 |
| TCGA_TCGA-AY-6196 | 4160.101 | 5814.485 | 9974.585 | NA |
| TCGA_TCGA-AA-3524 | 563.048 | 2539.842 | 3102.89 | 0.488475 |
| TCGA_TCGA-DM-A1D0 | -1850.59 | -145.501 | -1996.09 | 0.951727 |
| TCGA_TCGA-G4-6307 | -1433.24 | 685.959 | -747.282 | 0.879829 |
| TCGA_TCGA-DM-A1HB | -370.035 | 1407.91 | 1037.875 | 0.726669 |
| TCGA_TCGA-AA-3697 | 516.6708 | 2810.48 | 3327.151 | 0.459491 |
| TCGA_TCGA-CM-6679 | 2146.168 | 3217.193 | 5363.361 | 0.177583 |
| TCGA_TCGA-AY-6197 | -442.119 | 2571.354 | 2129.235 | 0.60778 |
| TCGA_TCGA-AA-3672 | 1191.662 | 4219.55 | 5411.212 | 0.170666 |
| TCGA_TCGA-AA-3684 | 2539.548 | 4012.849 | 6552.398 | 0.003993 |
| TCGA_TCGA-A6-2684 | 2863.898 | 3661.491 | 6525.389 | 0.007958 |
| TCGA_TCGA-AZ-4323 | 2224.951 | 5042.211 | 7267.161 | NA |
| TCGA_TCGA-AA-3971 | 886.4402 | 3803.782 | 4690.222 | 0.273797 |
| TCGA_TCGA-AA-3989 | 1631.867 | 3657.745 | 5289.611 | 0.188226 |
| TCGA_TCGA-AA-A00D | 1798.821 | 4617.797 | 6416.618 | 0.023922 |
| TCGA_TCGA-AD-6548 | 1695.108 | 3710.69 | 5405.798 | 0.171449 |
| TCGA_TCGA-D5-6539 | 452.3279 | 2938.149 | 3390.477 | 0.451215 |
| TCGA_TCGA-G4-6302 | 3182.871 | 3760.118 | 6942.989 | NA |
| TCGA_TCGA-A6-2679 | 890.9722 | 3692.691 | 4583.663 | 0.288807 |
| TCGA_TCGA-A6-2674 | 3288.425 | 4665.528 | 7953.953 | NA |
| TCGA_TCGA-AZ-4614 | -1411.37 | 1511.927 | 100.5539 | 0.814025 |
| TCGA_TCGA-NH-A5IV | 1182.562 | 3693.554 | 4876.116 | 0.247454 |
| TCGA_TCGA-A6-5664 | 2581.377 | 4011.216 | 6592.593 | NA |
| TCGA_TCGA-AZ-4315 | 938.2714 | 3031.332 | 3969.603 | 0.373814 |
| TCGA_TCGA-F4-6806 | 886.2118 | 2569.841 | 3456.053 | 0.442604 |
| TCGA_TCGA-AA-3510 | 937.2972 | 3659.348 | 4596.645 | 0.286982 |
| TCGA_TCGA-AY-A71X | -2760.52 | 212.9654 | -2547.55 | 0.97343 |
| TCGA_TCGA-AA-3488 | -393.09 | 2161.864 | 1768.773 | 0.648927 |
| TCGA_TCGA-DM-A28F | -701.328 | 1335.842 | 634.5148 | 0.766048 |
| TCGA_TCGA-G4-6293 | 631.443 | 3694.599 | 4326.042 | 0.324796 |
| TCGA_TCGA-5M-AAT4 | -692.599 | 768.6221 | 76.02298 | 0.816111 |
| TCGA_TCGA-D5-6533 | 834.7543 | 2393.897 | 3228.651 | 0.472284 |
| TCGA_TCGA-A6-5657 | 1907.35 | 3312.291 | 5219.642 | 0.198303 |
| TCGA_TCGA-AA-3941 | -254.468 | 1905.355 | 1650.887 | 0.661996 |
| TCGA_TCGA-DM-A28K | -822.785 | 1256.286 | 433.501 | 0.784678 |
| TCGA_TCGA-CM-5344 | 1536.238 | 1817.599 | 3353.837 | 0.456008 |
| TCGA_TCGA-AA-3517 | 920.6732 | 2902.013 | 3822.687 | 0.393727 |
| TCGA_TCGA-G4-6309 | -370.152 | 2050.925 | 1680.773 | 0.658701 |
| TCGA_TCGA-AY-6386 | 46.86245 | 2930.596 | 2977.459 | 0.504457 |
| TCGA_TCGA-AA-3678 | 414.2429 | 3381.155 | 3795.398 | 0.397407 |
| TCGA_TCGA-AA-A01X | 43.57533 | 1702.93 | 1746.505 | 0.651411 |
| TCGA_TCGA-AA-3821 | 1439.899 | 2990.256 | 4430.154 | 0.310305 |
| TCGA_TCGA-CM-6677 | 1612.897 | 2914.26 | 4527.156 | 0.296738 |
| TCGA_TCGA-CM-5861 | -293.279 | 1688.83 | 1395.551 | 0.689616 |
| TCGA_TCGA-D5-6923 | 1924.969 | 2545.844 | 4470.813 | 0.304626 |
| TCGA_TCGA-F4-6703 | 4082.294 | 5497.649 | 9579.943 | NA |
| TCGA_TCGA-AZ-6605 | 2981.279 | 4153.22 | 7134.499 | NA |
| TCGA_TCGA-NH-A6GB | 143.3552 | 2842.865 | 2986.221 | 0.503346 |
| TCGA_TCGA-CM-5349 | 1771.518 | 3201.415 | 4972.934 | 0.23366 |
| TCGA_TCGA-NH-A50U | 451.0088 | 1467.211 | 1918.22 | 0.632082 |
| TCGA_TCGA-AA-3530 | -103.879 | 2885.429 | 2781.55 | 0.529075 |
| TCGA_TCGA-G4-6627 | 2298.068 | 4033.718 | 6331.786 | 0.036368 |
| TCGA_TCGA-DM-A28G | -536.228 | 1573.302 | 1037.074 | 0.72675 |
| TCGA_TCGA-D5-6537 | -929.924 | 1872.464 | 942.5403 | 0.736212 |
| TCGA_TCGA-D5-5539 | 1914.864 | 3228.486 | 5143.35 | 0.209267 |
| TCGA_TCGA-CM-6163 | 1801.229 | 3629.376 | 5430.605 | 0.167861 |
| TCGA_TCGA-CM-4744 | -532.471 | 2979.733 | 2447.262 | 0.570061 |
| TCGA_TCGA-AA-A004 | 777.3857 | 3211.652 | 3989.038 | 0.371166 |
| TCGA_TCGA-A6-3808 | 2652.247 | 4074.378 | 6726.625 | NA |
| TCGA_TCGA-AA-A010 | 186.8248 | 2598.666 | 2785.49 | 0.528584 |
| TCGA_TCGA-AA-A02J | -1820.13 | 236.8461 | -1583.28 | 0.931391 |
| TCGA_TCGA-AA-3837 | 981.6176 | 2495.321 | 3476.939 | 0.439853 |
| TCGA_TCGA-CK-4947 | 1643.961 | 3734.612 | 5378.572 | 0.175385 |
| TCGA_TCGA-CA-5256 | 456.1434 | 2729.578 | 3185.722 | 0.477829 |
| TCGA_TCGA-CM-6167 | 3438.281 | 3926.24 | 7364.521 | NA |
| TCGA_TCGA-4N-A93T | -1937.55 | 953.4249 | -984.128 | 0.895818 |
| TCGA_TCGA-AA-3521 | 959.1134 | 2390.363 | 3349.477 | 0.456578 |
| TCGA_TCGA-CA-5797 | 1383.904 | 2478.789 | 3862.694 | 0.388323 |
| TCGA_TCGA-CM-6166 | 342.4494 | 599.5663 | 942.0158 | 0.736264 |
| TCGA_TCGA-CK-4951 | 2146.858 | 4113.828 | 6260.686 | 0.046796 |
| TCGA_TCGA-CA-6719 | 2187.168 | 3190.083 | 5377.251 | 0.175576 |
| TCGA_TCGA-AA-A02R | 1507.803 | 4440.167 | 5947.97 | 0.092583 |
| TCGA_TCGA-AA-A02O | -254.868 | 2248.786 | 1993.917 | 0.623433 |
| TCGA_TCGA-A6-5666 | -697.451 | 1210.294 | 512.8434 | 0.777405 |
| TCGA_TCGA-AY-5543 | -319.391 | 2730.015 | 2410.624 | 0.574471 |
| TCGA_TCGA-AA-3845 | 1161.204 | 3733.376 | 4894.58 | 0.244827 |
| TCGA_TCGA-F4-6704 | 3085.247 | 3485.088 | 6570.335 | 0.00136 |
| TCGA_TCGA-AA-3856 | 887.5012 | 4004.599 | 4892.1 | 0.24518 |
| TCGA_TCGA-AA-A00Q | 549.8993 | 2322.378 | 2872.278 | 0.517727 |
| TCGA_TCGA-A6-6781 | 3934.615 | 5002.801 | 8937.415 | NA |
| TCGA_TCGA-AA-A00F | 1034.028 | 2187.346 | 3221.374 | 0.473226 |
| TCGA_TCGA-D5-6920 | 667.6909 | 3421.304 | 4088.995 | 0.357502 |
| TCGA_TCGA-AA-3815 | 1071.989 | 4465.425 | 5537.414 | 0.152385 |
| TCGA_TCGA-D5-5538 | 2285.467 | 3992.423 | 6277.89 | 0.044273 |
| TCGA_TCGA-AA-3867 | 2070.214 | 2780.666 | 4850.88 | 0.251042 |
| TCGA_TCGA-A6-6782 | 2600.224 | 3793.528 | 6393.751 | 0.027277 |
| TCGA_TCGA-A6-6654 | 3359.237 | 4949.274 | 8308.512 | NA |
| TCGA_TCGA-AA-A01D | 1954.604 | 3157.73 | 5112.333 | 0.213717 |
| TCGA_TCGA-G4-6311 | 1593.502 | 2976.415 | 4569.917 | 0.290738 |
| TCGA_TCGA-A6-3807 | 1431.38 | 3391.831 | 4823.211 | 0.254971 |
| TCGA_TCGA-AZ-5403 | 1812.494 | 2473.989 | 4286.483 | 0.330283 |
| TCGA_TCGA-AD-6888 | -2506.62 | 956.238 | -1550.38 | 0.929623 |
| TCGA_TCGA-G4-6628 | 1718.527 | 4733.486 | 6452.012 | 0.018727 |
| TCGA_TCGA-DM-A1D6 | -1634.21 | -325.656 | -1959.87 | 0.950081 |
| TCGA_TCGA-CM-5341 | 2653.55 | 4221.046 | 6874.597 | NA |
| TCGA_TCGA-AA-3862 | 872.1491 | 3686.976 | 4559.125 | 0.292254 |
| TCGA_TCGA-AA-3939 | 978.1859 | 3226.574 | 4204.76 | 0.341582 |
| TCGA_TCGA-AA-3514 | 1746.411 | 2612.238 | 4358.649 | 0.320266 |
| TCGA_TCGA-AA-3660 | 737.3017 | 2318.635 | 3055.937 | 0.494477 |
| TCGA_TCGA-AA-3833 | 1806.627 | 3786.524 | 5593.151 | 0.144294 |
| TCGA_TCGA-AA-A01P | 2270.199 | 4795.284 | 7065.483 | NA |
| TCGA_TCGA-AA-A00Z | -28.6443 | 2078.98 | 2050.336 | 0.616936 |
| TCGA_TCGA-CM-5863 | 1939.031 | 2946.482 | 4885.512 | 0.246118 |
| TCGA_TCGA-AZ-6598 | 558.1043 | 3357.43 | 3915.534 | 0.381163 |
| TCGA_TCGA-AA-3679 | 713.4835 | 2600.168 | 3313.651 | 0.46125 |
| TCGA_TCGA-G4-6304 | -1463.69 | 2116.002 | 652.3125 | 0.764366 |
| TCGA_TCGA-DM-A285 | 622.2571 | 632.501 | 1254.758 | 0.704434 |
| TCGA_TCGA-G4-6294 | -38.3565 | 1990.372 | 1952.016 | 0.62823 |
| TCGA_TCGA-A6-4107 | 1726.455 | 3290.071 | 5016.526 | 0.227434 |
| TCGA_TCGA-DM-A1D4 | -1839.88 | 1530.149 | -309.726 | 0.847508 |
| TCGA_TCGA-CA-6716 | 681.5232 | 940.4723 | 1621.996 | 0.665168 |
| TCGA_TCGA-CK-4952 | 47.56635 | 2192.832 | 2240.399 | 0.594742 |
| TCGA_TCGA-AA-A01V | -1410.55 | 2525.222 | 1114.672 | 0.718879 |
| TCGA_TCGA-AA-3844 | 174.7655 | 2829.842 | 3004.608 | 0.501012 |
| TCGA_TCGA-D5-6541 | 2441.07 | 3943.21 | 6384.28 | 0.028667 |
| TCGA_TCGA-AA-A01G | -665.516 | 1361.544 | 696.0283 | 0.760213 |
| TCGA_TCGA-AA-3870 | 2256.554 | 3817.913 | 6074.467 | 0.07408 |
| TCGA_TCGA-D5-6532 | -293.518 | 1266.663 | 973.1453 | 0.733164 |
| TCGA_TCGA-CM-6169 | 3192.704 | 4562.383 | 7755.087 | NA |
| TCGA_TCGA-D5-5541 | 1858.054 | 3313.144 | 5171.197 | 0.205268 |
| TCGA_TCGA-DM-A1DB | -1770.73 | 1826.067 | 55.34143 | 0.817862 |
| TCGA_TCGA-AA-A00A | 1054.446 | 3132.399 | 4186.845 | 0.344052 |
| TCGA_TCGA-D5-6530 | 827.3482 | 3915.285 | 4742.633 | 0.26639 |
| TCGA_TCGA-AA-3855 | 621.193 | 3359.105 | 3980.299 | 0.372357 |
| TCGA_TCGA-CM-5868 | 1083.209 | 1872.769 | 2955.978 | 0.507177 |
| TCGA_TCGA-AA-3519 | 559.795 | 2736.297 | 3296.092 | 0.463535 |
| TCGA_TCGA-AA-3526 | 1166.018 | 3425.375 | 4591.393 | 0.287721 |
| TCGA_TCGA-DM-A28M | -1989.54 | 878.4395 | -1111.1 | 0.903945 |
| TCGA_TCGA-D5-6926 | 2278.262 | 3216.131 | 5494.393 | 0.158623 |
| TCGA_TCGA-AA-3534 | 884.0273 | 2138.909 | 3022.936 | 0.498682 |
| TCGA_TCGA-G4-6317 | -1229.66 | 130.3563 | -1099.3 | 0.903203 |
| TCGA_TCGA-AA-A03J | 839.314 | 3240.556 | 4079.87 | 0.358753 |
| TCGA_TCGA-CM-6680 | 1870.483 | 3527.098 | 5397.581 | 0.172638 |
| TCGA_TCGA-AA-A01K | 1615.689 | 2977.028 | 4592.717 | 0.287534 |
| TCGA_TCGA-AA-3984 | 1280.793 | 3192.697 | 4473.491 | 0.304252 |
| TCGA_TCGA-D5-6536 | 1086.066 | 2644.344 | 3730.41 | 0.406142 |
| TCGA_TCGA-AD-A5EK | 152.8954 | 1144.637 | 1297.532 | 0.699964 |
| TCGA_TCGA-AA-3663 | 269.2995 | 2724.151 | 2993.45 | 0.502429 |
| TCGA_TCGA-AZ-6607 | 3108.511 | 3829.88 | 6938.391 | NA |
| TCGA_TCGA-AA-3520 | 1895.954 | 3187.413 | 5083.366 | 0.217868 |
| TCGA_TCGA-AA-3543 | 1315.518 | 4335.274 | 5650.792 | 0.135916 |
| TCGA_TCGA-AZ-5407 | -661.188 | 2852.006 | 2190.818 | 0.600577 |
| TCGA_TCGA-G4-6314 | 2254.802 | 2598.202 | 4853.004 | 0.25074 |
| TCGA_TCGA-CM-5348 | 2800.076 | 3743.12 | 6543.196 | 0.005344 |
| TCGA_TCGA-CK-6751 | 1475.692 | 2595.137 | 4070.828 | 0.359992 |
| TCGA_TCGA-D5-6929 | 1882.078 | 3074.71 | 4956.788 | 0.235964 |
| TCGA_TCGA-AA-3675 | 437.4809 | 2266.285 | 2703.766 | 0.538729 |
| TCGA_TCGA-A6-2681 | 2132.072 | 3245.789 | 5377.861 | 0.175488 |
| TCGA_TCGA-AA-3869 | 1413.154 | 3736.602 | 5149.756 | 0.208347 |
| TCGA_TCGA-DM-A282 | -22.5222 | 784.9735 | 762.4513 | 0.753842 |
| TCGA_TCGA-D5-7000 | 1405.795 | 3097.172 | 4502.967 | 0.300127 |
| TCGA_TCGA-AM-5821 | 1188.232 | 3967.362 | 5155.594 | 0.207509 |
| TCGA_TCGA-G4-6298 | 285.9235 | 348.5556 | 634.4791 | 0.766051 |
| TCGA_TCGA-AA-A01S | -1818.06 | 485.571 | -1332.49 | 0.917362 |
| TCGA_TCGA-NH-A8F8 | 1400.605 | 2382.046 | 3782.651 | 0.399123 |
| TCGA_TCGA-AA-3966 | 2323.455 | 4847.841 | 7171.295 | NA |
| TCGA_TCGA-AA-3516 | 726.7988 | 3408.738 | 4135.537 | 0.351114 |
| TCGA_TCGA-A6-2676 | 1513.494 | 4342.895 | 5856.39 | 0.10596 |
| TCGA_TCGA-AA-3846 | 757.4202 | 3165.542 | 3922.962 | 0.380155 |
| TCGA_TCGA-AA-3544 | 2076.651 | 4402.487 | 6479.138 | 0.014746 |
| TCGA_TCGA-AA-3956 | 1292.205 | 3015.598 | 4307.804 | 0.327327 |
| TCGA_TCGA-AA-3532 | 1896.871 | 4155.802 | 6052.673 | 0.07727 |
| TCGA_TCGA-AA-3955 | 96.08225 | 2098.334 | 2194.416 | 0.600155 |
| TCGA_TCGA-CK-5912 | 706.2732 | 1796.544 | 2502.817 | 0.563342 |
| TCGA_TCGA-AD-6895 | 1291.211 | 3388.895 | 4680.106 | 0.275225 |
| TCGA_TCGA-AA-3556 | 707.4427 | 2908.865 | 3616.308 | 0.42139 |
| TCGA_TCGA-CM-6678 | 291.5247 | 1217.439 | 1508.964 | 0.677465 |
| TCGA_TCGA-AA-3851 | 1062.932 | 3514.757 | 4577.689 | 0.289647 |
| TCGA_TCGA-CM-5860 | 2439.617 | 3113.356 | 5552.974 | 0.150127 |
| TCGA_TCGA-A6-5661 | 1207.135 | 3339.967 | 4547.103 | 0.293941 |
| TCGA_TCGA-AA-A02F | -22.6636 | 455.9992 | 433.3356 | 0.784693 |
| TCGA_TCGA-AA-3861 | -779.755 | 3319.738 | 2539.983 | 0.558826 |
| TCGA_TCGA-DM-A1DA | -958.121 | 1173.849 | 215.7281 | 0.80409 |
| TCGA_TCGA-CM-6162 | 3575.24 | 4716.733 | 8291.973 | NA |
| TCGA_TCGA-AA-3522 | -64.2877 | 2817.442 | 2753.155 | 0.532607 |
| TCGA_TCGA-AA-A00U | -57.6798 | 1908.658 | 1850.978 | 0.639699 |
| TCGA_TCGA-F4-6807 | 3095.238 | 4296.422 | 7391.66 | NA |
| TCGA_TCGA-AA-3930 | 1282.462 | 3550.498 | 4832.96 | 0.253587 |
| TCGA_TCGA-AZ-6599 | -2351.29 | 760.3145 | -1590.97 | 0.931802 |
| TCGA_TCGA-AA-3947 | 1792.963 | 3202.544 | 4995.507 | 0.230437 |
| TCGA_TCGA-AA-3841 | 1187.161 | 3480.465 | 4667.626 | 0.276986 |
| TCGA_TCGA-CM-6171 | 467.4716 | 2834.482 | 3301.954 | 0.462773 |
| TCGA_TCGA-CM-4743 | 254.3763 | 3016.064 | 3270.44 | 0.466869 |
| TCGA_TCGA-AA-3814 | 2130.017 | 3968.548 | 6098.564 | 0.070552 |
| TCGA_TCGA-NH-A6GA | 174.9279 | 2010.138 | 2185.066 | 0.601252 |
| TCGA_TCGA-A6-2675 | 2566.221 | 3702.722 | 6268.943 | 0.045585 |
| TCGA_TCGA-G4-6297 | 2336.253 | 3358.793 | 5695.046 | 0.129478 |
| TCGA_TCGA-A6-5656 | 1337.534 | 1985.612 | 3323.147 | 0.460013 |
| TCGA_TCGA-DM-A0X9 | -103.924 | 2311.365 | 2207.441 | 0.598624 |
| TCGA_TCGA-AA-3553 | 1489.392 | 3652.724 | 5142.116 | 0.209444 |
| TCGA_TCGA-DM-A0XD | 193.1659 | 1526.046 | 1719.212 | 0.654445 |
| TCGA_TCGA-AA-3511 | 1687.454 | 2383.587 | 4071.04 | 0.359962 |
| TCGA_TCGA-A6-A56B | 973.0847 | 1335.443 | 2308.528 | 0.586673 |
| TCGA_TCGA-A6-5667 | 1126.461 | 1841.519 | 2967.98 | 0.505658 |
| TCGA_TCGA-AA-3866 | 2402.175 | 4523.557 | 6925.732 | NA |
| TCGA_TCGA-AA-A017 | 780.727 | 2315.393 | 3096.12 | 0.489342 |
| TCGA_TCGA-CM-6674 | 1542.652 | 3203.388 | 4746.039 | 0.265908 |
| TCGA_TCGA-DM-A1D8 | -831.452 | 821.3428 | -10.1093 | 0.823353 |
| TCGA_TCGA-CM-6170 | 1549.019 | 2970.776 | 4519.795 | 0.29777 |
| TCGA_TCGA-AZ-4616 | 593.2943 | 2766.679 | 3359.974 | 0.455206 |
| TCGA_TCGA-AA-3994 | 992.3296 | 2718.018 | 3710.347 | 0.408831 |
| TCGA_TCGA-AA-3877 | 1920.689 | 4052.815 | 5973.504 | 0.088851 |
| TCGA_TCGA-WS-AB45 | 4173.599 | 5298.678 | 9472.278 | NA |
| TCGA_TCGA-AA-A00L | -527.085 | 1208.661 | 681.5755 | 0.761589 |
| TCGA_TCGA-CM-5864 | 74.44321 | 2286.375 | 2360.818 | 0.58044 |
| TCGA_TCGA-CA-5254 | 951.6227 | 1867.475 | 2819.098 | 0.52439 |
| TCGA_TCGA-D5-6898 | 1985.3 | 3263.295 | 5248.595 | 0.194136 |
| TCGA_TCGA-AA-3696 | 404.0412 | 1416.41 | 1820.451 | 0.643137 |
| TCGA_TCGA-DM-A280 | 209.8142 | 1832.088 | 2041.902 | 0.61791 |
| TCGA_TCGA-AA-A00O | 1745.459 | 2565.395 | 4310.854 | 0.326904 |
| TCGA_TCGA-QG-A5YW | 750.1095 | 3171.409 | 3921.518 | 0.380351 |
| TCGA_TCGA-AD-6965 | 397.5032 | 1690.867 | 2088.371 | 0.612533 |
| TCGA_TCGA-CK-4948 | 1504.864 | 2797.137 | 4302.001 | 0.328132 |
| TCGA_TCGA-DM-A1HA | -2411.12 | 826.7402 | -1584.38 | 0.93145 |
| TCGA_TCGA-5M-AAT5 | -1139.41 | 823.8307 | -315.576 | 0.847964 |
| TCGA_TCGA-F4-6809 | 2204.225 | 3605.262 | 5809.487 | 0.112803 |
| TCGA_TCGA-CM-4748 | 1191.41 | 2631.055 | 3822.465 | 0.393757 |
| TCGA_TCGA-AA-3858 | 1164.996 | 2755.471 | 3920.467 | 0.380494 |
| TCGA_TCGA-D5-6531 | 1647.841 | 3296.706 | 4944.547 | 0.237709 |
| TCGA_TCGA-AA-3662 | 1330.35 | 3538.403 | 4868.753 | 0.248501 |
| TCGA_TCGA-AM-5820 | 980.8505 | 1773.92 | 2754.771 | 0.532406 |
| TCGA_TCGA-AA-A02K | -714.314 | 1260.78 | 546.4664 | 0.774291 |
| TCGA_TCGA-G4-6310 | 567.3417 | 342.7081 | 910.0498 | 0.739431 |
| TCGA_TCGA-AD-6889 | -148.464 | 2001.844 | 1853.381 | 0.639428 |
| TCGA_TCGA-AA-3872 | 2824.076 | 3645.571 | 6469.647 | 0.016139 |
| TCGA_TCGA-AA-A01R | 525.6744 | 4200.959 | 4726.633 | 0.268653 |
| TCGA_TCGA-AA-3975 | 1118.94 | 2988.941 | 4107.88 | 0.354912 |
| TCGA_TCGA-5M-AAT6 | 2317.222 | 4397.217 | 6714.439 | NA |
| TCGA_TCGA-D5-6931 | 1430.365 | 3448.87 | 4879.235 | 0.247011 |
| TCGA_TCGA-D5-6924 | 2565.603 | 3900.785 | 6466.388 | 0.016618 |
| TCGA_TCGA-AA-3860 | 1801.216 | 3699.042 | 5500.258 | 0.157773 |
| TCGA_TCGA-A6-5665 | 397.7832 | 2806.996 | 3204.779 | 0.47537 |
| TCGA_TCGA-5M-AATA | 722.0074 | 2528.475 | 3250.482 | 0.469457 |
| TCGA_TCGA-D5-6540 | 500.1612 | 2863.982 | 3364.144 | 0.454661 |
| TCGA_TCGA-CA-6718 | 1573.506 | 4416.19 | 5989.696 | 0.086483 |
| TCGA_TCGA-AA-3986 | 1116.471 | 4494.548 | 5611.019 | 0.141698 |
| TCGA_TCGA-G4-6323 | -1287.03 | 3403.868 | 2116.835 | 0.609225 |
| TCGA_TCGA-A6-A566 | 3848.564 | 4710.896 | 8559.46 | NA |
| TCGA_TCGA-AZ-6608 | -2444.58 | 700.4422 | -1744.13 | 0.939726 |
| TCGA_TCGA-AA-3712 | 1446.777 | 2473.154 | 3919.931 | 0.380566 |
| TCGA_TCGA-A6-5662 | 681.8407 | 1321.609 | 2003.449 | 0.622338 |
| TCGA_TCGA-AA-3831 | 912.0697 | 3261.168 | 4173.238 | 0.345927 |
| TCGA_TCGA-AA-A02H | 306.6763 | 1806.497 | 2113.173 | 0.609651 |
| TCGA_TCGA-CM-6676 | 962.135 | 1393.719 | 2355.854 | 0.581033 |
| TCGA_TCGA-AD-6899 | 2510.625 | 4024.94 | 6535.565 | 0.006464 |
| TCGA_TCGA-AA-3685 | 1423.659 | 3971.889 | 5395.548 | 0.172932 |
| TCGA_TCGA-CK-5915 | -725.359 | 450.6736 | -274.685 | 0.844767 |
| TCGA_TCGA-AA-3548 | 933.3023 | 3200.1 | 4133.402 | 0.351407 |
| TCGA_TCGA-F4-6569 | 3390.453 | 3957.79 | 7348.243 | NA |
| TCGA_TCGA-F4-6461 | 1797.114 | 3078.412 | 4875.527 | 0.247538 |
| TCGA_TCGA-F4-6854 | 1505.143 | 2691.685 | 4196.828 | 0.342676 |
| TCGA_TCGA-F4-6856 | 368.4283 | 2376.601 | 2745.029 | 0.533616 |
| TCGA_TCGA-AY-A54L | -2530.85 | 364.5619 | -2166.29 | 0.959097 |
| TCGA_TCGA-AA-3538 | 1308.695 | 2435.854 | 3744.549 | 0.404245 |
| TCGA_TCGA-AA-3842 | 1689.016 | 2776.626 | 4465.642 | 0.305349 |
| TCGA_TCGA-AZ-4308 | 1640.426 | 2770.541 | 4410.967 | 0.312981 |
| TCGA_TCGA-CK-6746 | -17.7898 | 3817.905 | 3800.115 | 0.396771 |
| TCGA_TCGA-AA-3509 | 978.5283 | 3006.326 | 3984.855 | 0.371736 |
| TCGA_TCGA-AA-3680 | 287.1626 | 3029.347 | 3316.509 | 0.460878 |
| TCGA_TCGA-AA-3542 | -20.5436 | 1942.727 | 1922.183 | 0.631631 |
| TCGA_TCGA-AD-6964 | 2961.864 | 4982.008 | 7943.872 | NA |
| TCGA_TCGA-A6-6650 | -146.828 | 1346.89 | 1200.062 | 0.71011 |
| TCGA_TCGA-A6-6140 | -517.366 | 2261.022 | 1743.656 | 0.651728 |
| TCGA_TCGA-AA-A024 | -899.315 | 1671.815 | 772.5003 | 0.752872 |
| TCGA_TCGA-A6-2682 | 1729.799 | 3236.338 | 4966.137 | 0.23463 |
| TCGA_TCGA-CM-6675 | -14.7863 | 1569.793 | 1555.007 | 0.672478 |
| TCGA_TCGA-DM-A1D7 | -83.1862 | 1777.889 | 1694.702 | 0.657161 |
| TCGA_TCGA-4T-AA8H | -2226.28 | 679.892 | -1546.38 | 0.929406 |
| TCGA_TCGA-A6-6653 | 818.3128 | 2756.581 | 3574.894 | 0.426895 |
| TCGA_TCGA-AA-3666 | 632.9244 | 3228.353 | 3861.278 | 0.388514 |
| TCGA_TCGA-F4-6460 | 1836.321 | 2865.712 | 4702.033 | 0.272129 |
| TCGA_TCGA-AA-3655 | 1134.427 | 2625.581 | 3760.007 | 0.402168 |
| TCGA_TCGA-AA-3673 | 845.4009 | 2607.861 | 3453.262 | 0.442972 |
| TCGA_TCGA-AA-3952 | 1568.019 | 2567.057 | 4135.076 | 0.351177 |
| TCGA_TCGA-T9-A92H | -985.485 | 1168.484 | 182.9985 | 0.806937 |
| TCGA_TCGA-AY-4070 | 750.6391 | 2773.504 | 3524.143 | 0.43362 |
| TCGA_TCGA-AY-A8YK | -326.91 | 1747.414 | 1420.504 | 0.686959 |
| TCGA_TCGA-CK-4950 | 1003.805 | 3759.457 | 4763.262 | 0.26347 |
| TCGA_TCGA-F4-6570 | 2417.554 | 4601.802 | 7019.356 | NA |
| TCGA_TCGA-AZ-6600 | 2107.906 | 3399.469 | 5507.375 | 0.156741 |
| TCGA_TCGA-AA-A00J | 1191.432 | 2606.363 | 3797.795 | 0.397084 |
| TCGA_TCGA-AA-3667 | 463.4947 | 2926.405 | 3389.899 | 0.451291 |
| TCGA_TCGA-AA-3554 | 2290.405 | 4521.426 | 6811.831 | NA |
| TCGA_TCGA-AZ-6606 | -1050.24 | 1563.577 | 513.3412 | 0.777359 |
| TCGA_TCGA-AA-3852 | 1628.042 | 3403.808 | 5031.85 | 0.225243 |
| TCGA_TCGA-D5-6922 | 2156.725 | 2661.371 | 4818.096 | 0.255697 |
| TCGA_TCGA-A6-6137 | 420.6082 | 3294.494 | 3715.102 | 0.408194 |
| TCGA_TCGA-AA-3495 | 630.1224 | 3151.877 | 3782 | 0.39921 |
| TCGA_TCGA-AA-3489 | 3565.905 | 5014.529 | 8580.435 | NA |
| TCGA_TCGA-G4-6322 | 204.5799 | 2191.05 | 2395.63 | 0.576271 |
| TCGA_TCGA-SS-A7HO | -924.445 | 459.0304 | -465.414 | 0.859416 |
| TCGA_TCGA-AA-3976 | 1001.449 | 2441.132 | 3442.581 | 0.444377 |
| TCGA_TCGA-AA-3811 | 1064.523 | 3212.61 | 4277.134 | 0.331578 |
| TCGA_TCGA-AD-A5EJ | 316.6013 | 2076.53 | 2393.131 | 0.576571 |
| TCGA_TCGA-AA-3819 | 249.4925 | 2293.317 | 2542.81 | 0.558482 |
| TCGA_TCGA-AA-A02Y | -2278.33 | 2367.158 | 88.82392 | 0.815024 |
| TCGA_TCGA-AZ-6603 | 1813.128 | 3202.338 | 5015.466 | 0.227585 |
| TCGA_TCGA-AA-A022 | 1076.969 | 3958.578 | 5035.547 | 0.224714 |
| TCGA_TCGA-G4-6626 | -1096.18 | 980.0631 | -116.117 | 0.832084 |
| TCGA_TCGA-AA-3875 | 1188.686 | 3642.64 | 4831.326 | 0.253819 |
| TCGA_TCGA-G4-6306 | -2071.66 | 1856.464 | -215.2 | 0.840062 |
| TCGA_TCGA-G4-6320 | -1254.39 | 1645.911 | 391.5189 | 0.788483 |
| TCGA_TCGA-CK-6748 | 2454.139 | 2955.624 | 5409.762 | 0.170876 |
| TCGA_TCGA-AA-3864 | 1211.068 | 2626.841 | 3837.91 | 0.391672 |
| TCGA_TCGA-A6-6142 | 2553.148 | 2885.26 | 5438.408 | 0.166732 |
| TCGA_TCGA-A6-2678 | 1180.03 | 3078.655 | 4258.686 | 0.334131 |
| TCGA_TCGA-CA-5796 | -293.753 | 2735.06 | 2441.307 | 0.570778 |
| TCGA_TCGA-A6-A567 | 321.7737 | 1195.08 | 1516.854 | 0.676613 |
| TCGA_TCGA-AA-3713 | 780.9171 | 3788.799 | 4569.717 | 0.290766 |
| TCGA_TCGA-A6-6648 | -344.421 | 1715.448 | 1371.027 | 0.692218 |
| TCGA_TCGA-A6-A565 | 2070.449 | 4740.829 | 6811.278 | NA |
| TCGA_TCGA-A6-5660 | 1296.021 | 2434.957 | 3730.977 | 0.406066 |
| TCGA_TCGA-AZ-6601 | 1996.317 | 4288.682 | 6284.999 | 0.043231 |
| TCGA_TCGA-5M-AATE | -214.513 | 1478.712 | 1264.199 | 0.70345 |
| TCGA_TCGA-CM-6161 | 1294.516 | 3343.251 | 4637.767 | 0.281195 |
| TCGA_TCGA-AA-A01Q | -219.934 | 3063.349 | 2843.415 | 0.521347 |
| TCGA_TCGA-AZ-4313 | -306.184 | 1599.377 | 1293.194 | 0.700418 |
| TCGA_TCGA-A6-6652 | -731.668 | 799.7453 | 68.07693 | 0.816785 |
| TCGA_TCGA-AA-A00E | 937.1495 | 3815.868 | 4753.018 | 0.26492 |
| TCGA_TCGA-F4-6808 | -697.247 | 1133.605 | 436.3581 | 0.784418 |
| TCGA_TCGA-AA-A02W | 11.29421 | 1476.159 | 1487.453 | 0.679784 |
| TCGA_TCGA-DM-A28C | -1165.78 | 428.2155 | -737.56 | 0.879149 |
| TCGA_TCGA-D5-6927 | 1878.918 | 4093.831 | 5972.748 | 0.088961 |
| TCGA_TCGA-AA-3980 | 1025.973 | 3906.046 | 4932.019 | 0.239495 |
| TCGA_TCGA-AA-3681 | 686.1797 | 3596.145 | 4282.325 | 0.330859 |
| TCGA_TCGA-AA-3492 | 1064.853 | 3118.115 | 4182.968 | 0.344586 |
| TCGA_TCGA-A6-2671 | 2064.012 | 2896.002 | 4960.013 | 0.235504 |
| TCGA_TCGA-D5-6535 | 1014.36 | 3224.91 | 4239.27 | 0.336816 |
| TCGA_TCGA-AA-A029 | -1040.3 | 1429.489 | 389.1877 | 0.788694 |
| TCGA_TCGA-AD-6963 | 65.45473 | 3274.096 | 3339.55 | 0.457874 |
| TCGA_TCGA-CM-4747 | 837.749 | 2134.926 | 2972.675 | 0.505063 |
| TCGA_TCGA-NH-A50T | -791.117 | 513.1623 | -277.954 | 0.845024 |
| TCGA_TCGA-CM-6168 | 3036.63 | 4060.216 | 7096.845 | NA |
| TCGA_TCGA-AA-3518 | 504.0874 | 3602.909 | 4106.996 | 0.355033 |
| TCGA_TCGA-AA-A01T | -879.07 | 1550.271 | 671.2018 | 0.762575 |
| TCGA_TCGA-RU-A8FL | -2971.53 | 77.5076 | -2894.02 | 0.983812 |
| TCGA_TCGA-A6-6651 | 3559.367 | 4679.781 | 8239.147 | NA |
| TCGA_TCGA-AA-3850 | 1431.253 | 3924.904 | 5356.157 | 0.178624 |
| TCGA_TCGA-AA-3972 | 668.5617 | 1932.515 | 2601.076 | 0.551367 |
| TCGA_TCGA-AA-3812 | 1921.797 | 3300.514 | 5222.311 | 0.197919 |
| TCGA_TCGA-AY-A69D | -83.5236 | 1733.608 | 1650.084 | 0.662084 |
| TCGA_TCGA-AA-A01Z | -782.231 | 729.2577 | -52.9732 | 0.826907 |
| TCGA_TCGA-A6-3810 | 2557.7 | 3429.918 | 5987.619 | 0.086787 |
| TCGA_TCGA-DM-A28H | -674.1 | 633.1017 | -40.9979 | 0.825917 |
| TCGA_TCGA-G4-6586 | -1090.35 | 2965.002 | 1874.653 | 0.637024 |
| TCGA_TCGA-D5-6928 | 3227.131 | 6079.087 | 9306.217 | NA |
| TCGA_TCGA-G4-6315 | -1362.78 | 901.2277 | -461.551 | 0.859126 |
| TCGA_TCGA-D5-6534 | 3821.028 | 5315.346 | 9136.374 | NA |
| TCGA_TCGA-AA-3664 | -244.321 | 2338.425 | 2094.104 | 0.611867 |
| TCGA_TCGA-G4-6303 | 1522.28 | 2395.456 | 3917.735 | 0.380864 |
| TCGA_TCGA-AA-3692 | 1227.38 | 3554.437 | 4781.817 | 0.260842 |
| TCGA_TCGA-AA-3968 | 1721.743 | 3171.924 | 4893.667 | 0.244957 |
| TCGA_TCGA-F4-6805 | 2754.67 | 3823.885 | 6578.554 | 0.000154 |
| TCGA_TCGA-AA-3560 | 1149.123 | 3172.234 | 4321.357 | 0.325447 |
| TCGA_TCGA-D5-5537 | 921.128 | 1784.334 | 2705.462 | 0.538519 |
| TCGA_TCGA-CM-5862 | 857.4357 | 905.1589 | 1762.595 | 0.649617 |
| TCGA_TCGA-D5-6932 | 1478.42 | 2774.313 | 4252.733 | 0.334955 |
| TCGA_TCGA-AA-3982 | 1636.505 | 3907.615 | 5544.121 | 0.151412 |
| TCGA_TCGA-DM-A28A | 699.0388 | 1438.763 | 2137.801 | 0.606781 |
| TCGA_TCGA-CM-6172 | 1102.785 | 2272.289 | 3375.074 | 0.453232 |
| TCGA_TCGA-D5-6930 | 2016.206 | 3824.513 | 5840.719 | 0.108247 |
| TCGA_TCGA-D5-6529 | 2498.745 | 4432.323 | 6931.068 | NA |
| TCGA_TCGA-A6-2683 | -391.697 | 1578.264 | 1186.567 | 0.711503 |
| TCGA_TCGA-CK-5913 | 1256.162 | 3320.468 | 4576.63 | 0.289795 |
| TCGA_TCGA-AA-3977 | 1228.862 | 3453.153 | 4682.016 | 0.274956 |
| TCGA_TCGA-AA-3496 | 2226.806 | 4027.348 | 6254.154 | 0.047754 |
| TCGA_TCGA-D5-5540 | 197.8757 | 1987.642 | 2185.518 | 0.601199 |
| TCGA_TCGA-AD-6901 | 2611.13 | 3639.496 | 6250.626 | 0.048271 |
| TCGA_TCGA-AA-A01F | -1827.63 | 1161.61 | -666.017 | 0.874097 |
| TCGA_TCGA-AA-3552 | 952.7602 | 3942.985 | 4895.745 | 0.244662 |
| TCGA_TCGA-F4-6855 | 2793.671 | 2816.852 | 5610.524 | 0.14177 |
| TCGA_TCGA-AD-5900 | 1660.467 | 3726.627 | 5387.094 | 0.174154 |
| TCGA_TCGA-AA-3979 | -588.735 | 1425.326 | 836.5917 | 0.746647 |
| TCGA_TCGA-AA-A01I | -341.85 | 2200.711 | 1858.861 | 0.63881 |
| TCGA_TCGA-AA-A03F | -580.386 | 2740.064 | 2159.677 | 0.604226 |
| TCGA_TCGA-3L-AA1B | 885.0309 | 2603.231 | 3488.262 | 0.43836 |
| TCGA_TCGA-AA-3693 | 103.3909 | 1970.322 | 2073.713 | 0.614232 |
| TCGA_TCGA-AA-3502 | -908.231 | 2892.874 | 1984.642 | 0.624497 |
| TCGA_TCGA-AA-A02E | -348.588 | 1891.812 | 1543.224 | 0.673757 |
| TCGA_TCGA-CM-4746 | -953.093 | 1772.114 | 819.0209 | 0.74836 |
| TCGA_TCGA-G4-6295 | 587.3834 | 3617.288 | 4204.672 | 0.341594 |
| TCGA_TCGA-AA-A01C | 1278.057 | 2150.4 | 3428.457 | 0.446233 |
| TCGA_TCGA-G4-6588 | 647.4288 | 2753.027 | 3400.456 | 0.449907 |
| TCGA_TCGA-A6-2680 | 994.5249 | 2906.353 | 3900.878 | 0.383151 |
| TCGA_TCGA-QG-A5YV | -575.008 | 1780.684 | 1205.676 | 0.709529 |
| TCGA_TCGA-AA-3561 | 177.7414 | 2238.771 | 2416.512 | 0.573763 |
| TCGA_TCGA-AA-3525 | 59.60106 | 2937.938 | 2997.539 | 0.50191 |
| TCGA_TCGA-AA-3531 | -254.976 | 1641.627 | 1386.651 | 0.690561 |
| TCGA_TCGA-AA-3973 | 626.5191 | 1658.122 | 2284.641 | 0.589509 |
| TCGA_TCGA-AA-3527 | 1680.288 | 3250.041 | 4930.329 | 0.239736 |
| TCGA_TCGA-AA-3555 | 1346.546 | 2635.009 | 3981.555 | 0.372186 |
| TCGA_TCGA-AG-A025 | 456.3201 | 1902.393 | 2358.713 | 0.580691 |
| TCGA_TCGA-EI-6884 | 1775.404 | 3578.455 | 5353.859 | 0.178956 |
| TCGA_TCGA-AG-3582 | 1239.333 | 2891.807 | 4131.14 | 0.351718 |
| TCGA_TCGA-DC-6154 | 430.9653 | 1907.971 | 2338.936 | 0.583052 |
| TCGA_TCGA-AG-3611 | -323.544 | 2871.406 | 2547.862 | 0.557866 |
| TCGA_TCGA-DY-A1DD | -271.31 | 843.7467 | 572.4364 | 0.771873 |
| TCGA_TCGA-AG-3609 | 2035.696 | 3337.303 | 5372.999 | 0.176191 |
| TCGA_TCGA-AG-A01L | -561.915 | 1510.733 | 948.8181 | 0.735588 |
| TCGA_TCGA-AG-4022 | 2095.124 | 3602.561 | 5697.684 | 0.129094 |
| TCGA_TCGA-AG-A01W | -271.009 | 2328.71 | 2057.7 | 0.616085 |
| TCGA_TCGA-AG-A02N | -799.262 | 2666.402 | 1867.141 | 0.637874 |
| TCGA_TCGA-AH-6549 | 1568.438 | 2256.484 | 3824.922 | 0.393426 |
| TCGA_TCGA-AH-6903 | -830.079 | 1481.889 | 651.8097 | 0.764413 |
| TCGA_TCGA-AG-3727 | 1052.495 | 2342.856 | 3395.35 | 0.450577 |
| TCGA_TCGA-F5-6863 | 226.4026 | 659.8756 | 886.2782 | 0.741775 |
| TCGA_TCGA-AG-3887 | 638.5283 | 2884.971 | 3523.5 | 0.433705 |
| TCGA_TCGA-AG-A01J | -753.541 | 1806.238 | 1052.697 | 0.725173 |
| TCGA_TCGA-AF-5654 | -483.82 | 1155.831 | 672.0105 | 0.762498 |
| TCGA_TCGA-DY-A1DE | 960.7467 | 2536.697 | 3497.443 | 0.437148 |
| TCGA_TCGA-AF-3913 | 1422.394 | 2014.576 | 3436.97 | 0.445114 |
| TCGA_TCGA-AG-A014 | -363.777 | 1235.336 | 871.5597 | 0.743223 |
| TCGA_TCGA-AG-4015 | 222.1797 | 2091.335 | 2313.515 | 0.58608 |
| TCGA_TCGA-G5-6572 | 1684.676 | 2637.997 | 4322.673 | 0.325264 |
| TCGA_TCGA-AG-A011 | 535.1098 | 2202.383 | 2737.492 | 0.534552 |
| TCGA_TCGA-EI-6511 | 2207.7 | 4583.36 | 6791.06 | NA |
| TCGA_TCGA-AG-4008 | 2020.998 | 2938.955 | 4959.953 | 0.235512 |
| TCGA_TCGA-AG-3731 | 2906.458 | 4322.208 | 7228.666 | NA |
| TCGA_TCGA-AG-A032 | -24.6487 | 1962.326 | 1937.677 | 0.629866 |
| TCGA_TCGA-AH-6544 | -1631.55 | 358.1044 | -1273.44 | 0.913878 |
| TCGA_TCGA-AG-A020 | -1579.39 | 1000.469 | -578.922 | 0.867815 |
| TCGA_TCGA-AG-A026 | 238.3269 | 864.0405 | 1102.367 | 0.720133 |
| TCGA_TCGA-DY-A1H8 | -2349.25 | 766.0475 | -1583.21 | 0.931387 |
| TCGA_TCGA-AG-3883 | 2466.322 | 3762.565 | 6228.887 | 0.051458 |
| TCGA_TCGA-EI-7004 | 3877.385 | 4003.307 | 7880.692 | NA |
| TCGA_TCGA-AF-6136 | 365.742 | 2173.751 | 2539.493 | 0.558885 |
| TCGA_TCGA-DC-4745 | 947.2591 | 2426.11 | 3373.37 | 0.453455 |
| TCGA_TCGA-EI-6883 | 278.7865 | 2532.428 | 2811.215 | 0.525375 |
| TCGA_TCGA-EI-6917 | 2431.175 | 4411.135 | 6842.31 | NA |
| TCGA_TCGA-F5-6861 | 406.6331 | 1832.697 | 2239.33 | 0.594868 |
| TCGA_TCGA-EI-6508 | 508.6755 | 1725.091 | 2233.766 | 0.595524 |
| TCGA_TCGA-F5-6465 | 2825.739 | 4136.934 | 6962.672 | NA |
| TCGA_TCGA-AF-6672 | -122.629 | 2028.197 | 1905.568 | 0.63352 |
| TCGA_TCGA-AG-3885 | 511.4897 | 3046.82 | 3558.309 | 0.429095 |
| TCGA_TCGA-EI-6509 | 666.6961 | 1245.447 | 1912.143 | 0.632773 |
| TCGA_TCGA-DC-5337 | 458.022 | 2906.943 | 3364.965 | 0.454554 |
| TCGA_TCGA-AG-3893 | 1655.238 | 2719.871 | 4375.109 | 0.317976 |
| TCGA_TCGA-AG-A02G | -1114.96 | 1663.407 | 548.4512 | 0.774107 |
| TCGA_TCGA-AG-3901 | 3165.343 | 4434.386 | 7599.73 | NA |
| TCGA_TCGA-AG-3726 | 757.0143 | 2486.986 | 3244 | 0.470297 |
| TCGA_TCGA-AG-3896 | 1350.395 | 2840.203 | 4190.598 | 0.343535 |
| TCGA_TCGA-DC-6160 | 134.5503 | 3140.759 | 3275.309 | 0.466236 |
| TCGA_TCGA-AG-3581 | 1026.459 | 3070.537 | 4096.996 | 0.356405 |
| TCGA_TCGA-G5-6641 | -2546.09 | 202.1857 | -2343.9 | 0.966151 |
| TCGA_TCGA-F5-6812 | 2514.923 | 3574.422 | 6089.345 | 0.071902 |
| TCGA_TCGA-AG-4001 | 2417.382 | 3247.19 | 5664.572 | 0.133912 |
| TCGA_TCGA-AF-2687 | 3291.448 | 4211.344 | 7502.792 | NA |
| TCGA_TCGA-AH-6644 | 2236.434 | 2968.907 | 5205.341 | 0.20036 |
| TCGA_TCGA-AG-3586 | 405.1782 | 2680.804 | 3085.982 | 0.490639 |
| TCGA_TCGA-AG-3909 | 844.9616 | 2695.838 | 3540.8 | 0.431415 |
| TCGA_TCGA-AG-3878 | 2337.043 | 4067.926 | 6404.969 | 0.025631 |
| TCGA_TCGA-AG-3592 | 891.5194 | 2671.703 | 3563.222 | 0.428444 |
| TCGA_TCGA-F5-6814 | 1185.542 | 3070.641 | 4256.183 | 0.334478 |
| TCGA_TCGA-AG-3605 | -461.117 | 2741.003 | 2279.887 | 0.590072 |
| TCGA_TCGA-EI-6513 | 771.7123 | 2244.396 | 3016.108 | 0.49955 |
| TCGA_TCGA-DC-6158 | 2829.741 | 3791.496 | 6621.237 | NA |
| TCGA_TCGA-EI-6512 | 459.0784 | 2585.287 | 3044.366 | 0.495953 |
| TCGA_TCGA-AG-3601 | 795.3596 | 1880.513 | 2675.872 | 0.542174 |
| TCGA_TCGA-CI-6622 | -145.523 | 1423.871 | 1278.348 | 0.701972 |
| TCGA_TCGA-BM-6198 | 1851.652 | 3948.018 | 5799.671 | 0.114235 |
| TCGA_TCGA-AG-3881 | 2620.775 | 4042.402 | 6663.177 | NA |
| TCGA_TCGA-DY-A1DC | 1002.012 | 2175.7 | 3177.711 | 0.478862 |
| TCGA_TCGA-AG-3612 | 1720.782 | 3120.522 | 4841.304 | 0.252402 |
| TCGA_TCGA-AG-3600 | 887.4259 | 3140.328 | 4027.754 | 0.365883 |
| TCGA_TCGA-AG-3599 | 169.9977 | 3061.699 | 3231.697 | 0.47189 |
| TCGA_TCGA-AG-3584 | 1348.83 | 2984.903 | 4333.733 | 0.323728 |
| TCGA_TCGA-DC-6156 | 3130.952 | 4487.289 | 7618.241 | NA |
| TCGA_TCGA-DT-5265 | 2248.445 | 2999.126 | 5247.572 | 0.194283 |
| TCGA_TCGA-AG-3902 | 676.3596 | 4212.628 | 4888.987 | 0.245623 |
| TCGA_TCGA-AG-A036 | 831.9229 | 2250.488 | 3082.411 | 0.491096 |
| TCGA_TCGA-AG-3593 | 680.7825 | 3562.117 | 4242.9 | 0.336315 |
| TCGA_TCGA-AG-A01Y | 960.5941 | 2884.842 | 3845.436 | 0.390656 |
| TCGA_TCGA-AG-3580 | 175.4924 | 3214.842 | 3390.335 | 0.451234 |
| TCGA_TCGA-AG-3742 | 1176.282 | 1847.141 | 3023.423 | 0.49862 |
| TCGA_TCGA-AG-A002 | -842.032 | 469.7939 | -372.239 | 0.852343 |
| TCGA_TCGA-DC-6683 | 1082.093 | 2020.517 | 3102.611 | 0.488511 |
| TCGA_TCGA-AF-6655 | 1951.154 | 2425.767 | 4376.921 | 0.317724 |
| TCGA_TCGA-AG-A023 | 1319.862 | 2632.113 | 3951.975 | 0.376212 |
| TCGA_TCGA-AG-3728 | 2176.096 | 4154.942 | 6331.038 | 0.036478 |
| TCGA_TCGA-AF-3400 | 3324.79 | 5188.191 | 8512.982 | NA |
| TCGA_TCGA-AF-2691 | 1457.993 | 3583.675 | 5041.668 | 0.223838 |
| TCGA_TCGA-CL-5918 | -917.033 | 1894.909 | 977.8767 | 0.732691 |
| TCGA_TCGA-F5-6864 | 2435.162 | 3121.335 | 5556.497 | 0.149616 |
| TCGA_TCGA-AF-A56K | 2049.775 | 3204.659 | 5254.434 | 0.193295 |
| TCGA_TCGA-AG-4021 | 1870.206 | 2260.458 | 4130.664 | 0.351783 |
| TCGA_TCGA-AG-A01N | -271.078 | 962.095 | 691.0171 | 0.76069 |
| TCGA_TCGA-DC-5869 | 1269.985 | 2136.43 | 3406.415 | 0.449126 |
| TCGA_TCGA-AG-3608 | 863.3602 | 3559.198 | 4422.558 | 0.311365 |
| TCGA_TCGA-DY-A0XA | 120.3552 | 1528.971 | 1649.326 | 0.662167 |
| TCGA_TCGA-AG-3594 | 782.2695 | 4422.354 | 5204.623 | 0.200463 |
| TCGA_TCGA-EF-5831 | 1452.446 | 2381.153 | 3833.599 | 0.392255 |
| TCGA_TCGA-AG-3725 | -495.849 | 2107.466 | 1611.616 | 0.666305 |
| TCGA_TCGA-EI-6514 | 1462.277 | 2241.152 | 3703.429 | 0.409758 |
| TCGA_TCGA-CI-6624 | 2034.778 | 3544.129 | 5578.907 | 0.146363 |
| TCGA_TCGA-CI-6620 | 1082.116 | 2236.515 | 3318.631 | 0.460601 |
| TCGA_TCGA-CI-6621 | 1777.885 | 3555.411 | 5333.296 | 0.181924 |
| TCGA_TCGA-EI-6510 | -1527.74 | 2122.37 | 594.6326 | 0.769798 |
| TCGA_TCGA-DY-A1DG | -2012.24 | 756.6397 | -1255.6 | 0.912811 |
| TCGA_TCGA-AG-A02X | -1129.57 | 1223.661 | 94.08731 | 0.814576 |
| TCGA_TCGA-AG-3587 | 900.2572 | 2548.373 | 3448.63 | 0.443581 |
| TCGA_TCGA-AG-3894 | 475.6566 | 2217.429 | 2693.086 | 0.540049 |
| TCGA_TCGA-AG-3999 | 736.9722 | 1921.416 | 2658.388 | 0.544329 |
| TCGA_TCGA-AG-A00H | 1315.161 | 1804.918 | 3120.08 | 0.486271 |
| TCGA_TCGA-AF-A56N | 1179.574 | 2052.732 | 3232.306 | 0.471811 |
| TCGA_TCGA-AG-A00C | -1585.95 | 1909.45 | 323.4997 | 0.794585 |
| TCGA_TCGA-EI-6885 | 1980.839 | 3310.635 | 5291.475 | 0.187957 |
| TCGA_TCGA-AG-3732 | 451.3526 | 3664.841 | 4116.193 | 0.353771 |
| TCGA_TCGA-AG-4007 | 1644.701 | 3522.176 | 5166.877 | 0.205889 |
| TCGA_TCGA-AG-A016 | -882.333 | 325.5527 | -556.78 | 0.866196 |
| TCGA_TCGA-EI-6882 | 556.6349 | 2790.406 | 3347.041 | 0.456896 |
| TCGA_TCGA-AG-3575 | 2002.812 | 3482 | 5484.812 | 0.160012 |
| TCGA_TCGA-AF-2693 | 1219.607 | 3172.715 | 4392.322 | 0.315579 |
| TCGA_TCGA-DC-6157 | 655.3221 | 1841.237 | 2496.559 | 0.5641 |
| TCGA_TCGA-AF-4110 | 2120.739 | 4034.43 | 6155.169 | 0.062261 |
| TCGA_TCGA-AG-3591 | 65.18597 | 2578.847 | 2644.033 | 0.546095 |
| TCGA_TCGA-EI-6881 | -305.912 | 2347.896 | 2041.983 | 0.617901 |
| TCGA_TCGA-AG-A008 | -1542.13 | 1589.816 | 47.68193 | 0.818509 |
| TCGA_TCGA-AG-A015 | -998.052 | 1268.777 | 270.7243 | 0.799264 |
| TCGA_TCGA-AG-3892 | 459.1569 | 4080.22 | 4539.377 | 0.295025 |
| TCGA_TCGA-G5-6233 | 705.6331 | 2251.482 | 2957.115 | 0.507033 |
| TCGA_TCGA-AG-3898 | 1442.38 | 3349 | 4791.38 | 0.259486 |
| TCGA_TCGA-AG-3578 | 717.6296 | 2941.985 | 3659.614 | 0.415616 |
| TCGA_TCGA-DC-6682 | -221.5 | 1702.516 | 1481.016 | 0.680477 |
| TCGA_TCGA-F5-6813 | 1654.562 | 3082.237 | 4736.799 | 0.267215 |
| TCGA_TCGA-F5-6702 | 3401.84 | 3479.465 | 6881.304 | NA |
| TCGA_TCGA-CL-5917 | 498.1766 | 1234.703 | 1732.88 | 0.652927 |
| TCGA_TCGA-CL-4957 | 376.8208 | 1518.504 | 1895.325 | 0.634682 |
| TCGA_TCGA-EI-6507 | 1787.366 | 4030.882 | 5818.248 | 0.111525 |
| TCGA_TCGA-DC-4749 | 39.90129 | 1591.63 | 1631.532 | 0.664122 |
| TCGA_TCGA-AF-2690 | 3937.58 | 5166.578 | 9104.158 | NA |
| TCGA_TCGA-EF-5830 | -92.7822 | 2031.989 | 1939.207 | 0.629692 |
| TCGA_TCGA-AF-3911 | 1377.28 | 2529.939 | 3907.219 | 0.382291 |
| TCGA_TCGA-DC-6155 | 186.9018 | 2424.085 | 2610.986 | 0.550152 |
| TCGA_TCGA-DC-6681 | 1692.755 | 2425.733 | 4118.488 | 0.353456 |
| TCGA_TCGA-AF-A56L | 799.2387 | 1799.356 | 2598.595 | 0.55167 |
| TCGA_TCGA-CI-6623 | 523.4065 | 2509.402 | 3032.808 | 0.497425 |
| TCGA_TCGA-AH-6897 | -742.594 | 1175.662 | 433.0677 | 0.784717 |
| TCGA_TCGA-AG-3574 | 572.7673 | 2402.24 | 2975.007 | 0.504768 |
| TCGA_TCGA-G5-6235 | -1786.19 | 1090.695 | -695.499 | 0.87619 |
| TCGA_TCGA-AG-4005 | 1309.404 | 2199.056 | 3508.46 | 0.435693 |
| TCGA_TCGA-AG-3583 | -144.448 | 2959.556 | 2815.108 | 0.524888 |
| TCGA_TCGA-AH-6547 | 2596.678 | 4850.653 | 7447.331 | NA |
| TCGA_TCGA-EI-7002 | 1549.13 | 2406.304 | 3955.434 | 0.375742 |
| TCGA_TCGA-AG-A00Y | -412.946 | 2677.573 | 2264.627 | 0.591879 |
| TCGA_TCGA-EI-6506 | 669.0039 | 3363.916 | 4032.92 | 0.365177 |
| TCGA_TCGA-AH-6643 | 613.2699 | 1864.523 | 2477.793 | 0.566373 |
| TCGA_TCGA-AG-3890 | 1103.829 | 3587.081 | 4690.91 | 0.2737 |
| TCGA_TCGA-DY-A1DF | 153.389 | 775.7574 | 929.1464 | 0.737541 |
| TCGA_TCGA-F5-6571 | 2409.289 | 4019.215 | 6428.504 | 0.022177 |
| TCGA_TCGA-AG-3882 | 1470.57 | 4492.975 | 5963.545 | 0.090307 |
| TCGA_TCGA-AG-3598 | 441.2988 | 3250.612 | 3691.91 | 0.4113 |
| TCGA_TCGA-F5-6811 | 2373.477 | 3091.877 | 5465.354 | 0.16283 |
| TCGA_TCGA-F5-6810 | 1959.873 | 2111.646 | 4071.519 | 0.359897 |
| TCGA_TCGA-F5-6464 | 3365.654 | 4246.769 | 7612.423 | NA |
| TCGA_TCGA-CI-6619 | 948.8133 | 3144.938 | 4093.751 | 0.35685 |
| TCGA_TCGA-AG-3602 | 1330.853 | 3210.405 | 4541.258 | 0.294761 |
| TCGA_TCGA-AF-2692 | 768.3579 | 2509.016 | 3277.373 | 0.465968 |
| GSE39582_GSM971957 | 1387.434 | 2783.133 | 4170.567 | 0.346294 |
| GSE39582_GSM971958 | 2395.849 | 3056.051 | 5451.9 | 0.164779 |
| GSE39582_GSM971959 | -777.457 | 1377.612 | 600.155 | 0.76928 |
| GSE39582_GSM971960 | -818.187 | 2158.854 | 1340.667 | 0.695428 |
| GSE39582_GSM971961 | 3707.078 | 4521.956 | 8229.034 | NA |
| GSE39582_GSM971962 | 3926.594 | 4296.682 | 8223.276 | NA |
| GSE39582_GSM971963 | 2508.544 | 6697.033 | 9205.578 | NA |
| GSE39582_GSM971964 | -185.248 | 2184.95 | 1999.702 | 0.622768 |
| GSE39582_GSM971965 | 898.103 | 2161.038 | 3059.141 | 0.494068 |
| GSE39582_GSM971966 | 1173.706 | 2279.809 | 3453.515 | 0.442938 |
| GSE39582_GSM971967 | -530.293 | 1258.91 | 728.6172 | 0.757096 |
| GSE39582_GSM971968 | 2856.422 | 4291.757 | 7148.179 | NA |
| GSE39582_GSM971969 | 911.2419 | 4498.677 | 5409.919 | 0.170853 |
| GSE39582_GSM971970 | -688.169 | 1043.57 | 355.4007 | 0.791733 |
| GSE39582_GSM971971 | 539.7882 | 4240.133 | 4779.921 | 0.26111 |
| GSE39582_GSM971972 | 579.7846 | 2213.362 | 2793.147 | 0.52763 |
| GSE39582_GSM971973 | -494.97 | 1096.258 | 601.2884 | 0.769174 |
| GSE39582_GSM971974 | 651.6232 | 2161.694 | 2813.318 | 0.525112 |
| GSE39582_GSM971975 | -399.93 | 2269.031 | 1869.101 | 0.637652 |
| GSE39582_GSM971976 | -549.615 | 619.0735 | 69.459 | 0.816668 |
| GSE39582_GSM971977 | 2384.466 | 4067.238 | 6451.704 | 0.018773 |
| GSE39582_GSM971978 | 823.4614 | 3067.037 | 3890.498 | 0.384558 |
| GSE39582_GSM971979 | 1145.971 | 2828.596 | 3974.567 | 0.373138 |
| GSE39582_GSM971980 | 885.0244 | 4038.889 | 4923.914 | 0.24065 |
| GSE39582_GSM971981 | 2554.089 | 4283.01 | 6837.099 | NA |
| GSE39582_GSM971982 | 2269.554 | 4385.797 | 6655.351 | NA |
| GSE39582_GSM971983 | 2224.522 | 4926.651 | 7151.173 | NA |
| GSE39582_GSM971984 | 1712.047 | 3635.57 | 5347.616 | 0.179857 |
| GSE39582_GSM971985 | -826.36 | 2282.477 | 1456.117 | 0.68315 |
| GSE39582_GSM971986 | 1559.92 | 3408.511 | 4968.431 | 0.234303 |
| GSE39582_GSM971987 | 2849.583 | 4214.187 | 7063.77 | NA |
| GSE39582_GSM971988 | -74.446 | 1497.846 | 1423.4 | 0.68665 |
| GSE39582_GSM971989 | -966.893 | 1058.925 | 92.03225 | 0.814751 |
| GSE39582_GSM971990 | 378.562 | 3651.711 | 4030.273 | 0.365539 |
| GSE39582_GSM971991 | 1673.939 | 2738.092 | 4412.031 | 0.312833 |
| GSE39582_GSM971992 | -866.093 | 2285.281 | 1419.188 | 0.687099 |
| GSE39582_GSM971993 | 507.468 | 3207.479 | 3714.947 | 0.408215 |
| GSE39582_GSM971994 | 439.8745 | 1156.63 | 1596.505 | 0.667957 |
| GSE39582_GSM971995 | 747.1138 | 3145.744 | 3892.858 | 0.384238 |
| GSE39582_GSM971996 | 4071.95 | 5494.884 | 9566.833 | NA |
| GSE39582_GSM971997 | 1553.159 | 6116.53 | 7669.688 | NA |
| GSE39582_GSM971998 | 857.0758 | 3090.037 | 3947.113 | 0.376874 |
| GSE39582_GSM971999 | 1581.791 | 4067.294 | 5649.085 | 0.136165 |
| GSE39582_GSM972000 | 933.383 | 1840.957 | 2774.34 | 0.529973 |
| GSE39582_GSM972001 | -647.112 | 2700.701 | 2053.589 | 0.61656 |
| GSE39582_GSM972002 | 56.16011 | 1714.184 | 1770.344 | 0.648752 |
| GSE39582_GSM972003 | -910.39 | 1518.888 | 608.4979 | 0.768497 |
| GSE39582_GSM972004 | 20.07642 | 2110.894 | 2130.97 | 0.607578 |
| GSE39582_GSM972005 | -2629.26 | -13.7333 | -2642.99 | 0.976542 |
| GSE39582_GSM972006 | 1610.699 | 2674.387 | 4285.086 | 0.330476 |
| GSE39582_GSM972007 | 2955.31 | 3950.556 | 6905.866 | NA |
| GSE39582_GSM972008 | 2273.886 | 3698.829 | 5972.715 | 0.088966 |
| GSE39582_GSM972009 | 1214.26 | 2605.524 | 3819.785 | 0.394119 |
| GSE39582_GSM972010 | 1717.64 | 2878.684 | 4596.324 | 0.287027 |
| GSE39582_GSM972011 | 4191.215 | 3686.432 | 7877.647 | NA |
| GSE39582_GSM972012 | -844.538 | 1945.159 | 1100.621 | 0.720311 |
| GSE39582_GSM972013 | 739.1146 | 905.946 | 1645.061 | 0.662636 |
| GSE39582_GSM972014 | -29.4535 | 1011.575 | 982.121 | 0.732267 |
| GSE39582_GSM972015 | -84.2023 | 1665.078 | 1580.876 | 0.669663 |
| GSE39582_GSM972016 | 887.3179 | 2593.039 | 3480.357 | 0.439402 |
| GSE39582_GSM972017 | 585.4996 | 2886.122 | 3471.621 | 0.440554 |
| GSE39582_GSM972018 | 1639.036 | 3913.548 | 5552.584 | 0.150184 |
| GSE39582_GSM972019 | 2346.082 | 4650.901 | 6996.982 | NA |
| GSE39582_GSM972020 | 968.0527 | 2827.719 | 3795.772 | 0.397356 |
| GSE39582_GSM972021 | -906.167 | 808.3146 | -97.8521 | 0.830594 |
| GSE39582_GSM972022 | 676.7599 | 2741.286 | 3418.046 | 0.4476 |
| GSE39582_GSM972023 | 461.9961 | 2193.152 | 2655.148 | 0.544728 |
| GSE39582_GSM972024 | -972.411 | 1120.967 | 148.5563 | 0.809912 |
| GSE39582_GSM972025 | 192.0303 | 2379.385 | 2571.415 | 0.554994 |
| GSE39582_GSM972026 | 2386.74 | 3692.694 | 6079.435 | 0.073353 |
| GSE39582_GSM972027 | -643.518 | 850.1989 | 206.6809 | 0.804879 |
| GSE39582_GSM972028 | 553.1254 | 3442.638 | 3995.763 | 0.370249 |
| GSE39582_GSM972029 | 1802.925 | 2490.691 | 4293.617 | 0.329294 |
| GSE39582_GSM972030 | 2115.969 | 2260.305 | 4376.274 | 0.317814 |
| GSE39582_GSM972031 | 865.6305 | 1486.355 | 2351.986 | 0.581495 |
| GSE39582_GSM972032 | 357.5234 | 1822.559 | 2180.082 | 0.601836 |
| GSE39582_GSM972033 | -2017.47 | 425.2112 | -1592.26 | 0.93187 |
| GSE39582_GSM972034 | -1506.85 | 1178.59 | -328.265 | 0.84895 |
| GSE39582_GSM972035 | -1578.02 | 687.5728 | -890.448 | 0.889622 |
| GSE39582_GSM972036 | -383.972 | 1534.074 | 1150.102 | 0.715254 |
| GSE39582_GSM972037 | -1312.34 | 1825.093 | 512.7533 | 0.777413 |
| GSE39582_GSM972038 | -538.656 | 1504.385 | 965.7294 | 0.733904 |
| GSE39582_GSM972039 | 3123.723 | 3706.589 | 6830.312 | NA |
| GSE39582_GSM972040 | -172.191 | 1584.696 | 1412.505 | 0.687812 |
| GSE39582_GSM972041 | 359.5265 | 1320.742 | 1680.268 | 0.658757 |
| GSE39582_GSM972042 | -727.902 | 2270.001 | 1542.099 | 0.673879 |
| GSE39582_GSM972043 | -162.662 | 1104.312 | 941.6498 | 0.7363 |
| GSE39582_GSM972044 | -2018.06 | 749.7774 | -1268.28 | 0.91357 |
| GSE39582_GSM972045 | -819.984 | 1143.337 | 323.3526 | 0.794598 |
| GSE39582_GSM972046 | 2034.127 | 3994.75 | 6028.876 | 0.080752 |
| GSE39582_GSM972047 | 554.1221 | 971.8564 | 1525.979 | 0.675626 |
| GSE39582_GSM972048 | -333.033 | 1261.788 | 928.7554 | 0.737579 |
| GSE39582_GSM972049 | -1323.48 | 741.1638 | -582.321 | 0.868063 |
| GSE39582_GSM972050 | -882.624 | 1967.741 | 1085.117 | 0.721888 |
| GSE39582_GSM972051 | 2875.967 | 2278.968 | 5154.936 | 0.207603 |
| GSE39582_GSM972052 | 680.6204 | 1827.097 | 2507.717 | 0.562747 |
| GSE39582_GSM972053 | -912.182 | 645.5924 | -266.59 | 0.844131 |
| GSE39582_GSM972054 | 2532.4 | 3537.791 | 6070.192 | 0.074706 |
| GSE39582_GSM972055 | 931.2242 | 3047.28 | 3978.504 | 0.372602 |
| GSE39582_GSM972056 | -166.78 | 1929.533 | 1762.753 | 0.649599 |
| GSE39582_GSM972057 | -492.249 | 1822.137 | 1329.888 | 0.696564 |
| GSE39582_GSM972058 | 2034.837 | 4649.868 | 6684.705 | NA |
| GSE39582_GSM972059 | 2263.66 | 4664.639 | 6928.298 | NA |
| GSE39582_GSM972060 | 1236.051 | 3243.169 | 4479.22 | 0.30345 |
| GSE39582_GSM972061 | 27.75367 | 2273.414 | 2301.167 | 0.587547 |
| GSE39582_GSM972062 | 794.3745 | 2623.622 | 3417.997 | 0.447606 |
| GSE39582_GSM972063 | 2072.512 | 3328.813 | 5401.325 | 0.172096 |
| GSE39582_GSM972064 | 2737.3 | 4241.371 | 6978.671 | NA |
| GSE39582_GSM972065 | 1312.473 | 2863.142 | 4175.615 | 0.345599 |
| GSE39582_GSM972066 | 868.1421 | 3151.69 | 4019.832 | 0.366965 |
| GSE39582_GSM972067 | 2172.213 | 3315.491 | 5487.704 | 0.159593 |
| GSE39582_GSM972068 | 4129.027 | 4664.556 | 8793.583 | NA |
| GSE39582_GSM972069 | 1152.129 | 1948.925 | 3101.054 | 0.48871 |
| GSE39582_GSM972070 | 805.8228 | 3212.241 | 4018.064 | 0.367207 |
| GSE39582_GSM972071 | 1379.166 | 2224.532 | 3603.698 | 0.423068 |
| GSE39582_GSM972072 | 2645.021 | 4687.345 | 7332.366 | NA |
| GSE39582_GSM972073 | 2844.651 | 4217.842 | 7062.493 | NA |
| GSE39582_GSM972074 | 819.2227 | 3398.79 | 4218.012 | 0.339753 |
| GSE39582_GSM972075 | 856.8258 | 3191.694 | 4048.52 | 0.363045 |
| GSE39582_GSM972076 | 2063.5 | 3994.088 | 6057.588 | 0.07655 |
| GSE39582_GSM972077 | 1802.715 | 3629.599 | 5432.314 | 0.167614 |
| GSE39582_GSM972078 | 1941.272 | 2418.127 | 4359.399 | 0.320161 |
| GSE39582_GSM972079 | 2290.223 | 4541.911 | 6832.134 | NA |
| GSE39582_GSM972080 | 1154.874 | 2731.12 | 3885.994 | 0.385168 |
| GSE39582_GSM972081 | 747.4977 | 2333.211 | 3080.708 | 0.491313 |
| GSE39582_GSM972082 | 3086.721 | 3696.751 | 6783.472 | NA |
| GSE39582_GSM972083 | 1361.319 | 3544.633 | 4905.952 | 0.243209 |
| GSE39582_GSM972084 | 2201.34 | 4439.602 | 6640.942 | NA |
| GSE39582_GSM972085 | 1205.345 | 3433.353 | 4638.698 | 0.281063 |
| GSE39582_GSM972086 | -148.616 | 1265.168 | 1116.552 | 0.718687 |
| GSE39582_GSM972087 | -737.15 | 2782.627 | 2045.476 | 0.617497 |
| GSE39582_GSM972088 | 1882.083 | 3692.565 | 5574.648 | 0.146981 |
| GSE39582_GSM972089 | 2699.515 | 3112.956 | 5812.471 | 0.112368 |
| GSE39582_GSM972090 | -1558.43 | 562.109 | -996.321 | 0.896612 |
| GSE39582_GSM972091 | -1548.3 | 1524.366 | -23.9306 | 0.824502 |
| GSE39582_GSM972092 | 2319.581 | 3092.67 | 5412.251 | 0.170516 |
| GSE39582_GSM972093 | 1967.244 | 2210.456 | 4177.7 | 0.345312 |
| GSE39582_GSM972094 | 1702.287 | 3494.888 | 5197.175 | 0.201534 |
| GSE39582_GSM972095 | 1596.739 | 2332.199 | 3928.938 | 0.379344 |
| GSE39582_GSM972096 | -1861.37 | 349.5159 | -1511.85 | 0.927524 |
| GSE39582_GSM972097 | 2713.343 | 2634.309 | 5347.652 | 0.179852 |
| GSE39582_GSM972098 | -1571.62 | 1081.57 | -490.049 | 0.861259 |
| GSE39582_GSM972099 | -860.68 | 1121.156 | 260.4762 | 0.800167 |
| GSE39582_GSM972100 | -485.072 | 1860.505 | 1375.433 | 0.691751 |
| GSE39582_GSM972101 | 282.2746 | 3001.263 | 3283.538 | 0.465168 |
| GSE39582_GSM972102 | 2149.78 | 5284.851 | 7434.631 | NA |
| GSE39582_GSM972103 | 1304.451 | 3523.428 | 4827.879 | 0.254308 |
| GSE39582_GSM972104 | 3117.788 | 3926.545 | 7044.333 | NA |
| GSE39582_GSM972105 | 125.9072 | 1315.927 | 1441.835 | 0.68468 |
| GSE39582_GSM972106 | 323.8725 | 1410.066 | 1733.939 | 0.652809 |
| GSE39582_GSM972107 | 750.878 | 3125.736 | 3876.614 | 0.386439 |
| GSE39582_GSM972108 | -76.269 | 2500.654 | 2424.385 | 0.572816 |
| GSE39582_GSM972109 | -272.726 | 2485.751 | 2213.025 | 0.597967 |
| GSE39582_GSM972110 | -308.092 | 2711.1 | 2403.009 | 0.575385 |
| GSE39582_GSM972111 | 182.8531 | 2941.518 | 3124.371 | 0.485721 |
| GSE39582_GSM972112 | 3057.157 | 4401.87 | 7459.028 | NA |
| GSE39582_GSM972113 | 3665.441 | 4002.412 | 7667.854 | NA |
| GSE39582_GSM972114 | -30.3731 | 2096.608 | 2066.234 | 0.615098 |
| GSE39582_GSM972115 | 131.2888 | 1861.592 | 1992.881 | 0.623552 |
| GSE39582_GSM972116 | 715.7699 | 2543.463 | 3259.233 | 0.468323 |
| GSE39582_GSM972117 | 1449.089 | 5375.821 | 6824.911 | NA |
| GSE39582_GSM972118 | -1110.54 | 956.8685 | -153.67 | 0.835128 |
| GSE39582_GSM972119 | 108.8061 | 1246.119 | 1354.925 | 0.693922 |
| GSE39582_GSM972120 | 1658.04 | 3520.896 | 5178.936 | 0.204156 |
| GSE39582_GSM972121 | 48.26789 | 2891.446 | 2939.714 | 0.509233 |
| GSE39582_GSM972122 | -1151.01 | 767.1276 | -383.879 | 0.853236 |
| GSE39582_GSM972123 | -1036.77 | 1559.229 | 522.4611 | 0.776516 |
| GSE39582_GSM972124 | 849.7991 | 2946.799 | 3796.598 | 0.397245 |
| GSE39582_GSM972125 | 1944.385 | 2895.329 | 4839.714 | 0.252628 |
| GSE39582_GSM972126 | -1134.64 | 656.3056 | -478.332 | 0.860384 |
| GSE39582_GSM972127 | 1407.609 | 2789.547 | 4197.156 | 0.34263 |
| GSE39582_GSM972128 | 1722.552 | 3370.912 | 5093.464 | 0.216422 |
| GSE39582_GSM972129 | 140.9829 | 2925.179 | 3066.162 | 0.493172 |
| GSE39582_GSM972130 | -1019.19 | 1255.623 | 236.4318 | 0.802279 |
| GSE39582_GSM972131 | 2793.165 | 3602.321 | 6395.486 | 0.027023 |
| GSE39582_GSM972132 | 1018.116 | 1818.275 | 2836.39 | 0.522227 |
| GSE39582_GSM972133 | 2283.416 | 3515.388 | 5798.803 | 0.114361 |
| GSE39582_GSM972134 | 1180.267 | 3021.89 | 4202.157 | 0.341941 |
| GSE39582_GSM972135 | -1803.5 | 683.4756 | -1120.03 | 0.904505 |
| GSE39582_GSM972136 | -50.782 | 1888.61 | 1837.828 | 0.641182 |
| GSE39582_GSM972137 | -342.785 | 1596.438 | 1253.653 | 0.704549 |
| GSE39582_GSM972138 | -708.639 | 1448.077 | 739.4381 | 0.756057 |
| GSE39582_GSM972139 | -699.205 | 1281.602 | 582.3974 | 0.770943 |
| GSE39582_GSM972140 | 3053.198 | 3355.392 | 6408.591 | 0.0251 |
| GSE39582_GSM972141 | 1804.767 | 3142.932 | 4947.699 | 0.23726 |
| GSE39582_GSM972142 | 152.0595 | 1800.777 | 1952.837 | 0.628136 |
| GSE39582_GSM972143 | 980.3022 | 2513.599 | 3493.901 | 0.437615 |
| GSE39582_GSM972144 | -601.154 | 1604.584 | 1003.43 | 0.730133 |
| GSE39582_GSM972145 | 620.469 | 2799.585 | 3420.054 | 0.447336 |
| GSE39582_GSM972146 | 1725.09 | 2390.289 | 4115.379 | 0.353883 |
| GSE39582_GSM972147 | 427.4937 | 1761.932 | 2189.426 | 0.60074 |
| GSE39582_GSM972148 | 461.1834 | 2232.124 | 2693.307 | 0.540022 |
| GSE39582_GSM972149 | -480.968 | 1597.493 | 1116.525 | 0.71869 |
| GSE39582_GSM972150 | 597.877 | 2854.096 | 3451.973 | 0.443141 |
| GSE39582_GSM972151 | -950.496 | 1811.971 | 861.4746 | 0.744212 |
| GSE39582_GSM972152 | -652.993 | 1640.6 | 987.6066 | 0.731719 |
| GSE39582_GSM972153 | 470.0112 | 2510.727 | 2980.738 | 0.504041 |
| GSE39582_GSM972154 | -224.395 | 1670.737 | 1446.342 | 0.684198 |
| GSE39582_GSM972155 | -970.446 | 971.0082 | 0.562632 | 0.822462 |
| GSE39582_GSM972156 | 535.2631 | 2147.84 | 2683.103 | 0.541282 |
| GSE39582_GSM972157 | 964.7453 | 2007.584 | 2972.33 | 0.505107 |
| GSE39582_GSM972158 | 2728.277 | 4560.918 | 7289.196 | NA |
| GSE39582_GSM972159 | -15.1019 | 1901.221 | 1886.12 | 0.635726 |
| GSE39582_GSM972160 | 799.8759 | 2905.061 | 3704.937 | 0.409556 |
| GSE39582_GSM972161 | -243.199 | 870.39 | 627.1914 | 0.766738 |
| GSE39582_GSM972162 | 1539.58 | 3106.078 | 4645.659 | 0.280083 |
| GSE39582_GSM972163 | 542.4047 | 2648.971 | 3191.376 | 0.4771 |
| GSE39582_GSM972164 | 1626.853 | 2599.488 | 4226.342 | 0.338603 |
| GSE39582_GSM972165 | 1820.633 | 2646.739 | 4467.372 | 0.305107 |
| GSE39582_GSM972166 | 395.1983 | 1869.572 | 2264.77 | 0.591862 |
| GSE39582_GSM972167 | 613.9316 | 3349.56 | 3963.491 | 0.374646 |
| GSE39582_GSM972168 | 1502.066 | 3516.367 | 5018.433 | 0.227161 |
| GSE39582_GSM972169 | -220.059 | 2142.642 | 1922.583 | 0.631585 |
| GSE39582_GSM972170 | 1486.534 | 2849.537 | 4336.071 | 0.323404 |
| GSE39582_GSM972171 | 2290.627 | 5282.296 | 7572.923 | NA |
| GSE39582_GSM972172 | -1153.13 | 1570.454 | 417.3237 | 0.786148 |
| GSE39582_GSM972173 | 65.96357 | 1716.156 | 1782.119 | 0.647436 |
| GSE39582_GSM972174 | -419.636 | 1676.258 | 1256.622 | 0.70424 |
| GSE39582_GSM972175 | -72.9394 | 1599.353 | 1526.413 | 0.675579 |
| GSE39582_GSM972176 | 1961.737 | 3163.172 | 5124.909 | 0.211913 |
| GSE39582_GSM972177 | 112.5954 | 2451.697 | 2564.293 | 0.555863 |
| GSE39582_GSM972178 | -815.556 | 2175.26 | 1359.704 | 0.693417 |
| GSE39582_GSM972179 | 1865.227 | 5131.888 | 6997.115 | NA |
| GSE39582_GSM972180 | 87.98731 | 4361.753 | 4449.741 | 0.307571 |
| GSE39582_GSM972181 | 3596.571 | 4018.836 | 7615.406 | NA |
| GSE39582_GSM972182 | -665.569 | 1541.051 | 875.4825 | 0.742837 |
| GSE39582_GSM972183 | -1808.66 | 687.8983 | -1120.76 | 0.90455 |
| GSE39582_GSM972184 | 2591.732 | 3278.496 | 5870.228 | 0.10394 |
| GSE39582_GSM972185 | -348.682 | 752.8799 | 404.1984 | 0.787337 |
| GSE39582_GSM972186 | 3090.465 | 4041.943 | 7132.408 | NA |
| GSE39582_GSM972187 | 2416.796 | 3201.004 | 5617.8 | 0.140713 |
| GSE39582_GSM972188 | -153.254 | 2036.055 | 1882.802 | 0.636102 |
| GSE39582_GSM972189 | 525.1546 | 4469.391 | 4994.545 | 0.230574 |
| GSE39582_GSM972190 | -372.717 | 1376.034 | 1003.317 | 0.730145 |
| GSE39582_GSM972191 | 34.68532 | 1334.085 | 1368.77 | 0.692457 |
| GSE39582_GSM972192 | 837.7287 | 3270.079 | 4107.807 | 0.354922 |
| GSE39582_GSM972193 | -674.915 | 1809.715 | 1134.8 | 0.716822 |
| GSE39582_GSM972194 | -293.368 | 2491.506 | 2198.138 | 0.599717 |
| GSE39582_GSM972195 | -570.036 | 2636.739 | 2066.703 | 0.615044 |
| GSE39582_GSM972196 | -789.319 | 2672.394 | 1883.075 | 0.636071 |
| GSE39582_GSM972197 | -359.304 | 1777.202 | 1417.898 | 0.687237 |
| GSE39582_GSM972198 | -849.324 | 1544.791 | 695.4672 | 0.760266 |
| GSE39582_GSM972199 | -340.662 | 1618.763 | 1278.1 | 0.701998 |
| GSE39582_GSM972200 | 663.8299 | 2065.83 | 2729.66 | 0.535523 |
| GSE39582_GSM972201 | 2547.247 | 3945.346 | 6492.592 | 0.012772 |
| GSE39582_GSM972202 | 6.768644 | 2476.882 | 2483.651 | 0.565664 |
| GSE39582_GSM972203 | 893.6265 | 3311.541 | 4205.167 | 0.341525 |
| GSE39582_GSM972204 | 2246.872 | 3703.927 | 5950.798 | 0.09217 |
| GSE39582_GSM972205 | 1097.418 | 4474 | 5571.419 | 0.14745 |
| GSE39582_GSM972206 | 1559.962 | 2326.033 | 3885.995 | 0.385168 |
| GSE39582_GSM972207 | 950.8701 | 2399.208 | 3350.078 | 0.456499 |
| GSE39582_GSM972208 | 3547.633 | 4859.71 | 8407.343 | NA |
| GSE39582_GSM972209 | 932.6816 | 4347.172 | 5279.854 | 0.189633 |
| GSE39582_GSM972210 | 1526.129 | 2143.785 | 3669.914 | 0.414241 |
| GSE39582_GSM972211 | 707.0554 | 1336.641 | 2043.697 | 0.617703 |
| GSE39582_GSM972212 | -575.467 | 2411.581 | 1836.114 | 0.641375 |
| GSE39582_GSM972213 | 440.6493 | 936.7932 | 1377.442 | 0.691538 |
| GSE39582_GSM972214 | -1206.41 | 1504.201 | 297.7866 | 0.796871 |
| GSE39582_GSM972215 | 1285.076 | 3408.61 | 4693.686 | 0.273308 |
| GSE39582_GSM972216 | -510.415 | 2250.781 | 1740.365 | 0.652094 |
| GSE39582_GSM972217 | 344.4436 | 1709.297 | 2053.74 | 0.616543 |
| GSE39582_GSM972218 | -1293.58 | 1678.768 | 385.1873 | 0.789054 |
| GSE39582_GSM972219 | 51.51426 | 1202.864 | 1254.378 | 0.704473 |
| GSE39582_GSM972220 | 1790.376 | 5133.375 | 6923.752 | NA |
| GSE39582_GSM972221 | -377.866 | 2313.018 | 1935.153 | 0.630154 |
| GSE39582_GSM972222 | 227.9135 | 2127.956 | 2355.87 | 0.581031 |
| GSE39582_GSM972223 | 1911.263 | 3695.579 | 5606.842 | 0.142305 |
| GSE39582_GSM972224 | 2460.195 | 4957.942 | 7418.137 | NA |
| GSE39582_GSM972225 | 752.8422 | 3133.217 | 3886.059 | 0.38516 |
| GSE39582_GSM972226 | 362.8213 | 2538.843 | 2901.665 | 0.514032 |
| GSE39582_GSM972227 | -675.25 | 2185.142 | 1509.892 | 0.677365 |
| GSE39582_GSM972228 | -540.127 | 2716.712 | 2176.586 | 0.602246 |
| GSE39582_GSM972229 | 1378.504 | 4016.512 | 5395.016 | 0.173009 |
| GSE39582_GSM972230 | -18.8654 | 1306.516 | 1287.651 | 0.700999 |
| GSE39582_GSM972231 | 3030.434 | 4170.142 | 7200.575 | NA |
| GSE39582_GSM972232 | 2413.68 | 4027.733 | 6441.412 | 0.020283 |
| GSE39582_GSM972233 | 969.9541 | 3611.338 | 4581.293 | 0.28914 |
| GSE39582_GSM972234 | 3777.221 | 3541.806 | 7319.027 | NA |
| GSE39582_GSM972235 | 2103.57 | 2841.978 | 4945.547 | 0.237567 |
| GSE39582_GSM972236 | 489.584 | 2012.526 | 2502.11 | 0.563427 |
| GSE39582_GSM972237 | 0.139861 | 1815.055 | 1815.195 | 0.643728 |
| GSE39582_GSM972238 | 1760.883 | 2757.619 | 4518.503 | 0.297951 |
| GSE39582_GSM972239 | 1922.666 | 4291.119 | 6213.785 | 0.053672 |
| GSE39582_GSM972240 | 2257.856 | 4262.378 | 6520.233 | 0.008714 |
| GSE39582_GSM972241 | 7.87666 | 2847.786 | 2855.663 | 0.519812 |
| GSE39582_GSM972242 | 2300.826 | 3486.444 | 5787.27 | 0.116043 |
| GSE39582_GSM972243 | 1037.704 | 3566.789 | 4604.493 | 0.285878 |
| GSE39582_GSM972244 | 1157.413 | 3991.154 | 5148.566 | 0.208518 |
| GSE39582_GSM972245 | 454.4621 | 2393.32 | 2847.782 | 0.5208 |
| GSE39582_GSM972246 | 349.1516 | 1185.037 | 1534.189 | 0.674737 |
| GSE39582_GSM972247 | -352.082 | 2278.741 | 1926.659 | 0.631121 |
| GSE39582_GSM972248 | 120.3412 | 2318.536 | 2438.877 | 0.571071 |
| GSE39582_GSM972249 | 986.5865 | 2735.894 | 3722.481 | 0.407205 |
| GSE39582_GSM972250 | 765.1545 | 1704.628 | 2469.782 | 0.567341 |
| GSE39582_GSM972251 | 2280.663 | 2223.029 | 4503.692 | 0.300026 |
| GSE39582_GSM972252 | 1879.505 | 3200.407 | 5079.913 | 0.218363 |
| GSE39582_GSM972253 | -30.8509 | 1978.501 | 1947.65 | 0.628728 |
| GSE39582_GSM972254 | 1942.833 | 2816.794 | 4759.626 | 0.263985 |
| GSE39582_GSM972255 | 2161.466 | 1884.539 | 4046.005 | 0.363389 |
| GSE39582_GSM972256 | 327.3785 | 2094.418 | 2421.797 | 0.573128 |
| GSE39582_GSM972257 | -122.073 | 2617.784 | 2495.711 | 0.564203 |
| GSE39582_GSM972258 | 423.5105 | 1835.563 | 2259.073 | 0.592536 |
| GSE39582_GSM972259 | 743.6596 | 3180.786 | 3924.446 | 0.379954 |
| GSE39582_GSM972260 | -94.8782 | 1989.371 | 1894.493 | 0.634777 |
| GSE39582_GSM972261 | 585.8566 | 3405.917 | 3991.774 | 0.370793 |
| GSE39582_GSM972262 | 4634.674 | 5141.509 | 9776.184 | NA |
| GSE39582_GSM972263 | 1173.36 | 3850.653 | 5024.013 | 0.226363 |
| GSE39582_GSM972264 | -160.35 | 2948.19 | 2787.839 | 0.528291 |
| GSE39582_GSM972265 | 546.4941 | 2277.737 | 2824.231 | 0.523748 |
| GSE39582_GSM972266 | -1434.44 | 463.4061 | -971.032 | 0.894962 |
| GSE39582_GSM972267 | 547.6803 | 2081.048 | 2628.728 | 0.547976 |
| GSE39582_GSM972268 | -411.453 | 1753.374 | 1341.92 | 0.695295 |
| GSE39582_GSM972269 | -290.604 | 3490.503 | 3199.898 | 0.476 |
| GSE39582_GSM972270 | 505.9678 | 3430.705 | 3936.673 | 0.378293 |
| GSE39582_GSM972271 | -605.046 | 1334.566 | 729.5201 | 0.75701 |
| GSE39582_GSM972272 | -793.832 | 1653.1 | 859.2684 | 0.744429 |
| GSE39582_GSM972273 | -701.347 | 2483.615 | 1782.268 | 0.647419 |
| GSE39582_GSM972274 | 1451.729 | 3892.619 | 5344.348 | 0.180329 |
| GSE39582_GSM972275 | 2828.35 | 5702.986 | 8531.336 | NA |
| GSE39582_GSM972276 | -244.694 | 2801.23 | 2556.536 | 0.556809 |
| GSE39582_GSM972277 | -685.412 | 1493.035 | 807.6229 | 0.749469 |
| GSE39582_GSM972278 | 290.4836 | 3385.018 | 3675.501 | 0.413494 |
| GSE39582_GSM972279 | 2577.125 | 3402.763 | 5979.888 | 0.087917 |
| GSE39582_GSM972280 | 1370.313 | 3746.143 | 5116.456 | 0.213125 |
| GSE39582_GSM972281 | 838.9541 | 3366.193 | 4205.147 | 0.341528 |
| GSE39582_GSM972282 | -71.3162 | 2271.474 | 2200.158 | 0.59948 |
| GSE39582_GSM972283 | 906.0467 | 3464.483 | 4370.529 | 0.318613 |
| GSE39582_GSM972284 | -1431.94 | 1408.717 | -23.2226 | 0.824443 |
| GSE39582_GSM972285 | 133.941 | 1750.197 | 1884.138 | 0.635951 |
| GSE39582_GSM972286 | 1020.077 | 3062.388 | 4082.464 | 0.358397 |
| GSE39582_GSM972287 | 2299.241 | 4470.311 | 6769.552 | NA |
| GSE39582_GSM972288 | -659.247 | 1292.54 | 633.2929 | 0.766163 |
| GSE39582_GSM972289 | -43.7982 | 1746.555 | 1702.757 | 0.65627 |
| GSE39582_GSM972290 | 2411.96 | 4538.856 | 6950.816 | NA |
| GSE39582_GSM972291 | 1509.066 | 2581.808 | 4090.874 | 0.357245 |
| GSE39582_GSM972292 | 192.0584 | 2959.631 | 3151.69 | 0.482212 |
| GSE39582_GSM972293 | 1716.768 | 3493.521 | 5210.289 | 0.199649 |
| GSE39582_GSM972294 | 1684.632 | 5566.786 | 7251.419 | NA |
| GSE39582_GSM972295 | -991.994 | 1005.097 | 13.10235 | 0.821414 |
| GSE39582_GSM972296 | -763.61 | 3019.852 | 2256.242 | 0.592871 |
| GSE39582_GSM972297 | 2466.363 | 4303.691 | 6770.054 | NA |
| GSE39582_GSM972298 | 167.1322 | 2446.923 | 2614.055 | 0.549776 |
| GSE39582_GSM972299 | 1645.195 | 2657.873 | 4303.068 | 0.327984 |
| GSE39582_GSM972300 | 60.37315 | 2550.335 | 2610.708 | 0.550186 |
| GSE39582_GSM972301 | 2591.446 | 4915.228 | 7506.674 | NA |
| GSE39582_GSM972302 | -257.075 | 1666.186 | 1409.111 | 0.688173 |
| GSE39582_GSM972303 | -752.888 | 1384.412 | 631.5237 | 0.76633 |
| GSE39582_GSM972304 | -1512.26 | 2026.479 | 514.2165 | 0.777278 |
| GSE39582_GSM972305 | 1747.024 | 5618.597 | 7365.621 | NA |
| GSE39582_GSM972306 | -613.931 | 1272.949 | 659.0176 | 0.763731 |
| GSE39582_GSM972307 | -909.091 | 2468.802 | 1559.711 | 0.671967 |
| GSE39582_GSM972308 | 781.549 | 4201.322 | 4982.871 | 0.232241 |
| GSE39582_GSM972309 | -1788.51 | 710.6336 | -1077.88 | 0.901849 |
| GSE39582_GSM972310 | 768.1668 | 2263.533 | 3031.699 | 0.497566 |
| GSE39582_GSM972311 | -124.377 | 668.0215 | 543.6444 | 0.774553 |
| GSE39582_GSM972312 | -1196.95 | 1080.052 | -116.901 | 0.832147 |
| GSE39582_GSM972313 | -1042.89 | 297.1628 | -745.73 | 0.87972 |
| GSE39582_GSM972314 | 282.7318 | 4082.13 | 4364.862 | 0.319402 |
| GSE39582_GSM972315 | -591.751 | 2610.351 | 2018.599 | 0.620596 |
| GSE39582_GSM972316 | 253.9255 | 2539.305 | 2793.231 | 0.527619 |
| GSE39582_GSM972317 | 895.9006 | 2801.434 | 3697.334 | 0.410574 |
| GSE39582_GSM972318 | 586.7607 | 2196.578 | 2783.338 | 0.528852 |
| GSE39582_GSM972319 | -543.625 | 1507.433 | 963.8082 | 0.734095 |
| GSE39582_GSM972320 | -214.878 | 1495.58 | 1280.702 | 0.701726 |
| GSE39582_GSM972321 | 712.4348 | 2542.762 | 3255.197 | 0.468846 |
| GSE39582_GSM972322 | 1060.446 | 4395.449 | 5455.895 | 0.1642 |
| GSE39582_GSM972323 | -395.067 | 1259.227 | 864.1602 | 0.743949 |
| GSE39582_GSM972324 | -18.4304 | 1950.445 | 1932.015 | 0.630511 |
| GSE39582_GSM972325 | 704.5785 | 3484.514 | 4189.093 | 0.343742 |
| GSE39582_GSM972326 | -1081.47 | 2138.616 | 1057.148 | 0.724723 |
| GSE39582_GSM972327 | 2807.731 | 4990.452 | 7798.183 | NA |
| GSE39582_GSM972328 | 805.8779 | 1155.128 | 1961.006 | 0.627203 |
| GSE39582_GSM972329 | 1830.108 | 3548.246 | 5378.354 | 0.175417 |
| GSE39582_GSM972330 | 1810.942 | 3832.8 | 5643.742 | 0.136942 |
| GSE39582_GSM972331 | 660.6449 | 2525.499 | 3186.144 | 0.477775 |
| GSE39582_GSM972332 | 967.1659 | 1840.911 | 2808.077 | 0.525767 |
| GSE39582_GSM972333 | 244.3299 | 1952.274 | 2196.604 | 0.599898 |
| GSE39582_GSM972334 | 3211.947 | 5484.023 | 8695.969 | NA |
| GSE39582_GSM972335 | 238.9084 | 2394.399 | 2633.307 | 0.547413 |
| GSE39582_GSM972336 | 540.7152 | 2003.206 | 2543.921 | 0.558346 |
| GSE39582_GSM972337 | 1845.331 | 6314.156 | 8159.488 | NA |
| GSE39582_GSM972338 | -93.0828 | 2912.883 | 2819.8 | 0.524302 |
| GSE39582_GSM972339 | 722.9767 | 2168.219 | 2891.196 | 0.515349 |
| GSE39582_GSM972340 | 25.68733 | 2583.676 | 2609.363 | 0.550351 |
| GSE39582_GSM972341 | 719.2167 | 3144.372 | 3863.589 | 0.388201 |
| GSE39582_GSM972342 | 1040.408 | 2324.076 | 3364.484 | 0.454617 |
| GSE39582_GSM972343 | 280.0743 | 2420.747 | 2700.821 | 0.539093 |
| GSE39582_GSM972344 | -280.464 | 1380.898 | 1100.433 | 0.72033 |
| GSE39582_GSM972345 | 2651.446 | 3030.659 | 5682.104 | 0.131361 |
| GSE39582_GSM972346 | -612.427 | 966.9335 | 354.5066 | 0.791813 |
| GSE39582_GSM972347 | 337.5691 | 2600.494 | 2938.063 | 0.509441 |
| GSE39582_GSM972348 | -308.683 | 2228.258 | 1919.575 | 0.631928 |
| GSE39582_GSM972349 | 4525.351 | 4320.432 | 8845.783 | NA |
| GSE39582_GSM972350 | -1812.39 | 1225.633 | -586.76 | 0.868386 |
| GSE39582_GSM972351 | 78.55379 | 1469.642 | 1548.196 | 0.673218 |
| GSE39582_GSM972352 | 3232.102 | 2694.558 | 5926.66 | 0.095698 |
| GSE39582_GSM972353 | 1084.86 | 3400.886 | 4485.747 | 0.302537 |
| GSE39582_GSM972354 | -44.0645 | 2507.879 | 2463.815 | 0.568063 |
| GSE39582_GSM972355 | 1904.042 | 2326.819 | 4230.861 | 0.337978 |
| GSE39582_GSM972356 | -171.683 | 2821.562 | 2649.879 | 0.545376 |
| GSE39582_GSM972357 | -317.206 | 2769.178 | 2451.972 | 0.569492 |
| GSE39582_GSM972358 | 1078.915 | 3181.76 | 4260.675 | 0.333856 |
| GSE39582_GSM972359 | 3042.988 | 4384.907 | 7427.894 | NA |
| GSE39582_GSM972360 | 1528.44 | 2799.077 | 4327.517 | 0.324592 |
| GSE39582_GSM972361 | 3844.38 | 6490.141 | 10334.52 | NA |
| GSE39582_GSM972362 | 76.26538 | 2082.093 | 2158.358 | 0.60438 |
| GSE39582_GSM972363 | 831.4696 | 3095.6 | 3927.07 | 0.379597 |
| GSE39582_GSM972364 | -251.284 | 899.1629 | 647.8789 | 0.764785 |
| GSE39582_GSM972365 | 2385.4 | 4249.778 | 6635.177 | NA |
| GSE39582_GSM972366 | 1827.245 | 3582.749 | 5409.994 | 0.170843 |
| GSE39582_GSM972367 | 3191.754 | 3779.098 | 6970.852 | NA |
| GSE39582_GSM972368 | -1335.58 | 2039.545 | 703.9696 | 0.759455 |
| GSE39582_GSM972369 | 3660.864 | 4865.93 | 8526.793 | NA |
| GSE39582_GSM972370 | 473.8323 | 2820.764 | 3294.597 | 0.46373 |
| GSE39582_GSM972371 | 204.6515 | 1906.866 | 2111.517 | 0.609843 |
| GSE39582_GSM972372 | 1174.916 | 2951.385 | 4126.302 | 0.352383 |
| GSE39582_GSM972373 | -507.677 | 359.1842 | -148.493 | 0.83471 |
| GSE39582_GSM972374 | 572.1649 | 3242.432 | 3814.597 | 0.394819 |
| GSE39582_GSM972375 | 1690.079 | 2906.378 | 4596.457 | 0.287009 |
| GSE39582_GSM972376 | 1316.498 | 1673.617 | 2990.115 | 0.502852 |
| GSE39582_GSM972377 | 2624.723 | 4227.511 | 6852.234 | NA |
| GSE39582_GSM972378 | 867.0853 | 2375.169 | 3242.254 | 0.470523 |
| GSE39582_GSM972379 | -1111.91 | 2862.959 | 1751.046 | 0.650905 |
| GSE39582_GSM972380 | 2236.243 | 3932.08 | 6168.322 | 0.060334 |
| GSE39582_GSM972381 | 1191.724 | 1751.968 | 2943.691 | 0.50873 |
| GSE39582_GSM972382 | 122.8704 | 1912.034 | 2034.905 | 0.618717 |
| GSE39582_GSM972383 | -359.76 | 2364.011 | 2004.252 | 0.622246 |
| GSE39582_GSM972384 | 690.1316 | 1547.665 | 2237.797 | 0.595049 |
| GSE39582_GSM972385 | 2713.015 | 4951.182 | 7664.197 | NA |
| GSE39582_GSM972386 | -243.726 | 1796.332 | 1552.606 | 0.672739 |
| GSE39582_GSM972387 | -789.19 | 1657.974 | 868.7844 | 0.743495 |
| GSE39582_GSM972388 | -702.193 | 307.9965 | -394.196 | 0.854024 |
| GSE39582_GSM972389 | -190.811 | 2896.719 | 2705.908 | 0.538464 |
| GSE39582_GSM972390 | -54.2267 | 2186.65 | 2132.423 | 0.607408 |
| GSE39582_GSM972391 | 948.3826 | 3115.302 | 4063.685 | 0.36097 |
| GSE39582_GSM972392 | 196.7995 | 2359.156 | 2555.956 | 0.55688 |
| GSE39582_GSM972393 | 648.4065 | 2356.305 | 3004.712 | 0.500999 |
| GSE39582_GSM972394 | 1776.574 | 2819.75 | 4596.324 | 0.287027 |
| GSE39582_GSM972395 | 1008.16 | 2935.323 | 3943.483 | 0.377367 |
| GSE39582_GSM972396 | -1392.49 | 257.0774 | -1135.41 | 0.905465 |
| GSE39582_GSM972397 | -592.942 | 2650.823 | 2057.881 | 0.616064 |
| GSE39582_GSM972398 | -15.6981 | 3358.194 | 3342.496 | 0.457489 |
| GSE39582_GSM972399 | 443.2695 | 1719.983 | 2163.252 | 0.603807 |
| GSE39582_GSM972400 | 790.5764 | 2859.888 | 3650.465 | 0.416837 |
| GSE39582_GSM972401 | 3533.968 | 6173.34 | 9707.308 | NA |
| GSE39582_GSM972402 | 1878 | 2329.635 | 4207.635 | 0.341185 |
| GSE39582_GSM972403 | 614.1256 | 2935.651 | 3549.777 | 0.430226 |
| GSE39582_GSM972404 | 1015.262 | 2760.93 | 3776.192 | 0.399992 |
| GSE39582_GSM972405 | 1242.706 | 2913.877 | 4156.583 | 0.348219 |
| GSE39582_GSM972406 | 1883.953 | 4206.39 | 6090.343 | 0.071756 |
| GSE39582_GSM972407 | 1358.43 | 2145.231 | 3503.661 | 0.436327 |
| GSE39582_GSM972408 | 824.4476 | 3544.273 | 4368.72 | 0.318865 |
| GSE39582_GSM972409 | 4547.302 | 6293.647 | 10840.95 | NA |
| GSE39582_GSM972410 | 2709.329 | 3475.132 | 6184.461 | 0.057969 |
| GSE39582_GSM972411 | -488.994 | 1797.776 | 1308.782 | 0.698783 |
| GSE39582_GSM972412 | 670.4645 | 2512.467 | 3182.932 | 0.478189 |
| GSE39582_GSM972413 | 4353.016 | 4192.066 | 8545.082 | NA |
| GSE39582_GSM972414 | 308.2464 | 2105.62 | 2413.866 | 0.574081 |
| GSE39582_GSM972415 | -1116.31 | 1081.62 | -34.6895 | 0.825395 |
| GSE39582_GSM972416 | 1364.122 | 1519.204 | 2883.327 | 0.516339 |
| GSE39582_GSM972417 | 911.7267 | 2160.566 | 3072.293 | 0.492389 |
| GSE39582_GSM972418 | 500.6688 | 1702.893 | 2203.562 | 0.59908 |
| GSE39582_GSM972419 | 1431.133 | 1876.246 | 3307.379 | 0.462067 |
| GSE39582_GSM972420 | 1488.768 | 4466.474 | 5955.241 | 0.091521 |
| GSE39582_GSM972421 | 1851.399 | 2440.602 | 4292.001 | 0.329518 |
| GSE39582_GSM972422 | 3441.486 | 4828.151 | 8269.637 | NA |
| GSE39582_GSM972423 | 917.6684 | 2886.699 | 3804.367 | 0.396198 |
| GSE39582_GSM972424 | -367.868 | 1038.29 | 670.422 | 0.762649 |
| GSE39582_GSM972425 | 1816.586 | 2447.934 | 4264.52 | 0.333324 |
| GSE39582_GSM972426 | 1514.549 | 3902.131 | 5416.68 | 0.169875 |
| GSE39582_GSM972427 | 715.2894 | 1809.96 | 2525.25 | 0.560618 |
| GSE39582_GSM972428 | 1837.492 | 3065.535 | 4903.027 | 0.243625 |
| GSE39582_GSM972429 | -43.4841 | 3088.314 | 3044.83 | 0.495894 |
| GSE39582_GSM972430 | 1255.586 | 4737.323 | 5992.909 | 0.086013 |
| GSE39582_GSM972431 | 2001.184 | 4094.937 | 6096.121 | 0.07091 |
| GSE39582_GSM972432 | 1150.383 | 1912.754 | 3063.136 | 0.493558 |
| GSE39582_GSM972433 | 3832.676 | 4152.951 | 7985.627 | NA |
| GSE39582_GSM972434 | -745.501 | 2154.922 | 1409.421 | 0.68814 |
| GSE39582_GSM972435 | 610.0284 | 1690.405 | 2300.433 | 0.587635 |
| GSE39582_GSM972436 | 629.7348 | 2339.405 | 2969.139 | 0.505511 |
| GSE39582_GSM972437 | 2981.231 | 3660.504 | 6641.735 | NA |
| GSE39582_GSM972438 | 4046.808 | 4206.239 | 8253.047 | NA |
| GSE39582_GSM972439 | 1080.722 | 1556.529 | 2637.251 | 0.546929 |
| GSE39582_GSM972440 | 2576.168 | 4002.475 | 6578.643 | 0.000141 |
| GSE39582_GSM972441 | 2684.65 | 4795.485 | 7480.135 | NA |
| GSE39582_GSM972442 | 865.0977 | 4951.821 | 5816.919 | 0.111719 |
| GSE39582_GSM972443 | -487.192 | 2604.844 | 2117.652 | 0.609129 |
| GSE39582_GSM972444 | 2861.783 | 4417.763 | 7279.546 | NA |
| GSE39582_GSM972445 | 846.3167 | 3505.518 | 4351.835 | 0.321213 |
| GSE39582_GSM972446 | 944.2418 | 2235.396 | 3179.638 | 0.478614 |
| GSE39582_GSM972447 | 1141.83 | 3780.532 | 4922.362 | 0.240871 |
| GSE39582_GSM972448 | 310.3432 | 725.8131 | 1036.156 | 0.726843 |
| GSE39582_GSM972449 | 1354.078 | 2862.672 | 4216.75 | 0.339927 |
| GSE39582_GSM972450 | -207.04 | 2352.368 | 2145.328 | 0.605903 |
| GSE39582_GSM972451 | -770.054 | 1463.945 | 693.8917 | 0.760416 |
| GSE39582_GSM972452 | 2716.65 | 6018.781 | 8735.431 | NA |
| GSE39582_GSM972453 | -339.137 | 2340.184 | 2001.048 | 0.622614 |
| GSE39582_GSM972454 | 563.5482 | 3601.197 | 4164.745 | 0.347096 |
| GSE39582_GSM972455 | 3507.587 | 5155.817 | 8663.404 | NA |
| GSE39582_GSM972456 | 1184.159 | 1981.733 | 3165.893 | 0.480384 |
| GSE39582_GSM972457 | -526.717 | 2434.412 | 1907.695 | 0.633278 |
| GSE39582_GSM972458 | 1795.959 | 5472.982 | 7268.941 | NA |
| GSE39582_GSM972459 | -1.85853 | 1798.328 | 1796.469 | 0.645829 |
| GSE39582_GSM972460 | 1129.501 | 2339.572 | 3469.073 | 0.44089 |
| GSE39582_GSM972461 | 83.06645 | 1994.258 | 2077.325 | 0.613813 |
| GSE39582_GSM972462 | -13.3735 | 2035.291 | 2021.918 | 0.620214 |
| GSE39582_GSM972463 | -503.767 | 2237.083 | 1733.315 | 0.652879 |
| GSE39582_GSM972464 | 1855.706 | 3683.111 | 5538.816 | 0.152182 |
| GSE39582_GSM972465 | 210.9678 | 1197.802 | 1408.769 | 0.688209 |
| GSE39582_GSM972466 | -589.451 | 2040.18 | 1450.73 | 0.683728 |
| GSE39582_GSM972467 | 3876.506 | 3298.697 | 7175.203 | NA |
| GSE39582_GSM972468 | 714.1966 | 2548.481 | 3262.677 | 0.467876 |
| GSE39582_GSM972469 | 813.7404 | 2495.306 | 3309.046 | 0.46185 |
| GSE39582_GSM972470 | 4125.168 | 6783.726 | 10908.89 | NA |
| GSE39582_GSM972471 | 701.1678 | 3578.755 | 4279.923 | 0.331192 |
| GSE39582_GSM972472 | 702.5182 | 2044.278 | 2746.796 | 0.533397 |
| GSE39582_GSM972473 | 645.8533 | 3562.867 | 4208.72 | 0.341035 |
| GSE39582_GSM972474 | 1451.69 | 2831.254 | 4282.944 | 0.330773 |
| GSE39582_GSM972475 | -635.534 | 2488.633 | 1853.099 | 0.63946 |
| GSE39582_GSM972476 | 3962.115 | 3557.793 | 7519.908 | NA |
| GSE39582_GSM972477 | -1260.58 | 1780.338 | 519.7539 | 0.776767 |
| GSE39582_GSM972478 | 344.9885 | 2410.334 | 2755.323 | 0.532338 |
| GSE39582_GSM972479 | 5211.478 | 4284.759 | 9496.237 | NA |
| GSE39582_GSM972480 | -957.877 | 2018.512 | 1060.635 | 0.72437 |
| GSE39582_GSM972481 | 2342.638 | 2548.12 | 4890.759 | 0.245371 |
| GSE39582_GSM972482 | 1897.653 | 1879.071 | 3776.724 | 0.39992 |
| GSE39582_GSM972483 | 915.1068 | 1199.345 | 2114.452 | 0.609502 |
| GSE39582_GSM972484 | 1706.038 | 2457.781 | 4163.82 | 0.347223 |
| GSE39582_GSM972485 | 1190.727 | 2871.173 | 4061.899 | 0.361214 |
| GSE39582_GSM972486 | 1140.778 | 1786.48 | 2927.258 | 0.510806 |
| GSE39582_GSM972487 | -251.114 | 2443.761 | 2192.647 | 0.600362 |
| GSE39582_GSM972488 | -54.7006 | 3232.374 | 3177.673 | 0.478867 |
| GSE39582_GSM972489 | 600.7321 | 2631.015 | 3231.747 | 0.471884 |
| GSE39582_GSM972490 | 129.1655 | 1549.79 | 1678.955 | 0.658902 |
| GSE39582_GSM972491 | 789.9269 | 1608.747 | 2398.674 | 0.575906 |
| GSE39582_GSM972492 | 384.585 | 2578.761 | 2963.346 | 0.506244 |
| GSE39582_GSM972493 | 1523.082 | 2447.9 | 3970.982 | 0.373626 |
| GSE39582_GSM972494 | 1146.088 | 4007.892 | 5153.98 | 0.207741 |
| GSE39582_GSM972495 | -108.82 | 2092.081 | 1983.261 | 0.624655 |
| GSE39582_GSM972496 | -832.481 | 4098.365 | 3265.885 | 0.46746 |
| GSE39582_GSM972497 | 466.7327 | 1295.181 | 1761.914 | 0.649693 |
| GSE39582_GSM972498 | -314.803 | 1161.517 | 846.7142 | 0.745658 |
| GSE39582_GSM972499 | -208.21 | 2932.087 | 2723.877 | 0.53624 |
| GSE39582_GSM972500 | 2559.901 | 3703.371 | 6263.272 | 0.046417 |
| GSE39582_GSM972501 | 754.6279 | 3881.626 | 4636.254 | 0.281408 |
| GSE39582_GSM972502 | -1323.68 | 1165.815 | -157.861 | 0.835467 |
| GSE39582_GSM972503 | 2285.757 | 4435.262 | 6721.02 | NA |
| GSE39582_GSM972504 | 1687.301 | 2914.113 | 4601.415 | 0.286311 |
| GSE39582_GSM972505 | 373.2217 | 2895.582 | 3268.804 | 0.467081 |
| GSE39582_GSM972506 | 1501.659 | 3565.893 | 5067.552 | 0.220133 |
| GSE39582_GSM972507 | 594.374 | 3230.385 | 3824.759 | 0.393448 |
| GSE39582_GSM972508 | 318.9727 | 3171.722 | 3490.695 | 0.438039 |
| GSE39582_GSM972509 | -116.625 | 1922.192 | 1805.568 | 0.644809 |
| GSE39582_GSM972510 | -1089.17 | 1789.627 | 700.4534 | 0.75979 |
| GSE39582_GSM972511 | 681.2298 | 2035.265 | 2716.495 | 0.537154 |
| GSE39582_GSM972512 | -374.509 | 2453.26 | 2078.751 | 0.613648 |
| GSE39582_GSM972513 | 264.0617 | 1199.564 | 1463.626 | 0.682345 |
| GSE39582_GSM972514 | 453.0652 | 2478.649 | 2931.714 | 0.510243 |
| GSE39582_GSM972515 | -557.582 | 1394.802 | 837.2204 | 0.746586 |
| GSE39582_GSM972516 | -1019.58 | 1288.792 | 269.2071 | 0.799398 |
| GSE39582_GSM972517 | 1050.649 | 2234.922 | 3285.571 | 0.464903 |
| GSE39582_GSM972518 | 1442.211 | 3229.376 | 4671.587 | 0.276427 |
| GSE39582_GSM972519 | 1316.89 | 2600.375 | 3917.266 | 0.380928 |
| GSE39582_GSM972520 | 884.1006 | 3418.508 | 4302.608 | 0.328048 |
| GSE39582_GSM972521 | 2294.433 | 3515.512 | 5809.945 | 0.112736 |
| GSE39582_GSM972522 | 2364.47 | 5306.246 | 7670.716 | NA |

**Supplementary Table S6** | The CMS subgroup of TCGA and GEO samples by CMScaller package.

| ID | type |
| --- | --- |
| TCGA_TCGA-G4-6299 | CMS1 |
| TCGA_TCGA-AZ-4615 | CMS1 |
| TCGA_TCGA-A6-3809 | CMS1 |
| TCGA_TCGA-CM-4751 | CMS1 |
| TCGA_TCGA-AA-3949 | CMS1 |
| TCGA_TCGA-AA-3710 | CMS1 |
| TCGA_TCGA-CK-5916 | CMS1 |
| TCGA_TCGA-A6-2686 | CMS1 |
| TCGA_TCGA-CA-5255 | CMS1 |
| TCGA_TCGA-AA-A00R | CMS1 |
| TCGA_TCGA-AU-6004 | CMS1 |
| TCGA_TCGA-A6-2672 | CMS1 |
| TCGA_TCGA-DM-A1HB | CMS1 |
| TCGA_TCGA-AY-6197 | CMS1 |
| TCGA_TCGA-AA-3672 | CMS1 |
| TCGA_TCGA-AA-A00D | CMS1 |
| TCGA_TCGA-AZ-4614 | CMS1 |
| TCGA_TCGA-AZ-4315 | CMS1 |
| TCGA_TCGA-AA-3941 | CMS1 |
| TCGA_TCGA-G4-6309 | CMS1 |
| TCGA_TCGA-AA-3821 | CMS1 |
| TCGA_TCGA-CM-5861 | CMS1 |
| TCGA_TCGA-CM-4744 | CMS1 |
| TCGA_TCGA-AA-A010 | CMS1 |
| TCGA_TCGA-CK-4951 | CMS1 |
| TCGA_TCGA-AA-A02R | CMS1 |
| TCGA_TCGA-AA-3845 | CMS1 |
| TCGA_TCGA-AA-3815 | CMS1 |
| TCGA_TCGA-G4-6628 | CMS1 |
| TCGA_TCGA-AA-3833 | CMS1 |
| TCGA_TCGA-AA-A01P | CMS1 |
| TCGA_TCGA-D5-6530 | CMS1 |
| TCGA_TCGA-AA-3543 | CMS1 |
| TCGA_TCGA-D5-7000 | CMS1 |
| TCGA_TCGA-AM-5821 | CMS1 |
| TCGA_TCGA-AA-3966 | CMS1 |
| TCGA_TCGA-AA-3516 | CMS1 |
| TCGA_TCGA-A6-2676 | CMS1 |
| TCGA_TCGA-AD-6895 | CMS1 |
| TCGA_TCGA-A6-5661 | CMS1 |
| TCGA_TCGA-AA-3930 | CMS1 |
| TCGA_TCGA-AA-3947 | CMS1 |
| TCGA_TCGA-CM-6171 | CMS1 |
| TCGA_TCGA-CM-4743 | CMS1 |
| TCGA_TCGA-NH-A6GA | CMS1 |
| TCGA_TCGA-CM-6674 | CMS1 |
| TCGA_TCGA-AZ-4616 | CMS1 |
| TCGA_TCGA-AA-3877 | CMS1 |
| TCGA_TCGA-DM-A280 | CMS1 |
| TCGA_TCGA-D5-6531 | CMS1 |
| TCGA_TCGA-AD-6889 | CMS1 |
| TCGA_TCGA-AA-A01R | CMS1 |
| TCGA_TCGA-D5-6931 | CMS1 |
| TCGA_TCGA-A6-5665 | CMS1 |
| TCGA_TCGA-D5-6540 | CMS1 |
| TCGA_TCGA-CA-6718 | CMS1 |
| TCGA_TCGA-CK-6746 | CMS1 |
| TCGA_TCGA-CM-6675 | CMS1 |
| TCGA_TCGA-A6-6653 | CMS1 |
| TCGA_TCGA-F4-6570 | CMS1 |
| TCGA_TCGA-AA-A00J | CMS1 |
| TCGA_TCGA-AA-3554 | CMS1 |
| TCGA_TCGA-AZ-6606 | CMS1 |
| TCGA_TCGA-AA-3811 | CMS1 |
| TCGA_TCGA-AD-A5EJ | CMS1 |
| TCGA_TCGA-AA-A022 | CMS1 |
| TCGA_TCGA-AA-3713 | CMS1 |
| TCGA_TCGA-AZ-6601 | CMS1 |
| TCGA_TCGA-AA-A00E | CMS1 |
| TCGA_TCGA-D5-6927 | CMS1 |
| TCGA_TCGA-AA-3681 | CMS1 |
| TCGA_TCGA-AA-3492 | CMS1 |
| TCGA_TCGA-D5-6535 | CMS1 |
| TCGA_TCGA-AA-A029 | CMS1 |
| TCGA_TCGA-AA-3518 | CMS1 |
| TCGA_TCGA-G4-6586 | CMS1 |
| TCGA_TCGA-D5-6928 | CMS1 |
| TCGA_TCGA-AA-3664 | CMS1 |
| TCGA_TCGA-D5-6930 | CMS1 |
| TCGA_TCGA-CK-5913 | CMS1 |
| TCGA_TCGA-AD-5900 | CMS1 |
| TCGA_TCGA-G4-6588 | CMS1 |
| TCGA_TCGA-AA-3525 | CMS1 |
| TCGA_TCGA-F5-6814 | CMS1 |
| TCGA_TCGA-EI-6512 | CMS1 |
| TCGA_TCGA-AG-3600 | CMS1 |
| TCGA_TCGA-AG-3599 | CMS1 |
| TCGA_TCGA-AG-3902 | CMS1 |
| TCGA_TCGA-AG-3892 | CMS1 |
| TCGA_TCGA-G5-6233 | CMS1 |
| TCGA_TCGA-EI-6507 | CMS1 |
| GSE39582_GSM971963 | CMS1 |
| GSE39582_GSM971969 | CMS1 |
| GSE39582_GSM971973 | CMS1 |
| GSE39582_GSM971978 | CMS1 |
| GSE39582_GSM971979 | CMS1 |
| GSE39582_GSM971980 | CMS1 |
| GSE39582_GSM971981 | CMS1 |
| GSE39582_GSM971982 | CMS1 |
| GSE39582_GSM971983 | CMS1 |
| GSE39582_GSM971984 | CMS1 |
| GSE39582_GSM971988 | CMS1 |
| GSE39582_GSM971993 | CMS1 |
| GSE39582_GSM971997 | CMS1 |
| GSE39582_GSM972015 | CMS1 |
| GSE39582_GSM972016 | CMS1 |
| GSE39582_GSM972037 | CMS1 |
| GSE39582_GSM972056 | CMS1 |
| GSE39582_GSM972058 | CMS1 |
| GSE39582_GSM972059 | CMS1 |
| GSE39582_GSM972072 | CMS1 |
| GSE39582_GSM972076 | CMS1 |
| GSE39582_GSM972080 | CMS1 |
| GSE39582_GSM972083 | CMS1 |
| GSE39582_GSM972100 | CMS1 |
| GSE39582_GSM972103 | CMS1 |
| GSE39582_GSM972107 | CMS1 |
| GSE39582_GSM972109 | CMS1 |
| GSE39582_GSM972110 | CMS1 |
| GSE39582_GSM972117 | CMS1 |
| GSE39582_GSM972118 | CMS1 |
| GSE39582_GSM972168 | CMS1 |
| GSE39582_GSM972171 | CMS1 |
| GSE39582_GSM972180 | CMS1 |
| GSE39582_GSM972192 | CMS1 |
| GSE39582_GSM972196 | CMS1 |
| GSE39582_GSM972209 | CMS1 |
| GSE39582_GSM972214 | CMS1 |
| GSE39582_GSM972220 | CMS1 |
| GSE39582_GSM972221 | CMS1 |
| GSE39582_GSM972222 | CMS1 |
| GSE39582_GSM972223 | CMS1 |
| GSE39582_GSM972224 | CMS1 |
| GSE39582_GSM972228 | CMS1 |
| GSE39582_GSM972229 | CMS1 |
| GSE39582_GSM972241 | CMS1 |
| GSE39582_GSM972261 | CMS1 |
| GSE39582_GSM972263 | CMS1 |
| GSE39582_GSM972264 | CMS1 |
| GSE39582_GSM972265 | CMS1 |
| GSE39582_GSM972270 | CMS1 |
| GSE39582_GSM972274 | CMS1 |
| GSE39582_GSM972276 | CMS1 |
| GSE39582_GSM972277 | CMS1 |
| GSE39582_GSM972278 | CMS1 |
| GSE39582_GSM972280 | CMS1 |
| GSE39582_GSM972283 | CMS1 |
| GSE39582_GSM972286 | CMS1 |
| GSE39582_GSM972287 | CMS1 |
| GSE39582_GSM972292 | CMS1 |
| GSE39582_GSM972294 | CMS1 |
| GSE39582_GSM972298 | CMS1 |
| GSE39582_GSM972305 | CMS1 |
| GSE39582_GSM972314 | CMS1 |
| GSE39582_GSM972316 | CMS1 |
| GSE39582_GSM972317 | CMS1 |
| GSE39582_GSM972325 | CMS1 |
| GSE39582_GSM972330 | CMS1 |
| GSE39582_GSM972332 | CMS1 |
| GSE39582_GSM972334 | CMS1 |
| GSE39582_GSM972337 | CMS1 |
| GSE39582_GSM972341 | CMS1 |
| GSE39582_GSM972346 | CMS1 |
| GSE39582_GSM972353 | CMS1 |
| GSE39582_GSM972354 | CMS1 |
| GSE39582_GSM972390 | CMS1 |
| GSE39582_GSM972391 | CMS1 |
| GSE39582_GSM972403 | CMS1 |
| GSE39582_GSM972406 | CMS1 |
| GSE39582_GSM972408 | CMS1 |
| GSE39582_GSM972420 | CMS1 |
| GSE39582_GSM972430 | CMS1 |
| GSE39582_GSM972445 | CMS1 |
| GSE39582_GSM972447 | CMS1 |
| GSE39582_GSM972448 | CMS1 |
| GSE39582_GSM972452 | CMS1 |
| GSE39582_GSM972458 | CMS1 |
| GSE39582_GSM972465 | CMS1 |
| GSE39582_GSM972471 | CMS1 |
| GSE39582_GSM972477 | CMS1 |
| GSE39582_GSM972494 | CMS1 |
| GSE39582_GSM972499 | CMS1 |
| GSE39582_GSM972507 | CMS1 |
| GSE39582_GSM972520 | CMS1 |
| GSE39582_GSM972522 | CMS1 |
| TCGA_TCGA-CM-6164 | CMS2 |
| TCGA_TCGA-AY-4071 | CMS2 |
| TCGA_TCGA-CM-4752 | CMS2 |
| TCGA_TCGA-AA-3688 | CMS2 |
| TCGA_TCGA-AA-3494 | CMS2 |
| TCGA_TCGA-CM-4750 | CMS2 |
| TCGA_TCGA-AZ-4682 | CMS2 |
| TCGA_TCGA-DM-A0XF | CMS2 |
| TCGA_TCGA-AA-3529 | CMS2 |
| TCGA_TCGA-AA-3848 | CMS2 |
| TCGA_TCGA-CA-6715 | CMS2 |
| TCGA_TCGA-AA-3506 | CMS2 |
| TCGA_TCGA-AD-6890 | CMS2 |
| TCGA_TCGA-A6-2677 | CMS2 |
| TCGA_TCGA-AA-3562 | CMS2 |
| TCGA_TCGA-AA-3970 | CMS2 |
| TCGA_TCGA-NH-A8F7 | CMS2 |
| TCGA_TCGA-AA-3524 | CMS2 |
| TCGA_TCGA-AA-3697 | CMS2 |
| TCGA_TCGA-AA-3971 | CMS2 |
| TCGA_TCGA-AA-3989 | CMS2 |
| TCGA_TCGA-A6-2679 | CMS2 |
| TCGA_TCGA-AA-3488 | CMS2 |
| TCGA_TCGA-G4-6293 | CMS2 |
| TCGA_TCGA-AA-3517 | CMS2 |
| TCGA_TCGA-AA-3678 | CMS2 |
| TCGA_TCGA-AA-A01X | CMS2 |
| TCGA_TCGA-AA-3530 | CMS2 |
| TCGA_TCGA-AA-A02J | CMS2 |
| TCGA_TCGA-CA-5256 | CMS2 |
| TCGA_TCGA-AA-3521 | CMS2 |
| TCGA_TCGA-CM-6166 | CMS2 |
| TCGA_TCGA-AA-3856 | CMS2 |
| TCGA_TCGA-AA-A00Q | CMS2 |
| TCGA_TCGA-AD-6888 | CMS2 |
| TCGA_TCGA-AA-3660 | CMS2 |
| TCGA_TCGA-AA-A00Z | CMS2 |
| TCGA_TCGA-AA-3679 | CMS2 |
| TCGA_TCGA-AA-3855 | CMS2 |
| TCGA_TCGA-AA-3519 | CMS2 |
| TCGA_TCGA-AA-3526 | CMS2 |
| TCGA_TCGA-AA-3534 | CMS2 |
| TCGA_TCGA-G4-6317 | CMS2 |
| TCGA_TCGA-AA-A03J | CMS2 |
| TCGA_TCGA-AA-3869 | CMS2 |
| TCGA_TCGA-DM-A282 | CMS2 |
| TCGA_TCGA-AA-3846 | CMS2 |
| TCGA_TCGA-AA-3956 | CMS2 |
| TCGA_TCGA-AA-3955 | CMS2 |
| TCGA_TCGA-AA-A02F | CMS2 |
| TCGA_TCGA-A6-5656 | CMS2 |
| TCGA_TCGA-AA-A017 | CMS2 |
| TCGA_TCGA-AA-A00L | CMS2 |
| TCGA_TCGA-CM-5864 | CMS2 |
| TCGA_TCGA-AD-6965 | CMS2 |
| TCGA_TCGA-5M-AAT5 | CMS2 |
| TCGA_TCGA-AA-3858 | CMS2 |
| TCGA_TCGA-AA-3662 | CMS2 |
| TCGA_TCGA-AA-3975 | CMS2 |
| TCGA_TCGA-AA-3986 | CMS2 |
| TCGA_TCGA-AA-3831 | CMS2 |
| TCGA_TCGA-AA-A02H | CMS2 |
| TCGA_TCGA-AA-3685 | CMS2 |
| TCGA_TCGA-AA-3538 | CMS2 |
| TCGA_TCGA-AA-3509 | CMS2 |
| TCGA_TCGA-AA-3542 | CMS2 |
| TCGA_TCGA-A6-6140 | CMS2 |
| TCGA_TCGA-AY-A8YK | CMS2 |
| TCGA_TCGA-AA-3667 | CMS2 |
| TCGA_TCGA-A6-6137 | CMS2 |
| TCGA_TCGA-AA-3495 | CMS2 |
| TCGA_TCGA-AA-3976 | CMS2 |
| TCGA_TCGA-AA-3819 | CMS2 |
| TCGA_TCGA-AA-3875 | CMS2 |
| TCGA_TCGA-A6-6648 | CMS2 |
| TCGA_TCGA-A6-5660 | CMS2 |
| TCGA_TCGA-5M-AATE | CMS2 |
| TCGA_TCGA-CM-6161 | CMS2 |
| TCGA_TCGA-F4-6808 | CMS2 |
| TCGA_TCGA-AA-A02W | CMS2 |
| TCGA_TCGA-NH-A50T | CMS2 |
| TCGA_TCGA-AA-3972 | CMS2 |
| TCGA_TCGA-G4-6315 | CMS2 |
| TCGA_TCGA-D5-5537 | CMS2 |
| TCGA_TCGA-AA-3552 | CMS2 |
| TCGA_TCGA-AA-3693 | CMS2 |
| TCGA_TCGA-A6-2680 | CMS2 |
| TCGA_TCGA-QG-A5YV | CMS2 |
| TCGA_TCGA-AA-3531 | CMS2 |
| TCGA_TCGA-AG-3582 | CMS2 |
| TCGA_TCGA-DY-A1DD | CMS2 |
| TCGA_TCGA-AG-A01J | CMS2 |
| TCGA_TCGA-AG-4015 | CMS2 |
| TCGA_TCGA-AG-A011 | CMS2 |
| TCGA_TCGA-AG-A032 | CMS2 |
| TCGA_TCGA-EI-6883 | CMS2 |
| TCGA_TCGA-F5-6861 | CMS2 |
| TCGA_TCGA-AG-3581 | CMS2 |
| TCGA_TCGA-AG-3586 | CMS2 |
| TCGA_TCGA-AG-3909 | CMS2 |
| TCGA_TCGA-EI-6513 | CMS2 |
| TCGA_TCGA-AG-3601 | CMS2 |
| TCGA_TCGA-CI-6622 | CMS2 |
| TCGA_TCGA-AG-A036 | CMS2 |
| TCGA_TCGA-AG-3593 | CMS2 |
| TCGA_TCGA-AG-3742 | CMS2 |
| TCGA_TCGA-DC-6683 | CMS2 |
| TCGA_TCGA-CL-5918 | CMS2 |
| TCGA_TCGA-AG-A01N | CMS2 |
| TCGA_TCGA-AG-3608 | CMS2 |
| TCGA_TCGA-AG-3587 | CMS2 |
| TCGA_TCGA-AG-3894 | CMS2 |
| TCGA_TCGA-AG-A016 | CMS2 |
| TCGA_TCGA-DC-6157 | CMS2 |
| TCGA_TCGA-EI-6881 | CMS2 |
| TCGA_TCGA-CL-4957 | CMS2 |
| TCGA_TCGA-DC-4749 | CMS2 |
| TCGA_TCGA-EF-5830 | CMS2 |
| TCGA_TCGA-AF-A56L | CMS2 |
| TCGA_TCGA-AG-3574 | CMS2 |
| TCGA_TCGA-G5-6235 | CMS2 |
| TCGA_TCGA-AG-3890 | CMS2 |
| TCGA_TCGA-AG-3882 | CMS2 |
| GSE39582_GSM971960 | CMS2 |
| GSE39582_GSM971964 | CMS2 |
| GSE39582_GSM971972 | CMS2 |
| GSE39582_GSM971975 | CMS2 |
| GSE39582_GSM971976 | CMS2 |
| GSE39582_GSM971992 | CMS2 |
| GSE39582_GSM971994 | CMS2 |
| GSE39582_GSM972000 | CMS2 |
| GSE39582_GSM972003 | CMS2 |
| GSE39582_GSM972004 | CMS2 |
| GSE39582_GSM972013 | CMS2 |
| GSE39582_GSM972014 | CMS2 |
| GSE39582_GSM972017 | CMS2 |
| GSE39582_GSM972018 | CMS2 |
| GSE39582_GSM972022 | CMS2 |
| GSE39582_GSM972024 | CMS2 |
| GSE39582_GSM972027 | CMS2 |
| GSE39582_GSM972028 | CMS2 |
| GSE39582_GSM972031 | CMS2 |
| GSE39582_GSM972032 | CMS2 |
| GSE39582_GSM972033 | CMS2 |
| GSE39582_GSM972035 | CMS2 |
| GSE39582_GSM972038 | CMS2 |
| GSE39582_GSM972040 | CMS2 |
| GSE39582_GSM972041 | CMS2 |
| GSE39582_GSM972043 | CMS2 |
| GSE39582_GSM972048 | CMS2 |
| GSE39582_GSM972052 | CMS2 |
| GSE39582_GSM972057 | CMS2 |
| GSE39582_GSM972061 | CMS2 |
| GSE39582_GSM972065 | CMS2 |
| GSE39582_GSM972066 | CMS2 |
| GSE39582_GSM972069 | CMS2 |
| GSE39582_GSM972071 | CMS2 |
| GSE39582_GSM972074 | CMS2 |
| GSE39582_GSM972081 | CMS2 |
| GSE39582_GSM972087 | CMS2 |
| GSE39582_GSM972090 | CMS2 |
| GSE39582_GSM972096 | CMS2 |
| GSE39582_GSM972099 | CMS2 |
| GSE39582_GSM972105 | CMS2 |
| GSE39582_GSM972108 | CMS2 |
| GSE39582_GSM972115 | CMS2 |
| GSE39582_GSM972119 | CMS2 |
| GSE39582_GSM972123 | CMS2 |
| GSE39582_GSM972124 | CMS2 |
| GSE39582_GSM972126 | CMS2 |
| GSE39582_GSM972130 | CMS2 |
| GSE39582_GSM972136 | CMS2 |
| GSE39582_GSM972137 | CMS2 |
| GSE39582_GSM972139 | CMS2 |
| GSE39582_GSM972142 | CMS2 |
| GSE39582_GSM972143 | CMS2 |
| GSE39582_GSM972144 | CMS2 |
| GSE39582_GSM972147 | CMS2 |
| GSE39582_GSM972148 | CMS2 |
| GSE39582_GSM972149 | CMS2 |
| GSE39582_GSM972150 | CMS2 |
| GSE39582_GSM972151 | CMS2 |
| GSE39582_GSM972152 | CMS2 |
| GSE39582_GSM972153 | CMS2 |
| GSE39582_GSM972156 | CMS2 |
| GSE39582_GSM972160 | CMS2 |
| GSE39582_GSM972167 | CMS2 |
| GSE39582_GSM972169 | CMS2 |
| GSE39582_GSM972174 | CMS2 |
| GSE39582_GSM972175 | CMS2 |
| GSE39582_GSM972177 | CMS2 |
| GSE39582_GSM972182 | CMS2 |
| GSE39582_GSM972185 | CMS2 |
| GSE39582_GSM972188 | CMS2 |
| GSE39582_GSM972190 | CMS2 |
| GSE39582_GSM972193 | CMS2 |
| GSE39582_GSM972194 | CMS2 |
| GSE39582_GSM972198 | CMS2 |
| GSE39582_GSM972199 | CMS2 |
| GSE39582_GSM972200 | CMS2 |
| GSE39582_GSM972202 | CMS2 |
| GSE39582_GSM972203 | CMS2 |
| GSE39582_GSM972211 | CMS2 |
| GSE39582_GSM972216 | CMS2 |
| GSE39582_GSM972217 | CMS2 |
| GSE39582_GSM972219 | CMS2 |
| GSE39582_GSM972230 | CMS2 |
| GSE39582_GSM972236 | CMS2 |
| GSE39582_GSM972243 | CMS2 |
| GSE39582_GSM972244 | CMS2 |
| GSE39582_GSM972247 | CMS2 |
| GSE39582_GSM972248 | CMS2 |
| GSE39582_GSM972249 | CMS2 |
| GSE39582_GSM972250 | CMS2 |
| GSE39582_GSM972256 | CMS2 |
| GSE39582_GSM972258 | CMS2 |
| GSE39582_GSM972260 | CMS2 |
| GSE39582_GSM972267 | CMS2 |
| GSE39582_GSM972271 | CMS2 |
| GSE39582_GSM972281 | CMS2 |
| GSE39582_GSM972288 | CMS2 |
| GSE39582_GSM972289 | CMS2 |
| GSE39582_GSM972303 | CMS2 |
| GSE39582_GSM972306 | CMS2 |
| GSE39582_GSM972309 | CMS2 |
| GSE39582_GSM972312 | CMS2 |
| GSE39582_GSM972319 | CMS2 |
| GSE39582_GSM972320 | CMS2 |
| GSE39582_GSM972321 | CMS2 |
| GSE39582_GSM972322 | CMS2 |
| GSE39582_GSM972323 | CMS2 |
| GSE39582_GSM972324 | CMS2 |
| GSE39582_GSM972331 | CMS2 |
| GSE39582_GSM972333 | CMS2 |
| GSE39582_GSM972335 | CMS2 |
| GSE39582_GSM972336 | CMS2 |
| GSE39582_GSM972338 | CMS2 |
| GSE39582_GSM972340 | CMS2 |
| GSE39582_GSM972342 | CMS2 |
| GSE39582_GSM972347 | CMS2 |
| GSE39582_GSM972351 | CMS2 |
| GSE39582_GSM972356 | CMS2 |
| GSE39582_GSM972358 | CMS2 |
| GSE39582_GSM972362 | CMS2 |
| GSE39582_GSM972363 | CMS2 |
| GSE39582_GSM972368 | CMS2 |
| GSE39582_GSM972370 | CMS2 |
| GSE39582_GSM972371 | CMS2 |
| GSE39582_GSM972372 | CMS2 |
| GSE39582_GSM972373 | CMS2 |
| GSE39582_GSM972374 | CMS2 |
| GSE39582_GSM972381 | CMS2 |
| GSE39582_GSM972382 | CMS2 |
| GSE39582_GSM972383 | CMS2 |
| GSE39582_GSM972387 | CMS2 |
| GSE39582_GSM972388 | CMS2 |
| GSE39582_GSM972392 | CMS2 |
| GSE39582_GSM972395 | CMS2 |
| GSE39582_GSM972399 | CMS2 |
| GSE39582_GSM972404 | CMS2 |
| GSE39582_GSM972405 | CMS2 |
| GSE39582_GSM972411 | CMS2 |
| GSE39582_GSM972414 | CMS2 |
| GSE39582_GSM972415 | CMS2 |
| GSE39582_GSM972418 | CMS2 |
| GSE39582_GSM972423 | CMS2 |
| GSE39582_GSM972424 | CMS2 |
| GSE39582_GSM972434 | CMS2 |
| GSE39582_GSM972435 | CMS2 |
| GSE39582_GSM972450 | CMS2 |
| GSE39582_GSM972451 | CMS2 |
| GSE39582_GSM972454 | CMS2 |
| GSE39582_GSM972461 | CMS2 |
| GSE39582_GSM972462 | CMS2 |
| GSE39582_GSM972468 | CMS2 |
| GSE39582_GSM972469 | CMS2 |
| GSE39582_GSM972478 | CMS2 |
| GSE39582_GSM972485 | CMS2 |
| GSE39582_GSM972490 | CMS2 |
| GSE39582_GSM972492 | CMS2 |
| GSE39582_GSM972495 | CMS2 |
| GSE39582_GSM972497 | CMS2 |
| GSE39582_GSM972498 | CMS2 |
| GSE39582_GSM972505 | CMS2 |
| GSE39582_GSM972508 | CMS2 |
| GSE39582_GSM972509 | CMS2 |
| GSE39582_GSM972511 | CMS2 |
| GSE39582_GSM972513 | CMS2 |
| GSE39582_GSM972514 | CMS2 |
| TCGA_TCGA-AA-3854 | CMS3 |
| TCGA_TCGA-AA-3818 | CMS3 |
| TCGA_TCGA-AA-A00K | CMS3 |
| TCGA_TCGA-NH-A6GC | CMS3 |
| TCGA_TCGA-A6-6141 | CMS3 |
| TCGA_TCGA-CK-6747 | CMS3 |
| TCGA_TCGA-QG-A5Z2 | CMS3 |
| TCGA_TCGA-G4-6321 | CMS3 |
| TCGA_TCGA-D5-6539 | CMS3 |
| TCGA_TCGA-F4-6806 | CMS3 |
| TCGA_TCGA-AY-A71X | CMS3 |
| TCGA_TCGA-DM-A28K | CMS3 |
| TCGA_TCGA-AY-6386 | CMS3 |
| TCGA_TCGA-DM-A28G | CMS3 |
| TCGA_TCGA-D5-5539 | CMS3 |
| TCGA_TCGA-AA-3837 | CMS3 |
| TCGA_TCGA-AA-A02O | CMS3 |
| TCGA_TCGA-D5-6920 | CMS3 |
| TCGA_TCGA-DM-A1D6 | CMS3 |
| TCGA_TCGA-AA-3939 | CMS3 |
| TCGA_TCGA-CK-4952 | CMS3 |
| TCGA_TCGA-AA-A01V | CMS3 |
| TCGA_TCGA-CM-6680 | CMS3 |
| TCGA_TCGA-AA-3663 | CMS3 |
| TCGA_TCGA-AZ-5407 | CMS3 |
| TCGA_TCGA-AA-3675 | CMS3 |
| TCGA_TCGA-AA-3556 | CMS3 |
| TCGA_TCGA-CM-6678 | CMS3 |
| TCGA_TCGA-AA-3851 | CMS3 |
| TCGA_TCGA-AA-3861 | CMS3 |
| TCGA_TCGA-AA-3522 | CMS3 |
| TCGA_TCGA-AZ-6599 | CMS3 |
| TCGA_TCGA-AA-3994 | CMS3 |
| TCGA_TCGA-AA-3696 | CMS3 |
| TCGA_TCGA-G4-6323 | CMS3 |
| TCGA_TCGA-AA-3548 | CMS3 |
| TCGA_TCGA-F4-6856 | CMS3 |
| TCGA_TCGA-AA-3680 | CMS3 |
| TCGA_TCGA-AA-A024 | CMS3 |
| TCGA_TCGA-4T-AA8H | CMS3 |
| TCGA_TCGA-AA-3655 | CMS3 |
| TCGA_TCGA-AA-3673 | CMS3 |
| TCGA_TCGA-CK-4950 | CMS3 |
| TCGA_TCGA-AA-3852 | CMS3 |
| TCGA_TCGA-G4-6322 | CMS3 |
| TCGA_TCGA-G4-6320 | CMS3 |
| TCGA_TCGA-AA-3864 | CMS3 |
| TCGA_TCGA-A6-2678 | CMS3 |
| TCGA_TCGA-CA-5796 | CMS3 |
| TCGA_TCGA-AZ-4313 | CMS3 |
| TCGA_TCGA-AA-3850 | CMS3 |
| TCGA_TCGA-AY-A69D | CMS3 |
| TCGA_TCGA-AA-3692 | CMS3 |
| TCGA_TCGA-AA-3560 | CMS3 |
| TCGA_TCGA-CM-6172 | CMS3 |
| TCGA_TCGA-AA-3977 | CMS3 |
| TCGA_TCGA-AA-A03F | CMS3 |
| TCGA_TCGA-AA-3502 | CMS3 |
| TCGA_TCGA-CM-4746 | CMS3 |
| TCGA_TCGA-AA-3555 | CMS3 |
| TCGA_TCGA-AG-3611 | CMS3 |
| TCGA_TCGA-AG-A02N | CMS3 |
| TCGA_TCGA-AH-6903 | CMS3 |
| TCGA_TCGA-AG-3887 | CMS3 |
| TCGA_TCGA-AG-A020 | CMS3 |
| TCGA_TCGA-AF-6136 | CMS3 |
| TCGA_TCGA-AF-6672 | CMS3 |
| TCGA_TCGA-AG-3896 | CMS3 |
| TCGA_TCGA-G5-6641 | CMS3 |
| TCGA_TCGA-AF-2691 | CMS3 |
| TCGA_TCGA-AG-3594 | CMS3 |
| TCGA_TCGA-EI-6510 | CMS3 |
| TCGA_TCGA-AG-A02X | CMS3 |
| TCGA_TCGA-AG-3732 | CMS3 |
| TCGA_TCGA-EI-6882 | CMS3 |
| TCGA_TCGA-AG-3591 | CMS3 |
| TCGA_TCGA-AG-A008 | CMS3 |
| TCGA_TCGA-AG-A015 | CMS3 |
| TCGA_TCGA-AG-3578 | CMS3 |
| TCGA_TCGA-AG-3583 | CMS3 |
| TCGA_TCGA-EI-6506 | CMS3 |
| TCGA_TCGA-AF-2692 | CMS3 |
| GSE39582_GSM971965 | CMS3 |
| GSE39582_GSM971967 | CMS3 |
| GSE39582_GSM971970 | CMS3 |
| GSE39582_GSM971971 | CMS3 |
| GSE39582_GSM971974 | CMS3 |
| GSE39582_GSM971985 | CMS3 |
| GSE39582_GSM971989 | CMS3 |
| GSE39582_GSM971995 | CMS3 |
| GSE39582_GSM971998 | CMS3 |
| GSE39582_GSM972002 | CMS3 |
| GSE39582_GSM972005 | CMS3 |
| GSE39582_GSM972012 | CMS3 |
| GSE39582_GSM972020 | CMS3 |
| GSE39582_GSM972023 | CMS3 |
| GSE39582_GSM972025 | CMS3 |
| GSE39582_GSM972036 | CMS3 |
| GSE39582_GSM972042 | CMS3 |
| GSE39582_GSM972044 | CMS3 |
| GSE39582_GSM972045 | CMS3 |
| GSE39582_GSM972049 | CMS3 |
| GSE39582_GSM972050 | CMS3 |
| GSE39582_GSM972062 | CMS3 |
| GSE39582_GSM972070 | CMS3 |
| GSE39582_GSM972091 | CMS3 |
| GSE39582_GSM972098 | CMS3 |
| GSE39582_GSM972101 | CMS3 |
| GSE39582_GSM972111 | CMS3 |
| GSE39582_GSM972114 | CMS3 |
| GSE39582_GSM972121 | CMS3 |
| GSE39582_GSM972129 | CMS3 |
| GSE39582_GSM972134 | CMS3 |
| GSE39582_GSM972135 | CMS3 |
| GSE39582_GSM972145 | CMS3 |
| GSE39582_GSM972146 | CMS3 |
| GSE39582_GSM972155 | CMS3 |
| GSE39582_GSM972159 | CMS3 |
| GSE39582_GSM972163 | CMS3 |
| GSE39582_GSM972166 | CMS3 |
| GSE39582_GSM972178 | CMS3 |
| GSE39582_GSM972183 | CMS3 |
| GSE39582_GSM972191 | CMS3 |
| GSE39582_GSM972197 | CMS3 |
| GSE39582_GSM972212 | CMS3 |
| GSE39582_GSM972218 | CMS3 |
| GSE39582_GSM972226 | CMS3 |
| GSE39582_GSM972227 | CMS3 |
| GSE39582_GSM972259 | CMS3 |
| GSE39582_GSM972268 | CMS3 |
| GSE39582_GSM972273 | CMS3 |
| GSE39582_GSM972285 | CMS3 |
| GSE39582_GSM972296 | CMS3 |
| GSE39582_GSM972300 | CMS3 |
| GSE39582_GSM972302 | CMS3 |
| GSE39582_GSM972304 | CMS3 |
| GSE39582_GSM972310 | CMS3 |
| GSE39582_GSM972315 | CMS3 |
| GSE39582_GSM972326 | CMS3 |
| GSE39582_GSM972329 | CMS3 |
| GSE39582_GSM972350 | CMS3 |
| GSE39582_GSM972357 | CMS3 |
| GSE39582_GSM972364 | CMS3 |
| GSE39582_GSM972379 | CMS3 |
| GSE39582_GSM972389 | CMS3 |
| GSE39582_GSM972396 | CMS3 |
| GSE39582_GSM972400 | CMS3 |
| GSE39582_GSM972429 | CMS3 |
| GSE39582_GSM972453 | CMS3 |
| GSE39582_GSM972457 | CMS3 |
| GSE39582_GSM972459 | CMS3 |
| GSE39582_GSM972463 | CMS3 |
| GSE39582_GSM972472 | CMS3 |
| GSE39582_GSM972473 | CMS3 |
| GSE39582_GSM972475 | CMS3 |
| GSE39582_GSM972480 | CMS3 |
| GSE39582_GSM972482 | CMS3 |
| GSE39582_GSM972487 | CMS3 |
| GSE39582_GSM972488 | CMS3 |
| GSE39582_GSM972489 | CMS3 |
| GSE39582_GSM972502 | CMS3 |
| GSE39582_GSM972510 | CMS3 |
| GSE39582_GSM972512 | CMS3 |
| TCGA_TCGA-F4-6463 | CMS4 |
| TCGA_TCGA-CM-6165 | CMS4 |
| TCGA_TCGA-G4-6625 | CMS4 |
| TCGA_TCGA-AA-3950 | CMS4 |
| TCGA_TCGA-AA-A00N | CMS4 |
| TCGA_TCGA-A6-6138 | CMS4 |
| TCGA_TCGA-AA-3715 | CMS4 |
| TCGA_TCGA-AU-3779 | CMS4 |
| TCGA_TCGA-QG-A5Z1 | CMS4 |
| TCGA_TCGA-A6-2685 | CMS4 |
| TCGA_TCGA-A6-A5ZU | CMS4 |
| TCGA_TCGA-NH-A50V | CMS4 |
| TCGA_TCGA-CA-6717 | CMS4 |
| TCGA_TCGA-A6-6649 | CMS4 |
| TCGA_TCGA-A6-4105 | CMS4 |
| TCGA_TCGA-AZ-4684 | CMS4 |
| TCGA_TCGA-F4-6459 | CMS4 |
| TCGA_TCGA-AY-6196 | CMS4 |
| TCGA_TCGA-CM-6679 | CMS4 |
| TCGA_TCGA-AA-3684 | CMS4 |
| TCGA_TCGA-A6-2684 | CMS4 |
| TCGA_TCGA-AZ-4323 | CMS4 |
| TCGA_TCGA-AD-6548 | CMS4 |
| TCGA_TCGA-G4-6302 | CMS4 |
| TCGA_TCGA-A6-2674 | CMS4 |
| TCGA_TCGA-A6-5664 | CMS4 |
| TCGA_TCGA-A6-5657 | CMS4 |
| TCGA_TCGA-CM-5344 | CMS4 |
| TCGA_TCGA-CM-6677 | CMS4 |
| TCGA_TCGA-D5-6923 | CMS4 |
| TCGA_TCGA-F4-6703 | CMS4 |
| TCGA_TCGA-AZ-6605 | CMS4 |
| TCGA_TCGA-CM-5349 | CMS4 |
| TCGA_TCGA-G4-6627 | CMS4 |
| TCGA_TCGA-CM-6163 | CMS4 |
| TCGA_TCGA-AA-A004 | CMS4 |
| TCGA_TCGA-A6-3808 | CMS4 |
| TCGA_TCGA-CK-4947 | CMS4 |
| TCGA_TCGA-CM-6167 | CMS4 |
| TCGA_TCGA-CA-5797 | CMS4 |
| TCGA_TCGA-CA-6719 | CMS4 |
| TCGA_TCGA-F4-6704 | CMS4 |
| TCGA_TCGA-A6-6781 | CMS4 |
| TCGA_TCGA-AA-A00F | CMS4 |
| TCGA_TCGA-D5-5538 | CMS4 |
| TCGA_TCGA-AA-3867 | CMS4 |
| TCGA_TCGA-A6-6782 | CMS4 |
| TCGA_TCGA-A6-6654 | CMS4 |
| TCGA_TCGA-AA-A01D | CMS4 |
| TCGA_TCGA-G4-6311 | CMS4 |
| TCGA_TCGA-A6-3807 | CMS4 |
| TCGA_TCGA-AZ-5403 | CMS4 |
| TCGA_TCGA-CM-5341 | CMS4 |
| TCGA_TCGA-AA-3514 | CMS4 |
| TCGA_TCGA-CM-5863 | CMS4 |
| TCGA_TCGA-DM-A285 | CMS4 |
| TCGA_TCGA-A6-4107 | CMS4 |
| TCGA_TCGA-D5-6541 | CMS4 |
| TCGA_TCGA-AA-3870 | CMS4 |
| TCGA_TCGA-CM-6169 | CMS4 |
| TCGA_TCGA-D5-5541 | CMS4 |
| TCGA_TCGA-CM-5868 | CMS4 |
| TCGA_TCGA-D5-6926 | CMS4 |
| TCGA_TCGA-AA-A01K | CMS4 |
| TCGA_TCGA-AA-3984 | CMS4 |
| TCGA_TCGA-D5-6536 | CMS4 |
| TCGA_TCGA-AZ-6607 | CMS4 |
| TCGA_TCGA-AA-3520 | CMS4 |
| TCGA_TCGA-G4-6314 | CMS4 |
| TCGA_TCGA-CM-5348 | CMS4 |
| TCGA_TCGA-CK-6751 | CMS4 |
| TCGA_TCGA-D5-6929 | CMS4 |
| TCGA_TCGA-A6-2681 | CMS4 |
| TCGA_TCGA-G4-6298 | CMS4 |
| TCGA_TCGA-NH-A8F8 | CMS4 |
| TCGA_TCGA-AA-3544 | CMS4 |
| TCGA_TCGA-AA-3532 | CMS4 |
| TCGA_TCGA-CM-5860 | CMS4 |
| TCGA_TCGA-CM-6162 | CMS4 |
| TCGA_TCGA-F4-6807 | CMS4 |
| TCGA_TCGA-AA-3841 | CMS4 |
| TCGA_TCGA-AA-3814 | CMS4 |
| TCGA_TCGA-A6-2675 | CMS4 |
| TCGA_TCGA-G4-6297 | CMS4 |
| TCGA_TCGA-AA-3553 | CMS4 |
| TCGA_TCGA-AA-3511 | CMS4 |
| TCGA_TCGA-A6-A56B | CMS4 |
| TCGA_TCGA-A6-5667 | CMS4 |
| TCGA_TCGA-AA-3866 | CMS4 |
| TCGA_TCGA-CM-6170 | CMS4 |
| TCGA_TCGA-WS-AB45 | CMS4 |
| TCGA_TCGA-D5-6898 | CMS4 |
| TCGA_TCGA-AA-A00O | CMS4 |
| TCGA_TCGA-CK-4948 | CMS4 |
| TCGA_TCGA-F4-6809 | CMS4 |
| TCGA_TCGA-AM-5820 | CMS4 |
| TCGA_TCGA-G4-6310 | CMS4 |
| TCGA_TCGA-AA-3872 | CMS4 |
| TCGA_TCGA-5M-AAT6 | CMS4 |
| TCGA_TCGA-D5-6924 | CMS4 |
| TCGA_TCGA-AA-3860 | CMS4 |
| TCGA_TCGA-A6-A566 | CMS4 |
| TCGA_TCGA-AA-3712 | CMS4 |
| TCGA_TCGA-A6-5662 | CMS4 |
| TCGA_TCGA-CM-6676 | CMS4 |
| TCGA_TCGA-AD-6899 | CMS4 |
| TCGA_TCGA-F4-6569 | CMS4 |
| TCGA_TCGA-F4-6461 | CMS4 |
| TCGA_TCGA-F4-6854 | CMS4 |
| TCGA_TCGA-AA-3842 | CMS4 |
| TCGA_TCGA-AZ-4308 | CMS4 |
| TCGA_TCGA-AD-6964 | CMS4 |
| TCGA_TCGA-A6-2682 | CMS4 |
| TCGA_TCGA-F4-6460 | CMS4 |
| TCGA_TCGA-AA-3952 | CMS4 |
| TCGA_TCGA-AY-4070 | CMS4 |
| TCGA_TCGA-AZ-6600 | CMS4 |
| TCGA_TCGA-D5-6922 | CMS4 |
| TCGA_TCGA-AA-3489 | CMS4 |
| TCGA_TCGA-AZ-6603 | CMS4 |
| TCGA_TCGA-CK-6748 | CMS4 |
| TCGA_TCGA-A6-6142 | CMS4 |
| TCGA_TCGA-A6-A567 | CMS4 |
| TCGA_TCGA-A6-A565 | CMS4 |
| TCGA_TCGA-A6-2671 | CMS4 |
| TCGA_TCGA-CM-4747 | CMS4 |
| TCGA_TCGA-CM-6168 | CMS4 |
| TCGA_TCGA-A6-6651 | CMS4 |
| TCGA_TCGA-AA-3812 | CMS4 |
| TCGA_TCGA-A6-3810 | CMS4 |
| TCGA_TCGA-D5-6534 | CMS4 |
| TCGA_TCGA-G4-6303 | CMS4 |
| TCGA_TCGA-AA-3968 | CMS4 |
| TCGA_TCGA-F4-6805 | CMS4 |
| TCGA_TCGA-D5-6932 | CMS4 |
| TCGA_TCGA-DM-A28A | CMS4 |
| TCGA_TCGA-D5-6529 | CMS4 |
| TCGA_TCGA-AA-3496 | CMS4 |
| TCGA_TCGA-AD-6901 | CMS4 |
| TCGA_TCGA-F4-6855 | CMS4 |
| TCGA_TCGA-3L-AA1B | CMS4 |
| TCGA_TCGA-AA-A01C | CMS4 |
| TCGA_TCGA-AA-3973 | CMS4 |
| TCGA_TCGA-AA-3527 | CMS4 |
| TCGA_TCGA-EI-6884 | CMS4 |
| TCGA_TCGA-DC-6154 | CMS4 |
| TCGA_TCGA-AG-3609 | CMS4 |
| TCGA_TCGA-AG-4022 | CMS4 |
| TCGA_TCGA-AH-6549 | CMS4 |
| TCGA_TCGA-AG-3727 | CMS4 |
| TCGA_TCGA-F5-6863 | CMS4 |
| TCGA_TCGA-AF-3913 | CMS4 |
| TCGA_TCGA-G5-6572 | CMS4 |
| TCGA_TCGA-EI-6511 | CMS4 |
| TCGA_TCGA-AG-4008 | CMS4 |
| TCGA_TCGA-AG-3731 | CMS4 |
| TCGA_TCGA-AG-A026 | CMS4 |
| TCGA_TCGA-AG-3883 | CMS4 |
| TCGA_TCGA-EI-7004 | CMS4 |
| TCGA_TCGA-EI-6917 | CMS4 |
| TCGA_TCGA-F5-6465 | CMS4 |
| TCGA_TCGA-EI-6509 | CMS4 |
| TCGA_TCGA-AG-3893 | CMS4 |
| TCGA_TCGA-AG-3901 | CMS4 |
| TCGA_TCGA-AG-3726 | CMS4 |
| TCGA_TCGA-F5-6812 | CMS4 |
| TCGA_TCGA-AG-4001 | CMS4 |
| TCGA_TCGA-AF-2687 | CMS4 |
| TCGA_TCGA-AH-6644 | CMS4 |
| TCGA_TCGA-AG-3878 | CMS4 |
| TCGA_TCGA-DC-6158 | CMS4 |
| TCGA_TCGA-BM-6198 | CMS4 |
| TCGA_TCGA-AG-3881 | CMS4 |
| TCGA_TCGA-DY-A1DC | CMS4 |
| TCGA_TCGA-AG-3612 | CMS4 |
| TCGA_TCGA-AG-3584 | CMS4 |
| TCGA_TCGA-DC-6156 | CMS4 |
| TCGA_TCGA-DT-5265 | CMS4 |
| TCGA_TCGA-AG-A01Y | CMS4 |
| TCGA_TCGA-AF-6655 | CMS4 |
| TCGA_TCGA-AG-A023 | CMS4 |
| TCGA_TCGA-AG-3728 | CMS4 |
| TCGA_TCGA-AF-3400 | CMS4 |
| TCGA_TCGA-F5-6864 | CMS4 |
| TCGA_TCGA-AF-A56K | CMS4 |
| TCGA_TCGA-AG-4021 | CMS4 |
| TCGA_TCGA-DC-5869 | CMS4 |
| TCGA_TCGA-EF-5831 | CMS4 |
| TCGA_TCGA-EI-6514 | CMS4 |
| TCGA_TCGA-CI-6624 | CMS4 |
| TCGA_TCGA-CI-6620 | CMS4 |
| TCGA_TCGA-CI-6621 | CMS4 |
| TCGA_TCGA-AG-3999 | CMS4 |
| TCGA_TCGA-AG-A00H | CMS4 |
| TCGA_TCGA-AF-A56N | CMS4 |
| TCGA_TCGA-EI-6885 | CMS4 |
| TCGA_TCGA-AG-4007 | CMS4 |
| TCGA_TCGA-AG-3575 | CMS4 |
| TCGA_TCGA-AF-4110 | CMS4 |
| TCGA_TCGA-AG-3898 | CMS4 |
| TCGA_TCGA-F5-6813 | CMS4 |
| TCGA_TCGA-F5-6702 | CMS4 |
| TCGA_TCGA-CL-5917 | CMS4 |
| TCGA_TCGA-AF-2690 | CMS4 |
| TCGA_TCGA-AF-3911 | CMS4 |
| TCGA_TCGA-DC-6681 | CMS4 |
| TCGA_TCGA-AG-4005 | CMS4 |
| TCGA_TCGA-AH-6547 | CMS4 |
| TCGA_TCGA-EI-7002 | CMS4 |
| TCGA_TCGA-AH-6643 | CMS4 |
| TCGA_TCGA-F5-6571 | CMS4 |
| TCGA_TCGA-F5-6811 | CMS4 |
| TCGA_TCGA-F5-6810 | CMS4 |
| TCGA_TCGA-F5-6464 | CMS4 |
| TCGA_TCGA-CI-6619 | CMS4 |
| GSE39582_GSM971957 | CMS4 |
| GSE39582_GSM971958 | CMS4 |
| GSE39582_GSM971961 | CMS4 |
| GSE39582_GSM971962 | CMS4 |
| GSE39582_GSM971966 | CMS4 |
| GSE39582_GSM971968 | CMS4 |
| GSE39582_GSM971977 | CMS4 |
| GSE39582_GSM971986 | CMS4 |
| GSE39582_GSM971987 | CMS4 |
| GSE39582_GSM971991 | CMS4 |
| GSE39582_GSM971996 | CMS4 |
| GSE39582_GSM971999 | CMS4 |
| GSE39582_GSM972006 | CMS4 |
| GSE39582_GSM972007 | CMS4 |
| GSE39582_GSM972008 | CMS4 |
| GSE39582_GSM972009 | CMS4 |
| GSE39582_GSM972010 | CMS4 |
| GSE39582_GSM972011 | CMS4 |
| GSE39582_GSM972019 | CMS4 |
| GSE39582_GSM972026 | CMS4 |
| GSE39582_GSM972029 | CMS4 |
| GSE39582_GSM972030 | CMS4 |
| GSE39582_GSM972039 | CMS4 |
| GSE39582_GSM972046 | CMS4 |
| GSE39582_GSM972047 | CMS4 |
| GSE39582_GSM972051 | CMS4 |
| GSE39582_GSM972054 | CMS4 |
| GSE39582_GSM972055 | CMS4 |
| GSE39582_GSM972063 | CMS4 |
| GSE39582_GSM972064 | CMS4 |
| GSE39582_GSM972067 | CMS4 |
| GSE39582_GSM972068 | CMS4 |
| GSE39582_GSM972073 | CMS4 |
| GSE39582_GSM972077 | CMS4 |
| GSE39582_GSM972078 | CMS4 |
| GSE39582_GSM972079 | CMS4 |
| GSE39582_GSM972082 | CMS4 |
| GSE39582_GSM972084 | CMS4 |
| GSE39582_GSM972085 | CMS4 |
| GSE39582_GSM972088 | CMS4 |
| GSE39582_GSM972089 | CMS4 |
| GSE39582_GSM972092 | CMS4 |
| GSE39582_GSM972093 | CMS4 |
| GSE39582_GSM972094 | CMS4 |
| GSE39582_GSM972095 | CMS4 |
| GSE39582_GSM972097 | CMS4 |
| GSE39582_GSM972102 | CMS4 |
| GSE39582_GSM972104 | CMS4 |
| GSE39582_GSM972112 | CMS4 |
| GSE39582_GSM972113 | CMS4 |
| GSE39582_GSM972116 | CMS4 |
| GSE39582_GSM972120 | CMS4 |
| GSE39582_GSM972125 | CMS4 |
| GSE39582_GSM972127 | CMS4 |
| GSE39582_GSM972128 | CMS4 |
| GSE39582_GSM972131 | CMS4 |
| GSE39582_GSM972132 | CMS4 |
| GSE39582_GSM972133 | CMS4 |
| GSE39582_GSM972140 | CMS4 |
| GSE39582_GSM972141 | CMS4 |
| GSE39582_GSM972157 | CMS4 |
| GSE39582_GSM972158 | CMS4 |
| GSE39582_GSM972162 | CMS4 |
| GSE39582_GSM972164 | CMS4 |
| GSE39582_GSM972165 | CMS4 |
| GSE39582_GSM972170 | CMS4 |
| GSE39582_GSM972176 | CMS4 |
| GSE39582_GSM972179 | CMS4 |
| GSE39582_GSM972181 | CMS4 |
| GSE39582_GSM972184 | CMS4 |
| GSE39582_GSM972186 | CMS4 |
| GSE39582_GSM972187 | CMS4 |
| GSE39582_GSM972201 | CMS4 |
| GSE39582_GSM972204 | CMS4 |
| GSE39582_GSM972206 | CMS4 |
| GSE39582_GSM972207 | CMS4 |
| GSE39582_GSM972208 | CMS4 |
| GSE39582_GSM972210 | CMS4 |
| GSE39582_GSM972213 | CMS4 |
| GSE39582_GSM972231 | CMS4 |
| GSE39582_GSM972232 | CMS4 |
| GSE39582_GSM972234 | CMS4 |
| GSE39582_GSM972235 | CMS4 |
| GSE39582_GSM972238 | CMS4 |
| GSE39582_GSM972240 | CMS4 |
| GSE39582_GSM972242 | CMS4 |
| GSE39582_GSM972251 | CMS4 |
| GSE39582_GSM972252 | CMS4 |
| GSE39582_GSM972254 | CMS4 |
| GSE39582_GSM972255 | CMS4 |
| GSE39582_GSM972262 | CMS4 |
| GSE39582_GSM972275 | CMS4 |
| GSE39582_GSM972279 | CMS4 |
| GSE39582_GSM972290 | CMS4 |
| GSE39582_GSM972293 | CMS4 |
| GSE39582_GSM972297 | CMS4 |
| GSE39582_GSM972299 | CMS4 |
| GSE39582_GSM972301 | CMS4 |
| GSE39582_GSM972327 | CMS4 |
| GSE39582_GSM972328 | CMS4 |
| GSE39582_GSM972345 | CMS4 |
| GSE39582_GSM972349 | CMS4 |
| GSE39582_GSM972352 | CMS4 |
| GSE39582_GSM972355 | CMS4 |
| GSE39582_GSM972359 | CMS4 |
| GSE39582_GSM972361 | CMS4 |
| GSE39582_GSM972365 | CMS4 |
| GSE39582_GSM972366 | CMS4 |
| GSE39582_GSM972367 | CMS4 |
| GSE39582_GSM972369 | CMS4 |
| GSE39582_GSM972375 | CMS4 |
| GSE39582_GSM972376 | CMS4 |
| GSE39582_GSM972377 | CMS4 |
| GSE39582_GSM972378 | CMS4 |
| GSE39582_GSM972380 | CMS4 |
| GSE39582_GSM972384 | CMS4 |
| GSE39582_GSM972385 | CMS4 |
| GSE39582_GSM972393 | CMS4 |
| GSE39582_GSM972394 | CMS4 |
| GSE39582_GSM972401 | CMS4 |
| GSE39582_GSM972402 | CMS4 |
| GSE39582_GSM972407 | CMS4 |
| GSE39582_GSM972409 | CMS4 |
| GSE39582_GSM972410 | CMS4 |
| GSE39582_GSM972412 | CMS4 |
| GSE39582_GSM972413 | CMS4 |
| GSE39582_GSM972416 | CMS4 |
| GSE39582_GSM972417 | CMS4 |
| GSE39582_GSM972419 | CMS4 |
| GSE39582_GSM972421 | CMS4 |
| GSE39582_GSM972422 | CMS4 |
| GSE39582_GSM972425 | CMS4 |
| GSE39582_GSM972426 | CMS4 |
| GSE39582_GSM972427 | CMS4 |
| GSE39582_GSM972428 | CMS4 |
| GSE39582_GSM972431 | CMS4 |
| GSE39582_GSM972432 | CMS4 |
| GSE39582_GSM972433 | CMS4 |
| GSE39582_GSM972437 | CMS4 |
| GSE39582_GSM972438 | CMS4 |
| GSE39582_GSM972439 | CMS4 |
| GSE39582_GSM972440 | CMS4 |
| GSE39582_GSM972441 | CMS4 |
| GSE39582_GSM972444 | CMS4 |
| GSE39582_GSM972446 | CMS4 |
| GSE39582_GSM972449 | CMS4 |
| GSE39582_GSM972455 | CMS4 |
| GSE39582_GSM972464 | CMS4 |
| GSE39582_GSM972467 | CMS4 |
| GSE39582_GSM972470 | CMS4 |
| GSE39582_GSM972474 | CMS4 |
| GSE39582_GSM972476 | CMS4 |
| GSE39582_GSM972479 | CMS4 |
| GSE39582_GSM972481 | CMS4 |
| GSE39582_GSM972483 | CMS4 |
| GSE39582_GSM972484 | CMS4 |
| GSE39582_GSM972486 | CMS4 |
| GSE39582_GSM972493 | CMS4 |
| GSE39582_GSM972500 | CMS4 |
| GSE39582_GSM972501 | CMS4 |
| GSE39582_GSM972503 | CMS4 |
| GSE39582_GSM972504 | CMS4 |
| GSE39582_GSM972506 | CMS4 |
| GSE39582_GSM972517 | CMS4 |
| GSE39582_GSM972518 | CMS4 |
| GSE39582_GSM972519 | CMS4 |
| GSE39582_GSM972521 | CMS4 |

**Supplementary Table S7** | The molecular subtypes of GSE39582 samples.

| ID | type |
| --- | --- |
| GSE39582_GSM971971 | dMMR |
| GSE39582_GSM971977 | dMMR |
| GSE39582_GSM971978 | dMMR |
| GSE39582_GSM971979 | dMMR |
| GSE39582_GSM971980 | dMMR |
| GSE39582_GSM971981 | dMMR |
| GSE39582_GSM971982 | dMMR |
| GSE39582_GSM971983 | dMMR |
| GSE39582_GSM971984 | dMMR |
| GSE39582_GSM971988 | dMMR |
| GSE39582_GSM971997 | dMMR |
| GSE39582_GSM972015 | dMMR |
| GSE39582_GSM972016 | dMMR |
| GSE39582_GSM972021 | dMMR |
| GSE39582_GSM972030 | dMMR |
| GSE39582_GSM972055 | dMMR |
| GSE39582_GSM972056 | dMMR |
| GSE39582_GSM972058 | dMMR |
| GSE39582_GSM972059 | dMMR |
| GSE39582_GSM972075 | dMMR |
| GSE39582_GSM972076 | dMMR |
| GSE39582_GSM972079 | dMMR |
| GSE39582_GSM972080 | dMMR |
| GSE39582_GSM972082 | dMMR |
| GSE39582_GSM972103 | dMMR |
| GSE39582_GSM972109 | dMMR |
| GSE39582_GSM972117 | dMMR |
| GSE39582_GSM972129 | dMMR |
| GSE39582_GSM972146 | dMMR |
| GSE39582_GSM972168 | dMMR |
| GSE39582_GSM972171 | dMMR |
| GSE39582_GSM972172 | dMMR |
| GSE39582_GSM972180 | dMMR |
| GSE39582_GSM972187 | dMMR |
| GSE39582_GSM972189 | dMMR |
| GSE39582_GSM972192 | dMMR |
| GSE39582_GSM972196 | dMMR |
| GSE39582_GSM972214 | dMMR |
| GSE39582_GSM972220 | dMMR |
| GSE39582_GSM972221 | dMMR |
| GSE39582_GSM972222 | dMMR |
| GSE39582_GSM972223 | dMMR |
| GSE39582_GSM972224 | dMMR |
| GSE39582_GSM972225 | dMMR |
| GSE39582_GSM972229 | dMMR |
| GSE39582_GSM972241 | dMMR |
| GSE39582_GSM972261 | dMMR |
| GSE39582_GSM972263 | dMMR |
| GSE39582_GSM972264 | dMMR |
| GSE39582_GSM972265 | dMMR |
| GSE39582_GSM972266 | dMMR |
| GSE39582_GSM972270 | dMMR |
| GSE39582_GSM972274 | dMMR |
| GSE39582_GSM972276 | dMMR |
| GSE39582_GSM972277 | dMMR |
| GSE39582_GSM972278 | dMMR |
| GSE39582_GSM972280 | dMMR |
| GSE39582_GSM972282 | dMMR |
| GSE39582_GSM972283 | dMMR |
| GSE39582_GSM972285 | dMMR |
| GSE39582_GSM972286 | dMMR |
| GSE39582_GSM972287 | dMMR |
| GSE39582_GSM972292 | dMMR |
| GSE39582_GSM972293 | dMMR |
| GSE39582_GSM972294 | dMMR |
| GSE39582_GSM972297 | dMMR |
| GSE39582_GSM972298 | dMMR |
| GSE39582_GSM972305 | dMMR |
| GSE39582_GSM972308 | dMMR |
| GSE39582_GSM972314 | dMMR |
| GSE39582_GSM972316 | dMMR |
| GSE39582_GSM972317 | dMMR |
| GSE39582_GSM972327 | dMMR |
| GSE39582_GSM972330 | dMMR |
| GSE39582_GSM972332 | dMMR |
| GSE39582_GSM972334 | dMMR |
| GSE39582_GSM972337 | dMMR |
| GSE39582_GSM972346 | dMMR |
| GSE39582_GSM972353 | dMMR |
| GSE39582_GSM972354 | dMMR |
| GSE39582_GSM972391 | dMMR |
| GSE39582_GSM972403 | dMMR |
| GSE39582_GSM972406 | dMMR |
| GSE39582_GSM972420 | dMMR |
| GSE39582_GSM972425 | dMMR |
| GSE39582_GSM972430 | dMMR |
| GSE39582_GSM972445 | dMMR |
| GSE39582_GSM972447 | dMMR |
| GSE39582_GSM972448 | dMMR |
| GSE39582_GSM972452 | dMMR |
| GSE39582_GSM972455 | dMMR |
| GSE39582_GSM972458 | dMMR |
| GSE39582_GSM972465 | dMMR |
| GSE39582_GSM972470 | dMMR |
| GSE39582_GSM972471 | dMMR |
| GSE39582_GSM972482 | dMMR |
| GSE39582_GSM972494 | dMMR |
| GSE39582_GSM972499 | dMMR |
| GSE39582_GSM972500 | dMMR |
| GSE39582_GSM972507 | dMMR |
| GSE39582_GSM972520 | dMMR |
| GSE39582_GSM972522 | dMMR |
| GSE39582_GSM971959 | KRASm |
| GSE39582_GSM971967 | KRASm |
| GSE39582_GSM971970 | KRASm |
| GSE39582_GSM971974 | KRASm |
| GSE39582_GSM971985 | KRASm |
| GSE39582_GSM971989 | KRASm |
| GSE39582_GSM971990 | KRASm |
| GSE39582_GSM971993 | KRASm |
| GSE39582_GSM972002 | KRASm |
| GSE39582_GSM972005 | KRASm |
| GSE39582_GSM972012 | KRASm |
| GSE39582_GSM972023 | KRASm |
| GSE39582_GSM972036 | KRASm |
| GSE39582_GSM972037 | KRASm |
| GSE39582_GSM972042 | KRASm |
| GSE39582_GSM972044 | KRASm |
| GSE39582_GSM972045 | KRASm |
| GSE39582_GSM972049 | KRASm |
| GSE39582_GSM972050 | KRASm |
| GSE39582_GSM972091 | KRASm |
| GSE39582_GSM972098 | KRASm |
| GSE39582_GSM972100 | KRASm |
| GSE39582_GSM972110 | KRASm |
| GSE39582_GSM972111 | KRASm |
| GSE39582_GSM972114 | KRASm |
| GSE39582_GSM972121 | KRASm |
| GSE39582_GSM972135 | KRASm |
| GSE39582_GSM972151 | KRASm |
| GSE39582_GSM972159 | KRASm |
| GSE39582_GSM972178 | KRASm |
| GSE39582_GSM972183 | KRASm |
| GSE39582_GSM972191 | KRASm |
| GSE39582_GSM972195 | KRASm |
| GSE39582_GSM972197 | KRASm |
| GSE39582_GSM972212 | KRASm |
| GSE39582_GSM972218 | KRASm |
| GSE39582_GSM972226 | KRASm |
| GSE39582_GSM972227 | KRASm |
| GSE39582_GSM972228 | KRASm |
| GSE39582_GSM972259 | KRASm |
| GSE39582_GSM972268 | KRASm |
| GSE39582_GSM972269 | KRASm |
| GSE39582_GSM972273 | KRASm |
| GSE39582_GSM972295 | KRASm |
| GSE39582_GSM972296 | KRASm |
| GSE39582_GSM972300 | KRASm |
| GSE39582_GSM972304 | KRASm |
| GSE39582_GSM972315 | KRASm |
| GSE39582_GSM972325 | KRASm |
| GSE39582_GSM972326 | KRASm |
| GSE39582_GSM972350 | KRASm |
| GSE39582_GSM972364 | KRASm |
| GSE39582_GSM972379 | KRASm |
| GSE39582_GSM972389 | KRASm |
| GSE39582_GSM972390 | KRASm |
| GSE39582_GSM972396 | KRASm |
| GSE39582_GSM972429 | KRASm |
| GSE39582_GSM972443 | KRASm |
| GSE39582_GSM972446 | KRASm |
| GSE39582_GSM972453 | KRASm |
| GSE39582_GSM972457 | KRASm |
| GSE39582_GSM972459 | KRASm |
| GSE39582_GSM972463 | KRASm |
| GSE39582_GSM972472 | KRASm |
| GSE39582_GSM972473 | KRASm |
| GSE39582_GSM972475 | KRASm |
| GSE39582_GSM972477 | KRASm |
| GSE39582_GSM972480 | KRASm |
| GSE39582_GSM972487 | KRASm |
| GSE39582_GSM972488 | KRASm |
| GSE39582_GSM972489 | KRASm |
| GSE39582_GSM972496 | KRASm |
| GSE39582_GSM972502 | KRASm |
| GSE39582_GSM972510 | KRASm |
| GSE39582_GSM972512 | KRASm |
| GSE39582_GSM971958 | CSC |
| GSE39582_GSM971961 | CSC |
| GSE39582_GSM971962 | CSC |
| GSE39582_GSM971968 | CSC |
| GSE39582_GSM971996 | CSC |
| GSE39582_GSM972007 | CSC |
| GSE39582_GSM972008 | CSC |
| GSE39582_GSM972011 | CSC |
| GSE39582_GSM972019 | CSC |
| GSE39582_GSM972026 | CSC |
| GSE39582_GSM972039 | CSC |
| GSE39582_GSM972051 | CSC |
| GSE39582_GSM972054 | CSC |
| GSE39582_GSM972064 | CSC |
| GSE39582_GSM972068 | CSC |
| GSE39582_GSM972072 | CSC |
| GSE39582_GSM972073 | CSC |
| GSE39582_GSM972089 | CSC |
| GSE39582_GSM972097 | CSC |
| GSE39582_GSM972104 | CSC |
| GSE39582_GSM972112 | CSC |
| GSE39582_GSM972113 | CSC |
| GSE39582_GSM972131 | CSC |
| GSE39582_GSM972140 | CSC |
| GSE39582_GSM972158 | CSC |
| GSE39582_GSM972181 | CSC |
| GSE39582_GSM972184 | CSC |
| GSE39582_GSM972186 | CSC |
| GSE39582_GSM972210 | CSC |
| GSE39582_GSM972231 | CSC |
| GSE39582_GSM972232 | CSC |
| GSE39582_GSM972234 | CSC |
| GSE39582_GSM972239 | CSC |
| GSE39582_GSM972240 | CSC |
| GSE39582_GSM972262 | CSC |
| GSE39582_GSM972275 | CSC |
| GSE39582_GSM972279 | CSC |
| GSE39582_GSM972291 | CSC |
| GSE39582_GSM972345 | CSC |
| GSE39582_GSM972349 | CSC |
| GSE39582_GSM972361 | CSC |
| GSE39582_GSM972365 | CSC |
| GSE39582_GSM972367 | CSC |
| GSE39582_GSM972375 | CSC |
| GSE39582_GSM972380 | CSC |
| GSE39582_GSM972385 | CSC |
| GSE39582_GSM972401 | CSC |
| GSE39582_GSM972409 | CSC |
| GSE39582_GSM972412 | CSC |
| GSE39582_GSM972413 | CSC |
| GSE39582_GSM972422 | CSC |
| GSE39582_GSM972433 | CSC |
| GSE39582_GSM972438 | CSC |
| GSE39582_GSM972440 | CSC |
| GSE39582_GSM972444 | CSC |
| GSE39582_GSM972467 | CSC |
| GSE39582_GSM972476 | CSC |
| GSE39582_GSM972479 | CSC |
| GSE39582_GSM972521 | CSC |
| GSE39582_GSM971987 | CINnormal |
| GSE39582_GSM971995 | CINnormal |
| GSE39582_GSM971998 | CINnormal |
| GSE39582_GSM971999 | CINnormal |
| GSE39582_GSM972009 | CINnormal |
| GSE39582_GSM972020 | CINnormal |
| GSE39582_GSM972028 | CINnormal |
| GSE39582_GSM972029 | CINnormal |
| GSE39582_GSM972062 | CINnormal |
| GSE39582_GSM972070 | CINnormal |
| GSE39582_GSM972077 | CINnormal |
| GSE39582_GSM972084 | CINnormal |
| GSE39582_GSM972088 | CINnormal |
| GSE39582_GSM972101 | CINnormal |
| GSE39582_GSM972102 | CINnormal |
| GSE39582_GSM972107 | CINnormal |
| GSE39582_GSM972115 | CINnormal |
| GSE39582_GSM972116 | CINnormal |
| GSE39582_GSM972124 | CINnormal |
| GSE39582_GSM972125 | CINnormal |
| GSE39582_GSM972127 | CINnormal |
| GSE39582_GSM972133 | CINnormal |
| GSE39582_GSM972145 | CINnormal |
| GSE39582_GSM972150 | CINnormal |
| GSE39582_GSM972156 | CINnormal |
| GSE39582_GSM972163 | CINnormal |
| GSE39582_GSM972165 | CINnormal |
| GSE39582_GSM972174 | CINnormal |
| GSE39582_GSM972177 | CINnormal |
| GSE39582_GSM972200 | CINnormal |
| GSE39582_GSM972206 | CINnormal |
| GSE39582_GSM972244 | CINnormal |
| GSE39582_GSM972301 | CINnormal |
| GSE39582_GSM972320 | CINnormal |
| GSE39582_GSM972321 | CINnormal |
| GSE39582_GSM972329 | CINnormal |
| GSE39582_GSM972336 | CINnormal |
| GSE39582_GSM972338 | CINnormal |
| GSE39582_GSM972351 | CINnormal |
| GSE39582_GSM972357 | CINnormal |
| GSE39582_GSM972359 | CINnormal |
| GSE39582_GSM972366 | CINnormal |
| GSE39582_GSM972374 | CINnormal |
| GSE39582_GSM972383 | CINnormal |
| GSE39582_GSM972394 | CINnormal |
| GSE39582_GSM972400 | CINnormal |
| GSE39582_GSM972423 | CINnormal |
| GSE39582_GSM972424 | CINnormal |
| GSE39582_GSM972426 | CINnormal |
| GSE39582_GSM972432 | CINnormal |
| GSE39582_GSM972439 | CINnormal |
| GSE39582_GSM972441 | CINnormal |
| GSE39582_GSM972449 | CINnormal |
| GSE39582_GSM972454 | CINnormal |
| GSE39582_GSM972474 | CINnormal |
| GSE39582_GSM972478 | CINnormal |
| GSE39582_GSM972503 | CINnormal |
| GSE39582_GSM972506 | CINnormal |
| GSE39582_GSM972518 | CINnormal |
| GSE39582_GSM972519 | CINnormal |

**Supplementary Table S8** | The univariate COX regression results of 260 genes with significant prognostic value in TCGA and GEO data.

| id | HR | HR.95L | HR.95H | pvalue |
| --- | --- | --- | --- | --- |
| CDKN2A | 1.19452 | 1.052865 | 1.355232 | 0.005783 |
| EVA1B | 1.499368 | 1.1245 | 1.999204 | 0.005792 |
| DLD | 0.71647 | 0.52347 | 0.980629 | 0.037332 |
| LYL1 | 1.340795 | 1.072454 | 1.676279 | 0.010056 |
| GYPC | 1.254606 | 1.048393 | 1.501382 | 0.013294 |
| DLAT | 0.741644 | 0.551147 | 0.997983 | 0.048462 |
| MFGE8 | 1.257766 | 1.022159 | 1.54768 | 0.03023 |
| IGFBP6 | 1.265546 | 1.081974 | 1.480264 | 0.003226 |
| SERBP1 | 0.625832 | 0.405116 | 0.966797 | 0.034675 |
| SMARCD3 | 1.579071 | 1.221036 | 2.04209 | 0.000497 |
| EGFL7 | 1.404964 | 1.095981 | 1.801056 | 0.007292 |
| MEIS3 | 1.324548 | 1.08742 | 1.613385 | 0.005228 |
| EFEMP2 | 1.242046 | 1.052648 | 1.465521 | 0.010235 |
| MCCC2 | 0.50154 | 0.344628 | 0.729896 | 0.000313 |
| STAB1 | 1.36746 | 1.067274 | 1.752077 | 0.01333 |
| LTBP3 | 1.590706 | 1.24616 | 2.030514 | 0.000194 |
| CLDN5 | 1.179514 | 1.010251 | 1.377135 | 0.036706 |
| CERCAM | 1.534534 | 1.237041 | 1.90357 | 9.83E-05 |
| TFAM | 0.712598 | 0.532997 | 0.952718 | 0.022204 |
| TRUB1 | 0.68808 | 0.478176 | 0.990125 | 0.044072 |
| NARS2 | 0.634792 | 0.47247 | 0.85288 | 0.00256 |
| GGT5 | 1.252213 | 1.044521 | 1.501203 | 0.015069 |
| LRPPRC | 0.665373 | 0.489071 | 0.905228 | 0.009489 |
| PLEKHO2 | 1.31932 | 1.020741 | 1.705236 | 0.034279 |
| CCDC47 | 0.675692 | 0.477344 | 0.956458 | 0.027032 |
| CLEC11A | 1.364954 | 1.099616 | 1.694319 | 0.004787 |
| SRSF1 | 0.618901 | 0.399751 | 0.958192 | 0.03144 |
| PRRX2 | 1.301267 | 1.132498 | 1.495187 | 0.000203 |
| PDLIM4 | 1.205976 | 1.008205 | 1.442541 | 0.040424 |
| NFATC1 | 1.228803 | 1.045834 | 1.443783 | 0.012254 |
| CRIP2 | 1.313607 | 1.101976 | 1.565881 | 0.00234 |
| COL6A2 | 1.211366 | 1.019241 | 1.439705 | 0.029535 |
| PTGIR | 1.324037 | 1.093241 | 1.603557 | 0.004077 |
| CHST12 | 1.32073 | 1.00224 | 1.74043 | 0.048171 |
| COX7A1 | 1.263655 | 1.074665 | 1.485879 | 0.004638 |
| EBF4 | 1.278823 | 1.054237 | 1.551253 | 0.01256 |
| PPM1M | 1.46142 | 1.111236 | 1.921957 | 0.006635 |
| RCN3 | 1.418864 | 1.142577 | 1.761958 | 0.001544 |
| TMEM204 | 1.4346 | 1.069027 | 1.925187 | 0.016184 |
| COPZ2 | 1.394078 | 1.134942 | 1.71238 | 0.001544 |
| FOLR2 | 1.185601 | 1.014496 | 1.385565 | 0.032278 |
| BRIP1 | 0.766443 | 0.646979 | 0.907966 | 0.002093 |
| SIPA1 | 1.594223 | 1.149043 | 2.211882 | 0.005246 |
| DIAPH3 | 0.725477 | 0.566251 | 0.929476 | 0.011135 |
| PLA2G12A | 0.712805 | 0.532626 | 0.953937 | 0.022777 |
| PDE12 | 0.442638 | 0.265392 | 0.73826 | 0.001792 |
| CLCN3 | 0.706856 | 0.5463 | 0.9146 | 0.008315 |
| ERI2 | 0.672867 | 0.486831 | 0.929993 | 0.016417 |
| MRS2 | 0.56153 | 0.387995 | 0.812682 | 0.002216 |
| PLD3 | 1.359917 | 1.027492 | 1.799891 | 0.031587 |
| RIOK2 | 0.708701 | 0.541185 | 0.928069 | 0.012332 |
| RPP14 | 0.495613 | 0.343995 | 0.714059 | 0.000165 |
| SRSF3 | 0.506093 | 0.309615 | 0.827253 | 0.0066 |
| AMD1 | 0.695733 | 0.508753 | 0.951431 | 0.023103 |
| DHX15 | 0.444495 | 0.295221 | 0.669248 | 0.000103 |
| LOXL1 | 1.146792 | 1.005731 | 1.307638 | 0.040825 |
| RBM47 | 0.710759 | 0.510604 | 0.989375 | 0.043046 |
| FAM110D | 1.239371 | 1.017451 | 1.509695 | 0.033022 |
| PLXND1 | 1.533377 | 1.157722 | 2.030924 | 0.002869 |
| VASN | 1.299356 | 1.033815 | 1.633103 | 0.024763 |
| RAD18 | 0.593658 | 0.437996 | 0.804642 | 0.000777 |
| TBXA2R | 1.37507 | 1.095065 | 1.726673 | 0.006112 |
| TCAIM | 0.705029 | 0.520148 | 0.955624 | 0.024292 |
| PARPBP | 0.654131 | 0.532531 | 0.803499 | 5.23E-05 |
| CPSF6 | 0.64961 | 0.446509 | 0.945094 | 0.024122 |
| FAM167B | 1.447249 | 1.111503 | 1.884411 | 0.006052 |
| TUBB6 | 1.238967 | 1.067826 | 1.437537 | 0.004725 |
| ZW10 | 0.643897 | 0.442376 | 0.937219 | 0.021533 |
| CCDC43 | 0.670856 | 0.493326 | 0.912272 | 0.010915 |
| NUMBL | 1.983069 | 1.487085 | 2.644478 | 3.13E-06 |
| NOL11 | 0.610362 | 0.450835 | 0.826338 | 0.001403 |
| AGGF1 | 0.5743 | 0.394527 | 0.83599 | 0.003791 |
| SLC39A13 | 2.292797 | 1.568416 | 3.351738 | 1.84E-05 |
| KLF2 | 1.58936 | 1.248493 | 2.023292 | 0.000169 |
| DDX52 | 0.586409 | 0.431879 | 0.796233 | 0.000626 |
| SYNGR1 | 1.17688 | 1.021837 | 1.355447 | 0.023841 |
| CENPK | 0.841746 | 0.732412 | 0.967401 | 0.015231 |
| KIF11 | 0.819709 | 0.700253 | 0.959544 | 0.013367 |
| SSX2IP | 0.629102 | 0.507144 | 0.780388 | 2.50E-05 |
| NXN | 1.214666 | 1.072295 | 1.37594 | 0.002233 |
| BGN | 1.197062 | 1.023461 | 1.400109 | 0.024445 |
| RAB3IP | 0.708559 | 0.511682 | 0.981186 | 0.03805 |
| HMGCR | 0.682589 | 0.537185 | 0.86735 | 0.001782 |
| DDAH1 | 0.626618 | 0.401485 | 0.977996 | 0.039597 |
| MAPKAPK5 | 0.542481 | 0.388248 | 0.757984 | 0.000339 |
| ABCD3 | 0.749359 | 0.593074 | 0.946828 | 0.015615 |
| LOXL3 | 1.385182 | 1.06128 | 1.807938 | 0.016502 |
| ARSI | 1.190824 | 1.045469 | 1.356389 | 0.008553 |
| MRAS | 1.284072 | 1.079902 | 1.526843 | 0.004655 |
| DLEU1 | 0.816267 | 0.675665 | 0.986126 | 0.035307 |
| WDR3 | 0.578101 | 0.392069 | 0.852404 | 0.005675 |
| NAGK | 1.429909 | 1.034477 | 1.976498 | 0.030373 |
| MPP7 | 0.785501 | 0.642288 | 0.960648 | 0.018728 |
| FIGNL1 | 0.765902 | 0.633988 | 0.925263 | 0.005685 |
| PRPF38A | 0.697282 | 0.488872 | 0.994538 | 0.04657 |
| HOMER3 | 1.453325 | 1.1797 | 1.790415 | 0.000443 |
| RTN4IP1 | 0.691518 | 0.535982 | 0.892188 | 0.004547 |
| GRPEL1 | 0.631172 | 0.450839 | 0.883636 | 0.007349 |
| ABHD8 | 1.375859 | 1.034724 | 1.829461 | 0.028181 |
| SDAD1 | 0.716817 | 0.515592 | 0.996577 | 0.047663 |
| GJA4 | 1.294679 | 1.017482 | 1.647394 | 0.035645 |
| COL6A1 | 1.394556 | 1.038573 | 1.872557 | 0.026991 |
| OCLN | 0.690382 | 0.546443 | 0.872238 | 0.001898 |
| RAB3IL1 | 1.320974 | 1.07215 | 1.627544 | 0.008943 |
| GPSM1 | 1.35542 | 1.140168 | 1.611309 | 0.000568 |
| GPC1 | 1.385902 | 1.095917 | 1.75262 | 0.006437 |
| MMAA | 0.70148 | 0.5253 | 0.936748 | 0.016272 |
| CAMKMT | 0.659186 | 0.468543 | 0.927399 | 0.016725 |
| OXNAD1 | 0.591822 | 0.432622 | 0.809606 | 0.001034 |
| FHL3 | 1.583338 | 1.204395 | 2.08151 | 0.000993 |
| CLCF1 | 1.533845 | 1.161767 | 2.02509 | 0.002547 |
| NSA2 | 0.739286 | 0.555223 | 0.984368 | 0.038657 |
| ATAD5 | 0.75568 | 0.593606 | 0.962007 | 0.022939 |
| TOP2A | 0.756702 | 0.607227 | 0.942972 | 0.013031 |
| HTRA1 | 1.235523 | 1.054641 | 1.447428 | 0.008827 |
| CPOX | 0.711213 | 0.510149 | 0.991521 | 0.044411 |
| GSN | 1.594116 | 1.060178 | 2.396961 | 0.025041 |
| RMI1 | 0.730769 | 0.585749 | 0.911693 | 0.005451 |
| BORA | 0.726774 | 0.585734 | 0.901776 | 0.003741 |
| TPD52 | 0.795532 | 0.642845 | 0.984483 | 0.035396 |
| PKP2 | 0.760981 | 0.608325 | 0.951945 | 0.0168 |
| PDIK1L | 0.769002 | 0.627596 | 0.942269 | 0.011292 |
| PRAM1 | 1.196497 | 1.022345 | 1.400315 | 0.025396 |
| KIF2A | 0.659098 | 0.512665 | 0.847356 | 0.001146 |
| TRNP1 | 1.144667 | 1.003266 | 1.305997 | 0.044598 |
| ITGA5 | 1.291619 | 1.111181 | 1.501358 | 0.000859 |
| SUSD2 | 1.186914 | 1.012918 | 1.390797 | 0.034119 |
| PPP1R18 | 1.334248 | 1.08557 | 1.639892 | 0.006141 |
| TMCC2 | 1.303339 | 1.058056 | 1.605485 | 0.012758 |
| COX11 | 0.516911 | 0.369332 | 0.723459 | 0.000119 |
| SATB2 | 0.904381 | 0.820287 | 0.997097 | 0.043555 |
| CHST11 | 1.202873 | 1.016045 | 1.424055 | 0.03197 |
| TOM1L1 | 0.722358 | 0.554875 | 0.940394 | 0.015666 |
| F2RL1 | 0.778438 | 0.632306 | 0.958343 | 0.018223 |
| PDZD8 | 0.806153 | 0.660443 | 0.98401 | 0.034138 |
| HNF4G | 0.816424 | 0.676194 | 0.985735 | 0.034911 |
| NUP107 | 0.744972 | 0.55646 | 0.997346 | 0.047949 |
| HOXB2 | 1.287734 | 1.117129 | 1.484395 | 0.000488 |
| TPRKB | 0.735388 | 0.555563 | 0.973418 | 0.031692 |
| GFPT2 | 1.115851 | 1.003688 | 1.240549 | 0.042553 |
| USP1 | 0.766538 | 0.6038 | 0.973137 | 0.028991 |
| CEBPG | 0.649547 | 0.447874 | 0.94203 | 0.022918 |
| CDC42SE2 | 0.759881 | 0.587718 | 0.982477 | 0.036186 |
| ADAP2 | 1.314389 | 1.05153 | 1.642959 | 0.016336 |
| FASTKD3 | 0.737748 | 0.572189 | 0.951212 | 0.01899 |
| COMP | 1.118917 | 1.038945 | 1.205044 | 0.00298 |
| PUS7 | 0.760211 | 0.611119 | 0.945676 | 0.013838 |
| DSN1 | 0.650918 | 0.511157 | 0.828894 | 0.000498 |
| TPMT | 0.574192 | 0.381208 | 0.864873 | 0.00794 |
| STARD4 | 0.836503 | 0.713633 | 0.980527 | 0.027624 |
| ACSL6 | 0.893569 | 0.824165 | 0.968818 | 0.006374 |
| LINGO3 | 1.172947 | 1.026833 | 1.339852 | 0.018771 |
| SHQ1 | 0.35951 | 0.235082 | 0.549796 | 2.36E-06 |
| SRPX | 1.171736 | 1.054968 | 1.301428 | 0.003086 |
| LAMP5 | 1.217106 | 1.092585 | 1.35582 | 0.00036 |
| EIF2S2 | 0.676207 | 0.494822 | 0.924084 | 0.01407 |
| CORO6 | 1.203966 | 1.037789 | 1.396753 | 0.014308 |
| TTC19 | 0.733141 | 0.548498 | 0.979939 | 0.036007 |
| MRPS25 | 0.580345 | 0.401832 | 0.838163 | 0.003716 |
| SOX18 | 1.277837 | 1.027826 | 1.588662 | 0.027312 |
| KCNJ5 | 1.189142 | 1.043469 | 1.355151 | 0.009373 |
| TRABD2B | 1.178877 | 1.009806 | 1.376256 | 0.037205 |
| HAS1 | 1.191309 | 1.082108 | 1.311531 | 0.000359 |
| SLC35B3 | 0.746138 | 0.559009 | 0.995908 | 0.046834 |
| MYRIP | 0.86412 | 0.769947 | 0.96981 | 0.013114 |
| LRRC2 | 0.893544 | 0.798915 | 0.999381 | 0.048746 |
| ZBTB4 | 1.599947 | 1.149064 | 2.227754 | 0.005392 |
| KIDINS220 | 1.873454 | 1.214988 | 2.888775 | 0.004493 |
| LRRC31 | 0.890939 | 0.80966 | 0.980378 | 0.017982 |
| KIAA2026 | 1.493601 | 1.059517 | 2.105528 | 0.022024 |
| MLLT3 | 0.826323 | 0.682915 | 0.999847 | 0.049817 |
| MAB21L3 | 0.863571 | 0.75752 | 0.984469 | 0.028227 |
| GOLIM4 | 0.732557 | 0.562877 | 0.953386 | 0.02061 |
| FHDC1 | 0.682986 | 0.564382 | 0.826515 | 8.94E-05 |
| VAV3 | 0.894517 | 0.82503 | 0.969857 | 0.006897 |
| CISD1 | 0.67968 | 0.481099 | 0.96023 | 0.028514 |
| SWI5 | 1.498525 | 1.070327 | 2.098029 | 0.018483 |
| SMARCA2 | 1.529868 | 1.008492 | 2.32079 | 0.045529 |
| RALGAPA2 | 0.758708 | 0.59331 | 0.970213 | 0.027737 |
| NOP16 | 0.581443 | 0.374375 | 0.903042 | 0.015779 |
| MCM4 | 0.754143 | 0.579964 | 0.980633 | 0.035211 |
| AUNIP | 0.783419 | 0.639306 | 0.960019 | 0.018605 |
| PLCB4 | 0.895417 | 0.831827 | 0.963869 | 0.003292 |
| RAN | 0.582282 | 0.394782 | 0.858835 | 0.006382 |
| RNF43 | 0.831129 | 0.73622 | 0.938272 | 0.002791 |
| AKNA | 0.643157 | 0.442318 | 0.935189 | 0.020845 |
| FMNL2 | 0.693771 | 0.495437 | 0.971501 | 0.033315 |
| TMX2 | 0.55571 | 0.36774 | 0.83976 | 0.005287 |
| GYG2 | 0.811675 | 0.702548 | 0.937752 | 0.00462 |
| TRIP13 | 0.791021 | 0.646538 | 0.967793 | 0.022721 |
| ACE2 | 0.922344 | 0.860559 | 0.988566 | 0.02231 |
| TNNT1 | 1.095096 | 1.017937 | 1.178102 | 0.014815 |
| PPP1R8 | 0.527346 | 0.329732 | 0.843395 | 0.007566 |
| PPIH | 0.668728 | 0.482258 | 0.927299 | 0.015843 |
| PLAGL2 | 0.721832 | 0.60607 | 0.859707 | 0.000257 |
| FNBP1 | 1.336353 | 1.051734 | 1.697995 | 0.017657 |
| ZNF234 | 0.741847 | 0.560745 | 0.981439 | 0.036513 |
| MAP2K1 | 1.481117 | 1.035976 | 2.117528 | 0.031259 |
| CDKN3 | 0.829493 | 0.690305 | 0.996746 | 0.046071 |
| SCUBE2 | 1.146769 | 1.010106 | 1.301921 | 0.034406 |
| CKS2 | 0.738196 | 0.582152 | 0.936068 | 0.012237 |
| PROX1 | 0.865918 | 0.757168 | 0.990287 | 0.035509 |
| CENPN | 0.70497 | 0.528355 | 0.940623 | 0.017502 |
| KIF2C | 0.729846 | 0.586596 | 0.908077 | 0.004729 |
| CDX2 | 0.836059 | 0.729153 | 0.958638 | 0.010315 |
| PKM | 1.376392 | 1.057866 | 1.790827 | 0.017367 |
| BIRC5 | 0.666229 | 0.53756 | 0.825695 | 0.000208 |
| OIP5 | 0.824988 | 0.699 | 0.973684 | 0.022884 |
| CACNA1D | 0.795266 | 0.673848 | 0.938562 | 0.006726 |
| ACOX2 | 0.818875 | 0.724322 | 0.925772 | 0.001413 |
| UPP1 | 1.280595 | 1.041265 | 1.574933 | 0.019127 |
| KCTD1 | 1.319958 | 1.110241 | 1.56929 | 0.001663 |
| CEACAM5 | 0.766894 | 0.603728 | 0.974158 | 0.02967 |
| BOLA3 | 0.607457 | 0.444125 | 0.830857 | 0.001811 |
| ORC1 | 0.630918 | 0.517538 | 0.769137 | 5.19E-06 |
| RBMS3 | 1.233897 | 1.04944 | 1.450777 | 0.010957 |
| CALCOCO1 | 1.521858 | 1.088324 | 2.12809 | 0.014099 |
| ARHGAP44 | 0.831958 | 0.711456 | 0.972871 | 0.021196 |
| NEGR1 | 1.183488 | 1.034025 | 1.354554 | 0.014457 |
| FAM83A | 1.119246 | 1.005255 | 1.246164 | 0.039821 |
| PTPRD | 0.891423 | 0.81237 | 0.97817 | 0.015274 |
| LGR5 | 0.874344 | 0.805682 | 0.948858 | 0.001291 |
| PFKP | 1.452771 | 1.022324 | 2.064457 | 0.037241 |
| STX18 | 0.522983 | 0.342653 | 0.798218 | 0.002659 |
| SLC26A2 | 0.897956 | 0.822051 | 0.980871 | 0.016913 |
| MRPL22 | 0.586541 | 0.420661 | 0.817831 | 0.001657 |
| QDPR | 0.615666 | 0.39383 | 0.962458 | 0.033352 |
| SNRPF | 0.661271 | 0.484612 | 0.90233 | 0.009106 |
| AIFM3 | 0.873159 | 0.773152 | 0.986101 | 0.028854 |
| POLG | 1.737167 | 1.072855 | 2.812821 | 0.024706 |
| DIP2C | 1.303372 | 1.080973 | 1.571527 | 0.005509 |
| NDUFA12 | 0.556586 | 0.346284 | 0.894608 | 0.015524 |
| FEN1 | 0.756428 | 0.590867 | 0.968379 | 0.026766 |
| SEMA5A | 0.860556 | 0.753244 | 0.983156 | 0.027109 |
| CHAF1B | 0.639103 | 0.476409 | 0.857359 | 0.00282 |
| RPA3 | 0.701278 | 0.520894 | 0.944128 | 0.01934 |
| ZC3H12D | 0.850887 | 0.726518 | 0.996546 | 0.04519 |
| NME1 | 0.660576 | 0.510983 | 0.853963 | 0.001551 |
| MRPL37 | 0.573306 | 0.409681 | 0.802284 | 0.001175 |
| SLC2A1 | 1.269607 | 1.060963 | 1.519282 | 0.00916 |
| DACH1 | 0.897714 | 0.822335 | 0.980004 | 0.015893 |
| CCNB2 | 0.593455 | 0.389257 | 0.90477 | 0.015306 |
| PTDSS1 | 0.686123 | 0.504869 | 0.932449 | 0.016092 |
| PPIL1 | 0.64796 | 0.480799 | 0.87324 | 0.004368 |
| NAPRT | 1.281981 | 1.014286 | 1.620329 | 0.037649 |
| FOXD1 | 1.130376 | 1.060361 | 1.205014 | 0.000172 |
| FMO5 | 0.842129 | 0.714292 | 0.992846 | 0.040812 |
| FBXW9 | 0.701573 | 0.511469 | 0.962336 | 0.027945 |
| ALYREF | 0.777588 | 0.634657 | 0.952709 | 0.015205 |
| CDCA5 | 0.786511 | 0.631495 | 0.979581 | 0.03202 |
| GNPDA1 | 0.704918 | 0.505619 | 0.982773 | 0.039164 |
| SHROOM4 | 0.832905 | 0.694411 | 0.99902 | 0.048777 |
| SUMO3 | 1.588316 | 1.029951 | 2.449386 | 0.036305 |
| SNRNP40 | 0.74427 | 0.584897 | 0.947068 | 0.016292 |
| KDM1A | 0.589331 | 0.419325 | 0.828263 | 0.002326 |
| RANBP1 | 0.460872 | 0.286521 | 0.741319 | 0.001402 |
| GPRASP1 | 1.269885 | 1.11988 | 1.439983 | 0.000195 |
| CDCA3 | 0.750452 | 0.604783 | 0.931208 | 0.009127 |
| MIR31HG | 1.213127 | 1.129802 | 1.302598 | 1.03E-07 |
| RPL35A | 1.962258 | 1.015553 | 3.791488 | 0.044868 |

**Supplementary Table S9** | The geneCluster group of TCGA and GEO samples.

| ID | geneCluster |
| --- | --- |
| TCGA_TCGA-DM-A288 | A |
| TCGA_TCGA-QL-A97D | B |
| TCGA_TCGA-CM-6164 | B |
| TCGA_TCGA-G4-6299 | A |
| TCGA_TCGA-F4-6463 | C |
| TCGA_TCGA-AZ-4615 | A |
| TCGA_TCGA-AA-3549 | B |
| TCGA_TCGA-AY-4071 | C |
| TCGA_TCGA-CM-4752 | B |
| TCGA_TCGA-DM-A1D9 | B |
| TCGA_TCGA-AA-3688 | B |
| TCGA_TCGA-AA-3854 | A |
| TCGA_TCGA-A6-3809 | A |
| TCGA_TCGA-CM-6165 | B |
| TCGA_TCGA-CM-4751 | C |
| TCGA_TCGA-A6-5659 | B |
| TCGA_TCGA-AA-3494 | B |
| TCGA_TCGA-CM-4750 | B |
| TCGA_TCGA-AZ-4682 | B |
| TCGA_TCGA-G4-6625 | C |
| TCGA_TCGA-DM-A0XF | B |
| TCGA_TCGA-AA-3529 | B |
| TCGA_TCGA-AA-3949 | A |
| TCGA_TCGA-AA-3848 | C |
| TCGA_TCGA-CA-6715 | B |
| TCGA_TCGA-AA-3818 | B |
| TCGA_TCGA-AA-3710 | A |
| TCGA_TCGA-AA-3950 | A |
| TCGA_TCGA-AA-A00N | C |
| TCGA_TCGA-A6-6138 | B |
| TCGA_TCGA-DM-A28E | B |
| TCGA_TCGA-D5-6538 | B |
| TCGA_TCGA-AA-A00K | B |
| TCGA_TCGA-AA-3715 | C |
| TCGA_TCGA-AA-3506 | C |
| TCGA_TCGA-AU-3779 | B |
| TCGA_TCGA-CK-5916 | A |
| TCGA_TCGA-QG-A5Z1 | B |
| TCGA_TCGA-AD-6890 | B |
| TCGA_TCGA-A6-2685 | C |
| TCGA_TCGA-AA-A00W | B |
| TCGA_TCGA-CK-5914 | B |
| TCGA_TCGA-A6-2686 | A |
| TCGA_TCGA-CA-5255 | A |
| TCGA_TCGA-A6-A5ZU | C |
| TCGA_TCGA-NH-A50V | C |
| TCGA_TCGA-QG-A5YX | B |
| TCGA_TCGA-A6-2677 | B |
| TCGA_TCGA-CA-6717 | C |
| TCGA_TCGA-A6-6649 | C |
| TCGA_TCGA-NH-A6GC | C |
| TCGA_TCGA-AA-3562 | B |
| TCGA_TCGA-A6-4105 | B |
| TCGA_TCGA-A6-6141 | B |
| TCGA_TCGA-AA-A00R | A |
| TCGA_TCGA-AZ-4684 | C |
| TCGA_TCGA-CK-6747 | B |
| TCGA_TCGA-AU-6004 | A |
| TCGA_TCGA-A6-2672 | A |
| TCGA_TCGA-QG-A5Z2 | B |
| TCGA_TCGA-A6-6780 | B |
| TCGA_TCGA-F4-6459 | C |
| TCGA_TCGA-AA-3970 | B |
| TCGA_TCGA-NH-A8F7 | B |
| TCGA_TCGA-G4-6321 | B |
| TCGA_TCGA-AY-6196 | C |
| TCGA_TCGA-AA-3524 | B |
| TCGA_TCGA-DM-A1D0 | B |
| TCGA_TCGA-G4-6307 | B |
| TCGA_TCGA-DM-A1HB | A |
| TCGA_TCGA-AA-3697 | B |
| TCGA_TCGA-CM-6679 | C |
| TCGA_TCGA-AY-6197 | A |
| TCGA_TCGA-AA-3672 | C |
| TCGA_TCGA-AA-3684 | C |
| TCGA_TCGA-A6-2684 | C |
| TCGA_TCGA-AZ-4323 | C |
| TCGA_TCGA-AA-3971 | B |
| TCGA_TCGA-AA-3989 | C |
| TCGA_TCGA-AA-A00D | A |
| TCGA_TCGA-AD-6548 | B |
| TCGA_TCGA-D5-6539 | B |
| TCGA_TCGA-G4-6302 | C |
| TCGA_TCGA-A6-2679 | C |
| TCGA_TCGA-A6-2674 | C |
| TCGA_TCGA-AZ-4614 | A |
| TCGA_TCGA-NH-A5IV | A |
| TCGA_TCGA-A6-5664 | C |
| TCGA_TCGA-AZ-4315 | A |
| TCGA_TCGA-F4-6806 | B |
| TCGA_TCGA-AA-3510 | B |
| TCGA_TCGA-AY-A71X | B |
| TCGA_TCGA-AA-3488 | B |
| TCGA_TCGA-DM-A28F | B |
| TCGA_TCGA-G4-6293 | B |
| TCGA_TCGA-5M-AAT4 | B |
| TCGA_TCGA-D5-6533 | B |
| TCGA_TCGA-A6-5657 | C |
| TCGA_TCGA-AA-3941 | A |
| TCGA_TCGA-DM-A28K | A |
| TCGA_TCGA-CM-5344 | C |
| TCGA_TCGA-AA-3517 | B |
| TCGA_TCGA-G4-6309 | A |
| TCGA_TCGA-AY-6386 | B |
| TCGA_TCGA-AA-3678 | B |
| TCGA_TCGA-AA-A01X | C |
| TCGA_TCGA-AA-3821 | A |
| TCGA_TCGA-CM-6677 | B |
| TCGA_TCGA-CM-5861 | A |
| TCGA_TCGA-D5-6923 | C |
| TCGA_TCGA-F4-6703 | C |
| TCGA_TCGA-AZ-6605 | C |
| TCGA_TCGA-NH-A6GB | A |
| TCGA_TCGA-CM-5349 | C |
| TCGA_TCGA-NH-A50U | B |
| TCGA_TCGA-AA-3530 | B |
| TCGA_TCGA-G4-6627 | C |
| TCGA_TCGA-DM-A28G | B |
| TCGA_TCGA-D5-6537 | B |
| TCGA_TCGA-D5-5539 | C |
| TCGA_TCGA-CM-6163 | B |
| TCGA_TCGA-CM-4744 | A |
| TCGA_TCGA-AA-A004 | C |
| TCGA_TCGA-A6-3808 | C |
| TCGA_TCGA-AA-A010 | B |
| TCGA_TCGA-AA-A02J | B |
| TCGA_TCGA-AA-3837 | C |
| TCGA_TCGA-CK-4947 | B |
| TCGA_TCGA-CA-5256 | B |
| TCGA_TCGA-CM-6167 | C |
| TCGA_TCGA-4N-A93T | B |
| TCGA_TCGA-AA-3521 | B |
| TCGA_TCGA-CA-5797 | B |
| TCGA_TCGA-CM-6166 | B |
| TCGA_TCGA-CK-4951 | A |
| TCGA_TCGA-CA-6719 | C |
| TCGA_TCGA-AA-A02R | A |
| TCGA_TCGA-AA-A02O | C |
| TCGA_TCGA-A6-5666 | B |
| TCGA_TCGA-AY-5543 | B |
| TCGA_TCGA-AA-3845 | A |
| TCGA_TCGA-F4-6704 | C |
| TCGA_TCGA-AA-3856 | B |
| TCGA_TCGA-AA-A00Q | B |
| TCGA_TCGA-A6-6781 | C |
| TCGA_TCGA-AA-A00F | C |
| TCGA_TCGA-D5-6920 | B |
| TCGA_TCGA-AA-3815 | A |
| TCGA_TCGA-D5-5538 | B |
| TCGA_TCGA-AA-3867 | C |
| TCGA_TCGA-A6-6782 | C |
| TCGA_TCGA-A6-6654 | C |
| TCGA_TCGA-AA-A01D | C |
| TCGA_TCGA-G4-6311 | C |
| TCGA_TCGA-A6-3807 | C |
| TCGA_TCGA-AZ-5403 | B |
| TCGA_TCGA-AD-6888 | B |
| TCGA_TCGA-G4-6628 | A |
| TCGA_TCGA-DM-A1D6 | B |
| TCGA_TCGA-CM-5341 | B |
| TCGA_TCGA-AA-3862 | B |
| TCGA_TCGA-AA-3939 | B |
| TCGA_TCGA-AA-3514 | C |
| TCGA_TCGA-AA-3660 | B |
| TCGA_TCGA-AA-3833 | A |
| TCGA_TCGA-AA-A01P | A |
| TCGA_TCGA-AA-A00Z | B |
| TCGA_TCGA-CM-5863 | C |
| TCGA_TCGA-AZ-6598 | A |
| TCGA_TCGA-AA-3679 | B |
| TCGA_TCGA-G4-6304 | B |
| TCGA_TCGA-DM-A285 | A |
| TCGA_TCGA-G4-6294 | B |
| TCGA_TCGA-A6-4107 | C |
| TCGA_TCGA-DM-A1D4 | B |
| TCGA_TCGA-CA-6716 | B |
| TCGA_TCGA-CK-4952 | A |
| TCGA_TCGA-AA-A01V | B |
| TCGA_TCGA-AA-3844 | B |
| TCGA_TCGA-D5-6541 | C |
| TCGA_TCGA-AA-A01G | B |
| TCGA_TCGA-AA-3870 | A |
| TCGA_TCGA-D5-6532 | B |
| TCGA_TCGA-CM-6169 | C |
| TCGA_TCGA-D5-5541 | B |
| TCGA_TCGA-DM-A1DB | B |
| TCGA_TCGA-AA-A00A | B |
| TCGA_TCGA-D5-6530 | B |
| TCGA_TCGA-AA-3855 | B |
| TCGA_TCGA-CM-5868 | B |
| TCGA_TCGA-AA-3519 | B |
| TCGA_TCGA-AA-3526 | B |
| TCGA_TCGA-DM-A28M | B |
| TCGA_TCGA-D5-6926 | C |
| TCGA_TCGA-AA-3534 | B |
| TCGA_TCGA-G4-6317 | B |
| TCGA_TCGA-AA-A03J | C |
| TCGA_TCGA-CM-6680 | C |
| TCGA_TCGA-AA-A01K | C |
| TCGA_TCGA-AA-3984 | B |
| TCGA_TCGA-D5-6536 | C |
| TCGA_TCGA-AD-A5EK | B |
| TCGA_TCGA-AA-3663 | A |
| TCGA_TCGA-AZ-6607 | C |
| TCGA_TCGA-AA-3520 | C |
| TCGA_TCGA-AA-3543 | A |
| TCGA_TCGA-AZ-5407 | B |
| TCGA_TCGA-G4-6314 | C |
| TCGA_TCGA-CM-5348 | C |
| TCGA_TCGA-CK-6751 | B |
| TCGA_TCGA-D5-6929 | C |
| TCGA_TCGA-AA-3675 | B |
| TCGA_TCGA-A6-2681 | C |
| TCGA_TCGA-AA-3869 | B |
| TCGA_TCGA-DM-A282 | B |
| TCGA_TCGA-D5-7000 | A |
| TCGA_TCGA-AM-5821 | A |
| TCGA_TCGA-G4-6298 | C |
| TCGA_TCGA-AA-A01S | B |
| TCGA_TCGA-NH-A8F8 | C |
| TCGA_TCGA-AA-3966 | A |
| TCGA_TCGA-AA-3516 | A |
| TCGA_TCGA-A6-2676 | A |
| TCGA_TCGA-AA-3846 | B |
| TCGA_TCGA-AA-3544 | C |
| TCGA_TCGA-AA-3956 | B |
| TCGA_TCGA-AA-3532 | B |
| TCGA_TCGA-AA-3955 | B |
| TCGA_TCGA-CK-5912 | B |
| TCGA_TCGA-AD-6895 | A |
| TCGA_TCGA-AA-3556 | A |
| TCGA_TCGA-CM-6678 | B |
| TCGA_TCGA-AA-3851 | B |
| TCGA_TCGA-CM-5860 | C |
| TCGA_TCGA-A6-5661 | A |
| TCGA_TCGA-AA-A02F | B |
| TCGA_TCGA-AA-3861 | B |
| TCGA_TCGA-DM-A1DA | A |
| TCGA_TCGA-CM-6162 | C |
| TCGA_TCGA-AA-3522 | B |
| TCGA_TCGA-AA-A00U | B |
| TCGA_TCGA-F4-6807 | C |
| TCGA_TCGA-AA-3930 | A |
| TCGA_TCGA-AZ-6599 | B |
| TCGA_TCGA-AA-3947 | B |
| TCGA_TCGA-AA-3841 | C |
| TCGA_TCGA-CM-6171 | A |
| TCGA_TCGA-CM-4743 | A |
| TCGA_TCGA-AA-3814 | C |
| TCGA_TCGA-NH-A6GA | A |
| TCGA_TCGA-A6-2675 | C |
| TCGA_TCGA-G4-6297 | C |
| TCGA_TCGA-A6-5656 | B |
| TCGA_TCGA-DM-A0X9 | B |
| TCGA_TCGA-AA-3553 | B |
| TCGA_TCGA-DM-A0XD | A |
| TCGA_TCGA-AA-3511 | B |
| TCGA_TCGA-A6-A56B | C |
| TCGA_TCGA-A6-5667 | B |
| TCGA_TCGA-AA-3866 | B |
| TCGA_TCGA-AA-A017 | C |
| TCGA_TCGA-CM-6674 | A |
| TCGA_TCGA-DM-A1D8 | B |
| TCGA_TCGA-CM-6170 | B |
| TCGA_TCGA-AZ-4616 | A |
| TCGA_TCGA-AA-3994 | A |
| TCGA_TCGA-AA-3877 | A |
| TCGA_TCGA-WS-AB45 | C |
| TCGA_TCGA-AA-A00L | B |
| TCGA_TCGA-CM-5864 | B |
| TCGA_TCGA-CA-5254 | A |
| TCGA_TCGA-D5-6898 | C |
| TCGA_TCGA-AA-3696 | B |
| TCGA_TCGA-DM-A280 | A |
| TCGA_TCGA-AA-A00O | C |
| TCGA_TCGA-QG-A5YW | B |
| TCGA_TCGA-AD-6965 | B |
| TCGA_TCGA-CK-4948 | C |
| TCGA_TCGA-DM-A1HA | A |
| TCGA_TCGA-5M-AAT5 | B |
| TCGA_TCGA-F4-6809 | C |
| TCGA_TCGA-CM-4748 | C |
| TCGA_TCGA-AA-3858 | C |
| TCGA_TCGA-D5-6531 | A |
| TCGA_TCGA-AA-3662 | B |
| TCGA_TCGA-AM-5820 | B |
| TCGA_TCGA-AA-A02K | B |
| TCGA_TCGA-G4-6310 | C |
| TCGA_TCGA-AD-6889 | A |
| TCGA_TCGA-AA-3872 | C |
| TCGA_TCGA-AA-A01R | A |
| TCGA_TCGA-AA-3975 | C |
| TCGA_TCGA-5M-AAT6 | A |
| TCGA_TCGA-D5-6931 | A |
| TCGA_TCGA-D5-6924 | C |
| TCGA_TCGA-AA-3860 | C |
| TCGA_TCGA-A6-5665 | A |
| TCGA_TCGA-5M-AATA | B |
| TCGA_TCGA-D5-6540 | A |
| TCGA_TCGA-CA-6718 | A |
| TCGA_TCGA-AA-3986 | B |
| TCGA_TCGA-G4-6323 | B |
| TCGA_TCGA-A6-A566 | C |
| TCGA_TCGA-AZ-6608 | B |
| TCGA_TCGA-AA-3712 | B |
| TCGA_TCGA-A6-5662 | C |
| TCGA_TCGA-AA-3831 | B |
| TCGA_TCGA-AA-A02H | B |
| TCGA_TCGA-CM-6676 | B |
| TCGA_TCGA-AD-6899 | C |
| TCGA_TCGA-AA-3685 | B |
| TCGA_TCGA-CK-5915 | B |
| TCGA_TCGA-AA-3548 | C |
| TCGA_TCGA-F4-6569 | C |
| TCGA_TCGA-F4-6461 | C |
| TCGA_TCGA-F4-6854 | B |
| TCGA_TCGA-F4-6856 | A |
| TCGA_TCGA-AY-A54L | B |
| TCGA_TCGA-AA-3538 | B |
| TCGA_TCGA-AA-3842 | B |
| TCGA_TCGA-AZ-4308 | C |
| TCGA_TCGA-CK-6746 | A |
| TCGA_TCGA-AA-3509 | B |
| TCGA_TCGA-AA-3680 | B |
| TCGA_TCGA-AA-3542 | C |
| TCGA_TCGA-AD-6964 | C |
| TCGA_TCGA-A6-6650 | B |
| TCGA_TCGA-A6-6140 | B |
| TCGA_TCGA-AA-A024 | B |
| TCGA_TCGA-A6-2682 | C |
| TCGA_TCGA-CM-6675 | A |
| TCGA_TCGA-DM-A1D7 | B |
| TCGA_TCGA-4T-AA8H | B |
| TCGA_TCGA-A6-6653 | A |
| TCGA_TCGA-AA-3666 | B |
| TCGA_TCGA-F4-6460 | C |
| TCGA_TCGA-AA-3655 | B |
| TCGA_TCGA-AA-3673 | B |
| TCGA_TCGA-AA-3952 | C |
| TCGA_TCGA-T9-A92H | B |
| TCGA_TCGA-AY-4070 | C |
| TCGA_TCGA-AY-A8YK | B |
| TCGA_TCGA-CK-4950 | B |
| TCGA_TCGA-F4-6570 | A |
| TCGA_TCGA-AZ-6600 | C |
| TCGA_TCGA-AA-A00J | A |
| TCGA_TCGA-AA-3667 | C |
| TCGA_TCGA-AA-3554 | A |
| TCGA_TCGA-AZ-6606 | B |
| TCGA_TCGA-AA-3852 | C |
| TCGA_TCGA-D5-6922 | C |
| TCGA_TCGA-A6-6137 | B |
| TCGA_TCGA-AA-3495 | B |
| TCGA_TCGA-AA-3489 | C |
| TCGA_TCGA-G4-6322 | B |
| TCGA_TCGA-SS-A7HO | B |
| TCGA_TCGA-AA-3976 | B |
| TCGA_TCGA-AA-3811 | A |
| TCGA_TCGA-AD-A5EJ | A |
| TCGA_TCGA-AA-3819 | B |
| TCGA_TCGA-AA-A02Y | B |
| TCGA_TCGA-AZ-6603 | B |
| TCGA_TCGA-AA-A022 | A |
| TCGA_TCGA-G4-6626 | B |
| TCGA_TCGA-AA-3875 | B |
| TCGA_TCGA-G4-6306 | B |
| TCGA_TCGA-G4-6320 | B |
| TCGA_TCGA-CK-6748 | C |
| TCGA_TCGA-AA-3864 | A |
| TCGA_TCGA-A6-6142 | C |
| TCGA_TCGA-A6-2678 | B |
| TCGA_TCGA-CA-5796 | B |
| TCGA_TCGA-A6-A567 | B |
| TCGA_TCGA-AA-3713 | A |
| TCGA_TCGA-A6-6648 | B |
| TCGA_TCGA-A6-A565 | C |
| TCGA_TCGA-A6-5660 | B |
| TCGA_TCGA-AZ-6601 | A |
| TCGA_TCGA-5M-AATE | B |
| TCGA_TCGA-CM-6161 | B |
| TCGA_TCGA-AA-A01Q | A |
| TCGA_TCGA-AZ-4313 | A |
| TCGA_TCGA-A6-6652 | B |
| TCGA_TCGA-AA-A00E | A |
| TCGA_TCGA-F4-6808 | B |
| TCGA_TCGA-AA-A02W | B |
| TCGA_TCGA-DM-A28C | B |
| TCGA_TCGA-D5-6927 | A |
| TCGA_TCGA-AA-3980 | B |
| TCGA_TCGA-AA-3681 | A |
| TCGA_TCGA-AA-3492 | A |
| TCGA_TCGA-A6-2671 | C |
| TCGA_TCGA-D5-6535 | B |
| TCGA_TCGA-AA-A029 | A |
| TCGA_TCGA-AD-6963 | B |
| TCGA_TCGA-CM-4747 | C |
| TCGA_TCGA-NH-A50T | B |
| TCGA_TCGA-CM-6168 | C |
| TCGA_TCGA-AA-3518 | A |
| TCGA_TCGA-AA-A01T | B |
| TCGA_TCGA-RU-A8FL | B |
| TCGA_TCGA-A6-6651 | C |
| TCGA_TCGA-AA-3850 | B |
| TCGA_TCGA-AA-3972 | B |
| TCGA_TCGA-AA-3812 | C |
| TCGA_TCGA-AY-A69D | B |
| TCGA_TCGA-AA-A01Z | B |
| TCGA_TCGA-A6-3810 | B |
| TCGA_TCGA-DM-A28H | B |
| TCGA_TCGA-G4-6586 | A |
| TCGA_TCGA-D5-6928 | C |
| TCGA_TCGA-G4-6315 | B |
| TCGA_TCGA-D5-6534 | C |
| TCGA_TCGA-AA-3664 | B |
| TCGA_TCGA-G4-6303 | B |
| TCGA_TCGA-AA-3692 | B |
| TCGA_TCGA-AA-3968 | B |
| TCGA_TCGA-F4-6805 | C |
| TCGA_TCGA-AA-3560 | B |
| TCGA_TCGA-D5-5537 | B |
| TCGA_TCGA-CM-5862 | B |
| TCGA_TCGA-D5-6932 | C |
| TCGA_TCGA-AA-3982 | B |
| TCGA_TCGA-DM-A28A | B |
| TCGA_TCGA-CM-6172 | B |
| TCGA_TCGA-D5-6930 | A |
| TCGA_TCGA-D5-6529 | C |
| TCGA_TCGA-A6-2683 | C |
| TCGA_TCGA-CK-5913 | A |
| TCGA_TCGA-AA-3977 | B |
| TCGA_TCGA-AA-3496 | C |
| TCGA_TCGA-D5-5540 | B |
| TCGA_TCGA-AD-6901 | C |
| TCGA_TCGA-AA-A01F | B |
| TCGA_TCGA-AA-3552 | C |
| TCGA_TCGA-F4-6855 | C |
| TCGA_TCGA-AD-5900 | A |
| TCGA_TCGA-AA-3979 | B |
| TCGA_TCGA-AA-A01I | B |
| TCGA_TCGA-AA-A03F | A |
| TCGA_TCGA-3L-AA1B | C |
| TCGA_TCGA-AA-3693 | B |
| TCGA_TCGA-AA-3502 | B |
| TCGA_TCGA-AA-A02E | B |
| TCGA_TCGA-CM-4746 | B |
| TCGA_TCGA-G4-6295 | B |
| TCGA_TCGA-AA-A01C | C |
| TCGA_TCGA-G4-6588 | A |
| TCGA_TCGA-A6-2680 | C |
| TCGA_TCGA-QG-A5YV | B |
| TCGA_TCGA-AA-3561 | B |
| TCGA_TCGA-AA-3525 | A |
| TCGA_TCGA-AA-3531 | B |
| TCGA_TCGA-AA-3973 | C |
| TCGA_TCGA-AA-3527 | C |
| TCGA_TCGA-AA-3555 | A |
| TCGA_TCGA-AG-A025 | B |
| TCGA_TCGA-EI-6884 | B |
| TCGA_TCGA-AG-3582 | B |
| TCGA_TCGA-DC-6154 | B |
| TCGA_TCGA-AG-3611 | B |
| TCGA_TCGA-DY-A1DD | C |
| TCGA_TCGA-AG-3609 | C |
| TCGA_TCGA-AG-A01L | B |
| TCGA_TCGA-AG-4022 | C |
| TCGA_TCGA-AG-A01W | B |
| TCGA_TCGA-AG-A02N | B |
| TCGA_TCGA-AH-6549 | B |
| TCGA_TCGA-AH-6903 | B |
| TCGA_TCGA-AG-3727 | C |
| TCGA_TCGA-F5-6863 | B |
| TCGA_TCGA-AG-3887 | B |
| TCGA_TCGA-AG-A01J | B |
| TCGA_TCGA-AF-5654 | B |
| TCGA_TCGA-DY-A1DE | B |
| TCGA_TCGA-AF-3913 | C |
| TCGA_TCGA-AG-A014 | A |
| TCGA_TCGA-AG-4015 | B |
| TCGA_TCGA-G5-6572 | C |
| TCGA_TCGA-AG-A011 | B |
| TCGA_TCGA-EI-6511 | B |
| TCGA_TCGA-AG-4008 | C |
| TCGA_TCGA-AG-3731 | C |
| TCGA_TCGA-AG-A032 | B |
| TCGA_TCGA-AH-6544 | B |
| TCGA_TCGA-AG-A020 | B |
| TCGA_TCGA-AG-A026 | C |
| TCGA_TCGA-DY-A1H8 | B |
| TCGA_TCGA-AG-3883 | C |
| TCGA_TCGA-EI-7004 | C |
| TCGA_TCGA-AF-6136 | B |
| TCGA_TCGA-DC-4745 | B |
| TCGA_TCGA-EI-6883 | B |
| TCGA_TCGA-EI-6917 | A |
| TCGA_TCGA-F5-6861 | B |
| TCGA_TCGA-EI-6508 | B |
| TCGA_TCGA-F5-6465 | C |
| TCGA_TCGA-AF-6672 | B |
| TCGA_TCGA-AG-3885 | C |
| TCGA_TCGA-EI-6509 | C |
| TCGA_TCGA-DC-5337 | B |
| TCGA_TCGA-AG-3893 | C |
| TCGA_TCGA-AG-A02G | B |
| TCGA_TCGA-AG-3901 | C |
| TCGA_TCGA-AG-3726 | C |
| TCGA_TCGA-AG-3896 | B |
| TCGA_TCGA-DC-6160 | B |
| TCGA_TCGA-AG-3581 | C |
| TCGA_TCGA-G5-6641 | B |
| TCGA_TCGA-F5-6812 | C |
| TCGA_TCGA-AG-4001 | C |
| TCGA_TCGA-AF-2687 | C |
| TCGA_TCGA-AH-6644 | C |
| TCGA_TCGA-AG-3586 | B |
| TCGA_TCGA-AG-3909 | C |
| TCGA_TCGA-AG-3878 | C |
| TCGA_TCGA-AG-3592 | B |
| TCGA_TCGA-F5-6814 | B |
| TCGA_TCGA-AG-3605 | B |
| TCGA_TCGA-EI-6513 | B |
| TCGA_TCGA-DC-6158 | C |
| TCGA_TCGA-EI-6512 | B |
| TCGA_TCGA-AG-3601 | B |
| TCGA_TCGA-CI-6622 | B |
| TCGA_TCGA-BM-6198 | C |
| TCGA_TCGA-AG-3881 | C |
| TCGA_TCGA-DY-A1DC | B |
| TCGA_TCGA-AG-3612 | C |
| TCGA_TCGA-AG-3600 | A |
| TCGA_TCGA-AG-3599 | B |
| TCGA_TCGA-AG-3584 | C |
| TCGA_TCGA-DC-6156 | C |
| TCGA_TCGA-DT-5265 | C |
| TCGA_TCGA-AG-3902 | A |
| TCGA_TCGA-AG-A036 | B |
| TCGA_TCGA-AG-3593 | B |
| TCGA_TCGA-AG-A01Y | B |
| TCGA_TCGA-AG-3580 | B |
| TCGA_TCGA-AG-3742 | B |
| TCGA_TCGA-AG-A002 | A |
| TCGA_TCGA-DC-6683 | B |
| TCGA_TCGA-AF-6655 | C |
| TCGA_TCGA-AG-A023 | C |
| TCGA_TCGA-AG-3728 | C |
| TCGA_TCGA-AF-3400 | C |
| TCGA_TCGA-AF-2691 | C |
| TCGA_TCGA-CL-5918 | B |
| TCGA_TCGA-F5-6864 | C |
| TCGA_TCGA-AF-A56K | C |
| TCGA_TCGA-AG-4021 | A |
| TCGA_TCGA-AG-A01N | B |
| TCGA_TCGA-DC-5869 | B |
| TCGA_TCGA-AG-3608 | B |
| TCGA_TCGA-DY-A0XA | B |
| TCGA_TCGA-AG-3594 | A |
| TCGA_TCGA-EF-5831 | B |
| TCGA_TCGA-AG-3725 | B |
| TCGA_TCGA-EI-6514 | B |
| TCGA_TCGA-CI-6624 | B |
| TCGA_TCGA-CI-6620 | B |
| TCGA_TCGA-CI-6621 | B |
| TCGA_TCGA-EI-6510 | B |
| TCGA_TCGA-DY-A1DG | B |
| TCGA_TCGA-AG-A02X | B |
| TCGA_TCGA-AG-3587 | C |
| TCGA_TCGA-AG-3894 | B |
| TCGA_TCGA-AG-3999 | C |
| TCGA_TCGA-AG-A00H | C |
| TCGA_TCGA-AF-A56N | B |
| TCGA_TCGA-AG-A00C | B |
| TCGA_TCGA-EI-6885 | C |
| TCGA_TCGA-AG-3732 | B |
| TCGA_TCGA-AG-4007 | C |
| TCGA_TCGA-AG-A016 | B |
| TCGA_TCGA-EI-6882 | B |
| TCGA_TCGA-AG-3575 | A |
| TCGA_TCGA-AF-2693 | B |
| TCGA_TCGA-DC-6157 | B |
| TCGA_TCGA-AF-4110 | C |
| TCGA_TCGA-AG-3591 | B |
| TCGA_TCGA-EI-6881 | B |
| TCGA_TCGA-AG-A008 | B |
| TCGA_TCGA-AG-A015 | B |
| TCGA_TCGA-AG-3892 | B |
| TCGA_TCGA-G5-6233 | A |
| TCGA_TCGA-AG-3898 | B |
| TCGA_TCGA-AG-3578 | A |
| TCGA_TCGA-DC-6682 | B |
| TCGA_TCGA-F5-6813 | C |
| TCGA_TCGA-F5-6702 | C |
| TCGA_TCGA-CL-5917 | B |
| TCGA_TCGA-CL-4957 | B |
| TCGA_TCGA-EI-6507 | A |
| TCGA_TCGA-DC-4749 | B |
| TCGA_TCGA-AF-2690 | C |
| TCGA_TCGA-EF-5830 | B |
| TCGA_TCGA-AF-3911 | B |
| TCGA_TCGA-DC-6155 | C |
| TCGA_TCGA-DC-6681 | C |
| TCGA_TCGA-AF-A56L | B |
| TCGA_TCGA-CI-6623 | B |
| TCGA_TCGA-AH-6897 | B |
| TCGA_TCGA-AG-3574 | B |
| TCGA_TCGA-G5-6235 | B |
| TCGA_TCGA-AG-4005 | C |
| TCGA_TCGA-AG-3583 | B |
| TCGA_TCGA-AH-6547 | C |
| TCGA_TCGA-EI-7002 | B |
| TCGA_TCGA-AG-A00Y | B |
| TCGA_TCGA-EI-6506 | B |
| TCGA_TCGA-AH-6643 | A |
| TCGA_TCGA-AG-3890 | C |
| TCGA_TCGA-DY-A1DF | C |
| TCGA_TCGA-F5-6571 | C |
| TCGA_TCGA-AG-3882 | C |
| TCGA_TCGA-AG-3598 | B |
| TCGA_TCGA-F5-6811 | C |
| TCGA_TCGA-F5-6810 | C |
| TCGA_TCGA-F5-6464 | C |
| TCGA_TCGA-CI-6619 | B |
| TCGA_TCGA-AG-3602 | B |
| TCGA_TCGA-AF-2692 | B |
| GSE39582_GSM971957 | A |
| GSE39582_GSM971958 | C |
| GSE39582_GSM971959 | A |
| GSE39582_GSM971960 | B |
| GSE39582_GSM971961 | C |
| GSE39582_GSM971962 | C |
| GSE39582_GSM971963 | A |
| GSE39582_GSM971964 | B |
| GSE39582_GSM971965 | B |
| GSE39582_GSM971966 | A |
| GSE39582_GSM971967 | A |
| GSE39582_GSM971968 | A |
| GSE39582_GSM971969 | A |
| GSE39582_GSM971970 | A |
| GSE39582_GSM971971 | B |
| GSE39582_GSM971972 | B |
| GSE39582_GSM971973 | A |
| GSE39582_GSM971974 | C |
| GSE39582_GSM971975 | B |
| GSE39582_GSM971976 | B |
| GSE39582_GSM971977 | C |
| GSE39582_GSM971978 | A |
| GSE39582_GSM971979 | A |
| GSE39582_GSM971980 | A |
| GSE39582_GSM971981 | A |
| GSE39582_GSM971982 | A |
| GSE39582_GSM971983 | A |
| GSE39582_GSM971984 | A |
| GSE39582_GSM971985 | B |
| GSE39582_GSM971986 | C |
| GSE39582_GSM971987 | C |
| GSE39582_GSM971988 | A |
| GSE39582_GSM971989 | A |
| GSE39582_GSM971990 | B |
| GSE39582_GSM971991 | B |
| GSE39582_GSM971992 | B |
| GSE39582_GSM971993 | B |
| GSE39582_GSM971994 | B |
| GSE39582_GSM971995 | B |
| GSE39582_GSM971996 | C |
| GSE39582_GSM971997 | A |
| GSE39582_GSM971998 | B |
| GSE39582_GSM971999 | B |
| GSE39582_GSM972000 | B |
| GSE39582_GSM972001 | B |
| GSE39582_GSM972002 | A |
| GSE39582_GSM972003 | B |
| GSE39582_GSM972004 | B |
| GSE39582_GSM972005 | B |
| GSE39582_GSM972006 | C |
| GSE39582_GSM972007 | C |
| GSE39582_GSM972008 | A |
| GSE39582_GSM972009 | C |
| GSE39582_GSM972010 | A |
| GSE39582_GSM972011 | C |
| GSE39582_GSM972012 | B |
| GSE39582_GSM972013 | B |
| GSE39582_GSM972014 | B |
| GSE39582_GSM972015 | A |
| GSE39582_GSM972016 | C |
| GSE39582_GSM972017 | B |
| GSE39582_GSM972018 | B |
| GSE39582_GSM972019 | C |
| GSE39582_GSM972020 | B |
| GSE39582_GSM972021 | A |
| GSE39582_GSM972022 | B |
| GSE39582_GSM972023 | B |
| GSE39582_GSM972024 | B |
| GSE39582_GSM972025 | B |
| GSE39582_GSM972026 | A |
| GSE39582_GSM972027 | B |
| GSE39582_GSM972028 | C |
| GSE39582_GSM972029 | C |
| GSE39582_GSM972030 | C |
| GSE39582_GSM972031 | B |
| GSE39582_GSM972032 | B |
| GSE39582_GSM972033 | B |
| GSE39582_GSM972034 | B |
| GSE39582_GSM972035 | B |
| GSE39582_GSM972036 | B |
| GSE39582_GSM972037 | B |
| GSE39582_GSM972038 | B |
| GSE39582_GSM972039 | C |
| GSE39582_GSM972040 | B |
| GSE39582_GSM972041 | B |
| GSE39582_GSM972042 | B |
| GSE39582_GSM972043 | B |
| GSE39582_GSM972044 | B |
| GSE39582_GSM972045 | B |
| GSE39582_GSM972046 | C |
| GSE39582_GSM972047 | B |
| GSE39582_GSM972048 | B |
| GSE39582_GSM972049 | A |
| GSE39582_GSM972050 | B |
| GSE39582_GSM972051 | C |
| GSE39582_GSM972052 | B |
| GSE39582_GSM972053 | B |
| GSE39582_GSM972054 | C |
| GSE39582_GSM972055 | C |
| GSE39582_GSM972056 | A |
| GSE39582_GSM972057 | B |
| GSE39582_GSM972058 | A |
| GSE39582_GSM972059 | C |
| GSE39582_GSM972060 | B |
| GSE39582_GSM972061 | C |
| GSE39582_GSM972062 | B |
| GSE39582_GSM972063 | B |
| GSE39582_GSM972064 | C |
| GSE39582_GSM972065 | B |
| GSE39582_GSM972066 | B |
| GSE39582_GSM972067 | C |
| GSE39582_GSM972068 | C |
| GSE39582_GSM972069 | B |
| GSE39582_GSM972070 | B |
| GSE39582_GSM972071 | B |
| GSE39582_GSM972072 | A |
| GSE39582_GSM972073 | C |
| GSE39582_GSM972074 | B |
| GSE39582_GSM972075 | B |
| GSE39582_GSM972076 | A |
| GSE39582_GSM972077 | C |
| GSE39582_GSM972078 | C |
| GSE39582_GSM972079 | C |
| GSE39582_GSM972080 | A |
| GSE39582_GSM972081 | B |
| GSE39582_GSM972082 | C |
| GSE39582_GSM972083 | B |
| GSE39582_GSM972084 | C |
| GSE39582_GSM972085 | B |
| GSE39582_GSM972086 | B |
| GSE39582_GSM972087 | B |
| GSE39582_GSM972088 | C |
| GSE39582_GSM972089 | C |
| GSE39582_GSM972090 | B |
| GSE39582_GSM972091 | B |
| GSE39582_GSM972092 | C |
| GSE39582_GSM972093 | C |
| GSE39582_GSM972094 | B |
| GSE39582_GSM972095 | B |
| GSE39582_GSM972096 | B |
| GSE39582_GSM972097 | C |
| GSE39582_GSM972098 | B |
| GSE39582_GSM972099 | B |
| GSE39582_GSM972100 | A |
| GSE39582_GSM972101 | B |
| GSE39582_GSM972102 | C |
| GSE39582_GSM972103 | B |
| GSE39582_GSM972104 | C |
| GSE39582_GSM972105 | C |
| GSE39582_GSM972106 | B |
| GSE39582_GSM972107 | A |
| GSE39582_GSM972108 | B |
| GSE39582_GSM972109 | B |
| GSE39582_GSM972110 | B |
| GSE39582_GSM972111 | C |
| GSE39582_GSM972112 | C |
| GSE39582_GSM972113 | C |
| GSE39582_GSM972114 | B |
| GSE39582_GSM972115 | B |
| GSE39582_GSM972116 | C |
| GSE39582_GSM972117 | B |
| GSE39582_GSM972118 | B |
| GSE39582_GSM972119 | B |
| GSE39582_GSM972120 | A |
| GSE39582_GSM972121 | B |
| GSE39582_GSM972122 | B |
| GSE39582_GSM972123 | B |
| GSE39582_GSM972124 | B |
| GSE39582_GSM972125 | C |
| GSE39582_GSM972126 | B |
| GSE39582_GSM972127 | C |
| GSE39582_GSM972128 | B |
| GSE39582_GSM972129 | A |
| GSE39582_GSM972130 | B |
| GSE39582_GSM972131 | C |
| GSE39582_GSM972132 | B |
| GSE39582_GSM972133 | C |
| GSE39582_GSM972134 | B |
| GSE39582_GSM972135 | B |
| GSE39582_GSM972136 | B |
| GSE39582_GSM972137 | B |
| GSE39582_GSM972138 | B |
| GSE39582_GSM972139 | B |
| GSE39582_GSM972140 | C |
| GSE39582_GSM972141 | B |
| GSE39582_GSM972142 | B |
| GSE39582_GSM972143 | B |
| GSE39582_GSM972144 | B |
| GSE39582_GSM972145 | B |
| GSE39582_GSM972146 | C |
| GSE39582_GSM972147 | B |
| GSE39582_GSM972148 | B |
| GSE39582_GSM972149 | B |
| GSE39582_GSM972150 | B |
| GSE39582_GSM972151 | B |
| GSE39582_GSM972152 | B |
| GSE39582_GSM972153 | B |
| GSE39582_GSM972154 | B |
| GSE39582_GSM972155 | B |
| GSE39582_GSM972156 | B |
| GSE39582_GSM972157 | B |
| GSE39582_GSM972158 | C |
| GSE39582_GSM972159 | B |
| GSE39582_GSM972160 | B |
| GSE39582_GSM972161 | B |
| GSE39582_GSM972162 | B |
| GSE39582_GSM972163 | B |
| GSE39582_GSM972164 | B |
| GSE39582_GSM972165 | B |
| GSE39582_GSM972166 | B |
| GSE39582_GSM972167 | B |
| GSE39582_GSM972168 | A |
| GSE39582_GSM972169 | B |
| GSE39582_GSM972170 | B |
| GSE39582_GSM972171 | A |
| GSE39582_GSM972172 | A |
| GSE39582_GSM972173 | C |
| GSE39582_GSM972174 | B |
| GSE39582_GSM972175 | B |
| GSE39582_GSM972176 | C |
| GSE39582_GSM972177 | C |
| GSE39582_GSM972178 | B |
| GSE39582_GSM972179 | C |
| GSE39582_GSM972180 | A |
| GSE39582_GSM972181 | C |
| GSE39582_GSM972182 | B |
| GSE39582_GSM972183 | B |
| GSE39582_GSM972184 | A |
| GSE39582_GSM972185 | B |
| GSE39582_GSM972186 | C |
| GSE39582_GSM972187 | C |
| GSE39582_GSM972188 | B |
| GSE39582_GSM972189 | B |
| GSE39582_GSM972190 | B |
| GSE39582_GSM972191 | B |
| GSE39582_GSM972192 | C |
| GSE39582_GSM972193 | C |
| GSE39582_GSM972194 | B |
| GSE39582_GSM972195 | B |
| GSE39582_GSM972196 | A |
| GSE39582_GSM972197 | A |
| GSE39582_GSM972198 | B |
| GSE39582_GSM972199 | B |
| GSE39582_GSM972200 | B |
| GSE39582_GSM972201 | C |
| GSE39582_GSM972202 | B |
| GSE39582_GSM972203 | B |
| GSE39582_GSM972204 | B |
| GSE39582_GSM972205 | B |
| GSE39582_GSM972206 | B |
| GSE39582_GSM972207 | B |
| GSE39582_GSM972208 | B |
| GSE39582_GSM972209 | A |
| GSE39582_GSM972210 | B |
| GSE39582_GSM972211 | B |
| GSE39582_GSM972212 | B |
| GSE39582_GSM972213 | A |
| GSE39582_GSM972214 | A |
| GSE39582_GSM972215 | B |
| GSE39582_GSM972216 | B |
| GSE39582_GSM972217 | B |
| GSE39582_GSM972218 | B |
| GSE39582_GSM972219 | B |
| GSE39582_GSM972220 | A |
| GSE39582_GSM972221 | A |
| GSE39582_GSM972222 | B |
| GSE39582_GSM972223 | A |
| GSE39582_GSM972224 | A |
| GSE39582_GSM972225 | B |
| GSE39582_GSM972226 | A |
| GSE39582_GSM972227 | B |
| GSE39582_GSM972228 | B |
| GSE39582_GSM972229 | A |
| GSE39582_GSM972230 | B |
| GSE39582_GSM972231 | C |
| GSE39582_GSM972232 | C |
| GSE39582_GSM972233 | B |
| GSE39582_GSM972234 | C |
| GSE39582_GSM972235 | B |
| GSE39582_GSM972236 | B |
| GSE39582_GSM972237 | B |
| GSE39582_GSM972238 | B |
| GSE39582_GSM972239 | B |
| GSE39582_GSM972240 | C |
| GSE39582_GSM972241 | B |
| GSE39582_GSM972242 | B |
| GSE39582_GSM972243 | B |
| GSE39582_GSM972244 | C |
| GSE39582_GSM972245 | B |
| GSE39582_GSM972246 | B |
| GSE39582_GSM972247 | B |
| GSE39582_GSM972248 | B |
| GSE39582_GSM972249 | B |
| GSE39582_GSM972250 | B |
| GSE39582_GSM972251 | C |
| GSE39582_GSM972252 | B |
| GSE39582_GSM972253 | B |
| GSE39582_GSM972254 | C |
| GSE39582_GSM972255 | C |
| GSE39582_GSM972256 | C |
| GSE39582_GSM972257 | C |
| GSE39582_GSM972258 | C |
| GSE39582_GSM972259 | C |
| GSE39582_GSM972260 | C |
| GSE39582_GSM972261 | A |
| GSE39582_GSM972262 | C |
| GSE39582_GSM972263 | A |
| GSE39582_GSM972264 | B |
| GSE39582_GSM972265 | A |
| GSE39582_GSM972266 | B |
| GSE39582_GSM972267 | B |
| GSE39582_GSM972268 | B |
| GSE39582_GSM972269 | A |
| GSE39582_GSM972270 | A |
| GSE39582_GSM972271 | B |
| GSE39582_GSM972272 | B |
| GSE39582_GSM972273 | B |
| GSE39582_GSM972274 | A |
| GSE39582_GSM972275 | A |
| GSE39582_GSM972276 | A |
| GSE39582_GSM972277 | A |
| GSE39582_GSM972278 | A |
| GSE39582_GSM972279 | A |
| GSE39582_GSM972280 | A |
| GSE39582_GSM972281 | B |
| GSE39582_GSM972282 | B |
| GSE39582_GSM972283 | A |
| GSE39582_GSM972284 | B |
| GSE39582_GSM972285 | B |
| GSE39582_GSM972286 | A |
| GSE39582_GSM972287 | A |
| GSE39582_GSM972288 | C |
| GSE39582_GSM972289 | B |
| GSE39582_GSM972290 | B |
| GSE39582_GSM972291 | B |
| GSE39582_GSM972292 | A |
| GSE39582_GSM972293 | C |
| GSE39582_GSM972294 | A |
| GSE39582_GSM972295 | B |
| GSE39582_GSM972296 | B |
| GSE39582_GSM972297 | C |
| GSE39582_GSM972298 | A |
| GSE39582_GSM972299 | B |
| GSE39582_GSM972300 | B |
| GSE39582_GSM972301 | C |
| GSE39582_GSM972302 | B |
| GSE39582_GSM972303 | B |
| GSE39582_GSM972304 | B |
| GSE39582_GSM972305 | C |
| GSE39582_GSM972306 | B |
| GSE39582_GSM972307 | B |
| GSE39582_GSM972308 | A |
| GSE39582_GSM972309 | B |
| GSE39582_GSM972310 | B |
| GSE39582_GSM972311 | C |
| GSE39582_GSM972312 | B |
| GSE39582_GSM972313 | B |
| GSE39582_GSM972314 | A |
| GSE39582_GSM972315 | B |
| GSE39582_GSM972316 | A |
| GSE39582_GSM972317 | A |
| GSE39582_GSM972318 | B |
| GSE39582_GSM972319 | B |
| GSE39582_GSM972320 | B |
| GSE39582_GSM972321 | B |
| GSE39582_GSM972322 | B |
| GSE39582_GSM972323 | B |
| GSE39582_GSM972324 | B |
| GSE39582_GSM972325 | A |
| GSE39582_GSM972326 | B |
| GSE39582_GSM972327 | C |
| GSE39582_GSM972328 | B |
| GSE39582_GSM972329 | B |
| GSE39582_GSM972330 | A |
| GSE39582_GSM972331 | B |
| GSE39582_GSM972332 | A |
| GSE39582_GSM972333 | B |
| GSE39582_GSM972334 | A |
| GSE39582_GSM972335 | B |
| GSE39582_GSM972336 | B |
| GSE39582_GSM972337 | A |
| GSE39582_GSM972338 | B |
| GSE39582_GSM972339 | B |
| GSE39582_GSM972340 | B |
| GSE39582_GSM972341 | B |
| GSE39582_GSM972342 | B |
| GSE39582_GSM972343 | B |
| GSE39582_GSM972344 | B |
| GSE39582_GSM972345 | C |
| GSE39582_GSM972346 | A |
| GSE39582_GSM972347 | B |
| GSE39582_GSM972348 | B |
| GSE39582_GSM972349 | C |
| GSE39582_GSM972350 | B |
| GSE39582_GSM972351 | B |
| GSE39582_GSM972352 | C |
| GSE39582_GSM972353 | A |
| GSE39582_GSM972354 | B |
| GSE39582_GSM972355 | C |
| GSE39582_GSM972356 | B |
| GSE39582_GSM972357 | C |
| GSE39582_GSM972358 | B |
| GSE39582_GSM972359 | C |
| GSE39582_GSM972360 | A |
| GSE39582_GSM972361 | A |
| GSE39582_GSM972362 | B |
| GSE39582_GSM972363 | B |
| GSE39582_GSM972364 | A |
| GSE39582_GSM972365 | A |
| GSE39582_GSM972366 | C |
| GSE39582_GSM972367 | C |
| GSE39582_GSM972368 | B |
| GSE39582_GSM972369 | C |
| GSE39582_GSM972370 | B |
| GSE39582_GSM972371 | B |
| GSE39582_GSM972372 | B |
| GSE39582_GSM972373 | B |
| GSE39582_GSM972374 | B |
| GSE39582_GSM972375 | C |
| GSE39582_GSM972376 | C |
| GSE39582_GSM972377 | C |
| GSE39582_GSM972378 | C |
| GSE39582_GSM972379 | B |
| GSE39582_GSM972380 | C |
| GSE39582_GSM972381 | C |
| GSE39582_GSM972382 | B |
| GSE39582_GSM972383 | B |
| GSE39582_GSM972384 | B |
| GSE39582_GSM972385 | C |
| GSE39582_GSM972386 | B |
| GSE39582_GSM972387 | B |
| GSE39582_GSM972388 | B |
| GSE39582_GSM972389 | B |
| GSE39582_GSM972390 | A |
| GSE39582_GSM972391 | A |
| GSE39582_GSM972392 | B |
| GSE39582_GSM972393 | C |
| GSE39582_GSM972394 | C |
| GSE39582_GSM972395 | B |
| GSE39582_GSM972396 | B |
| GSE39582_GSM972397 | B |
| GSE39582_GSM972398 | B |
| GSE39582_GSM972399 | B |
| GSE39582_GSM972400 | B |
| GSE39582_GSM972401 | C |
| GSE39582_GSM972402 | C |
| GSE39582_GSM972403 | A |
| GSE39582_GSM972404 | B |
| GSE39582_GSM972405 | B |
| GSE39582_GSM972406 | A |
| GSE39582_GSM972407 | B |
| GSE39582_GSM972408 | B |
| GSE39582_GSM972409 | C |
| GSE39582_GSM972410 | C |
| GSE39582_GSM972411 | B |
| GSE39582_GSM972412 | C |
| GSE39582_GSM972413 | C |
| GSE39582_GSM972414 | B |
| GSE39582_GSM972415 | B |
| GSE39582_GSM972416 | C |
| GSE39582_GSM972417 | C |
| GSE39582_GSM972418 | B |
| GSE39582_GSM972419 | C |
| GSE39582_GSM972420 | C |
| GSE39582_GSM972421 | C |
| GSE39582_GSM972422 | C |
| GSE39582_GSM972423 | C |
| GSE39582_GSM972424 | B |
| GSE39582_GSM972425 | C |
| GSE39582_GSM972426 | C |
| GSE39582_GSM972427 | C |
| GSE39582_GSM972428 | C |
| GSE39582_GSM972429 | B |
| GSE39582_GSM972430 | A |
| GSE39582_GSM972431 | B |
| GSE39582_GSM972432 | C |
| GSE39582_GSM972433 | C |
| GSE39582_GSM972434 | B |
| GSE39582_GSM972435 | B |
| GSE39582_GSM972436 | B |
| GSE39582_GSM972437 | C |
| GSE39582_GSM972438 | C |
| GSE39582_GSM972439 | B |
| GSE39582_GSM972440 | A |
| GSE39582_GSM972441 | C |
| GSE39582_GSM972442 | B |
| GSE39582_GSM972443 | B |
| GSE39582_GSM972444 | C |
| GSE39582_GSM972445 | A |
| GSE39582_GSM972446 | A |
| GSE39582_GSM972447 | A |
| GSE39582_GSM972448 | A |
| GSE39582_GSM972449 | C |
| GSE39582_GSM972450 | B |
| GSE39582_GSM972451 | B |
| GSE39582_GSM972452 | A |
| GSE39582_GSM972453 | B |
| GSE39582_GSM972454 | B |
| GSE39582_GSM972455 | C |
| GSE39582_GSM972456 | B |
| GSE39582_GSM972457 | B |
| GSE39582_GSM972458 | A |
| GSE39582_GSM972459 | B |
| GSE39582_GSM972460 | B |
| GSE39582_GSM972461 | B |
| GSE39582_GSM972462 | B |
| GSE39582_GSM972463 | B |
| GSE39582_GSM972464 | C |
| GSE39582_GSM972465 | A |
| GSE39582_GSM972466 | B |
| GSE39582_GSM972467 | C |
| GSE39582_GSM972468 | B |
| GSE39582_GSM972469 | B |
| GSE39582_GSM972470 | C |
| GSE39582_GSM972471 | A |
| GSE39582_GSM972472 | A |
| GSE39582_GSM972473 | B |
| GSE39582_GSM972474 | B |
| GSE39582_GSM972475 | B |
| GSE39582_GSM972476 | C |
| GSE39582_GSM972477 | B |
| GSE39582_GSM972478 | B |
| GSE39582_GSM972479 | C |
| GSE39582_GSM972480 | B |
| GSE39582_GSM972481 | C |
| GSE39582_GSM972482 | C |
| GSE39582_GSM972483 | B |
| GSE39582_GSM972484 | B |
| GSE39582_GSM972485 | B |
| GSE39582_GSM972486 | C |
| GSE39582_GSM972487 | B |
| GSE39582_GSM972488 | B |
| GSE39582_GSM972489 | B |
| GSE39582_GSM972490 | B |
| GSE39582_GSM972491 | B |
| GSE39582_GSM972492 | B |
| GSE39582_GSM972493 | B |
| GSE39582_GSM972494 | B |
| GSE39582_GSM972495 | B |
| GSE39582_GSM972496 | B |
| GSE39582_GSM972497 | B |
| GSE39582_GSM972498 | B |
| GSE39582_GSM972499 | A |
| GSE39582_GSM972500 | C |
| GSE39582_GSM972501 | C |
| GSE39582_GSM972502 | B |
| GSE39582_GSM972503 | C |
| GSE39582_GSM972504 | C |
| GSE39582_GSM972505 | C |
| GSE39582_GSM972506 | C |
| GSE39582_GSM972507 | A |
| GSE39582_GSM972508 | B |
| GSE39582_GSM972509 | B |
| GSE39582_GSM972510 | B |
| GSE39582_GSM972511 | B |
| GSE39582_GSM972512 | A |
| GSE39582_GSM972513 | B |
| GSE39582_GSM972514 | B |
| GSE39582_GSM972515 | B |
| GSE39582_GSM972516 | B |
| GSE39582_GSM972517 | B |
| GSE39582_GSM972518 | C |
| GSE39582_GSM972519 | C |
| GSE39582_GSM972520 | B |
| GSE39582_GSM972521 | C |
| GSE39582_GSM972522 | C |

**Supplementary Table S10** | The CuproptosisScore of TCGA and GEO samples.

| id | CuproptosisScore |
| --- | --- |
| TCGA_TCGA-DM-A288 | -9.23564 |
| TCGA_TCGA-QL-A97D | -7.76934 |
| TCGA_TCGA-CM-6164 | 3.401967 |
| TCGA_TCGA-G4-6299 | 3.917485 |
| TCGA_TCGA-F4-6463 | 6.069186 |
| TCGA_TCGA-AZ-4615 | -8.6136 |
| TCGA_TCGA-AA-3549 | 1.777761 |
| TCGA_TCGA-AY-4071 | 14.36357 |
| TCGA_TCGA-CM-4752 | 1.707344 |
| TCGA_TCGA-DM-A1D9 | -9.69891 |
| TCGA_TCGA-AA-3688 | -6.1525 |
| TCGA_TCGA-AA-3854 | -3.14009 |
| TCGA_TCGA-A6-3809 | -9.00047 |
| TCGA_TCGA-CM-6165 | 4.178629 |
| TCGA_TCGA-CM-4751 | 3.567657 |
| TCGA_TCGA-A6-5659 | 5.395422 |
| TCGA_TCGA-AA-3494 | -3.74339 |
| TCGA_TCGA-CM-4750 | -7.62649 |
| TCGA_TCGA-AZ-4682 | -11.828 |
| TCGA_TCGA-G4-6625 | 7.588002 |
| TCGA_TCGA-DM-A0XF | -9.6152 |
| TCGA_TCGA-AA-3529 | -3.8392 |
| TCGA_TCGA-AA-3949 | -7.12276 |
| TCGA_TCGA-AA-3848 | 7.165494 |
| TCGA_TCGA-CA-6715 | -9.47281 |
| TCGA_TCGA-AA-3818 | -4.46923 |
| TCGA_TCGA-AA-3710 | -3.28848 |
| TCGA_TCGA-AA-3950 | -10.8691 |
| TCGA_TCGA-AA-A00N | 4.787973 |
| TCGA_TCGA-A6-6138 | 2.731349 |
| TCGA_TCGA-DM-A28E | -14.3837 |
| TCGA_TCGA-D5-6538 | -7.41292 |
| TCGA_TCGA-AA-A00K | -2.20679 |
| TCGA_TCGA-AA-3715 | 14.06705 |
| TCGA_TCGA-AA-3506 | 3.994994 |
| TCGA_TCGA-AU-3779 | 5.601225 |
| TCGA_TCGA-CK-5916 | -7.80666 |
| TCGA_TCGA-QG-A5Z1 | 9.153562 |
| TCGA_TCGA-AD-6890 | -4.83026 |
| TCGA_TCGA-A6-2685 | 20.85071 |
| TCGA_TCGA-AA-A00W | -8.4277 |
| TCGA_TCGA-CK-5914 | -11.29 |
| TCGA_TCGA-A6-2686 | 4.731589 |
| TCGA_TCGA-CA-5255 | -9.3002 |
| TCGA_TCGA-A6-A5ZU | 15.37603 |
| TCGA_TCGA-NH-A50V | 12.30684 |
| TCGA_TCGA-QG-A5YX | -15.0257 |
| TCGA_TCGA-A6-2677 | 1.315955 |
| TCGA_TCGA-CA-6717 | 4.730778 |
| TCGA_TCGA-A6-6649 | 11.96546 |
| TCGA_TCGA-NH-A6GC | 18.12452 |
| TCGA_TCGA-AA-3562 | 0.716078 |
| TCGA_TCGA-A6-4105 | -0.74341 |
| TCGA_TCGA-A6-6141 | 4.606387 |
| TCGA_TCGA-AA-A00R | -9.81998 |
| TCGA_TCGA-AZ-4684 | 2.020435 |
| TCGA_TCGA-CK-6747 | -5.61219 |
| TCGA_TCGA-AU-6004 | 0.043622 |
| TCGA_TCGA-A6-2672 | 10.70359 |
| TCGA_TCGA-QG-A5Z2 | -1.69188 |
| TCGA_TCGA-A6-6780 | -6.65924 |
| TCGA_TCGA-F4-6459 | 13.57205 |
| TCGA_TCGA-AA-3970 | -3.34336 |
| TCGA_TCGA-NH-A8F7 | -7.72232 |
| TCGA_TCGA-G4-6321 | 13.48307 |
| TCGA_TCGA-AY-6196 | 34.88761 |
| TCGA_TCGA-AA-3524 | -19.5675 |
| TCGA_TCGA-DM-A1D0 | -14.7002 |
| TCGA_TCGA-G4-6307 | -6.54464 |
| TCGA_TCGA-DM-A1HB | -17.7976 |
| TCGA_TCGA-AA-3697 | -4.92211 |
| TCGA_TCGA-CM-6679 | 14.538 |
| TCGA_TCGA-AY-6197 | -7.82548 |
| TCGA_TCGA-AA-3672 | 7.386981 |
| TCGA_TCGA-AA-3684 | 21.95205 |
| TCGA_TCGA-A6-2684 | 16.18075 |
| TCGA_TCGA-AZ-4323 | 18.5831 |
| TCGA_TCGA-AA-3971 | 4.524644 |
| TCGA_TCGA-AA-3989 | 5.194345 |
| TCGA_TCGA-AA-A00D | 2.532838 |
| TCGA_TCGA-AD-6548 | -5.56344 |
| TCGA_TCGA-D5-6539 | 0.80516 |
| TCGA_TCGA-G4-6302 | 22.68137 |
| TCGA_TCGA-A6-2679 | 14.53126 |
| TCGA_TCGA-A6-2674 | 24.95289 |
| TCGA_TCGA-AZ-4614 | -10.8495 |
| TCGA_TCGA-NH-A5IV | 1.445618 |
| TCGA_TCGA-A6-5664 | 8.324939 |
| TCGA_TCGA-AZ-4315 | -14.1299 |
| TCGA_TCGA-F4-6806 | 7.935895 |
| TCGA_TCGA-AA-3510 | -2.84522 |
| TCGA_TCGA-AY-A71X | -1.62152 |
| TCGA_TCGA-AA-3488 | -4.17399 |
| TCGA_TCGA-DM-A28F | -6.06475 |
| TCGA_TCGA-G4-6293 | 5.358917 |
| TCGA_TCGA-5M-AAT4 | -5.8181 |
| TCGA_TCGA-D5-6533 | -3.80271 |
| TCGA_TCGA-A6-5657 | 4.114224 |
| TCGA_TCGA-AA-3941 | -9.86015 |
| TCGA_TCGA-DM-A28K | -9.93254 |
| TCGA_TCGA-CM-5344 | 3.844499 |
| TCGA_TCGA-AA-3517 | 8.599653 |
| TCGA_TCGA-G4-6309 | -13.1334 |
| TCGA_TCGA-AY-6386 | -8.70626 |
| TCGA_TCGA-AA-3678 | 0.543965 |
| TCGA_TCGA-AA-A01X | 15.13541 |
| TCGA_TCGA-AA-3821 | -1.50287 |
| TCGA_TCGA-CM-6677 | -3.75209 |
| TCGA_TCGA-CM-5861 | -5.04394 |
| TCGA_TCGA-D5-6923 | 3.225411 |
| TCGA_TCGA-F4-6703 | 17.01795 |
| TCGA_TCGA-AZ-6605 | 10.76882 |
| TCGA_TCGA-NH-A6GB | -11.0158 |
| TCGA_TCGA-CM-5349 | 1.582154 |
| TCGA_TCGA-NH-A50U | 0.127602 |
| TCGA_TCGA-AA-3530 | -0.81776 |
| TCGA_TCGA-G4-6627 | 11.76601 |
| TCGA_TCGA-DM-A28G | -2.23916 |
| TCGA_TCGA-D5-6537 | -17.0023 |
| TCGA_TCGA-D5-5539 | 5.019566 |
| TCGA_TCGA-CM-6163 | 5.854214 |
| TCGA_TCGA-CM-4744 | -22.9271 |
| TCGA_TCGA-AA-A004 | 24.04172 |
| TCGA_TCGA-A6-3808 | 4.796369 |
| TCGA_TCGA-AA-A010 | -7.21857 |
| TCGA_TCGA-AA-A02J | -13.6965 |
| TCGA_TCGA-AA-3837 | 7.36377 |
| TCGA_TCGA-CK-4947 | -3.73499 |
| TCGA_TCGA-CA-5256 | -16.8297 |
| TCGA_TCGA-CM-6167 | 23.40874 |
| TCGA_TCGA-4N-A93T | 7.706428 |
| TCGA_TCGA-AA-3521 | 6.219849 |
| TCGA_TCGA-CA-5797 | -2.71323 |
| TCGA_TCGA-CM-6166 | -9.32159 |
| TCGA_TCGA-CK-4951 | -0.66911 |
| TCGA_TCGA-CA-6719 | 7.651065 |
| TCGA_TCGA-AA-A02R | -6.50295 |
| TCGA_TCGA-AA-A02O | 4.115347 |
| TCGA_TCGA-A6-5666 | -19.0034 |
| TCGA_TCGA-AY-5543 | -14.0889 |
| TCGA_TCGA-AA-3845 | -0.82481 |
| TCGA_TCGA-F4-6704 | 13.81212 |
| TCGA_TCGA-AA-3856 | 2.714328 |
| TCGA_TCGA-AA-A00Q | 4.853712 |
| TCGA_TCGA-A6-6781 | 26.24203 |
| TCGA_TCGA-AA-A00F | 8.691479 |
| TCGA_TCGA-D5-6920 | -0.32755 |
| TCGA_TCGA-AA-3815 | -4.32258 |
| TCGA_TCGA-D5-5538 | 0.724315 |
| TCGA_TCGA-AA-3867 | 9.11528 |
| TCGA_TCGA-A6-6782 | 10.9193 |
| TCGA_TCGA-A6-6654 | 15.9554 |
| TCGA_TCGA-AA-A01D | 8.132249 |
| TCGA_TCGA-G4-6311 | 3.073966 |
| TCGA_TCGA-A6-3807 | 1.833302 |
| TCGA_TCGA-AZ-5403 | -4.06756 |
| TCGA_TCGA-AD-6888 | -12.4248 |
| TCGA_TCGA-G4-6628 | -5.72623 |
| TCGA_TCGA-DM-A1D6 | -10.4928 |
| TCGA_TCGA-CM-5341 | -5.93716 |
| TCGA_TCGA-AA-3862 | -6.34058 |
| TCGA_TCGA-AA-3939 | -9.28927 |
| TCGA_TCGA-AA-3514 | 13.6421 |
| TCGA_TCGA-AA-3660 | -2.78416 |
| TCGA_TCGA-AA-3833 | 5.71308 |
| TCGA_TCGA-AA-A01P | 5.467124 |
| TCGA_TCGA-AA-A00Z | -5.07331 |
| TCGA_TCGA-CM-5863 | 12.37552 |
| TCGA_TCGA-AZ-6598 | -8.19941 |
| TCGA_TCGA-AA-3679 | -0.41902 |
| TCGA_TCGA-G4-6304 | -10.8179 |
| TCGA_TCGA-DM-A285 | 0.20908 |
| TCGA_TCGA-G4-6294 | -3.59708 |
| TCGA_TCGA-A6-4107 | 3.905744 |
| TCGA_TCGA-DM-A1D4 | -11.6123 |
| TCGA_TCGA-CA-6716 | 8.106204 |
| TCGA_TCGA-CK-4952 | -17.0669 |
| TCGA_TCGA-AA-A01V | 2.078736 |
| TCGA_TCGA-AA-3844 | -10.4911 |
| TCGA_TCGA-D5-6541 | 6.496146 |
| TCGA_TCGA-AA-A01G | -11.9886 |
| TCGA_TCGA-AA-3870 | 11.24636 |
| TCGA_TCGA-D5-6532 | -13.7396 |
| TCGA_TCGA-CM-6169 | 13.44782 |
| TCGA_TCGA-D5-5541 | -0.1899 |
| TCGA_TCGA-DM-A1DB | -9.31883 |
| TCGA_TCGA-AA-A00A | 0.466442 |
| TCGA_TCGA-D5-6530 | -0.18696 |
| TCGA_TCGA-AA-3855 | 1.230877 |
| TCGA_TCGA-CM-5868 | -3.45862 |
| TCGA_TCGA-AA-3519 | -3.61702 |
| TCGA_TCGA-AA-3526 | -10.8998 |
| TCGA_TCGA-DM-A28M | -17.6081 |
| TCGA_TCGA-D5-6926 | 10.24932 |
| TCGA_TCGA-AA-3534 | -1.50274 |
| TCGA_TCGA-G4-6317 | -6.26805 |
| TCGA_TCGA-AA-A03J | 10.15395 |
| TCGA_TCGA-CM-6680 | 11.91137 |
| TCGA_TCGA-AA-A01K | 5.942292 |
| TCGA_TCGA-AA-3984 | -12.1074 |
| TCGA_TCGA-D5-6536 | 9.530685 |
| TCGA_TCGA-AD-A5EK | -2.99779 |
| TCGA_TCGA-AA-3663 | -15.6664 |
| TCGA_TCGA-AZ-6607 | 23.56473 |
| TCGA_TCGA-AA-3520 | 1.178167 |
| TCGA_TCGA-AA-3543 | 0.329243 |
| TCGA_TCGA-AZ-5407 | -10.6376 |
| TCGA_TCGA-G4-6314 | 8.180182 |
| TCGA_TCGA-CM-5348 | 21.12883 |
| TCGA_TCGA-CK-6751 | -7.43093 |
| TCGA_TCGA-D5-6929 | 4.141161 |
| TCGA_TCGA-AA-3675 | -6.64305 |
| TCGA_TCGA-A6-2681 | 9.334646 |
| TCGA_TCGA-AA-3869 | -3.35401 |
| TCGA_TCGA-DM-A282 | -1.26867 |
| TCGA_TCGA-D5-7000 | -1.91734 |
| TCGA_TCGA-AM-5821 | -14.3377 |
| TCGA_TCGA-G4-6298 | 2.103043 |
| TCGA_TCGA-AA-A01S | -14.1263 |
| TCGA_TCGA-NH-A8F8 | 8.10135 |
| TCGA_TCGA-AA-3966 | 5.478534 |
| TCGA_TCGA-AA-3516 | -8.74103 |
| TCGA_TCGA-A6-2676 | -12.8366 |
| TCGA_TCGA-AA-3846 | -4.32647 |
| TCGA_TCGA-AA-3544 | 0.348162 |
| TCGA_TCGA-AA-3956 | 2.428904 |
| TCGA_TCGA-AA-3532 | -1.95278 |
| TCGA_TCGA-AA-3955 | -17.0413 |
| TCGA_TCGA-CK-5912 | -5.90219 |
| TCGA_TCGA-AD-6895 | -1.40925 |
| TCGA_TCGA-AA-3556 | -6.74971 |
| TCGA_TCGA-CM-6678 | -0.37502 |
| TCGA_TCGA-AA-3851 | -0.42639 |
| TCGA_TCGA-CM-5860 | -1.80745 |
| TCGA_TCGA-A6-5661 | 0.430202 |
| TCGA_TCGA-AA-A02F | -8.13496 |
| TCGA_TCGA-AA-3861 | -8.1731 |
| TCGA_TCGA-DM-A1DA | -17.9392 |
| TCGA_TCGA-CM-6162 | 10.56803 |
| TCGA_TCGA-AA-3522 | -4.30933 |
| TCGA_TCGA-AA-A00U | -9.81569 |
| TCGA_TCGA-F4-6807 | 10.85518 |
| TCGA_TCGA-AA-3930 | -9.95699 |
| TCGA_TCGA-AZ-6599 | -2.16209 |
| TCGA_TCGA-AA-3947 | -26.5189 |
| TCGA_TCGA-AA-3841 | 13.98398 |
| TCGA_TCGA-CM-6171 | -6.39756 |
| TCGA_TCGA-CM-4743 | -10.7016 |
| TCGA_TCGA-AA-3814 | 4.502937 |
| TCGA_TCGA-NH-A6GA | 3.438567 |
| TCGA_TCGA-A6-2675 | 15.3573 |
| TCGA_TCGA-G4-6297 | 10.40346 |
| TCGA_TCGA-A6-5656 | 14.90716 |
| TCGA_TCGA-DM-A0X9 | -11.317 |
| TCGA_TCGA-AA-3553 | -4.61291 |
| TCGA_TCGA-DM-A0XD | -12.7529 |
| TCGA_TCGA-AA-3511 | 3.349501 |
| TCGA_TCGA-A6-A56B | 4.476934 |
| TCGA_TCGA-A6-5667 | -1.13754 |
| TCGA_TCGA-AA-3866 | -0.70801 |
| TCGA_TCGA-AA-A017 | 9.600208 |
| TCGA_TCGA-CM-6674 | -4.884 |
| TCGA_TCGA-DM-A1D8 | -3.5508 |
| TCGA_TCGA-CM-6170 | 5.451073 |
| TCGA_TCGA-AZ-4616 | -8.32133 |
| TCGA_TCGA-AA-3994 | 1.895476 |
| TCGA_TCGA-AA-3877 | -2.90266 |
| TCGA_TCGA-WS-AB45 | 19.12021 |
| TCGA_TCGA-AA-A00L | -4.85082 |
| TCGA_TCGA-CM-5864 | -6.60579 |
| TCGA_TCGA-CA-5254 | -2.35973 |
| TCGA_TCGA-D5-6898 | 2.161189 |
| TCGA_TCGA-AA-3696 | 5.04308 |
| TCGA_TCGA-DM-A280 | -9.97751 |
| TCGA_TCGA-AA-A00O | 18.08522 |
| TCGA_TCGA-QG-A5YW | 0.666259 |
| TCGA_TCGA-AD-6965 | -6.28625 |
| TCGA_TCGA-CK-4948 | -2.34537 |
| TCGA_TCGA-DM-A1HA | -10.1844 |
| TCGA_TCGA-5M-AAT5 | -13.18 |
| TCGA_TCGA-F4-6809 | 14.75926 |
| TCGA_TCGA-CM-4748 | 14.24036 |
| TCGA_TCGA-AA-3858 | -2.10936 |
| TCGA_TCGA-D5-6531 | -7.28886 |
| TCGA_TCGA-AA-3662 | 4.191632 |
| TCGA_TCGA-AM-5820 | -6.99608 |
| TCGA_TCGA-AA-A02K | -3.99392 |
| TCGA_TCGA-G4-6310 | 8.604063 |
| TCGA_TCGA-AD-6889 | -11.3099 |
| TCGA_TCGA-AA-3872 | 13.20122 |
| TCGA_TCGA-AA-A01R | -1.34774 |
| TCGA_TCGA-AA-3975 | 10.00445 |
| TCGA_TCGA-5M-AAT6 | 0.911667 |
| TCGA_TCGA-D5-6931 | -2.24579 |
| TCGA_TCGA-D5-6924 | 8.638529 |
| TCGA_TCGA-AA-3860 | 3.375038 |
| TCGA_TCGA-A6-5665 | 0.315787 |
| TCGA_TCGA-5M-AATA | -1.51007 |
| TCGA_TCGA-D5-6540 | -4.41507 |
| TCGA_TCGA-CA-6718 | -5.66804 |
| TCGA_TCGA-AA-3986 | 6.380197 |
| TCGA_TCGA-G4-6323 | 3.878934 |
| TCGA_TCGA-A6-A566 | 13.31712 |
| TCGA_TCGA-AZ-6608 | -20.0471 |
| TCGA_TCGA-AA-3712 | -2.14847 |
| TCGA_TCGA-A6-5662 | 6.744505 |
| TCGA_TCGA-AA-3831 | -3.06696 |
| TCGA_TCGA-AA-A02H | 4.930247 |
| TCGA_TCGA-CM-6676 | 1.315129 |
| TCGA_TCGA-AD-6899 | 11.93255 |
| TCGA_TCGA-AA-3685 | -7.61399 |
| TCGA_TCGA-CK-5915 | -13.5499 |
| TCGA_TCGA-AA-3548 | 1.934961 |
| TCGA_TCGA-F4-6569 | 12.57817 |
| TCGA_TCGA-F4-6461 | 11.15854 |
| TCGA_TCGA-F4-6854 | 1.759816 |
| TCGA_TCGA-F4-6856 | -4.81244 |
| TCGA_TCGA-AY-A54L | -14.933 |
| TCGA_TCGA-AA-3538 | 1.21394 |
| TCGA_TCGA-AA-3842 | -9.39767 |
| TCGA_TCGA-AZ-4308 | 8.822764 |
| TCGA_TCGA-CK-6746 | -10.1113 |
| TCGA_TCGA-AA-3509 | -6.61467 |
| TCGA_TCGA-AA-3680 | 8.422427 |
| TCGA_TCGA-AA-3542 | -5.22421 |
| TCGA_TCGA-AD-6964 | 10.59433 |
| TCGA_TCGA-A6-6650 | -2.21875 |
| TCGA_TCGA-A6-6140 | -7.21101 |
| TCGA_TCGA-AA-A024 | 7.443463 |
| TCGA_TCGA-A6-2682 | 4.861983 |
| TCGA_TCGA-CM-6675 | -3.85492 |
| TCGA_TCGA-DM-A1D7 | -8.97029 |
| TCGA_TCGA-4T-AA8H | -8.73984 |
| TCGA_TCGA-A6-6653 | -9.16056 |
| TCGA_TCGA-AA-3666 | -10.4457 |
| TCGA_TCGA-F4-6460 | 13.85463 |
| TCGA_TCGA-AA-3655 | -3.6302 |
| TCGA_TCGA-AA-3673 | 0.185494 |
| TCGA_TCGA-AA-3952 | 0.171027 |
| TCGA_TCGA-T9-A92H | -11.6998 |
| TCGA_TCGA-AY-4070 | 10.30193 |
| TCGA_TCGA-AY-A8YK | -1.72822 |
| TCGA_TCGA-CK-4950 | -2.09758 |
| TCGA_TCGA-F4-6570 | 6.290013 |
| TCGA_TCGA-AZ-6600 | 6.050849 |
| TCGA_TCGA-AA-A00J | -5.36217 |
| TCGA_TCGA-AA-3667 | 7.849931 |
| TCGA_TCGA-AA-3554 | 4.707977 |
| TCGA_TCGA-AZ-6606 | -11.3397 |
| TCGA_TCGA-AA-3852 | 2.378074 |
| TCGA_TCGA-D5-6922 | 7.559377 |
| TCGA_TCGA-A6-6137 | 5.828286 |
| TCGA_TCGA-AA-3495 | -7.05799 |
| TCGA_TCGA-AA-3489 | 17.10106 |
| TCGA_TCGA-G4-6322 | -2.20282 |
| TCGA_TCGA-SS-A7HO | -6.88709 |
| TCGA_TCGA-AA-3976 | -10.2092 |
| TCGA_TCGA-AA-3811 | -0.11841 |
| TCGA_TCGA-AD-A5EJ | -11.4231 |
| TCGA_TCGA-AA-3819 | -3.74646 |
| TCGA_TCGA-AA-A02Y | -17.6572 |
| TCGA_TCGA-AZ-6603 | 6.164052 |
| TCGA_TCGA-AA-A022 | 14.22646 |
| TCGA_TCGA-G4-6626 | -11.9123 |
| TCGA_TCGA-AA-3875 | -4.83139 |
| TCGA_TCGA-G4-6306 | -13.5048 |
| TCGA_TCGA-G4-6320 | -3.40145 |
| TCGA_TCGA-CK-6748 | 14.62165 |
| TCGA_TCGA-AA-3864 | -15.1665 |
| TCGA_TCGA-A6-6142 | 14.13593 |
| TCGA_TCGA-A6-2678 | -8.22331 |
| TCGA_TCGA-CA-5796 | 0.007427 |
| TCGA_TCGA-A6-A567 | 2.173348 |
| TCGA_TCGA-AA-3713 | -1.24552 |
| TCGA_TCGA-A6-6648 | -10.8386 |
| TCGA_TCGA-A6-A565 | 16.80116 |
| TCGA_TCGA-A6-5660 | -0.32874 |
| TCGA_TCGA-AZ-6601 | -2.66407 |
| TCGA_TCGA-5M-AATE | -4.47545 |
| TCGA_TCGA-CM-6161 | -2.52315 |
| TCGA_TCGA-AA-A01Q | -6.19562 |
| TCGA_TCGA-AZ-4313 | -11.1003 |
| TCGA_TCGA-A6-6652 | 0.503367 |
| TCGA_TCGA-AA-A00E | -3.84726 |
| TCGA_TCGA-F4-6808 | -9.12353 |
| TCGA_TCGA-AA-A02W | -2.19395 |
| TCGA_TCGA-DM-A28C | -3.41252 |
| TCGA_TCGA-D5-6927 | -3.24759 |
| TCGA_TCGA-AA-3980 | -10.0485 |
| TCGA_TCGA-AA-3681 | -4.90838 |
| TCGA_TCGA-AA-3492 | -10.0168 |
| TCGA_TCGA-A6-2671 | 6.432885 |
| TCGA_TCGA-D5-6535 | 1.238921 |
| TCGA_TCGA-AA-A029 | -12.4007 |
| TCGA_TCGA-AD-6963 | -9.70858 |
| TCGA_TCGA-CM-4747 | 15.24371 |
| TCGA_TCGA-NH-A50T | -19.0148 |
| TCGA_TCGA-CM-6168 | 6.172879 |
| TCGA_TCGA-AA-3518 | -4.44937 |
| TCGA_TCGA-AA-A01T | 2.431455 |
| TCGA_TCGA-RU-A8FL | -20.896 |
| TCGA_TCGA-A6-6651 | 20.20446 |
| TCGA_TCGA-AA-3850 | 5.524983 |
| TCGA_TCGA-AA-3972 | -7.9128 |
| TCGA_TCGA-AA-3812 | 24.29826 |
| TCGA_TCGA-AY-A69D | -7.1833 |
| TCGA_TCGA-AA-A01Z | -7.17165 |
| TCGA_TCGA-A6-3810 | 20.49789 |
| TCGA_TCGA-DM-A28H | -20.8921 |
| TCGA_TCGA-G4-6586 | -14.5162 |
| TCGA_TCGA-D5-6928 | 15.39789 |
| TCGA_TCGA-G4-6315 | -11.2808 |
| TCGA_TCGA-D5-6534 | 29.83738 |
| TCGA_TCGA-AA-3664 | -4.39396 |
| TCGA_TCGA-G4-6303 | 6.386365 |
| TCGA_TCGA-AA-3692 | 0.542637 |
| TCGA_TCGA-AA-3968 | 1.268158 |
| TCGA_TCGA-F4-6805 | 4.329407 |
| TCGA_TCGA-AA-3560 | -7.32929 |
| TCGA_TCGA-D5-5537 | 4.46857 |
| TCGA_TCGA-CM-5862 | -4.37518 |
| TCGA_TCGA-D5-6932 | 4.834721 |
| TCGA_TCGA-AA-3982 | -2.1752 |
| TCGA_TCGA-DM-A28A | -1.18906 |
| TCGA_TCGA-CM-6172 | 2.41051 |
| TCGA_TCGA-D5-6930 | -2.10998 |
| TCGA_TCGA-D5-6529 | 9.123211 |
| TCGA_TCGA-A6-2683 | 13.09264 |
| TCGA_TCGA-CK-5913 | -8.22359 |
| TCGA_TCGA-AA-3977 | -14.4713 |
| TCGA_TCGA-AA-3496 | 6.875988 |
| TCGA_TCGA-D5-5540 | -25.8158 |
| TCGA_TCGA-AD-6901 | 24.84545 |
| TCGA_TCGA-AA-A01F | -6.15685 |
| TCGA_TCGA-AA-3552 | 9.195261 |
| TCGA_TCGA-F4-6855 | 14.66837 |
| TCGA_TCGA-AD-5900 | -5.76781 |
| TCGA_TCGA-AA-3979 | -22.9622 |
| TCGA_TCGA-AA-A01I | -14.8471 |
| TCGA_TCGA-AA-A03F | 14.22617 |
| TCGA_TCGA-3L-AA1B | 10.92112 |
| TCGA_TCGA-AA-3693 | -6.52131 |
| TCGA_TCGA-AA-3502 | -2.41251 |
| TCGA_TCGA-AA-A02E | 2.352872 |
| TCGA_TCGA-CM-4746 | -13.0233 |
| TCGA_TCGA-G4-6295 | -11.5753 |
| TCGA_TCGA-AA-A01C | 2.270089 |
| TCGA_TCGA-G4-6588 | -12.6322 |
| TCGA_TCGA-A6-2680 | 2.681038 |
| TCGA_TCGA-QG-A5YV | -9.54102 |
| TCGA_TCGA-AA-3561 | -19.9635 |
| TCGA_TCGA-AA-3525 | -4.3837 |
| TCGA_TCGA-AA-3531 | 0.899486 |
| TCGA_TCGA-AA-3973 | 13.88029 |
| TCGA_TCGA-AA-3527 | 12.6755 |
| TCGA_TCGA-AA-3555 | -5.47359 |
| TCGA_TCGA-AG-A025 | -0.54962 |
| TCGA_TCGA-EI-6884 | 5.157116 |
| TCGA_TCGA-AG-3582 | -0.53097 |
| TCGA_TCGA-DC-6154 | 7.783771 |
| TCGA_TCGA-AG-3611 | 4.272176 |
| TCGA_TCGA-DY-A1DD | 8.347929 |
| TCGA_TCGA-AG-3609 | 3.353628 |
| TCGA_TCGA-AG-A01L | -0.52438 |
| TCGA_TCGA-AG-4022 | 12.37334 |
| TCGA_TCGA-AG-A01W | -10.377 |
| TCGA_TCGA-AG-A02N | -6.78876 |
| TCGA_TCGA-AH-6549 | -5.65943 |
| TCGA_TCGA-AH-6903 | -7.16085 |
| TCGA_TCGA-AG-3727 | 15.90957 |
| TCGA_TCGA-F5-6863 | 4.82892 |
| TCGA_TCGA-AG-3887 | -5.35517 |
| TCGA_TCGA-AG-A01J | 1.533446 |
| TCGA_TCGA-AF-5654 | -12.7555 |
| TCGA_TCGA-DY-A1DE | -0.05107 |
| TCGA_TCGA-AF-3913 | -0.09091 |
| TCGA_TCGA-AG-A014 | -5.94539 |
| TCGA_TCGA-AG-4015 | 0.156292 |
| TCGA_TCGA-G5-6572 | 21.17824 |
| TCGA_TCGA-AG-A011 | 2.051936 |
| TCGA_TCGA-EI-6511 | 4.56321 |
| TCGA_TCGA-AG-4008 | 4.808083 |
| TCGA_TCGA-AG-3731 | 4.027798 |
| TCGA_TCGA-AG-A032 | 5.653768 |
| TCGA_TCGA-AH-6544 | -17.5087 |
| TCGA_TCGA-AG-A020 | -13.623 |
| TCGA_TCGA-AG-A026 | 9.586017 |
| TCGA_TCGA-DY-A1H8 | -10.8066 |
| TCGA_TCGA-AG-3883 | 11.03467 |
| TCGA_TCGA-EI-7004 | 17.95981 |
| TCGA_TCGA-AF-6136 | -1.43734 |
| TCGA_TCGA-DC-4745 | -12.3444 |
| TCGA_TCGA-EI-6883 | 1.017628 |
| TCGA_TCGA-EI-6917 | -3.37933 |
| TCGA_TCGA-F5-6861 | -4.99515 |
| TCGA_TCGA-EI-6508 | -5.82343 |
| TCGA_TCGA-F5-6465 | 12.83652 |
| TCGA_TCGA-AF-6672 | 6.824338 |
| TCGA_TCGA-AG-3885 | 9.972003 |
| TCGA_TCGA-EI-6509 | -0.39289 |
| TCGA_TCGA-DC-5337 | -17.0978 |
| TCGA_TCGA-AG-3893 | 10.78506 |
| TCGA_TCGA-AG-A02G | 4.487163 |
| TCGA_TCGA-AG-3901 | 14.56377 |
| TCGA_TCGA-AG-3726 | 17.94043 |
| TCGA_TCGA-AG-3896 | 1.259463 |
| TCGA_TCGA-DC-6160 | -8.48725 |
| TCGA_TCGA-AG-3581 | 5.409611 |
| TCGA_TCGA-G5-6641 | -2.73288 |
| TCGA_TCGA-F5-6812 | 14.37273 |
| TCGA_TCGA-AG-4001 | 11.83817 |
| TCGA_TCGA-AF-2687 | 9.876023 |
| TCGA_TCGA-AH-6644 | 5.488108 |
| TCGA_TCGA-AG-3586 | -8.7958 |
| TCGA_TCGA-AG-3909 | 8.40704 |
| TCGA_TCGA-AG-3878 | 23.81445 |
| TCGA_TCGA-AG-3592 | -7.17962 |
| TCGA_TCGA-F5-6814 | -9.31512 |
| TCGA_TCGA-AG-3605 | -9.66588 |
| TCGA_TCGA-EI-6513 | -4.95194 |
| TCGA_TCGA-DC-6158 | 7.904142 |
| TCGA_TCGA-EI-6512 | -7.70679 |
| TCGA_TCGA-AG-3601 | -4.11402 |
| TCGA_TCGA-CI-6622 | -7.27848 |
| TCGA_TCGA-BM-6198 | 5.376422 |
| TCGA_TCGA-AG-3881 | 18.98518 |
| TCGA_TCGA-DY-A1DC | -5.60975 |
| TCGA_TCGA-AG-3612 | 2.060104 |
| TCGA_TCGA-AG-3600 | -10.0402 |
| TCGA_TCGA-AG-3599 | -3.21086 |
| TCGA_TCGA-AG-3584 | 6.793451 |
| TCGA_TCGA-DC-6156 | 19.9064 |
| TCGA_TCGA-DT-5265 | 13.13956 |
| TCGA_TCGA-AG-3902 | 2.42479 |
| TCGA_TCGA-AG-A036 | -3.18483 |
| TCGA_TCGA-AG-3593 | -1.15068 |
| TCGA_TCGA-AG-A01Y | 0.373911 |
| TCGA_TCGA-AG-3580 | -7.93438 |
| TCGA_TCGA-AG-3742 | -4.11252 |
| TCGA_TCGA-AG-A002 | -5.39617 |
| TCGA_TCGA-DC-6683 | -2.20241 |
| TCGA_TCGA-AF-6655 | 1.562664 |
| TCGA_TCGA-AG-A023 | 9.79301 |
| TCGA_TCGA-AG-3728 | 28.0027 |
| TCGA_TCGA-AF-3400 | 6.366032 |
| TCGA_TCGA-AF-2691 | 6.101654 |
| TCGA_TCGA-CL-5918 | -9.77631 |
| TCGA_TCGA-F5-6864 | 13.95986 |
| TCGA_TCGA-AF-A56K | 14.72164 |
| TCGA_TCGA-AG-4021 | 0.041688 |
| TCGA_TCGA-AG-A01N | -0.32626 |
| TCGA_TCGA-DC-5869 | -10.4678 |
| TCGA_TCGA-AG-3608 | -3.05682 |
| TCGA_TCGA-DY-A0XA | 1.020583 |
| TCGA_TCGA-AG-3594 | -1.36806 |
| TCGA_TCGA-EF-5831 | -2.07131 |
| TCGA_TCGA-AG-3725 | -4.94034 |
| TCGA_TCGA-EI-6514 | 9.192278 |
| TCGA_TCGA-CI-6624 | 8.045372 |
| TCGA_TCGA-CI-6620 | -8.11255 |
| TCGA_TCGA-CI-6621 | 0.21173 |
| TCGA_TCGA-EI-6510 | -7.611 |
| TCGA_TCGA-DY-A1DG | -7.42987 |
| TCGA_TCGA-AG-A02X | -14.1416 |
| TCGA_TCGA-AG-3587 | -0.58029 |
| TCGA_TCGA-AG-3894 | -2.47666 |
| TCGA_TCGA-AG-3999 | 4.04569 |
| TCGA_TCGA-AG-A00H | 4.776049 |
| TCGA_TCGA-AF-A56N | 0.311218 |
| TCGA_TCGA-AG-A00C | -7.7456 |
| TCGA_TCGA-EI-6885 | 12.04319 |
| TCGA_TCGA-AG-3732 | 9.177806 |
| TCGA_TCGA-AG-4007 | 8.04206 |
| TCGA_TCGA-AG-A016 | -8.17767 |
| TCGA_TCGA-EI-6882 | -5.73696 |
| TCGA_TCGA-AG-3575 | -6.40304 |
| TCGA_TCGA-AF-2693 | -2.71753 |
| TCGA_TCGA-DC-6157 | 0.253028 |
| TCGA_TCGA-AF-4110 | 10.48461 |
| TCGA_TCGA-AG-3591 | -6.36664 |
| TCGA_TCGA-EI-6881 | -10.4226 |
| TCGA_TCGA-AG-A008 | -8.57492 |
| TCGA_TCGA-AG-A015 | -7.74855 |
| TCGA_TCGA-AG-3892 | -7.08742 |
| TCGA_TCGA-G5-6233 | -1.8625 |
| TCGA_TCGA-AG-3898 | -4.23147 |
| TCGA_TCGA-AG-3578 | 2.750198 |
| TCGA_TCGA-DC-6682 | -17.8283 |
| TCGA_TCGA-F5-6813 | 2.328005 |
| TCGA_TCGA-F5-6702 | 17.78228 |
| TCGA_TCGA-CL-5917 | -0.17143 |
| TCGA_TCGA-CL-4957 | -12.2002 |
| TCGA_TCGA-EI-6507 | 4.875315 |
| TCGA_TCGA-DC-4749 | -14.8641 |
| TCGA_TCGA-AF-2690 | 19.88016 |
| TCGA_TCGA-EF-5830 | -11.7728 |
| TCGA_TCGA-AF-3911 | -2.91382 |
| TCGA_TCGA-DC-6155 | -4.38947 |
| TCGA_TCGA-DC-6681 | 6.225192 |
| TCGA_TCGA-AF-A56L | -1.06984 |
| TCGA_TCGA-CI-6623 | -10.7193 |
| TCGA_TCGA-AH-6897 | -17.99 |
| TCGA_TCGA-AG-3574 | -0.02636 |
| TCGA_TCGA-G5-6235 | -10.8318 |
| TCGA_TCGA-AG-4005 | -0.34168 |
| TCGA_TCGA-AG-3583 | -9.37351 |
| TCGA_TCGA-AH-6547 | 19.53542 |
| TCGA_TCGA-EI-7002 | 10.84093 |
| TCGA_TCGA-AG-A00Y | -2.31019 |
| TCGA_TCGA-EI-6506 | 2.01306 |
| TCGA_TCGA-AH-6643 | -0.44778 |
| TCGA_TCGA-AG-3890 | 13.52004 |
| TCGA_TCGA-DY-A1DF | 10.4296 |
| TCGA_TCGA-F5-6571 | 14.79806 |
| TCGA_TCGA-AG-3882 | 24.94693 |
| TCGA_TCGA-AG-3598 | -12.3618 |
| TCGA_TCGA-F5-6811 | 8.561871 |
| TCGA_TCGA-F5-6810 | 2.525183 |
| TCGA_TCGA-F5-6464 | 18.5399 |
| TCGA_TCGA-CI-6619 | -1.07782 |
| TCGA_TCGA-AG-3602 | -12.0873 |
| TCGA_TCGA-AF-2692 | 1.103616 |
| GSE39582_GSM971957 | -6.40347 |
| GSE39582_GSM971958 | 2.955745 |
| GSE39582_GSM971959 | -14.2831 |
| GSE39582_GSM971960 | -6.49835 |
| GSE39582_GSM971961 | 21.05423 |
| GSE39582_GSM971962 | 13.37084 |
| GSE39582_GSM971963 | 7.390715 |
| GSE39582_GSM971964 | -9.62826 |
| GSE39582_GSM971965 | 6.307282 |
| GSE39582_GSM971966 | -5.64893 |
| GSE39582_GSM971967 | -7.52197 |
| GSE39582_GSM971968 | 11.96363 |
| GSE39582_GSM971969 | -8.26967 |
| GSE39582_GSM971970 | -14.2565 |
| GSE39582_GSM971971 | -10.3476 |
| GSE39582_GSM971972 | -7.40639 |
| GSE39582_GSM971973 | -14.1515 |
| GSE39582_GSM971974 | 8.530439 |
| GSE39582_GSM971975 | -10.2251 |
| GSE39582_GSM971976 | -10.3473 |
| GSE39582_GSM971977 | 4.628742 |
| GSE39582_GSM971978 | -14.271 |
| GSE39582_GSM971979 | -6.06726 |
| GSE39582_GSM971980 | 7.411996 |
| GSE39582_GSM971981 | -5.46633 |
| GSE39582_GSM971982 | 0.927968 |
| GSE39582_GSM971983 | -2.21274 |
| GSE39582_GSM971984 | -13.3947 |
| GSE39582_GSM971985 | -4.39031 |
| GSE39582_GSM971986 | 27.21896 |
| GSE39582_GSM971987 | 18.12613 |
| GSE39582_GSM971988 | 3.499406 |
| GSE39582_GSM971989 | -3.48191 |
| GSE39582_GSM971990 | -10.8289 |
| GSE39582_GSM971991 | 3.100112 |
| GSE39582_GSM971992 | -8.78863 |
| GSE39582_GSM971993 | -14.6676 |
| GSE39582_GSM971994 | -8.95064 |
| GSE39582_GSM971995 | 5.760489 |
| GSE39582_GSM971996 | 15.36155 |
| GSE39582_GSM971997 | 0.574251 |
| GSE39582_GSM971998 | 6.901576 |
| GSE39582_GSM971999 | 1.112852 |
| GSE39582_GSM972000 | 1.747852 |
| GSE39582_GSM972001 | -6.44735 |
| GSE39582_GSM972002 | -15.9113 |
| GSE39582_GSM972003 | -5.92954 |
| GSE39582_GSM972004 | -1.09793 |
| GSE39582_GSM972005 | 0.633667 |
| GSE39582_GSM972006 | 8.932755 |
| GSE39582_GSM972007 | 18.01782 |
| GSE39582_GSM972008 | 3.822636 |
| GSE39582_GSM972009 | 13.45577 |
| GSE39582_GSM972010 | -3.38264 |
| GSE39582_GSM972011 | 31.13829 |
| GSE39582_GSM972012 | -2.86274 |
| GSE39582_GSM972013 | -7.38424 |
| GSE39582_GSM972014 | -13.1434 |
| GSE39582_GSM972015 | -6.88466 |
| GSE39582_GSM972016 | -1.21852 |
| GSE39582_GSM972017 | 3.007846 |
| GSE39582_GSM972018 | 3.476553 |
| GSE39582_GSM972019 | 8.840472 |
| GSE39582_GSM972020 | -6.08898 |
| GSE39582_GSM972021 | -14.3772 |
| GSE39582_GSM972022 | -0.15013 |
| GSE39582_GSM972023 | 1.057376 |
| GSE39582_GSM972024 | -10.8969 |
| GSE39582_GSM972025 | 0.542815 |
| GSE39582_GSM972026 | 1.940023 |
| GSE39582_GSM972027 | -1.87672 |
| GSE39582_GSM972028 | 5.895345 |
| GSE39582_GSM972029 | 12.65306 |
| GSE39582_GSM972030 | 9.223662 |
| GSE39582_GSM972031 | 7.692942 |
| GSE39582_GSM972032 | 1.769571 |
| GSE39582_GSM972033 | -4.62933 |
| GSE39582_GSM972034 | -4.52183 |
| GSE39582_GSM972035 | -15.2555 |
| GSE39582_GSM972036 | -0.89967 |
| GSE39582_GSM972037 | -8.63239 |
| GSE39582_GSM972038 | -5.91117 |
| GSE39582_GSM972039 | 27.93036 |
| GSE39582_GSM972040 | -9.69491 |
| GSE39582_GSM972041 | -2.32552 |
| GSE39582_GSM972042 | -7.96565 |
| GSE39582_GSM972043 | -9.53857 |
| GSE39582_GSM972044 | -14.0297 |
| GSE39582_GSM972045 | -9.21379 |
| GSE39582_GSM972046 | 15.08108 |
| GSE39582_GSM972047 | -4.81838 |
| GSE39582_GSM972048 | -5.30809 |
| GSE39582_GSM972049 | -10.1772 |
| GSE39582_GSM972050 | 7.855812 |
| GSE39582_GSM972051 | 16.72622 |
| GSE39582_GSM972052 | -6.77697 |
| GSE39582_GSM972053 | -11.1466 |
| GSE39582_GSM972054 | 18.04269 |
| GSE39582_GSM972055 | -2.95461 |
| GSE39582_GSM972056 | -6.07986 |
| GSE39582_GSM972057 | -6.72906 |
| GSE39582_GSM972058 | -7.1379 |
| GSE39582_GSM972059 | 9.403875 |
| GSE39582_GSM972060 | -5.81392 |
| GSE39582_GSM972061 | 6.38635 |
| GSE39582_GSM972062 | -5.70085 |
| GSE39582_GSM972063 | -1.97609 |
| GSE39582_GSM972064 | 11.69106 |
| GSE39582_GSM972065 | -6.42803 |
| GSE39582_GSM972066 | -6.07113 |
| GSE39582_GSM972067 | 5.581079 |
| GSE39582_GSM972068 | 16.70613 |
| GSE39582_GSM972069 | -10.7667 |
| GSE39582_GSM972070 | 6.945008 |
| GSE39582_GSM972071 | -6.85136 |
| GSE39582_GSM972072 | -6.86073 |
| GSE39582_GSM972073 | -0.22825 |
| GSE39582_GSM972074 | -0.7667 |
| GSE39582_GSM972075 | -13.5877 |
| GSE39582_GSM972076 | -8.2552 |
| GSE39582_GSM972077 | 22.67062 |
| GSE39582_GSM972078 | -0.93982 |
| GSE39582_GSM972079 | 12.55943 |
| GSE39582_GSM972080 | -10.2109 |
| GSE39582_GSM972081 | -5.34257 |
| GSE39582_GSM972082 | 5.189561 |
| GSE39582_GSM972083 | -2.05582 |
| GSE39582_GSM972084 | 9.306444 |
| GSE39582_GSM972085 | -2.47436 |
| GSE39582_GSM972086 | -16.9337 |
| GSE39582_GSM972087 | -9.86747 |
| GSE39582_GSM972088 | 16.22381 |
| GSE39582_GSM972089 | 18.5749 |
| GSE39582_GSM972090 | -8.51093 |
| GSE39582_GSM972091 | -10.8652 |
| GSE39582_GSM972092 | 4.96413 |
| GSE39582_GSM972093 | 9.604878 |
| GSE39582_GSM972094 | -0.50196 |
| GSE39582_GSM972095 | -2.0533 |
| GSE39582_GSM972096 | -4.72933 |
| GSE39582_GSM972097 | 40.88216 |
| GSE39582_GSM972098 | 4.485264 |
| GSE39582_GSM972099 | 0.168788 |
| GSE39582_GSM972100 | -4.60699 |
| GSE39582_GSM972101 | 3.804259 |
| GSE39582_GSM972102 | 15.57569 |
| GSE39582_GSM972103 | -3.20496 |
| GSE39582_GSM972104 | 14.54695 |
| GSE39582_GSM972105 | 14.51805 |
| GSE39582_GSM972106 | -2.0012 |
| GSE39582_GSM972107 | -1.86859 |
| GSE39582_GSM972108 | -1.18905 |
| GSE39582_GSM972109 | -4.22172 |
| GSE39582_GSM972110 | -8.06771 |
| GSE39582_GSM972111 | 5.207232 |
| GSE39582_GSM972112 | 8.756671 |
| GSE39582_GSM972113 | 24.30241 |
| GSE39582_GSM972114 | -6.54043 |
| GSE39582_GSM972115 | 10.28221 |
| GSE39582_GSM972116 | 14.29667 |
| GSE39582_GSM972117 | 4.128885 |
| GSE39582_GSM972118 | -9.54313 |
| GSE39582_GSM972119 | -0.04982 |
| GSE39582_GSM972120 | -8.79024 |
| GSE39582_GSM972121 | 5.152027 |
| GSE39582_GSM972122 | -9.71294 |
| GSE39582_GSM972123 | -10.8124 |
| GSE39582_GSM972124 | 3.696489 |
| GSE39582_GSM972125 | 9.43352 |
| GSE39582_GSM972126 | -6.90751 |
| GSE39582_GSM972127 | 11.91012 |
| GSE39582_GSM972128 | -0.28573 |
| GSE39582_GSM972129 | -12.316 |
| GSE39582_GSM972130 | -1.174 |
| GSE39582_GSM972131 | 14.29753 |
| GSE39582_GSM972132 | -5.38965 |
| GSE39582_GSM972133 | 12.74937 |
| GSE39582_GSM972134 | 3.267582 |
| GSE39582_GSM972135 | -12.8007 |
| GSE39582_GSM972136 | -7.69306 |
| GSE39582_GSM972137 | -7.46435 |
| GSE39582_GSM972138 | -2.81919 |
| GSE39582_GSM972139 | -1.7044 |
| GSE39582_GSM972140 | 7.830145 |
| GSE39582_GSM972141 | 2.932029 |
| GSE39582_GSM972142 | -1.38948 |
| GSE39582_GSM972143 | 0.084605 |
| GSE39582_GSM972144 | 2.802469 |
| GSE39582_GSM972145 | 6.041429 |
| GSE39582_GSM972146 | 0.237953 |
| GSE39582_GSM972147 | -7.19326 |
| GSE39582_GSM972148 | -1.67776 |
| GSE39582_GSM972149 | 0.52872 |
| GSE39582_GSM972150 | -0.50024 |
| GSE39582_GSM972151 | -7.38112 |
| GSE39582_GSM972152 | -7.5849 |
| GSE39582_GSM972153 | -5.04594 |
| GSE39582_GSM972154 | -7.71721 |
| GSE39582_GSM972155 | -9.53714 |
| GSE39582_GSM972156 | -1.59955 |
| GSE39582_GSM972157 | -3.75288 |
| GSE39582_GSM972158 | 4.520098 |
| GSE39582_GSM972159 | -6.70338 |
| GSE39582_GSM972160 | 0.905125 |
| GSE39582_GSM972161 | -7.86207 |
| GSE39582_GSM972162 | 3.303522 |
| GSE39582_GSM972163 | 3.292497 |
| GSE39582_GSM972164 | -1.62278 |
| GSE39582_GSM972165 | 3.709011 |
| GSE39582_GSM972166 | -1.06653 |
| GSE39582_GSM972167 | -2.97298 |
| GSE39582_GSM972168 | -0.82308 |
| GSE39582_GSM972169 | -1.28797 |
| GSE39582_GSM972170 | -5.16375 |
| GSE39582_GSM972171 | 2.314073 |
| GSE39582_GSM972172 | 3.440842 |
| GSE39582_GSM972173 | -2.61984 |
| GSE39582_GSM972174 | 1.39593 |
| GSE39582_GSM972175 | -5.01454 |
| GSE39582_GSM972176 | 6.679647 |
| GSE39582_GSM972177 | 11.88781 |
| GSE39582_GSM972178 | 6.788813 |
| GSE39582_GSM972179 | 15.20861 |
| GSE39582_GSM972180 | -8.0863 |
| GSE39582_GSM972181 | 27.27755 |
| GSE39582_GSM972182 | -2.52965 |
| GSE39582_GSM972183 | 0.495452 |
| GSE39582_GSM972184 | 4.834892 |
| GSE39582_GSM972185 | -0.71165 |
| GSE39582_GSM972186 | 20.9649 |
| GSE39582_GSM972187 | -0.44768 |
| GSE39582_GSM972188 | -0.36532 |
| GSE39582_GSM972189 | -9.55649 |
| GSE39582_GSM972190 | -0.80908 |
| GSE39582_GSM972191 | -8.20334 |
| GSE39582_GSM972192 | 2.350862 |
| GSE39582_GSM972193 | 1.749349 |
| GSE39582_GSM972194 | -4.42834 |
| GSE39582_GSM972195 | -8.89825 |
| GSE39582_GSM972196 | -16.0836 |
| GSE39582_GSM972197 | -9.44853 |
| GSE39582_GSM972198 | -12.1068 |
| GSE39582_GSM972199 | -7.73965 |
| GSE39582_GSM972200 | -2.28923 |
| GSE39582_GSM972201 | 16.11798 |
| GSE39582_GSM972202 | -8.77535 |
| GSE39582_GSM972203 | -5.07409 |
| GSE39582_GSM972204 | 2.518461 |
| GSE39582_GSM972205 | -1.195 |
| GSE39582_GSM972206 | 5.700409 |
| GSE39582_GSM972207 | -4.1258 |
| GSE39582_GSM972208 | 12.4508 |
| GSE39582_GSM972209 | -1.06299 |
| GSE39582_GSM972210 | 0.138841 |
| GSE39582_GSM972211 | 5.127259 |
| GSE39582_GSM972212 | -12.6125 |
| GSE39582_GSM972213 | -4.68791 |
| GSE39582_GSM972214 | -15.7089 |
| GSE39582_GSM972215 | -7.33281 |
| GSE39582_GSM972216 | -6.81408 |
| GSE39582_GSM972217 | 0.135698 |
| GSE39582_GSM972218 | -12.2332 |
| GSE39582_GSM972219 | 0.009229 |
| GSE39582_GSM972220 | -0.03854 |
| GSE39582_GSM972221 | -14.6856 |
| GSE39582_GSM972222 | -8.65806 |
| GSE39582_GSM972223 | -1.9572 |
| GSE39582_GSM972224 | 5.629568 |
| GSE39582_GSM972225 | -9.2651 |
| GSE39582_GSM972226 | 4.380987 |
| GSE39582_GSM972227 | -7.83927 |
| GSE39582_GSM972228 | -3.03443 |
| GSE39582_GSM972229 | -7.37572 |
| GSE39582_GSM972230 | -3.41925 |
| GSE39582_GSM972231 | 7.152433 |
| GSE39582_GSM972232 | 8.702549 |
| GSE39582_GSM972233 | -13.2901 |
| GSE39582_GSM972234 | 16.47322 |
| GSE39582_GSM972235 | -2.87859 |
| GSE39582_GSM972236 | -6.78306 |
| GSE39582_GSM972237 | -10.26 |
| GSE39582_GSM972238 | -3.62141 |
| GSE39582_GSM972239 | -11.1521 |
| GSE39582_GSM972240 | 10.49928 |
| GSE39582_GSM972241 | -7.64249 |
| GSE39582_GSM972242 | -0.4437 |
| GSE39582_GSM972243 | 0.778394 |
| GSE39582_GSM972244 | 7.404787 |
| GSE39582_GSM972245 | 2.94988 |
| GSE39582_GSM972246 | -2.19729 |
| GSE39582_GSM972247 | 1.275918 |
| GSE39582_GSM972248 | 1.041315 |
| GSE39582_GSM972249 | -3.11362 |
| GSE39582_GSM972250 | -10.9957 |
| GSE39582_GSM972251 | 10.13812 |
| GSE39582_GSM972252 | -5.58545 |
| GSE39582_GSM972253 | 0.793017 |
| GSE39582_GSM972254 | 14.98651 |
| GSE39582_GSM972255 | 4.655977 |
| GSE39582_GSM972256 | 8.98764 |
| GSE39582_GSM972257 | -5.43109 |
| GSE39582_GSM972258 | 2.825261 |
| GSE39582_GSM972259 | 5.869912 |
| GSE39582_GSM972260 | 4.633857 |
| GSE39582_GSM972261 | -4.77591 |
| GSE39582_GSM972262 | 16.66195 |
| GSE39582_GSM972263 | -5.2858 |
| GSE39582_GSM972264 | -11.5728 |
| GSE39582_GSM972265 | -3.84977 |
| GSE39582_GSM972266 | -12.3711 |
| GSE39582_GSM972267 | 3.219377 |
| GSE39582_GSM972268 | -3.32533 |
| GSE39582_GSM972269 | -1.8882 |
| GSE39582_GSM972270 | -8.40739 |
| GSE39582_GSM972271 | -3.04146 |
| GSE39582_GSM972272 | 5.320029 |
| GSE39582_GSM972273 | 2.590016 |
| GSE39582_GSM972274 | 3.808536 |
| GSE39582_GSM972275 | 9.58818 |
| GSE39582_GSM972276 | -5.46666 |
| GSE39582_GSM972277 | -11.677 |
| GSE39582_GSM972278 | -13.2131 |
| GSE39582_GSM972279 | -4.63407 |
| GSE39582_GSM972280 | -5.6222 |
| GSE39582_GSM972281 | -11.0598 |
| GSE39582_GSM972282 | -5.52505 |
| GSE39582_GSM972283 | 1.349283 |
| GSE39582_GSM972284 | -6.49956 |
| GSE39582_GSM972285 | 2.223051 |
| GSE39582_GSM972286 | -5.02111 |
| GSE39582_GSM972287 | -1.32097 |
| GSE39582_GSM972288 | 7.688528 |
| GSE39582_GSM972289 | -9.42037 |
| GSE39582_GSM972290 | 0.861209 |
| GSE39582_GSM972291 | -2.51725 |
| GSE39582_GSM972292 | -2.5294 |
| GSE39582_GSM972293 | 3.901757 |
| GSE39582_GSM972294 | -3.01057 |
| GSE39582_GSM972295 | 0.840376 |
| GSE39582_GSM972296 | -9.41984 |
| GSE39582_GSM972297 | 8.186139 |
| GSE39582_GSM972298 | -9.96607 |
| GSE39582_GSM972299 | 6.951324 |
| GSE39582_GSM972300 | -0.84807 |
| GSE39582_GSM972301 | 22.54407 |
| GSE39582_GSM972302 | -5.72382 |
| GSE39582_GSM972303 | -13.6495 |
| GSE39582_GSM972304 | -1.55871 |
| GSE39582_GSM972305 | 11.05059 |
| GSE39582_GSM972306 | 0.748526 |
| GSE39582_GSM972307 | -4.72816 |
| GSE39582_GSM972308 | 3.740153 |
| GSE39582_GSM972309 | -13.3129 |
| GSE39582_GSM972310 | -4.00237 |
| GSE39582_GSM972311 | 14.89471 |
| GSE39582_GSM972312 | -7.2404 |
| GSE39582_GSM972313 | -9.92817 |
| GSE39582_GSM972314 | -9.99461 |
| GSE39582_GSM972315 | 1.549174 |
| GSE39582_GSM972316 | -8.46014 |
| GSE39582_GSM972317 | -2.69015 |
| GSE39582_GSM972318 | -4.40463 |
| GSE39582_GSM972319 | -7.54761 |
| GSE39582_GSM972320 | 1.414419 |
| GSE39582_GSM972321 | 0.201854 |
| GSE39582_GSM972322 | 7.23774 |
| GSE39582_GSM972323 | -8.06317 |
| GSE39582_GSM972324 | -4.71899 |
| GSE39582_GSM972325 | -13.1133 |
| GSE39582_GSM972326 | 9.339401 |
| GSE39582_GSM972327 | 13.91725 |
| GSE39582_GSM972328 | -2.4561 |
| GSE39582_GSM972329 | 6.070411 |
| GSE39582_GSM972330 | 1.400235 |
| GSE39582_GSM972331 | -2.43196 |
| GSE39582_GSM972332 | -4.02993 |
| GSE39582_GSM972333 | -4.40005 |
| GSE39582_GSM972334 | -3.19885 |
| GSE39582_GSM972335 | -4.36236 |
| GSE39582_GSM972336 | -4.50624 |
| GSE39582_GSM972337 | 2.112829 |
| GSE39582_GSM972338 | -2.59618 |
| GSE39582_GSM972339 | -8.24278 |
| GSE39582_GSM972340 | 2.791695 |
| GSE39582_GSM972341 | -4.41107 |
| GSE39582_GSM972342 | 7.749964 |
| GSE39582_GSM972343 | -5.38625 |
| GSE39582_GSM972344 | -9.79352 |
| GSE39582_GSM972345 | 23.79085 |
| GSE39582_GSM972346 | -9.09167 |
| GSE39582_GSM972347 | -3.59293 |
| GSE39582_GSM972348 | -9.0028 |
| GSE39582_GSM972349 | 21.84396 |
| GSE39582_GSM972350 | -12.1597 |
| GSE39582_GSM972351 | -4.95076 |
| GSE39582_GSM972352 | 8.340514 |
| GSE39582_GSM972353 | -10.8757 |
| GSE39582_GSM972354 | -9.16042 |
| GSE39582_GSM972355 | 3.410775 |
| GSE39582_GSM972356 | -11.9456 |
| GSE39582_GSM972357 | 26.37872 |
| GSE39582_GSM972358 | -10.4038 |
| GSE39582_GSM972359 | 26.38124 |
| GSE39582_GSM972360 | -9.77753 |
| GSE39582_GSM972361 | 7.105907 |
| GSE39582_GSM972362 | -5.38297 |
| GSE39582_GSM972363 | -8.0118 |
| GSE39582_GSM972364 | -9.73396 |
| GSE39582_GSM972365 | 8.650405 |
| GSE39582_GSM972366 | 12.51594 |
| GSE39582_GSM972367 | 24.78151 |
| GSE39582_GSM972368 | -11.2062 |
| GSE39582_GSM972369 | 15.33731 |
| GSE39582_GSM972370 | -0.85203 |
| GSE39582_GSM972371 | 3.421942 |
| GSE39582_GSM972372 | -1.74571 |
| GSE39582_GSM972373 | 4.016444 |
| GSE39582_GSM972374 | 2.844738 |
| GSE39582_GSM972375 | 6.586086 |
| GSE39582_GSM972376 | 5.429554 |
| GSE39582_GSM972377 | 15.3907 |
| GSE39582_GSM972378 | 12.05397 |
| GSE39582_GSM972379 | 8.1558 |
| GSE39582_GSM972380 | 3.868939 |
| GSE39582_GSM972381 | 16.17987 |
| GSE39582_GSM972382 | -3.89147 |
| GSE39582_GSM972383 | -2.9408 |
| GSE39582_GSM972384 | -4.43726 |
| GSE39582_GSM972385 | 14.08259 |
| GSE39582_GSM972386 | -12.6165 |
| GSE39582_GSM972387 | -11.7551 |
| GSE39582_GSM972388 | -13.6175 |
| GSE39582_GSM972389 | -6.13674 |
| GSE39582_GSM972390 | 2.573857 |
| GSE39582_GSM972391 | -4.91417 |
| GSE39582_GSM972392 | 0.290835 |
| GSE39582_GSM972393 | 2.834141 |
| GSE39582_GSM972394 | 12.58133 |
| GSE39582_GSM972395 | -2.29988 |
| GSE39582_GSM972396 | -0.58747 |
| GSE39582_GSM972397 | -14.9991 |
| GSE39582_GSM972398 | -11.9011 |
| GSE39582_GSM972399 | -6.42348 |
| GSE39582_GSM972400 | 4.469476 |
| GSE39582_GSM972401 | 12.31137 |
| GSE39582_GSM972402 | 5.861786 |
| GSE39582_GSM972403 | -11.0244 |
| GSE39582_GSM972404 | -3.59972 |
| GSE39582_GSM972405 | -1.33879 |
| GSE39582_GSM972406 | -3.17366 |
| GSE39582_GSM972407 | 2.213653 |
| GSE39582_GSM972408 | 1.713398 |
| GSE39582_GSM972409 | 10.93628 |
| GSE39582_GSM972410 | 15.94472 |
| GSE39582_GSM972411 | -11.4681 |
| GSE39582_GSM972412 | 8.865919 |
| GSE39582_GSM972413 | 23.50192 |
| GSE39582_GSM972414 | -7.83811 |
| GSE39582_GSM972415 | -7.89914 |
| GSE39582_GSM972416 | 4.105343 |
| GSE39582_GSM972417 | 1.572629 |
| GSE39582_GSM972418 | -0.86459 |
| GSE39582_GSM972419 | -3.58158 |
| GSE39582_GSM972420 | 10.63276 |
| GSE39582_GSM972421 | 16.41519 |
| GSE39582_GSM972422 | 19.88297 |
| GSE39582_GSM972423 | 7.744462 |
| GSE39582_GSM972424 | 2.959201 |
| GSE39582_GSM972425 | 6.319316 |
| GSE39582_GSM972426 | 14.43054 |
| GSE39582_GSM972427 | 8.914668 |
| GSE39582_GSM972428 | 12.9047 |
| GSE39582_GSM972429 | 0.329639 |
| GSE39582_GSM972430 | -4.21492 |
| GSE39582_GSM972431 | -5.06468 |
| GSE39582_GSM972432 | 5.783762 |
| GSE39582_GSM972433 | 12.91817 |
| GSE39582_GSM972434 | -7.76695 |
| GSE39582_GSM972435 | -0.00223 |
| GSE39582_GSM972436 | -2.88974 |
| GSE39582_GSM972437 | 10.16757 |
| GSE39582_GSM972438 | 15.91438 |
| GSE39582_GSM972439 | 7.982572 |
| GSE39582_GSM972440 | 4.172815 |
| GSE39582_GSM972441 | 12.1548 |
| GSE39582_GSM972442 | 0.07721 |
| GSE39582_GSM972443 | -5.7629 |
| GSE39582_GSM972444 | 7.971881 |
| GSE39582_GSM972445 | -6.80114 |
| GSE39582_GSM972446 | -3.85598 |
| GSE39582_GSM972447 | -1.46777 |
| GSE39582_GSM972448 | -3.52514 |
| GSE39582_GSM972449 | -0.2232 |
| GSE39582_GSM972450 | -5.54435 |
| GSE39582_GSM972451 | -6.90247 |
| GSE39582_GSM972452 | -9.38859 |
| GSE39582_GSM972453 | -6.64825 |
| GSE39582_GSM972454 | -1.70629 |
| GSE39582_GSM972455 | 7.218187 |
| GSE39582_GSM972456 | -0.02968 |
| GSE39582_GSM972457 | 6.474299 |
| GSE39582_GSM972458 | -7.35324 |
| GSE39582_GSM972459 | -1.66964 |
| GSE39582_GSM972460 | -0.19992 |
| GSE39582_GSM972461 | -4.30105 |
| GSE39582_GSM972462 | -6.66104 |
| GSE39582_GSM972463 | 9.682266 |
| GSE39582_GSM972464 | 10.60975 |
| GSE39582_GSM972465 | -16.1182 |
| GSE39582_GSM972466 | -15.6298 |
| GSE39582_GSM972467 | 28.30226 |
| GSE39582_GSM972468 | -1.41314 |
| GSE39582_GSM972469 | -1.90442 |
| GSE39582_GSM972470 | 3.688278 |
| GSE39582_GSM972471 | -5.87487 |
| GSE39582_GSM972472 | -2.67589 |
| GSE39582_GSM972473 | -4.49661 |
| GSE39582_GSM972474 | -2.72131 |
| GSE39582_GSM972475 | 1.907357 |
| GSE39582_GSM972476 | 15.49147 |
| GSE39582_GSM972477 | -7.01039 |
| GSE39582_GSM972478 | 0.117838 |
| GSE39582_GSM972479 | 30.47553 |
| GSE39582_GSM972480 | -4.74846 |
| GSE39582_GSM972481 | 3.063997 |
| GSE39582_GSM972482 | 4.689715 |
| GSE39582_GSM972483 | 3.374331 |
| GSE39582_GSM972484 | -3.07081 |
| GSE39582_GSM972485 | 4.874587 |
| GSE39582_GSM972486 | 10.19772 |
| GSE39582_GSM972487 | 2.907441 |
| GSE39582_GSM972488 | -0.88358 |
| GSE39582_GSM972489 | 3.337686 |
| GSE39582_GSM972490 | -13.6439 |
| GSE39582_GSM972491 | -8.73353 |
| GSE39582_GSM972492 | -11.9667 |
| GSE39582_GSM972493 | 2.232848 |
| GSE39582_GSM972494 | 1.881577 |
| GSE39582_GSM972495 | 6.322144 |
| GSE39582_GSM972496 | -3.46508 |
| GSE39582_GSM972497 | -7.4271 |
| GSE39582_GSM972498 | -13.01 |
| GSE39582_GSM972499 | -19.1609 |
| GSE39582_GSM972500 | 28.99482 |
| GSE39582_GSM972501 | 3.079588 |
| GSE39582_GSM972502 | -0.5501 |
| GSE39582_GSM972503 | 12.75743 |
| GSE39582_GSM972504 | 10.8386 |
| GSE39582_GSM972505 | 7.453334 |
| GSE39582_GSM972506 | 8.669948 |
| GSE39582_GSM972507 | -7.14862 |
| GSE39582_GSM972508 | -8.8347 |
| GSE39582_GSM972509 | -4.55306 |
| GSE39582_GSM972510 | 1.240591 |
| GSE39582_GSM972511 | -10.9666 |
| GSE39582_GSM972512 | -3.5175 |
| GSE39582_GSM972513 | -4.95067 |
| GSE39582_GSM972514 | -1.5804 |
| GSE39582_GSM972515 | -13.645 |
| GSE39582_GSM972516 | -7.01077 |
| GSE39582_GSM972517 | -1.95215 |
| GSE39582_GSM972518 | 10.23909 |
| GSE39582_GSM972519 | 5.04469 |
| GSE39582_GSM972520 | -11.7314 |
| GSE39582_GSM972521 | 5.420921 |
| GSE39582_GSM972522 | 14.2594 |

**Supplementary Table S11** | The IPS of TCGA samples.

| barcode | ips_ctla4_neg_pd1_neg | ips_ctla4_neg_pd1_pos | ips_ctla4_pos_pd1_neg | ips_ctla4_pos_pd1_pos |
| --- | --- | --- | --- | --- |
| TCGA-QG-A5YX | 10 | 8 | 10 | 8 |
| TCGA-G4-6306 | 10 | 9 | 10 | 9 |
| TCGA-AA-A02E | 10 | 8 | 9 | 8 |
| TCGA-AA-A00L | 10 | 8 | 10 | 8 |
| TCGA-G4-6626 | 10 | 8 | 9 | 8 |
| TCGA-AZ-4308 | 10 | 8 | 9 | 8 |
| TCGA-DM-A28K | 10 | 8 | 10 | 8 |
| TCGA-AA-3531 | 10 | 9 | 10 | 8 |
| TCGA-G4-6294 | 10 | 8 | 9 | 7 |
| TCGA-AD-6963 | 10 | 9 | 10 | 9 |
| TCGA-A6-5661 | 10 | 8 | 9 | 8 |
| TCGA-AA-3530 | 10 | 8 | 9 | 8 |
| TCGA-AA-3851 | 10 | 8 | 9 | 8 |
| TCGA-A6-2679 | 10 | 10 | 10 | 10 |
| TCGA-AA-3837 | 10 | 8 | 9 | 7 |
| TCGA-AA-3971 | 10 | 8 | 9 | 8 |
| TCGA-AZ-4313 | 10 | 9 | 10 | 8 |
| TCGA-AA-3552 | 10 | 9 | 10 | 8 |
| TCGA-AA-3527 | 10 | 10 | 10 | 9 |
| TCGA-D5-6930 | 10 | 9 | 10 | 9 |
| TCGA-AA-A03F | 10 | 8 | 10 | 8 |
| TCGA-AA-3688 | 10 | 8 | 9 | 8 |
| TCGA-DM-A28F | 10 | 8 | 9 | 7 |
| TCGA-AY-4071 | 10 | 9 | 10 | 9 |
| TCGA-AA-3862 | 10 | 9 | 10 | 9 |
| TCGA-NH-A6GC | 10 | 8 | 9 | 7 |
| TCGA-AA-3519 | 10 | 8 | 9 | 8 |
| TCGA-AA-3502 | 10 | 8 | 9 | 7 |
| TCGA-AY-5543 | 10 | 8 | 9 | 8 |
| TCGA-DM-A1D4 | 10 | 8 | 9 | 7 |
| TCGA-QG-A5Z2 | 10 | 9 | 10 | 9 |
| TCGA-AA-A01S | 10 | 8 | 9 | 7 |
| TCGA-DM-A28E | 10 | 8 | 9 | 7 |
| TCGA-4N-A93T | 10 | 8 | 9 | 7 |
| TCGA-DM-A1D8 | 10 | 9 | 10 | 8 |
| TCGA-AA-A01I | 10 | 8 | 10 | 8 |
| TCGA-A6-5656 | 10 | 8 | 9 | 7 |
| TCGA-AA-3848 | 10 | 8 | 9 | 8 |
| TCGA-AA-A029 | 10 | 8 | 9 | 8 |
| TCGA-AA-3856 | 10 | 9 | 9 | 8 |
| TCGA-G4-6323 | 10 | 8 | 9 | 8 |
| TCGA-CM-5861 | 10 | 9 | 10 | 8 |
| TCGA-AA-A01R | 10 | 10 | 10 | 10 |
| TCGA-D5-6532 | 10 | 8 | 9 | 7 |
| TCGA-AA-3518 | 10 | 9 | 9 | 8 |
| TCGA-CK-5912 | 10 | 8 | 9 | 7 |
| TCGA-AA-3516 | 10 | 10 | 10 | 10 |
| TCGA-AA-3846 | 10 | 9 | 10 | 9 |
| TCGA-G4-6320 | 10 | 8 | 9 | 7 |
| TCGA-DM-A1DA | 10 | 8 | 9 | 7 |
| TCGA-AA-3672 | 10 | 10 | 10 | 10 |
| TCGA-DM-A28M | 10 | 8 | 9 | 7 |
| TCGA-AY-6197 | 10 | 8 | 9 | 8 |
| TCGA-AA-A01T | 10 | 9 | 10 | 9 |
| TCGA-AA-A00W | 10 | 8 | 9 | 7 |
| TCGA-AA-A01G | 10 | 8 | 9 | 7 |
| TCGA-AZ-4681 | 10 | 8 | 9 | 7 |
| TCGA-A6-2683 | 10 | 8 | 9 | 8 |
| TCGA-AZ-6599 | 10 | 8 | 9 | 7 |
| TCGA-CM-6675 | 10 | 8 | 9 | 7 |
| TCGA-CK-6747 | 10 | 8 | 9 | 8 |
| TCGA-DM-A0X9 | 10 | 8 | 9 | 8 |
| TCGA-AA-A022 | 10 | 10 | 10 | 10 |
| TCGA-CK-6751 | 10 | 8 | 9 | 8 |
| TCGA-CM-4752 | 10 | 9 | 10 | 9 |
| TCGA-AD-6895 | 10 | 9 | 9 | 9 |
| TCGA-AA-3844 | 10 | 8 | 9 | 8 |
| TCGA-T9-A92H | 10 | 9 | 10 | 8 |
| TCGA-A6-3807 | 10 | 9 | 9 | 8 |
| TCGA-CA-5255 | 10 | 8 | 9 | 8 |
| TCGA-DM-A1HA | 10 | 9 | 10 | 8 |
| TCGA-CK-5914 | 10 | 9 | 10 | 8 |
| TCGA-AA-3861 | 10 | 8 | 9 | 8 |
| TCGA-RU-A8FL | 10 | 8 | 9 | 7 |
| TCGA-D5-6929 | 10 | 8 | 9 | 8 |
| TCGA-F4-6856 | 10 | 8 | 9 | 8 |
| TCGA-CA-5796 | 10 | 8 | 9 | 8 |
| TCGA-A6-6653 | 10 | 9 | 10 | 8 |
| TCGA-G4-6321 | 10 | 9 | 10 | 9 |
| TCGA-AA-3855 | 10 | 8 | 9 | 8 |
| TCGA-DM-A0XD | 10 | 8 | 9 | 7 |
| TCGA-DM-A1D6 | 10 | 8 | 9 | 7 |
| TCGA-AA-3679 | 10 | 8 | 9 | 7 |
| TCGA-AA-A004 | 10 | 9 | 10 | 9 |
| TCGA-D5-5537 | 10 | 8 | 9 | 7 |
| TCGA-CM-4750 | 10 | 9 | 10 | 8 |
| TCGA-F4-6806 | 10 | 9 | 10 | 8 |
| TCGA-AZ-6606 | 10 | 8 | 9 | 8 |
| TCGA-CM-4743 | 10 | 10 | 10 | 10 |
| TCGA-NH-A6GB | 10 | 8 | 9 | 8 |
| TCGA-QL-A97D | 10 | 9 | 10 | 9 |
| TCGA-A6-4107 | 10 | 8 | 9 | 7 |
| TCGA-AA-A01Z | 10 | 8 | 9 | 7 |
| TCGA-D5-6535 | 10 | 9 | 10 | 9 |
| TCGA-CM-4751 | 10 | 9 | 9 | 9 |
| TCGA-AZ-4682 | 10 | 8 | 9 | 7 |
| TCGA-AA-3525 | 10 | 9 | 10 | 8 |
| TCGA-AA-3684 | 10 | 9 | 9 | 8 |
| TCGA-AA-3522 | 10 | 8 | 9 | 8 |
| TCGA-CM-4744 | 10 | 10 | 10 | 9 |
| TCGA-AA-A03J | 10 | 9 | 10 | 9 |
| TCGA-AZ-4616 | 10 | 9 | 9 | 9 |
| TCGA-G4-6322 | 10 | 8 | 9 | 8 |
| TCGA-CM-4748 | 10 | 8 | 9 | 7 |
| TCGA-AA-A02Y | 10 | 9 | 9 | 8 |
| TCGA-A6-2680 | 10 | 8 | 9 | 8 |
| TCGA-AG-A023 | 10 | 8 | 10 | 8 |
| TCGA-EI-6883 | 10 | 8 | 9 | 8 |
| TCGA-AG-A020 | 10 | 8 | 9 | 7 |
| TCGA-DC-5337 | 10 | 8 | 9 | 8 |
| TCGA-DC-6682 | 10 | 8 | 9 | 8 |
| TCGA-AG-3599 | 10 | 9 | 10 | 8 |
| TCGA-EI-6510 | 10 | 8 | 9 | 8 |
| TCGA-AG-A01L | 10 | 8 | 9 | 7 |
| TCGA-AG-3728 | 10 | 9 | 10 | 9 |
| TCGA-AG-3882 | 10 | 9 | 10 | 9 |
| TCGA-AG-A02G | 10 | 8 | 9 | 8 |
| TCGA-AG-A032 | 10 | 8 | 10 | 8 |
| TCGA-AG-3602 | 10 | 9 | 10 | 8 |
| TCGA-AG-A036 | 10 | 8 | 10 | 8 |
| TCGA-G5-6235 | 10 | 8 | 9 | 8 |
| TCGA-AG-3608 | 10 | 9 | 10 | 8 |
| TCGA-AG-3598 | 10 | 9 | 9 | 8 |
| TCGA-AG-3611 | 10 | 9 | 10 | 8 |
| TCGA-AG-3909 | 10 | 8 | 9 | 7 |
| TCGA-DY-A1DG | 10 | 9 | 10 | 8 |
| TCGA-AG-3887 | 10 | 8 | 9 | 8 |
| TCGA-AG-4007 | 10 | 8 | 9 | 8 |
| TCGA-AF-2691 | 10 | 8 | 9 | 8 |
| TCGA-AH-6544 | 10 | 9 | 10 | 8 |
| TCGA-AG-3890 | 10 | 8 | 10 | 8 |
| TCGA-CI-6622 | 10 | 8 | 9 | 7 |
| TCGA-AG-A01J | 10 | 8 | 9 | 7 |
| TCGA-DC-4749 | 10 | 8 | 9 | 7 |
| TCGA-AG-3591 | 10 | 8 | 9 | 8 |
| TCGA-CL-4957 | 10 | 8 | 9 | 7 |
| TCGA-F5-6812 | 10 | 8 | 9 | 8 |
| TCGA-AG-3885 | 10 | 9 | 9 | 8 |
| TCGA-AG-A02X | 10 | 8 | 9 | 7 |
| TCGA-AG-A00C | 10 | 9 | 10 | 8 |
| TCGA-A6-6137 | 9 | 8 | 9 | 8 |
| TCGA-AU-6004 | 9 | 8 | 9 | 8 |
| TCGA-AZ-4614 | 9 | 7 | 9 | 7 |
| TCGA-AA-3693 | 9 | 7 | 8 | 6 |
| TCGA-AA-3510 | 9 | 8 | 9 | 8 |
| TCGA-AY-6386 | 9 | 8 | 9 | 7 |
| TCGA-5M-AAT5 | 9 | 7 | 9 | 7 |
| TCGA-CK-5913 | 9 | 8 | 9 | 8 |
| TCGA-AA-3980 | 9 | 8 | 9 | 8 |
| TCGA-A6-6649 | 9 | 7 | 8 | 7 |
| TCGA-AA-3869 | 9 | 8 | 9 | 8 |
| TCGA-CA-5797 | 9 | 7 | 8 | 7 |
| TCGA-A6-6782 | 9 | 7 | 8 | 7 |
| TCGA-AA-3675 | 9 | 7 | 8 | 7 |
| TCGA-AA-3858 | 9 | 8 | 9 | 7 |
| TCGA-AA-3812 | 9 | 8 | 9 | 7 |
| TCGA-A6-A5ZU | 9 | 8 | 8 | 7 |
| TCGA-NH-A50T | 9 | 7 | 9 | 7 |
| TCGA-F4-6808 | 9 | 7 | 9 | 7 |
| TCGA-CM-5868 | 9 | 7 | 9 | 7 |
| TCGA-A6-6138 | 9 | 9 | 9 | 9 |
| TCGA-AA-A00A | 9 | 8 | 9 | 8 |
| TCGA-NH-A8F8 | 9 | 7 | 8 | 7 |
| TCGA-AA-A02K | 9 | 7 | 8 | 6 |
| TCGA-AA-3872 | 9 | 7 | 8 | 7 |
| TCGA-AA-3562 | 9 | 7 | 9 | 7 |
| TCGA-AA-A01F | 9 | 8 | 9 | 7 |
| TCGA-AA-3850 | 9 | 8 | 9 | 8 |
| TCGA-A6-6140 | 9 | 8 | 9 | 7 |
| TCGA-G4-6299 | 9 | 8 | 9 | 8 |
| TCGA-AA-3821 | 9 | 8 | 9 | 8 |
| TCGA-AA-A00E | 9 | 8 | 9 | 8 |
| TCGA-A6-5666 | 9 | 7 | 8 | 6 |
| TCGA-CA-5254 | 9 | 8 | 9 | 7 |
| TCGA-G4-6293 | 9 | 8 | 9 | 8 |
| TCGA-CM-6172 | 9 | 8 | 9 | 7 |
| TCGA-AA-3506 | 9 | 7 | 8 | 7 |
| TCGA-F4-6461 | 9 | 8 | 9 | 8 |
| TCGA-F4-6460 | 9 | 7 | 8 | 7 |
| TCGA-AA-3712 | 9 | 8 | 9 | 7 |
| TCGA-G4-6628 | 9 | 9 | 9 | 9 |
| TCGA-AA-3560 | 9 | 8 | 9 | 7 |
| TCGA-CM-5341 | 9 | 7 | 8 | 7 |
| TCGA-AA-3548 | 9 | 8 | 9 | 7 |
| TCGA-AA-3561 | 9 | 7 | 9 | 7 |
| TCGA-AA-3655 | 9 | 7 | 8 | 7 |
| TCGA-AA-3866 | 9 | 8 | 8 | 8 |
| TCGA-AA-3524 | 9 | 8 | 9 | 8 |
| TCGA-AA-3877 | 9 | 8 | 9 | 8 |
| TCGA-AA-3986 | 9 | 8 | 9 | 8 |
| TCGA-AA-A00D | 9 | 9 | 9 | 9 |
| TCGA-AA-A00U | 9 | 7 | 8 | 7 |
| TCGA-AA-A01P | 9 | 9 | 9 | 9 |
| TCGA-AD-6888 | 9 | 7 | 8 | 7 |
| TCGA-DM-A288 | 9 | 7 | 8 | 6 |
| TCGA-G4-6310 | 9 | 7 | 8 | 6 |
| TCGA-AA-3976 | 9 | 7 | 8 | 7 |
| TCGA-AZ-6605 | 9 | 8 | 8 | 7 |
| TCGA-A6-A567 | 9 | 7 | 8 | 7 |
| TCGA-AA-3495 | 9 | 7 | 8 | 7 |
| TCGA-AA-3973 | 9 | 8 | 9 | 7 |
| TCGA-G4-6588 | 9 | 7 | 8 | 7 |
| TCGA-F4-6807 | 9 | 8 | 9 | 8 |
| TCGA-AA-3972 | 9 | 7 | 8 | 7 |
| TCGA-AA-3511 | 9 | 7 | 9 | 7 |
| TCGA-CA-5256 | 9 | 8 | 9 | 7 |
| TCGA-AA-3526 | 9 | 8 | 9 | 8 |
| TCGA-AD-6889 | 9 | 8 | 9 | 7 |
| TCGA-A6-5662 | 9 | 8 | 8 | 7 |
| TCGA-AA-A01V | 9 | 9 | 10 | 9 |
| TCGA-AA-A00K | 9 | 7 | 9 | 7 |
| TCGA-CK-6746 | 9 | 9 | 9 | 9 |
| TCGA-AA-A01K | 9 | 8 | 9 | 8 |
| TCGA-G4-6627 | 9 | 8 | 9 | 7 |
| TCGA-AA-3819 | 9 | 7 | 8 | 6 |
| TCGA-A6-6652 | 9 | 7 | 9 | 7 |
| TCGA-CM-5860 | 9 | 8 | 9 | 8 |
| TCGA-CM-6677 | 9 | 7 | 8 | 7 |
| TCGA-A6-5659 | 9 | 7 | 8 | 6 |
| TCGA-AA-3975 | 9 | 7 | 9 | 7 |
| TCGA-AA-3532 | 9 | 9 | 9 | 9 |
| TCGA-DM-A28A | 9 | 7 | 8 | 7 |
| TCGA-AA-3681 | 9 | 8 | 8 | 7 |
| TCGA-AA-3860 | 9 | 8 | 9 | 8 |
| TCGA-AY-A69D | 9 | 7 | 8 | 7 |
| TCGA-AZ-4323 | 9 | 8 | 9 | 8 |
| TCGA-F4-6704 | 9 | 8 | 8 | 7 |
| TCGA-AY-A8YK | 9 | 7 | 9 | 7 |
| TCGA-AZ-6608 | 9 | 7 | 9 | 7 |
| TCGA-AD-6548 | 9 | 8 | 8 | 7 |
| TCGA-AA-3952 | 9 | 7 | 8 | 6 |
| TCGA-AA-3811 | 9 | 8 | 9 | 8 |
| TCGA-AY-A54L | 9 | 7 | 8 | 6 |
| TCGA-A6-2677 | 9 | 7 | 8 | 6 |
| TCGA-AA-3814 | 9 | 7 | 8 | 7 |
| TCGA-AA-3680 | 9 | 7 | 9 | 7 |
| TCGA-CK-4950 | 9 | 8 | 9 | 8 |
| TCGA-AA-3662 | 9 | 8 | 8 | 7 |
| TCGA-AA-3710 | 9 | 9 | 8 | 8 |
| TCGA-AA-3517 | 9 | 7 | 9 | 7 |
| TCGA-A6-5657 | 9 | 8 | 9 | 8 |
| TCGA-D5-6920 | 9 | 8 | 9 | 7 |
| TCGA-AA-3685 | 9 | 8 | 9 | 8 |
| TCGA-CM-6164 | 9 | 8 | 9 | 7 |
| TCGA-AY-4070 | 9 | 7 | 9 | 7 |
| TCGA-A6-A565 | 9 | 8 | 9 | 8 |
| TCGA-AA-3697 | 9 | 8 | 9 | 8 |
| TCGA-AA-3514 | 9 | 7 | 9 | 7 |
| TCGA-DM-A28C | 9 | 7 | 8 | 6 |
| TCGA-A6-6650 | 9 | 7 | 9 | 7 |
| TCGA-CA-6718 | 9 | 9 | 9 | 9 |
| TCGA-AA-3660 | 9 | 7 | 9 | 7 |
| TCGA-D5-6533 | 9 | 7 | 8 | 7 |
| TCGA-AA-A02O | 9 | 7 | 9 | 7 |
| TCGA-AA-3852 | 9 | 8 | 9 | 7 |
| TCGA-DM-A1D7 | 9 | 7 | 9 | 7 |
| TCGA-AU-3779 | 9 | 8 | 9 | 8 |
| TCGA-G4-6302 | 9 | 7 | 8 | 7 |
| TCGA-G4-6295 | 9 | 8 | 9 | 8 |
| TCGA-CM-6680 | 9 | 8 | 8 | 7 |
| TCGA-AA-3556 | 9 | 8 | 9 | 8 |
| TCGA-AA-3818 | 9 | 7 | 8 | 7 |
| TCGA-AA-3970 | 9 | 8 | 9 | 8 |
| TCGA-AA-A02W | 9 | 7 | 8 | 6 |
| TCGA-AA-A02J | 9 | 7 | 8 | 6 |
| TCGA-CK-4947 | 9 | 8 | 9 | 8 |
| TCGA-AA-3994 | 9 | 8 | 9 | 8 |
| TCGA-AA-A02R | 9 | 9 | 9 | 8 |
| TCGA-CM-6679 | 9 | 7 | 8 | 7 |
| TCGA-CA-6715 | 9 | 7 | 8 | 6 |
| TCGA-AA-3854 | 9 | 7 | 8 | 7 |
| TCGA-AA-3956 | 9 | 8 | 9 | 7 |
| TCGA-A6-2681 | 9 | 7 | 8 | 7 |
| TCGA-AA-3841 | 9 | 8 | 9 | 8 |
| TCGA-A6-2676 | 9 | 9 | 9 | 9 |
| TCGA-A6-3808 | 9 | 8 | 9 | 8 |
| TCGA-AA-3842 | 9 | 8 | 9 | 7 |
| TCGA-CM-6163 | 9 | 7 | 8 | 7 |
| TCGA-AA-A01C | 9 | 8 | 9 | 7 |
| TCGA-AA-3939 | 9 | 7 | 9 | 7 |
| TCGA-AA-3930 | 9 | 8 | 9 | 7 |
| TCGA-NH-A6GA | 9 | 7 | 9 | 7 |
| TCGA-AA-3941 | 9 | 7 | 9 | 7 |
| TCGA-AA-3875 | 9 | 7 | 8 | 7 |
| TCGA-CM-6674 | 9 | 8 | 9 | 7 |
| TCGA-G4-6304 | 9 | 8 | 9 | 7 |
| TCGA-AZ-6603 | 9 | 7 | 8 | 7 |
| TCGA-A6-6141 | 9 | 8 | 8 | 7 |
| TCGA-AA-3982 | 9 | 8 | 9 | 7 |
| TCGA-CM-5864 | 9 | 8 | 9 | 7 |
| TCGA-AA-3955 | 9 | 7 | 8 | 7 |
| TCGA-D5-6931 | 9 | 8 | 8 | 7 |
| TCGA-G4-6625 | 9 | 9 | 9 | 9 |
| TCGA-AA-3520 | 9 | 8 | 9 | 7 |
| TCGA-AA-A00F | 9 | 7 | 8 | 7 |
| TCGA-G4-6297 | 9 | 8 | 9 | 8 |
| TCGA-AA-3673 | 9 | 7 | 8 | 7 |
| TCGA-AA-A00Q | 9 | 7 | 8 | 7 |
| TCGA-A6-6648 | 9 | 8 | 9 | 7 |
| TCGA-DM-A280 | 9 | 7 | 8 | 7 |
| TCGA-AA-A02H | 9 | 7 | 9 | 7 |
| TCGA-F4-6809 | 9 | 8 | 9 | 7 |
| TCGA-AA-3544 | 9 | 8 | 9 | 8 |
| TCGA-AD-6890 | 9 | 8 | 9 | 8 |
| TCGA-AD-A5EK | 9 | 7 | 8 | 6 |
| TCGA-CM-5344 | 9 | 7 | 8 | 6 |
| TCGA-G4-6317 | 9 | 7 | 8 | 7 |
| TCGA-4T-AA8H | 9 | 7 | 9 | 7 |
| TCGA-AD-6899 | 9 | 8 | 8 | 7 |
| TCGA-AZ-5407 | 9 | 7 | 9 | 7 |
| TCGA-A6-3810 | 9 | 8 | 9 | 7 |
| TCGA-DM-A0XF | 9 | 7 | 8 | 7 |
| TCGA-AA-3553 | 9 | 8 | 9 | 8 |
| TCGA-NH-A50U | 9 | 7 | 8 | 7 |
| TCGA-F4-6569 | 9 | 7 | 8 | 7 |
| TCGA-QG-A5YV | 9 | 7 | 8 | 7 |
| TCGA-AD-6965 | 9 | 8 | 9 | 7 |
| TCGA-CM-6167 | 9 | 7 | 8 | 7 |
| TCGA-QG-A5YW | 9 | 8 | 9 | 7 |
| TCGA-AA-3845 | 9 | 9 | 8 | 9 |
| TCGA-AA-3715 | 9 | 9 | 9 | 9 |
| TCGA-AZ-6600 | 9 | 8 | 9 | 7 |
| TCGA-AA-3979 | 9 | 8 | 9 | 7 |
| TCGA-AA-A00Z | 9 | 8 | 9 | 7 |
| TCGA-AY-A71X | 9 | 7 | 9 | 7 |
| TCGA-AA-A024 | 9 | 8 | 9 | 8 |
| TCGA-AA-3989 | 9 | 8 | 8 | 7 |
| TCGA-AA-3667 | 9 | 8 | 9 | 7 |
| TCGA-D5-6539 | 9 | 8 | 9 | 7 |
| TCGA-G4-6315 | 9 | 7 | 8 | 6 |
| TCGA-AA-3968 | 9 | 8 | 8 | 7 |
| TCGA-AZ-4684 | 9 | 7 | 8 | 7 |
| TCGA-AA-3870 | 9 | 8 | 8 | 8 |
| TCGA-DM-A1D9 | 9 | 7 | 8 | 6 |
| TCGA-AZ-4615 | 9 | 9 | 9 | 8 |
| TCGA-CM-6678 | 9 | 7 | 8 | 6 |
| TCGA-DM-A1DB | 9 | 8 | 9 | 7 |
| TCGA-G4-6309 | 9 | 8 | 9 | 7 |
| TCGA-D5-6531 | 9 | 8 | 8 | 8 |
| TCGA-D5-6540 | 9 | 8 | 9 | 7 |
| TCGA-CM-5863 | 9 | 7 | 8 | 6 |
| TCGA-AA-3549 | 9 | 8 | 8 | 7 |
| TCGA-AA-3666 | 9 | 8 | 9 | 8 |
| TCGA-5M-AATE | 9 | 7 | 9 | 7 |
| TCGA-DM-A282 | 9 | 7 | 8 | 6 |
| TCGA-5M-AATA | 9 | 7 | 8 | 7 |
| TCGA-DC-6154 | 9 | 7 | 8 | 6 |
| TCGA-CI-6624 | 9 | 7 | 8 | 7 |
| TCGA-EI-6881 | 9 | 8 | 9 | 8 |
| TCGA-AG-A011 | 9 | 7 | 8 | 7 |
| TCGA-DC-6681 | 9 | 8 | 9 | 7 |
| TCGA-EI-6514 | 9 | 8 | 9 | 7 |
| TCGA-AG-3593 | 9 | 7 | 8 | 7 |
| TCGA-AH-6903 | 9 | 8 | 9 | 7 |
| TCGA-AG-A01N | 9 | 6 | 8 | 6 |
| TCGA-DY-A1DC | 9 | 7 | 9 | 7 |
| TCGA-AG-3592 | 9 | 8 | 9 | 7 |
| TCGA-AG-3726 | 9 | 7 | 8 | 7 |
| TCGA-EI-6512 | 9 | 7 | 8 | 7 |
| TCGA-DC-6160 | 9 | 8 | 9 | 7 |
| TCGA-AF-2693 | 9 | 8 | 9 | 8 |
| TCGA-AG-3581 | 9 | 7 | 8 | 7 |
| TCGA-AG-A026 | 9 | 7 | 8 | 6 |
| TCGA-AG-4015 | 9 | 8 | 9 | 7 |
| TCGA-AG-4022 | 9 | 7 | 9 | 7 |
| TCGA-AG-A015 | 9 | 7 | 8 | 7 |
| TCGA-G5-6641 | 9 | 7 | 9 | 7 |
| TCGA-F5-6571 | 9 | 8 | 8 | 8 |
| TCGA-AG-3612 | 9 | 7 | 8 | 7 |
| TCGA-DC-6683 | 9 | 7 | 8 | 6 |
| TCGA-AG-4005 | 9 | 7 | 8 | 7 |
| TCGA-AF-3911 | 9 | 8 | 9 | 7 |
| TCGA-EF-5830 | 9 | 7 | 8 | 7 |
| TCGA-AG-3605 | 9 | 7 | 8 | 7 |
| TCGA-AG-3732 | 9 | 8 | 9 | 8 |
| TCGA-EI-6513 | 9 | 7 | 8 | 7 |
| TCGA-AG-3586 | 9 | 8 | 9 | 8 |
| TCGA-F5-6813 | 9 | 8 | 9 | 8 |
| TCGA-AG-3896 | 9 | 8 | 9 | 7 |
| TCGA-CI-6623 | 9 | 7 | 8 | 7 |
| TCGA-AH-6547 | 9 | 8 | 8 | 7 |
| TCGA-AG-A016 | 9 | 7 | 9 | 7 |
| TCGA-AG-3582 | 9 | 8 | 8 | 7 |
| TCGA-CL-5918 | 9 | 7 | 9 | 7 |
| TCGA-AG-3892 | 9 | 9 | 9 | 9 |
| TCGA-AG-3725 | 9 | 8 | 9 | 8 |
| TCGA-AG-3878 | 9 | 8 | 9 | 8 |
| TCGA-AG-4008 | 9 | 7 | 8 | 7 |
| TCGA-AG-A01W | 9 | 8 | 9 | 7 |
| TCGA-F5-6861 | 9 | 7 | 8 | 6 |
| TCGA-EF-5831 | 9 | 7 | 8 | 7 |
| TCGA-AG-A02N | 9 | 8 | 9 | 7 |
| TCGA-DY-A1DD | 9 | 7 | 8 | 6 |
| TCGA-AG-3609 | 9 | 8 | 9 | 8 |
| TCGA-AG-A014 | 9 | 7 | 8 | 7 |
| TCGA-DY-A1DE | 9 | 8 | 9 | 8 |
| TCGA-AG-3580 | 9 | 8 | 9 | 7 |
| TCGA-AG-3898 | 9 | 8 | 9 | 7 |
| TCGA-AG-3583 | 9 | 8 | 9 | 7 |
| TCGA-AF-6136 | 9 | 8 | 9 | 7 |
| TCGA-AG-3575 | 9 | 9 | 9 | 8 |
| TCGA-AF-4110 | 9 | 8 | 9 | 8 |
| TCGA-AF-6672 | 9 | 8 | 9 | 7 |
| TCGA-DC-6157 | 9 | 7 | 8 | 7 |
| TCGA-DC-4745 | 9 | 7 | 9 | 7 |
| TCGA-AG-3881 | 9 | 8 | 9 | 8 |
| TCGA-AG-3883 | 9 | 8 | 9 | 8 |
| TCGA-AG-A025 | 9 | 8 | 9 | 7 |
| TCGA-AG-3893 | 9 | 8 | 9 | 7 |
| TCGA-AG-A00Y | 9 | 8 | 9 | 7 |
| TCGA-AG-A00H | 9 | 7 | 8 | 6 |
| TCGA-EI-6882 | 9 | 8 | 8 | 7 |
| TCGA-EI-6506 | 9 | 8 | 9 | 8 |
| TCGA-AG-3902 | 9 | 9 | 9 | 9 |
| TCGA-AF-A56L | 9 | 7 | 8 | 7 |
| TCGA-AG-3587 | 9 | 8 | 9 | 7 |
| TCGA-AG-3594 | 9 | 9 | 9 | 8 |
| TCGA-DY-A1DF | 9 | 7 | 8 | 6 |
| TCGA-F5-6465 | 9 | 8 | 8 | 8 |
| TCGA-CI-6620 | 9 | 7 | 8 | 7 |
| TCGA-EI-6884 | 9 | 8 | 9 | 7 |
| TCGA-F5-6811 | 9 | 8 | 9 | 7 |
| TCGA-AG-3727 | 9 | 8 | 8 | 7 |
| TCGA-DC-5869 | 9 | 7 | 8 | 7 |
| TCGA-BM-6198 | 9 | 8 | 9 | 8 |
| TCGA-AG-A008 | 9 | 8 | 9 | 7 |
| TCGA-G5-6233 | 9 | 8 | 9 | 7 |
| TCGA-AA-3977 | 8 | 7 | 8 | 7 |
| TCGA-CM-6161 | 8 | 7 | 8 | 6 |
| TCGA-CK-5916 | 8 | 7 | 7 | 7 |
| TCGA-D5-6541 | 8 | 7 | 8 | 7 |
| TCGA-A6-2678 | 8 | 7 | 8 | 6 |
| TCGA-CM-6676 | 8 | 6 | 7 | 5 |
| TCGA-A6-2684 | 8 | 7 | 8 | 7 |
| TCGA-AA-A01Q | 8 | 7 | 8 | 7 |
| TCGA-A6-5665 | 8 | 7 | 8 | 6 |
| TCGA-AA-3833 | 8 | 7 | 8 | 7 |
| TCGA-D5-5538 | 8 | 7 | 8 | 7 |
| TCGA-A6-5660 | 8 | 6 | 8 | 6 |
| TCGA-A6-6651 | 8 | 7 | 7 | 6 |
| TCGA-DM-A1D0 | 8 | 6 | 7 | 5 |
| TCGA-F4-6855 | 8 | 6 | 7 | 6 |
| TCGA-AD-5900 | 8 | 7 | 8 | 7 |
| TCGA-5M-AAT4 | 8 | 7 | 8 | 6 |
| TCGA-D5-6932 | 8 | 7 | 8 | 7 |
| TCGA-CM-5349 | 8 | 6 | 7 | 6 |
| TCGA-F4-6854 | 8 | 7 | 8 | 6 |
| TCGA-G4-6303 | 8 | 6 | 7 | 6 |
| TCGA-AM-5820 | 8 | 6 | 7 | 6 |
| TCGA-A6-5667 | 8 | 6 | 8 | 6 |
| TCGA-AY-6196 | 8 | 8 | 8 | 8 |
| TCGA-CK-4951 | 8 | 7 | 8 | 7 |
| TCGA-AA-A01X | 8 | 6 | 7 | 5 |
| TCGA-AA-A00O | 8 | 7 | 8 | 6 |
| TCGA-D5-5540 | 8 | 6 | 7 | 6 |
| TCGA-A6-2675 | 8 | 7 | 8 | 7 |
| TCGA-AA-3713 | 8 | 7 | 8 | 7 |
| TCGA-CM-6168 | 8 | 7 | 8 | 7 |
| TCGA-CM-6166 | 8 | 6 | 7 | 5 |
| TCGA-AZ-5403 | 8 | 6 | 7 | 6 |
| TCGA-AA-3492 | 8 | 7 | 8 | 7 |
| TCGA-A6-3809 | 8 | 8 | 8 | 8 |
| TCGA-AA-3543 | 8 | 8 | 8 | 7 |
| TCGA-D5-5539 | 8 | 7 | 8 | 6 |
| TCGA-D5-6922 | 8 | 7 | 8 | 7 |
| TCGA-D5-5541 | 8 | 7 | 7 | 6 |
| TCGA-A6-6780 | 8 | 8 | 8 | 8 |
| TCGA-CA-6716 | 8 | 6 | 7 | 5 |
| TCGA-A6-4105 | 8 | 7 | 8 | 7 |
| TCGA-AZ-6598 | 8 | 7 | 7 | 6 |
| TCGA-3L-AA1B | 8 | 7 | 8 | 6 |
| TCGA-DM-A285 | 8 | 6 | 8 | 5 |
| TCGA-AA-3696 | 8 | 6 | 7 | 5 |
| TCGA-G4-6314 | 8 | 6 | 8 | 6 |
| TCGA-AA-3538 | 8 | 6 | 7 | 5 |
| TCGA-AA-3678 | 8 | 7 | 8 | 7 |
| TCGA-CK-5915 | 8 | 6 | 7 | 5 |
| TCGA-D5-6529 | 8 | 8 | 8 | 7 |
| TCGA-QG-A5Z1 | 8 | 6 | 7 | 5 |
| TCGA-CA-6719 | 8 | 7 | 8 | 7 |
| TCGA-CM-4746 | 8 | 7 | 8 | 6 |
| TCGA-AA-3984 | 8 | 7 | 8 | 7 |
| TCGA-CM-6165 | 8 | 7 | 8 | 6 |
| TCGA-AA-A02F | 8 | 6 | 7 | 5 |
| TCGA-AZ-4315 | 8 | 7 | 7 | 6 |
| TCGA-AA-3534 | 8 | 6 | 7 | 5 |
| TCGA-A6-5664 | 8 | 7 | 8 | 7 |
| TCGA-D5-6530 | 8 | 7 | 7 | 7 |
| TCGA-AA-3488 | 8 | 7 | 8 | 6 |
| TCGA-A6-A56B | 8 | 6 | 7 | 5 |
| TCGA-G4-6586 | 8 | 7 | 8 | 7 |
| TCGA-A6-2682 | 8 | 6 | 7 | 6 |
| TCGA-G4-6311 | 8 | 7 | 8 | 6 |
| TCGA-AA-3509 | 8 | 7 | 8 | 6 |
| TCGA-CM-6169 | 8 | 7 | 8 | 7 |
| TCGA-A6-2671 | 8 | 7 | 8 | 7 |
| TCGA-G4-6307 | 8 | 6 | 8 | 6 |
| TCGA-CM-4747 | 8 | 7 | 8 | 6 |
| TCGA-A6-2674 | 8 | 8 | 8 | 7 |
| TCGA-CK-4948 | 8 | 6 | 8 | 6 |
| TCGA-AD-6964 | 8 | 8 | 8 | 8 |
| TCGA-AM-5821 | 8 | 9 | 8 | 8 |
| TCGA-AA-3496 | 8 | 8 | 8 | 7 |
| TCGA-NH-A8F7 | 8 | 6 | 8 | 6 |
| TCGA-AA-3494 | 8 | 7 | 8 | 6 |
| TCGA-D5-6536 | 8 | 6 | 8 | 6 |
| TCGA-A6-6781 | 8 | 7 | 7 | 7 |
| TCGA-A6-2672 | 8 | 8 | 8 | 8 |
| TCGA-AD-6901 | 8 | 7 | 8 | 7 |
| TCGA-CM-5348 | 8 | 6 | 7 | 6 |
| TCGA-CM-6170 | 8 | 7 | 8 | 7 |
| TCGA-AA-3542 | 8 | 6 | 8 | 6 |
| TCGA-AA-3521 | 8 | 7 | 8 | 6 |
| TCGA-D5-6534 | 8 | 8 | 8 | 8 |
| TCGA-D5-6537 | 8 | 7 | 8 | 6 |
| TCGA-CM-6162 | 8 | 8 | 8 | 7 |
| TCGA-AA-3489 | 8 | 8 | 8 | 7 |
| TCGA-A6-6142 | 8 | 6 | 7 | 6 |
| TCGA-AA-3554 | 8 | 8 | 8 | 7 |
| TCGA-NH-A50V | 8 | 7 | 8 | 7 |
| TCGA-AA-3864 | 8 | 7 | 8 | 6 |
| TCGA-F4-6570 | 8 | 7 | 7 | 7 |
| TCGA-D5-6928 | 8 | 9 | 8 | 9 |
| TCGA-CM-6171 | 8 | 6 | 7 | 6 |
| TCGA-AA-A017 | 8 | 6 | 8 | 6 |
| TCGA-AA-3692 | 8 | 7 | 8 | 6 |
| TCGA-AA-3831 | 8 | 6 | 7 | 6 |
| TCGA-D5-7000 | 8 | 7 | 8 | 7 |
| TCGA-AA-A00R | 8 | 8 | 8 | 8 |
| TCGA-F4-6459 | 8 | 6 | 7 | 6 |
| TCGA-AA-3664 | 8 | 7 | 8 | 7 |
| TCGA-D5-6924 | 8 | 7 | 8 | 7 |
| TCGA-D5-6538 | 8 | 6 | 7 | 5 |
| TCGA-AA-A00N | 8 | 7 | 8 | 7 |
| TCGA-AA-3529 | 8 | 6 | 8 | 6 |
| TCGA-AA-3867 | 8 | 7 | 8 | 6 |
| TCGA-AA-3949 | 8 | 9 | 8 | 8 |
| TCGA-F4-6463 | 8 | 6 | 8 | 6 |
| TCGA-CM-5862 | 8 | 7 | 8 | 6 |
| TCGA-A6-2685 | 8 | 7 | 8 | 7 |
| TCGA-D5-6923 | 8 | 6 | 7 | 6 |
| TCGA-5M-AAT6 | 8 | 8 | 8 | 8 |
| TCGA-F4-6805 | 8 | 7 | 8 | 7 |
| TCGA-DM-A28G | 8 | 7 | 8 | 6 |
| TCGA-F5-6864 | 8 | 7 | 8 | 6 |
| TCGA-AF-2690 | 8 | 7 | 8 | 7 |
| TCGA-CI-6619 | 8 | 7 | 8 | 7 |
| TCGA-DT-5265 | 8 | 6 | 8 | 6 |
| TCGA-AH-6549 | 8 | 7 | 8 | 6 |
| TCGA-DC-6156 | 8 | 7 | 7 | 6 |
| TCGA-F5-6810 | 8 | 6 | 7 | 6 |
| TCGA-EI-7002 | 8 | 7 | 8 | 6 |
| TCGA-G5-6572 | 8 | 6 | 7 | 6 |
| TCGA-DC-6158 | 8 | 7 | 8 | 7 |
| TCGA-AF-3913 | 8 | 6 | 7 | 6 |
| TCGA-AG-3578 | 8 | 6 | 7 | 6 |
| TCGA-AG-3894 | 8 | 7 | 8 | 6 |
| TCGA-CI-6621 | 8 | 7 | 8 | 7 |
| TCGA-AG-3600 | 8 | 7 | 8 | 7 |
| TCGA-AH-6897 | 8 | 7 | 8 | 6 |
| TCGA-EI-6917 | 8 | 7 | 8 | 7 |
| TCGA-AG-A01Y | 8 | 7 | 8 | 7 |
| TCGA-F5-6702 | 8 | 7 | 7 | 6 |
| TCGA-AG-4001 | 8 | 7 | 8 | 6 |
| TCGA-EI-6508 | 8 | 6 | 8 | 6 |
| TCGA-AH-6644 | 8 | 6 | 8 | 6 |
| TCGA-AF-2687 | 8 | 7 | 8 | 7 |
| TCGA-AF-2692 | 8 | 7 | 8 | 6 |
| TCGA-AG-3731 | 8 | 7 | 8 | 7 |
| TCGA-AG-3742 | 8 | 6 | 8 | 6 |
| TCGA-F5-6863 | 8 | 6 | 7 | 5 |
| TCGA-CL-5917 | 8 | 6 | 7 | 5 |
| TCGA-F5-6464 | 8 | 7 | 7 | 7 |
| TCGA-AF-A56N | 8 | 7 | 8 | 6 |
| TCGA-AF-5654 | 8 | 6 | 7 | 6 |
| TCGA-AG-3999 | 8 | 6 | 7 | 6 |
| TCGA-EI-6509 | 8 | 6 | 8 | 6 |
| TCGA-EI-6511 | 8 | 8 | 8 | 7 |
| TCGA-DC-6155 | 8 | 7 | 8 | 6 |
| TCGA-AG-3601 | 8 | 6 | 8 | 6 |
| TCGA-DY-A0XA | 8 | 7 | 8 | 6 |
| TCGA-AH-6643 | 8 | 7 | 9 | 7 |
| TCGA-AF-A56K | 8 | 6 | 8 | 6 |
| TCGA-AG-3584 | 8 | 7 | 8 | 6 |
| TCGA-EI-7004 | 8 | 7 | 8 | 7 |
| TCGA-AF-6655 | 8 | 7 | 8 | 7 |
| TCGA-AA-3555 | 7 | 6 | 7 | 5 |
| TCGA-D5-6926 | 7 | 7 | 7 | 6 |
| TCGA-A6-2686 | 7 | 7 | 7 | 7 |
| TCGA-CA-6717 | 7 | 6 | 7 | 6 |
| TCGA-D5-6898 | 7 | 6 | 7 | 5 |
| TCGA-AA-A010 | 7 | 6 | 8 | 6 |
| TCGA-F4-6703 | 7 | 7 | 7 | 7 |
| TCGA-G4-6298 | 7 | 5 | 6 | 5 |
| TCGA-AA-3663 | 7 | 6 | 7 | 6 |
| TCGA-A6-A566 | 7 | 6 | 7 | 6 |
| TCGA-AZ-6601 | 7 | 7 | 7 | 7 |
| TCGA-CK-6748 | 7 | 6 | 7 | 5 |
| TCGA-DM-A1HB | 7 | 6 | 7 | 5 |
| TCGA-A6-6654 | 7 | 7 | 7 | 7 |
| TCGA-CK-4952 | 7 | 6 | 7 | 5 |
| TCGA-AA-A00J | 7 | 6 | 7 | 5 |
| TCGA-AZ-6607 | 7 | 7 | 7 | 7 |
| TCGA-AA-A01D | 7 | 5 | 7 | 5 |
| TCGA-AA-3966 | 7 | 6 | 7 | 6 |
| TCGA-DM-A28H | 7 | 6 | 7 | 5 |
| TCGA-AA-3815 | 7 | 7 | 7 | 7 |
| TCGA-NH-A5IV | 7 | 6 | 6 | 5 |
| TCGA-AA-3950 | 7 | 7 | 7 | 7 |
| TCGA-AG-3574 | 7 | 6 | 7 | 5 |
| TCGA-EI-6885 | 7 | 6 | 6 | 6 |
| TCGA-F5-6814 | 7 | 7 | 7 | 7 |
| TCGA-AG-3901 | 7 | 6 | 7 | 6 |
| TCGA-DY-A1H8 | 7 | 6 | 7 | 5 |
| TCGA-AG-4021 | 7 | 5 | 6 | 5 |
| TCGA-AG-A002 | 7 | 5 | 7 | 5 |
| TCGA-AF-3400 | 7 | 7 | 7 | 7 |
| TCGA-D5-6927 | 6 | 6 | 6 | 6 |
| TCGA-WS-AB45 | 6 | 6 | 6 | 6 |
| TCGA-SS-A7HO | 6 | 5 | 6 | 5 |
| TCGA-AD-A5EJ | 6 | 5 | 6 | 5 |
| TCGA-AA-3947 | 6 | 5 | 6 | 5 |
| TCGA-EI-6507 | 6 | 6 | 6 | 6 |
